# Evolution of *hedgehog *and *hedgehog*-related genes, their origin from Hog proteins in ancestral eukaryotes and discovery of a novel Hint motif

**DOI:** 10.1186/1471-2164-9-127

**Published:** 2008-03-11

**Authors:** Thomas R Bürglin

**Affiliations:** 1 Dept. of Biosciences and Nutrition, Karolinska Institutet & School of Life Sciences, Södertörns Högskola, Alfred Nobels Allé 7, SE-141 89 Huddinge, Sweden

## Abstract

**Background:**

The Hedgehog (Hh) signaling pathway plays important roles in human and animal development as well as in carcinogenesis. Hh molecules have been found in both protostomes and deuterostomes, but curiously the nematode *Caenorhabditis elegans *lacks a bona-fide Hh. Instead a series of Hh-related proteins are found, which share the Hint/Hog domain with Hh, but have distinct N-termini.

**Results:**

We performed extensive genome searches such as the cnidarian *Nematostella vectensis *and several nematodes to gain further insights into Hh evolution. We found six genes in *N. vectensis *with a relationship to Hh: two Hh genes, one gene with a Hh N-terminal domain fused to a Willebrand factor type A domain (VWA), and three genes containing Hint/Hog domains with distinct novel N-termini. In the nematode *Brugia malayi *we find the same types of *hh*-related genes as in *C. elegans*. In the more distantly related Enoplea nematodes Xiphinema and *Trichinella spiralis *we find a bona-fide Hh. In addition, *T. spiralis *also has a *quahog *gene like *C. elegans*, and there are several additional *hh*-related genes, some of which have secreted N-terminal domains of only 15 to 25 residues. Examination of other Hh pathway components revealed that *T. spiralis* - like *C. elegans* - lacks some of these components. Extending our search to all eukaryotes, we recovered genes containing a Hog domain similar to Hh from many different groups of protists. In addition, we identified a novel Hint gene family present in many eukaryote groups that encodes a VWA domain fused to a distinct Hint domain we call Vint. Further members of a poorly characterized Hint family were also retrieved from bacteria.

**Conclusion:**

In Cnidaria and nematodes the evolution of *hh *genes occurred in parallel to the evolution of other genes that contain a Hog domain but have different N-termini. The fact that Hog genes comprising a secreted N-terminus and a Hog domain are found in many protists indicates that this gene family must have arisen in very early eukaryotic evolution, and gave rise eventually to *hh *and *hh*-related genes in animals. The results indicate a hitherto unsuspected ability of Hog domain encoding genes to evolve new N-termini. In one instance in Cnidaria, the Hh N-terminal signaling domain is associated with a VWA domain and lacks a Hog domain, suggesting a modular mode of evolution also for the N-terminal domain. The Hog domain proteins, the inteins and VWA-Vint proteins are three families of Hint domain proteins that evolved in parallel in eukaryotes.

## Background

The Hedgehog (Hh) signaling pathway has been shown to be of fundamental importance for patterning and cell proliferation in animal development (for review see [1-4]). Mutations in this pathway cause congenital defects and several types of cancer such as basal cell carcinoma and medulloblastoma [5-8]. A key molecule of the pathway is Hh, a secreted ligand that can act as morphogen. *Drosophila melanogaster *has a single *hedgehog *(*hh*) gene, while mammalian genomes contain three paralogous genes, Sonic Hh (Shh), Desert Hh (Dhh), and Indian Hh (Ihh) [9]. In zebrafish, five *hh *genes are present due to an extra round of genome duplication during evolution of ray-finned fish [10,11]. The Hh protein is synthesized as a precursor composed of two domains, the N-terminal signaling domain and the C-terminal autoprocessing domain. A substantial part of the autoprocessing domain shares sequence similarity with self-splicing inteins and therefore this domain has been named Hint [12]. C-terminal to the Hint domain is a sterol recognition region (SRR). A crucial function of the autoprocessing domain is to add a cholesterol moiety to the N-terminal signaling domain, which is required for the proper function of the N-terminal ligand [13-16]. In the nematode *Caenorhabditis elegans *no bona-fide *hh *is present, i.e. there is no gene that encodes both the N-terminal signalling domain as well as the C-terminal Hint domain. Instead ten genes encoding the C-terminal autoprocessing domain are found that, however, have N-terminal regions very distinct from Hh. Furthermore, a large number of additional genes are found that encode only these new N-terminal domains and lack the C-terminal autoprocessing domain. Overall, these genes can be grouped into four families that have been named *quahog *(*qua*), *warthog *(*wrt*), *groundhog *(*grd*) and *ground-like *(*grl*) and are collectively referred to as *hh*-related genes [17-19]. At present it is not clear, whether the C-terminal domains of the *C. elegans *Hh-related proteins can add a cholesterol moiety to the N-terminus analogous to Hh, since there are sequence differences in the SRR equivalent region. Therefore, this region of the Hh-related proteins was named ARR (adduct recognition region) [20]; here we refer to the combined Hint/SRR or Hint/ARR region as Hog domain for simplicity, as others have done as well [21].

The N-terminal domains of the *C. elegans hh*-related genes were not found in vertebrates and flies using blast searches, giving rise to the notion that these genes were perhaps derived from *hh *in early nematode evolution [17,18]. Recently, a Hog domain containing protein, Hoglet, was discovered in the choanoflagellate *Monosiga ovata*, but its N-terminal region is distinct from Hh and other Hh-related proteins, instead sharing sequence similarity with cellulose-binding domains (CBD) [22]. Choanoflagellates are unicellular protists most closely related to multicellular animals [23,24] and therefore Hoglet might represent an ancestral precursor form of Hh. A Hh protein was also described from the cnidarian *Nematostella vectensis *[25,26], indicating that Hh already existed before the rise of bilaterian animals. An EST with sequence similarity to Hh was also recovered from the sponge Oscarella carmela [27], indicating that the "Hedge" domain originated before the advent of Eumetazoa. In order to understand the origin and evolution of the *C. elegans hh*-related genes, we had already performed cursory searches of the genome of the parasitic nematode *Brugia malayi *and found that it also contains several *hh*-related genes [18,17]. Here we performed comprehensive searches of the genomes of the cnidarian *N. vectensis *[28], the nematodes *B. malayi *and *Trichinella spiralis *as well as the NCBI protein, DNA and EST databases to find additional *hh *and *hh*-related genes that may shed light on the evolution of these genes. In these searches we found a previously described gene from the fungus *Glomus mosseae *that shares sequence similarity with Hh through the Hog domain [29], but has not been considered in recent evolutionary analyses [22]. Furthermore, we found a number of additional genes with similarity to the Hog domain in Alveolata, moss, red algae, and other protists, indicating that the origin of the Hog domain occurred already in lower eukaryotes. As stated above, the Hog domain shares sequence similarity to self-splicing inteins, which have been found in Archaea, Bacteria, as well as fungi, algae and a few protists [30-32]. Recently, two other types of Hint related domains have been described, primarily from bacteria, that have been named bacterial intein-like proteins (BIL) type A and B [21,33]. Several conserved sequence motifs within the Hint domain have been described for inteins that have been named motif A, B, E and F [34-37]. Our searches revealed also ORFs in Tetrahymena, fungi and several other protist branches that have similarity to the Hint domain via motifs A and B, but cannot be classified as inteins, Hog, or BIL domains.

## Results

### Retrieval and analysis of sequences

**Figure 1:**
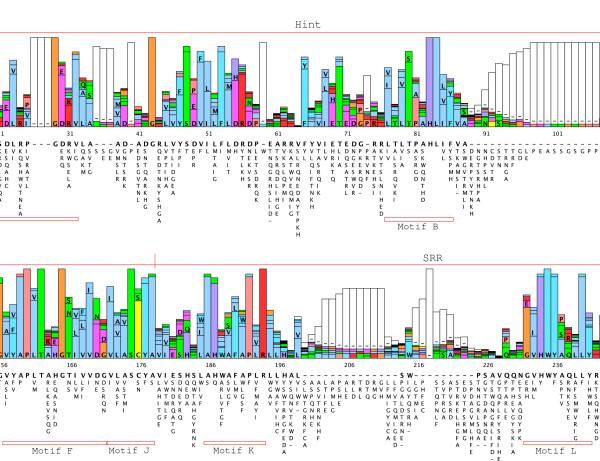
**Protein sequence logo of Hh Hog domains**. Central section of the protein sequence logo that was generated from aligned Hog domains of diverse Hh proteins using LogoBar. For the full image see Additional file 2. The color scheme is similar to the one used in the multiple sequence alignments (N,Q,S,T: green; C: yellow; P: pink; G: orange; K,R: red; A,I,L,M,V: blue; F,W,Y: cyan blue; H, purple, D,E: magenta; gaps: white). The extend of the Hint domain and the SRR region are indicated above the logo with a red line. Red boxes underneath the logo indicate the different motifs A, B, F, J, K, L.

**Figure 2:**
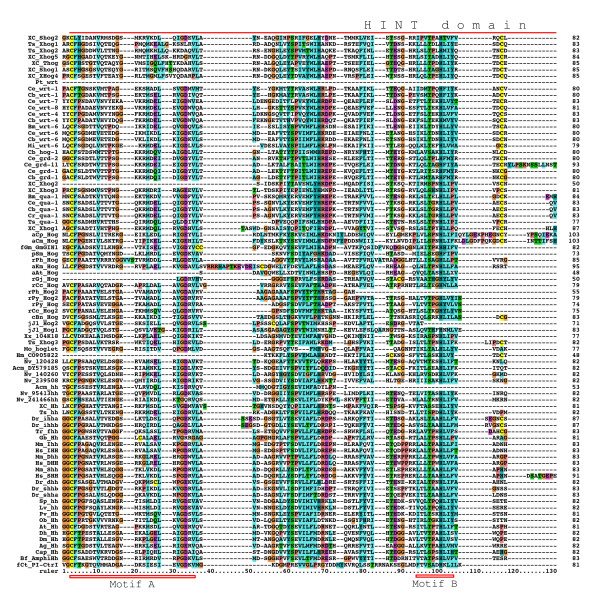
**Multiple sequence alignment of Hog domains, part 1**. Multiple sequence alignment in this and other figures was carried out using first MUSCLE and then imported into Clustal_X. Manual adjustments to the alignment were carried out using SeaView. Color coding was modified from default Clustal_X color coding by marking all cysteine residues in yellow, small hydrophobic residues in light blue and large hydrophobic residues in cyan blue. The Hint domain, as well as the C-terminal SRR or ARR regions are indicated above the alignment. Motifs A, B, F, J, K, and L are indicated with red rectangles underneath the alignment. Species abbreviations are shown in Table 3. Note that not all sequences in this alignment are complete.

**Figure 3:**
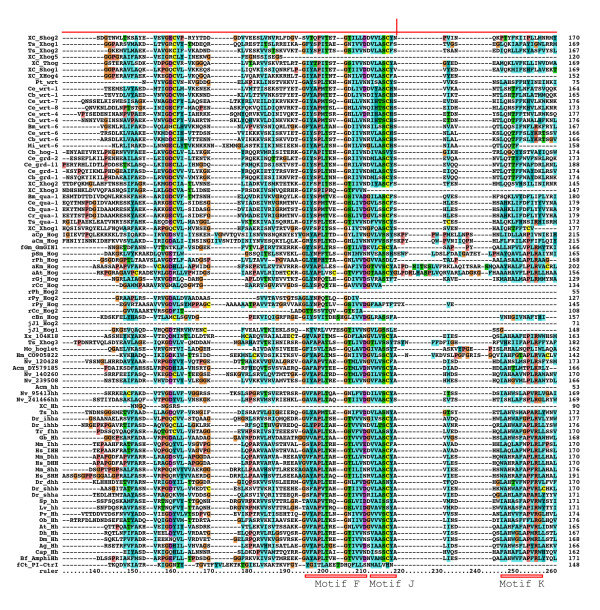
**Multiple sequence alignment of Hog domains, part 2**. Continuation of the multiple sequence alignment of Figure 2.

**Figure 4:**
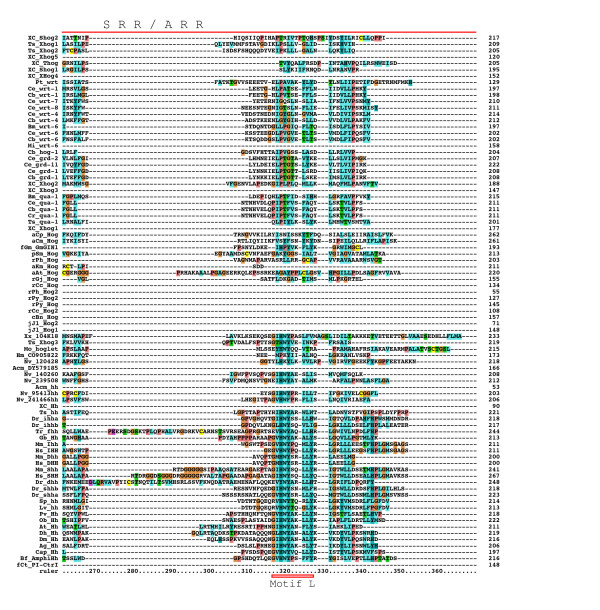
**Multiple sequence alignment of Hog domains, part 3**. Continuation of the multiple sequence alignment of Figure 3.

We have previously characterized one *qua*, one *hog*-only, ten *wrt*, 17 *grd*, and 32 *grl *ORFs from *C. elegans*, three of which are pseudogenes [19,38]. Furthermore, we have identified 49 *hh*-related genes in the related nematode *Caenorhabditis briggsae *[38]. We correct this number to 48 *hh*-related genes here, because *C. briggsae wrt-8 *is the same locus as *wrt-4*. To retrieve sequences from other species we used selected Hh, WRT, QUA, GRD and GRL protein sequences as queries for tblastn and blastp searches at Stellabase, the DOE Joint Genome Institute, The Genome Sequencing Center at the Washington University School of Medicine, The Institute for Genomic Research (TIGR), and NCBI (see Methods). The recovered sequences were aligned to sequences that we had assembled previously [18,19,38]. When obvious discrepancies in conserved regions were found in the newly retrieved ORFs, genomic sequences were inspected for additional or extraneous exons or alternative splice sites, and ESTs were examined for frameshifts. ORFs were corrected to optimize matches to existing motifs, and extraneous N-terminal residues were truncated when methionine residues followed by good N-terminal signal peptides for secretion were found. One caveat is that our ORF predictions from genomic sequences are still limited due to partial nature of the various contig assemblies. In some instances an ORF runs into an unsequenced region (e.g., *B. malayi wrt-4*). In the case of ESTs it was often possible to assemble several ESTs into contigs, but in most instances ORFs derived from ESTs lack either N-terminus and/or C-terminus. Considering also that the various genome projects are are in different states of completion, the nomenclature given here to the ORFs should be considered preliminary. After correction of the ORFs multiple sequence alignments of the different protein domains were made and used for phylogenetic analyses using Neighbor Joining and Maximum Likelihood. We also prepared protein sequence logos of the Hog domains of Hh and nematode Hh-related genes to aid with the analysis of more divergent Hog domains (Figure 1, Additional files 1, 2, 3). We extended the motif nomenclature of inteins by introducing motifs J, K, and L (Figure 1, Additional file 2). Motif J corresponds to motif G in inteins [34-37], however, because this motif is so distinct in Hog domains, we have ventured to give it its own name here. Motifs K and L are located in the SRR and are primarily found in Hog domains of Hh proteins (Figure 2, 3, 4, Additional file 1). In the Hog domains of nematode Hh-related proteins, these two regions show a number of differences compared to the Hh proteins (Figure 2, 3, 4, Additional files 1, 2, 3), and, as will be shown below, motifs K and L provide useful diagnostic functions for evaluating Hog domains.

### *hh*-related genes in *B. malayi *and other Chromadorea

**Figure 5:**
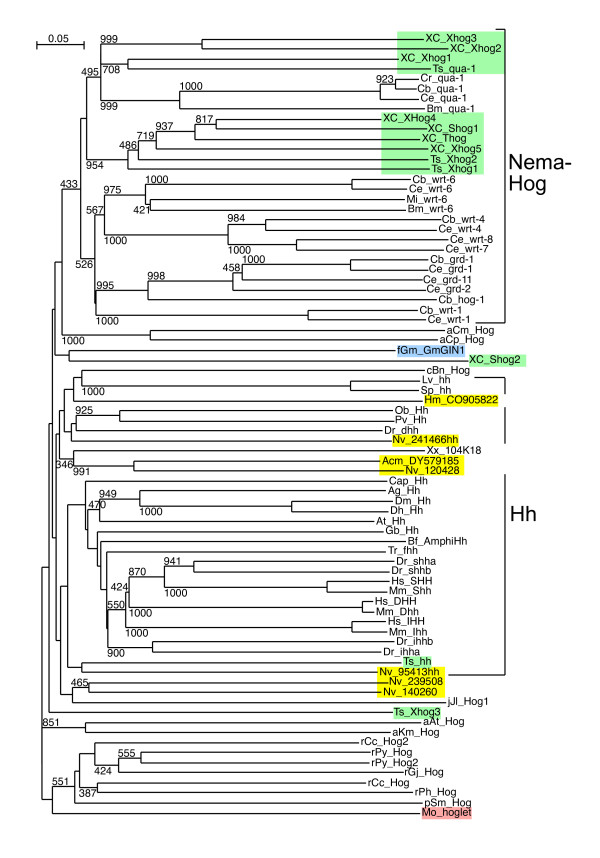
**Phylogenetic tree of Hog domains**. Phylogenetic trees were built from aligned Hog domains (Figure 2 – 4). The Neighbor joining tree was created using the default settings of Clustal_X. Bootstrap values of 1000 trials are indicated in the figure. In this and subsequent phylogenetic tree figures Enoplea sequences are highlighted in light green, Cnidarian sequences in yellow, Choanoflagellate sequences in light red and fungal sequences in blue. The Hh sequences are marked with Hh and the nematode Hh-related sequences are marked with NemaHog. The root was placed between the red algae/plant sequences and the remaining sequences. Some incomplete sequences were omitted in this tree. Additional phylogenetic analyses were also carried out, for example by omitting the protist sequences and using the fungal sequence GmGIN1 as outgroup (Additional files 5, 6, 7). Overall, the results were very similar.

**Figure 6:**
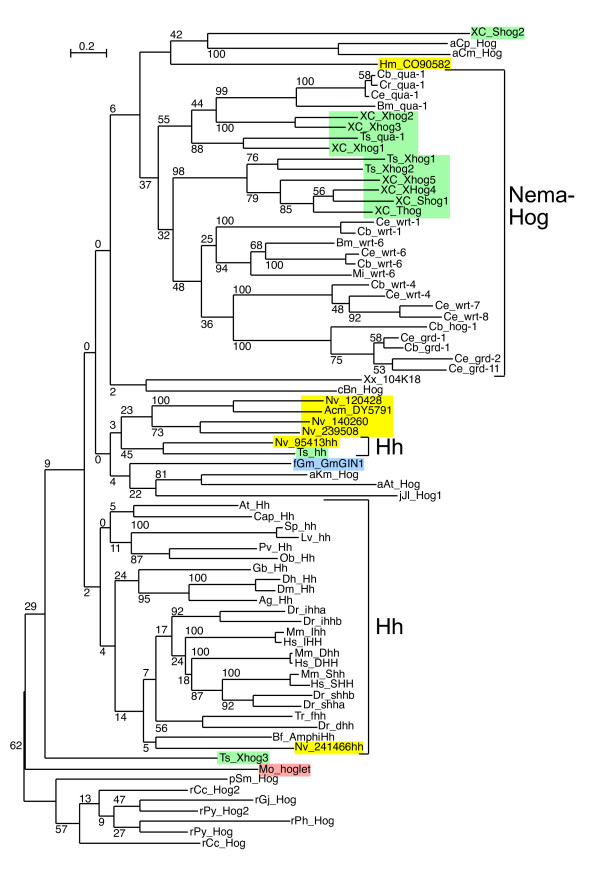
**Maximum likelihood phylogenetic tree of Hog domains**. A Maximum likelihood phylogenetic tree was constructed using the same data as in Figure 5. Phyml default values were used, and bootstrap values for 100 trials are shown.

**Figure 7:**
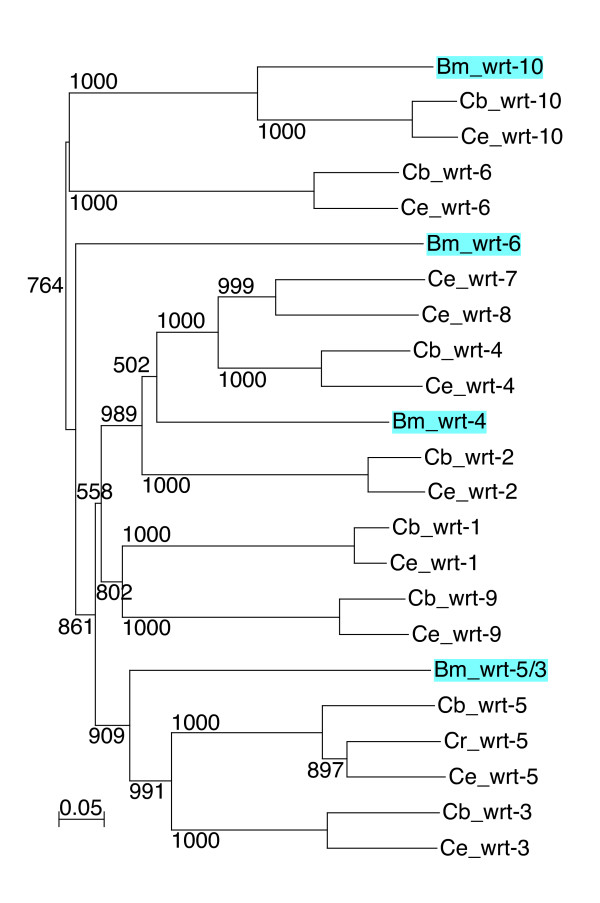
**Phylogenetic tree of Wart domains**. A multiple sequence alignment of Wart domains (see Additional file 4) was used to generate at Neighbor joining tree with the default settings of Clustal_X. *B. malayi *sequences are highlighted in light blue. This tree is unrooted. Results of 1000 bootstrap trials are shown.

The nematode *B. malayi *is a parasitic nematode that is one of the more distantly related members to *C. elegans *within the order of Rhabditiada [39]. We have previously described a *quahog *gene, *qua-1*, in *B. malayi *[19], and obtained partial sequences from ESTs for two *wrt*, one *grd *and two *grl *genes [17]. Here we retrieved a total of four *wrt *genes, one with a Hog domain (Bm *wrt-6*), two without a Hog domain (Bm *wrt-10*, Bm *wrt-5/3*) and one whose C-terminus is presently unknown (Bm *wrt-4*) (Table 1, Fig. 2, 3, 4, Additional file 4). Based on phylogenetic analyses of both the Hog domain and the Wart domain (Figure 5, 6, 7, Additional files 5, 6, 7), Bm *wrt-10 *is a clear orthologue of Ce *wrt-10*, *wrt-5/3 *is a co-orthologue of Ce *wrt-5 *and *wrt-3*, and – based primarily on the Hog domain – Bm *wrt-6 *is an orthologue of Ce *wrt-6*. The *wrt-6 *ORF encodes a full Wart domain, however the previously identified *wrt-6 *EST [17] lacks the C-terminal half of the Wart domain. Comparison with the genomic sequence revealed that this EST spans exons 1, 2, the first 10 nucleotides of exon 3 and continues then into exon 10, 11, and 12, which contain the Hog domain (data not shown). The point of discrepancy in exon 3 is not at a splice site, therefore this unusual EST might represent a cloning artifact. Bm *wrt-4 *cannot be assigned unequivocally as orthologue of Cb *wrt-2 *or Cb *wrt-4*, but it appears to group with them. Ce *wrt-10 *lies next to Ce *wrt-1 *on the chromosome [17], however the Bm *wrt-10 *contig is too small to determine, whether another *wrt *gene resides next to it.

**Table 1:** Number of *hh *and *hh*-related genes found in different species.

Gene structure	Nv	XC	Ts	Bm	Ce	Cb	Dm	Mm
Hedgehog	2	1	1		-	-	1	3
Hedge-VWA	1							
Wart-only				2	5	5		
Warthog				1 + 1?	5	3		
Ground-only				1	13 (1P)	10		
Groundhog					3	1		
Ground-like				13	30 (2P)	27		
Quahog		3?	1	1	1	1		
Hog only					1	1		
Y0-hog	1							
Y1-hog	1							
Y2-hog	1							
Enop-hog		1	2					
T-hog		1						
Short-hog		2	1					
Unknown hog		1						
Total	6	9	5	19	58 (3P)	48	1	3

The left column indicates the gene structure, with Hog referring to the combined Hint/SRR or Hint/ARR domain. Known pseudogenes are indicated in brackets.

**Figure 8:**
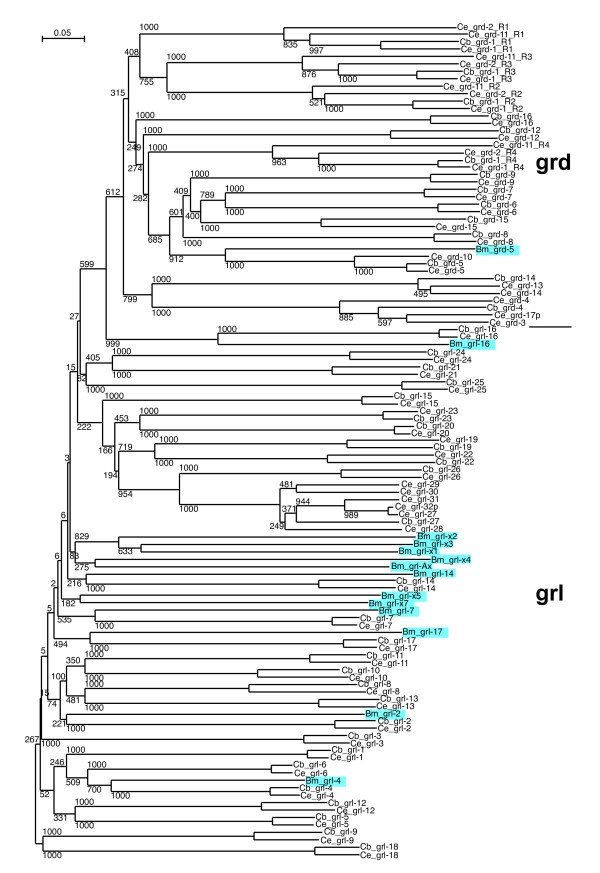
**Phylogenetic tree of Ground and Ground-like domains**. A multiple sequence alignment of Ground and Ground-like domains (see Additional file 8) was used to generate a Neighbor joining tree with the default settings of Clustal_X. For *grd-1*, *grd-2 *and *grd-11 *the four Ground domains were extracted manually prior to alignment; the R1 to R4 postscripts indicate the repeat number. *B. malayi *sequences are highlighted in light blue. This tree is unrooted. Results of 1000 bootstrap trials are shown.

One Ground domain gene was recovered from *B. malayi*, Bm *grd-5*, that is co-orthologous to Ce *grd-5 *and *grd-10 *(Figure 8, Additional file 8). We have previously identified a few *grl *genes from *B. malayi *in searches of ESTs [17]. Here, thirteen *grl *genes were recovered from *B. malayi*, however, only a few could be identified as orthologues of *C. elegans *genes, i.e. *grl-4*, *grl-16*, and perhaps *grl-7 *and *grl-17 *(Figure 8, Additional file 8). Other *grl *genes have clearly duplicated within the *B. malayi *branch, e.g. Bm *grl-x1*, *gr l-x2 *and *grl-3*, which are more similar to each other than to other genes.

A few ESTs were retrieved from other Chromadorea nematodes: In *Meloidogyne incognita *we found a gene with similarity to *wrt-6 *(Figure 2, 3, 4, 5, 6), and one *grl *gene, Msp3, which is expressed in the esophagal gland cells [40] (Additional file 8). In *Parastrongyloides trichosuri *a gene with similarity to *wrt *genes was found (Additional file 7).

### *hh *and *hh*-related genes in Enoplea nematodes: *Xiphinema sp*. and *Trichinella spiralis*

**Figure 9:**
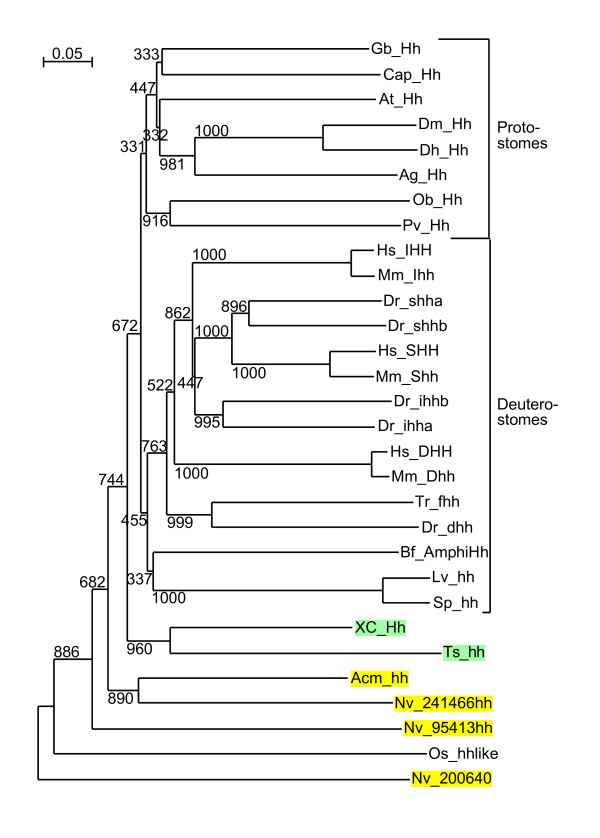
**Phylogenetic tree of Hedge domains**. A multiple sequence alignment of Hedge and Hedgehog proteins (see Additional file 10) was used to generate at Neighbor joining tree with the default settings of Clustal_X. This tree is unrooted. Results of 1000 bootstrap trials are shown.

**Figure 10:**
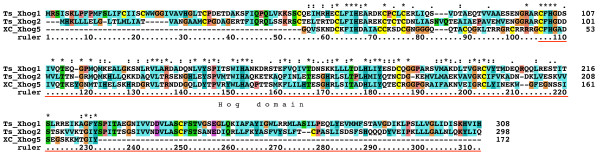
**Multiple sequence alignment of Enoplea Hog proteins with a new upstream motif**. Multiple sequence alignment of Enoplea Ts Xhog1, Ts Xhog2, and XC Xhog5 reveals new conserved regions upstream of the Hog domain.

*C. elegans *and *B. malayi *belong to the class of Chromadorea. Our database searches revealed now also Hog-containing genes from the distantly related class of Enoplea nematodes, i.e. *Xiphinema *index CSEQDL01, and *T. spiralis*, both members of the Dorylaimia [39]. From *Xiphinema *we retrieved ESTs for nine distinct genes, and from *T. spiralis *five (Table 1), one of which (Ts Xhog1) is also supported by ESTs. All five *T. spirals *ORFs have a signal peptide sequence for secretion, and although many of the Xiphinema ESTs are incomplete, in several instances methionine residues followed by good signal peptides could be found at the 5' of the ESTs (XC Thog, Shog1, Shog2) (Additional file 9). One gene from Xiphinema (XC hh) and one gene from *T. spiralis *(Ts hh) are clearly *hh *genes (Figure 2, 3, 4, 9, Additional file 10), since they both have a Hedge domain and a Hog domain. One gene from *T. spiralis *has a QUA domain upstream of the Hog domain (Ts qua-1). While its Qua domain is rather divergent, the cysteine residues are all conserved (Additional file 11). Ts Xhog3 has a rather short region upstream of the Hog domain, which cannot be extended, because it is delimited by an upstream cyclin gene, for which ESTs are available (data not shown). After cleavage of the signal peptide and subsequent autoprocessing through the Hog domain the predicted N-terminal peptide of Ts Xhog3 would only be 34 residues long. In Xiphinema the three ORFs with a signal peptide (XC Shog1, Shog2, and Thog) have rather short predicted N-terminal sequences as well. In the case of XC Shog1 it is only 15 residues long, in the case of XC Shog2 it is 25 residues, and in the case of XC Thog it is 79 residues long with an unusual stretch of about 70 residues almost entirely composed of threonine and serine residues. Two *T. spiralis *genes reside next to each other on the chromosome (Ts Xhog1 and Xhog2). They share sequence similarity upstream of the Hog domain with six conserved cysteine residues. In addition, XC Xhog5 also has sequence similarity to the upstream regions of Ts Xhog1 and Xhog 2 (Figure 10).

Apart from the similarities in the regions N-terminal to the Hog domain indicated above, the remaining N-terminal sequences show no obvious similarities between each other or to any other proteins. Only the threonine-rich stretch is reminiscent of the 200 residue long threonine stretch in the N-terminal region of the choanoflagellate Hoglet protein [22]. However, this may be a case of convergent evolution. No Wart, Ground, or Ground-like domains could be detected in the genome of *T. spiralis *or in EST database searches restricted to Enoplea.

Based on phylogenetic analyses of the Hog domains, XC Xhog1, Xhog2, Xhog3, and Ts Qua-1 form a clade with the Quahog proteins (Figure 5, 6, Additional files 5, 6, 7). While N-terminal sequences for XC Xhog1, 2 and 3 are lacking they could be bona-fide Quahog proteins. A second, distinct clade is formed by Ts Xhog1, Xhog2, and XC Shog1, Xhog4, Xhog5 and Thog, indicating that they are derived from a common ancestor (Figure 5, 6, Additional files 5, 6, 7). In three cases, Ts Xhog1, Xhog2 and XC Xhog5), a common upstream sequence (Enop) has been identified (Figure 10), which seems to be specific to Enoplea nematodes, suggesting that at least in the cases of XC Shog1 and Thog the N-terminal regions have diverged relatively recently.

Almost all nematode Hh-related proteins form a distinct clade, the only exception being the Hh proteins, and Ts Xhog3 and XC S2hog, which are both very divergent and do not fall into the Hh clade of genes either (Figure 5, 6, Additional files 5, 6, 7). Two features distinguish the Hog domains of the nematode Hh-related proteins from those of the Hh proteins (Figure 1, 2, 3, 4, Additional files 1, 2, 3). 1) The regions corresponding to motifs K and L have characteristic differences in their conserved residues in nematode Hh-related proteins. 2) Two conserved cysteine residues are found in the central region of the Hog domain. When these two residues are mapped onto the X-ray structure of the C-terminal autoprocessing domain of Drosophila Hh [12], it emerges that they lie adjacent to each other and therefore could form a disulfide bond. This feature might stabilize this type of Hog domain in an extracellular environment, and this extra stability might possibly provide some new functionality. It is however not unique to nematode Hog domains. Zebrafish ihha and ihhb and fugu dhh (fhh) also have this extra cysteine pair, which must represent convergent evolution. It is worth pointing out that Ts Hh lacks the two cysteine residues and has motifs K and L as expected from a bona-fide Hh molecule. However, the quite divergent Ts Xhog3 protein, which lacks a Hedge domain, also lacks the cysteine residues and has motifs K and L.

### *hh *and *hh*-related genes in Cnidaria

**Figure 11:**
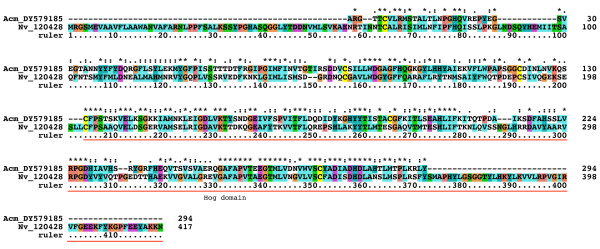
**Multiple sequence alignment of two cnidarian Hog proteins with a new upstream motif**. Pairwise sequence alignment of cnidarian Nv 120428 and Acm DY579185.

tlastn searches of the *N. vectensis *predicted ORFs returned 10 hits. Several turned out to be differently predicted ORF variants most likely derived from the same locus, since corresponding genomic sequences for some of these loci displayed >99% identity. In the end six distinct ORFs were retrieved that all had good signal sequence for secretion. For four of the ORFs ESTs were found that at least partially support the predictions (Table 1, Additional file 9). In the case of Nv 239508 the corresponding genomic region seems to have undergone a recent duplication as two virtually identical Hog domains are present there (Additional file 12). In addition to the *N. vectensis *sequences, ESTs for two genes from *Acropora millepora *and one gene from *Hydra magnipapillata *were identified (Additional file 9). The EST from *H. magnipapillata *could be extended using the blastn of the NCBI trace archives, which also revealed a second, closely related paralogous gene (Additional file 9). Two genes from *N. vectensis *and one from *A. millepora *are bona-fide *hh *genes, because they both encode a Hedge domain and a Hog domain (Figure 2, 3, 4, Additional file 10). Two other ORFs, Nv 120428 and Acm DY579185, share conserved sequences upstream of the Hog domain with at least 3 conserved cysteine residues (Figure 11). The N-termini of Nv 140260 and 239508 do not show any similarity with known motifs, and the processed N-terminal peptide of Nv 140260 is only 86 residues long. Similarly, the upstream region of Hm CO905822 and its close paralog do not shown any similarity to the upstream regions of other cnidarian Hog proteins.

Last but not least, Nv 200640 is predicted to be 3592 amino acids long and is highly unusual. It is similar to the Hh proteins through the N-terminal Hedge domain (blast expected probability: 1e-18 to Ciona Hh), but no Hog domain follows (Additional files 10, 13). The Hedge domain is encoded by two exons, and after an intron of 600 bp many additional exons continue the ORF of the JGI prediction, but nowhere in this genomic region resides a Hog domain. Analysis of the ORF using the SMART server revealed that these extra exons encode multiple motifs with significant sequence similarity to other proteins (Additional file 13). The first motif, encoded by exons 3 and 4, contains a von Willebrand factor (vWF) type A domain (VWA). For example, the VWA domain of chicken collagen, type XIV, alpha 1 (undulin) is retrieved with a blastp probability of 8e-28. After the VWA domain, 21 CA (Cadherin repeat) domains follow, they occur as repeats in extracellular regions and are thought to mediate cell-cell contact when bound to calcium. Further follow two Immunoglobulin C-2 Type domains, two EGF repeats, a transmembrane region, and finally an SH2 domain.

The phylogenetic analysis of the cnidarian Hog domains reveals that they cluster primarily with the Hh Hog domains (Figure 5, 6, Additional files 5, 6, 7), albeit mostly with insignificant bootstrap values. The Hog domain of Nv 241466 Hh has the best similarity to the Hh Hog domains, and clusters with the deuterostome Hh proteins. Nv 140260 and Nv 239508 are most similar to each other, suggesting a likely duplication event within the cnidarian lineage. Nv 120428 and Acm DY579185 may also be related to these two proteins via their Hog domain (Figure 5, 6, Additional files 5, 6, 7), but the bootstrap values are not significant. The Nv 95413 Hh protein is rather divergent, and the Hydra sequence Hm CO905822 is also very divergent and does not from a clade with any of the *N. vectensis *sequences. Therefore, it is not possible to determine, whether all the cnidarian Hog genes originated all from a single ancestral gene in the cnidarian lineage, or whether *hh *and other Hog genes were already present before the split of Cnidaria and Bilateria. The Hedge domains of the three *N. vectensis *ORFs are more divergent than the bilaterian Hedge domains (Figure 9, Additional file 10). The Hedge domain of Nv 241466 Hh is most similar to bilaterian Hh proteins, with a best blast probability of 2e-52 to a fish Hedge domain. Nv 95413 Hh is more divergent, with a best blast probability of 5e-36, and Nv 200640 is the most divergent Hedge domain, with a probability of 1e-18 to a Ciona Hh.

### Hog genes in lower eukaryotes

In order to detect Hh sequences from lower eukaryotes, tblastn searches were performed using the organism restriction "eukaryotes NOT bilateria". This recovered a number of genomic and EST matches from lower animals, fungi, plants and protists (Figure 2, 3, 4, Additonal files 9, 14). One EST was recovered from the sponge *Oscarella carmela*, which was previously described [27]. Analysis of this sequence shows that, while it does have a Hedge domain, the downstream sequence does not contain the start of a Hog domain in any frame (Additional file 10). No sequence similarity to a VWA domain is detected in that fragment either. Nevertheless, it indicates that as in the case of Nv 200640, this gene may not contain a Hog domain.

A match was detected to the gene GmGIN1 from the fungus *Glomus mosseae*, which belongs to the Glomeromycota, a sister group of ascomycetes and basidiomycetes and had already been described as having similarity to Hh [29]. The Hog domain has a blast probability of 7e-18 to the best matching Hh Hog domain, which is much better than the blast probability of choanoflagellate Hoglet to the best matching Hh Hog domains (4e-10). Furthermore, good matches to motifs J and K, as well as a region with similarity to motif L. Therefore, GmGIN1 contains a bona-fide Hog domain (Figure 2, 3, 4, Additional file 14). The upstream domain of GmGIN1 shares similarity with Ras-like GTPases, e.g. the Arabidopsis protein AIG1 (avrRpt2-induced gene 1) and the animal The IAN/IMAP subfamily [29]. However, this ORF lacks a signal peptide and may therefore not be secreted.

**Figure 12:**
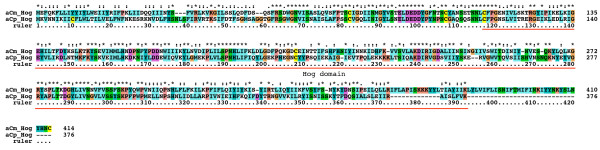
Pairwise sequence alignment of Alveolata aCm and aCp Hog.

A number of matches were found in Alveolata, i.e. in the dinoflagellates *Alexandrium tamarense*, *Amphidinium carterae*, and *Karlodinium micrum *(blast expected probability of aKm Hog: 9e-17 to the best matching Hh Hog domain; note: blast probabilities below also refer to Hh Hog domains) and the apicomplexans *Cryptosporidium muris *and *Cryptosporidium parvum *(blast prob. of aCp Hog: 4e-08). Their Hog domains contain motifs J and K, although in a few cases the cysteine has been replaced with a serine in motif J (Figure 2, 3, 4, Additional file 14). The aCm and aCp sequences are most likely full length, they have signal peptide sequences for secretion and share a conserved upstream region of about 100 residues in length that contains two conserved cysteine residues (Figure 12), but no sequence similarity of this motif to other known domains was found.

**Figure 13:**
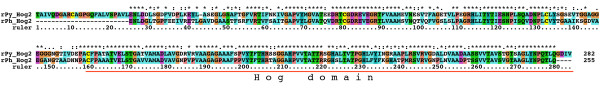
Pairwise sequence alignment of red algae rPy and rPh Hog2.

**Figure 14:**
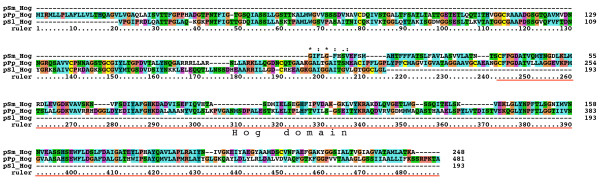
Multiple sequence alignment of moss pPp Hog, pSl Hog and pSm Hog.

Further Hog sequences were found in red algae and mosses (Figure 2, 3, 4, Additional file 14): In the mosses *Selaginella moellendorffii *(blast prob.: 1e-09) and *Physcomitrella patens *one sequence each with a Hog domain; in the red algae *Chondrus crispus *two sequences (blast prob. of rCc Hog: 1e-10); in *Griffithsia japonica *one sequence (blast prob.: 3e-08); in *Porphyra haitanensis *(blast prob. of rPh Hog: 4e-07) and *Porphyra yezoensis *two Hog domain ORFs each; and in *Gracilaria changii *six ORF fragments (blast prob. of rGc Hog1: 3e-12). Those moss and red algae Hog domains that are not truncated have motifs J and K, although the cysteine residue in motif J has been changed to serine, threonine, or aspartate. Alignment of rPy and rPh Hog2 revealed conserved sequences upstream of the Hog domain, however, these two sequences are relatively closely related so this conservation is not surprising (Figure 13). Blast searches with this upstream region did not reveal matches in any other organisms. Similarity, alignment of the moss sequences revealed also a conserved upstream region that was not found in other organisms (Figure 14). The *P. patens *sequence is presumably full length, since it was predicated from genomic sequence, and it has a good signal peptide. One EST sequence supposedly stems from rice (XX 104K18), however, it has a much better match to Hh Hog domains (blast prob.: 3e-24) than other non-metazoan Hog domains, and we could not find any match to rice genomic sequences. Therefore, this sequence may come from a contaminating organism and is designated as species XX here.

**Figure 15:**
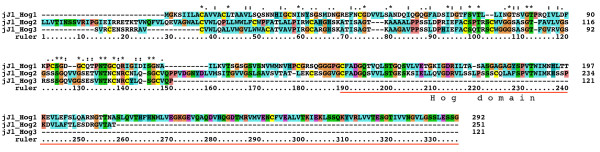
Multiple sequence alignment of jakobid jJl Hog1, Hog2, and Hog3.

**Figure 16:**
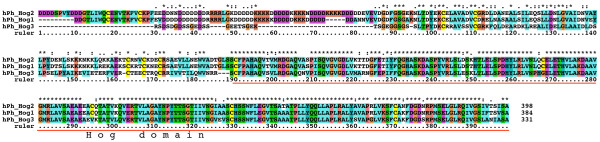
Multiple sequence alignment of haptophyte hPh Hog1, Hog2, and Hog3.

Additional Hog-like sequences were recovered from the cercozoan *Bigelowiella natans *(blast prob.: 9e-10), from the cryptophyte *Guillardia theta *(blast prob. of crGt Hog1: 6e-11 to Hog of Mo hoglet), and from the jakobid *Jakoba libera *(blast prob. jJl Hog1: 2e-05) (Additional file 14). These sequences have motif J, although the cysteine has been replaced, and in those cases, where the C-terminal region is complete, it is clear that motif K is not conserved. Sequence alignment of the *J. libera *Hog sequences revealed conserved upstream sequences with some conserved cysteine residues (Figure 15). Numerous ESTs cover jJl Hog1 and therefore its ORF could be complete. If this is the case, the putative start methionine has a good signal sequence for secretion, and therefore jJl Hog1 has the same global structural features as the animal Hh and Hh-related proteins, i.e. a secreted N-terminal domain followed by a Hog domain. Finally, three sequences were recovered from the haptophyte *Pleurochrysis haptonemofera*. Sequence alignment revealed sequence conservation upstream of the Hog domain with conserved cysteine residues. However, it is noteworthy that the Hog domain is much better conserved than the upstream region, indicating that the upstream region can evolve more rapidly (Figure 16).

Overall, these results show that Hog domains occur in many different branches of the major groups of eukaryotes. However, multiple losses seem to have occurred, since in many branches we did not detect Hog domains, for example, in *Arabidopsis thaliana *and other higher plants, or in the currently sequenced ascomycetes and basidiomycetes, or in other sequenced organisms such as Dictyostelium.

### Other genes of the Hh pathway in Enoplea and *N. vectensis*

*C. elegans *not only lacks a bona-fide Hh molecule, but several other components of the Hh signaling pathway have been lost as well. In particular orthologs of the Hh signaling pathway in recipient cells, i.e. *smoothened*, *fused*, *suppressor of fused *(*sufu*), and *costa *are missing [18]. On the other hand, a homolog of the transcription factor Cubitus interruptus (Ci/Gli) is present, albeit it has been adapted for sex determination. And multiple homologs for the receptor of Hh, i.e. Patched, have been found in *C. elegans *[18], as well as the related molecule Dispatched, required for secretion of Hh. Patched, Dispatched, Smoothened, Ci/Gli and Hip have already been found in *N. vectensis *[25,26]. We were particularly interested to find components lacking in *C. elegans *in the relatively well sequence genomes of *N. vectensis *and *T. spiralis*. Using reciprocal blast searches, we have attempted to identify these components of the pathway in Nematostella and Enoplea (Table 2). In Xiphinema we only detected an EST for *patched*, but this is not surprising giving the limitations of the current dataset. In *T. spiralis *we detected *patched*, *dispatched*, *dally-like *and Ci/Gli, but found no evidence for *Ihog*, *smoothed*, *costa*, *fused*, and *sufu*. This is actually identical to the situation in *C. elegans*. Presently about 56.8 Mb of an estimated genome size of about 65 Mb has been sequenced for *T. spiralis *[41]. If we assume that only about 80% has been sequenced, the probability of finding only the genes listed in Table 2, but missing the others is 0.013%. If the sequence coverage is higher, this probability would even be lower. Therefore, we have to assume that in *T. spiralis*, even though it has a bona-fide *hh *gene, the Hh signaling pathway is compromised in a similar way as in *C. elegans*.

**Table 2:** Components of the Hh signaling pathway in *N. vectensis *and *Xiphinema *sp. The absence of a gene does not mean it is not present, it just may not have been sequenced yet. Numbers indicate the protein prediction in JGI (Nv) or the accession number (XC). For more information on pathway components and *C. elegans *genes see [18]. Best blast scores are given for the Nv predictions in parenthesis.

Gene	Nv	XC	Ts	Ce
dispatched	2), 88278 (e-100)	-	yes (2 copies)	*ceh-14*, *ptd-2*
Ihog	-***	-	-	no
dally-like	247677 (4e-71)	-	yes	*gpn-1*
Patched	1), 84424 (0.0)	CV511563	yes	*ptc-1*, *ptc-3*
smoothened	2), 208236 (e-123), 92220 (4e-84)	-	-*	no
Costa	79512 (e-135) #	-	-	no
Fused	136852 (4–63)	-	-**	no
Sufu	246114 (2e-89)	-	-	no
cubitus interruptus (Ci/Gli)	2), 116463 (3e-85)	-	yes	*tra-1*

# This match is to human KIF27, costa itself is rather divergent and may not be a bona-fide ortholog of KIF27, and there is functional divergence between mammals and Drosophila in this aspect of the pathway [42].

* best reciprocal match found is to Drosophila frizzled dFz2

** best reciprocal match found is to ULK3 kinase

*** best reciprocal match of Nv185528 is to fish protogenin 5e-75

1) mentioned in [25]

2) mentioned in [26]

In *N. vectensis *we found good orthologues for *dispatched*, *dally-like*, *patched*, *smoothened*, *fused*, *sufu *and *Ci *(Table 2). No obvious homolog was found for Ihog. In the case of Drosophila *costa*, good matches to its human homologs were found, and Drosophila *costa *is rather divergent. Recently it has been shown that the mammalian homologues of *fused *and *costa *do not play the same key role in the pathway as in flies, instead *sufu *plays a major role [42,43]. Overall, it looks like most of the key players of the Hh pathway are present in *N. vectensis *so that it is clear that the pathway was already well established before the split of Cnidaria and Bilateria.

### Genes with novel Hint-like (Vint) domains

**Figure 17:**
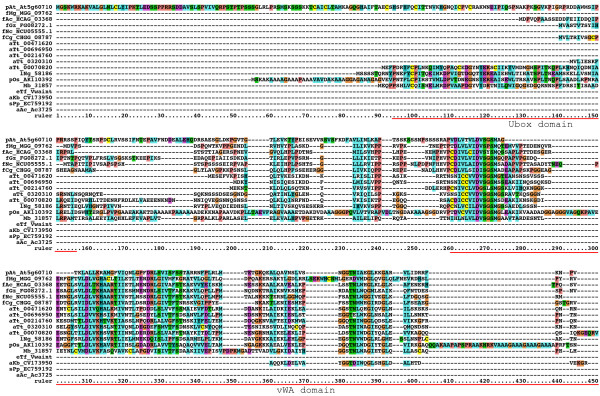
**Multiple sequence alignment of VWA domain – Hint-like domain proteins, part 1**. Proteins containing a VWA merged to a Hint-like domain were discovered in Tetrahymena, several fungal species, as well as several other eukaryote branches, including choanoflagellates. The VWA domain and the Hint-like domain (Vint) with motifs A and B of the Hint domain are marked in the alignment. A new domain between the VWA and Vint domain is marked with Vwaint. Four proteins also have an Ubox upstream of the VWA domain. An alignment of selected Vint domains to Hh Hog domains is presented in additional file 15. *A. thaliana *At5g60710 is not a Vint protein, but one of the best matching VWA domain containing proteins. While it lacks the Vint domain, it does have some weak similarity to the Vwaint domain, and upstream of the VWA domain is a Ring finger, which shares similarity with the Ubox motif. It would be worthwhile to investigate this similarity with a detailed evolutionary analysis in the future.

**Figure 18:**
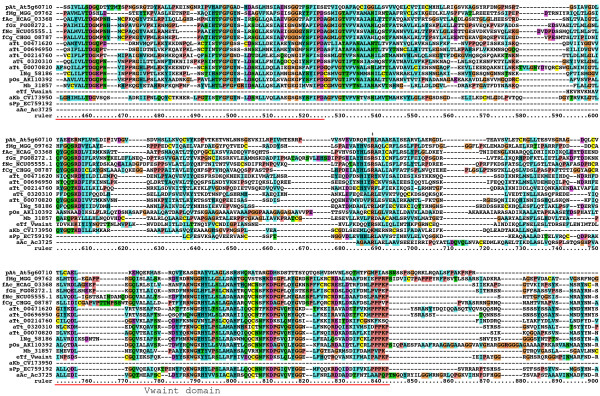
**Multiple sequence alignment of VWA domain – Hint-like domain proteins, part 2**. Continuation of the multiple sequence alignment of Figure 17.

**Figure 19:**
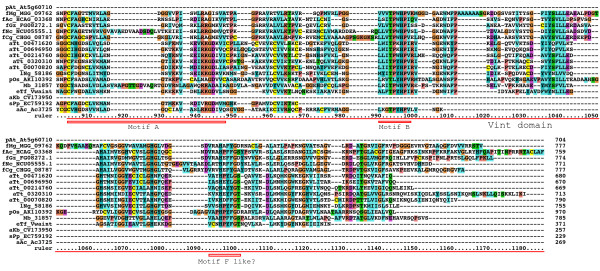
**Multiple sequence alignment of VWA domain – Hint-like domain proteins, part 3**. Continuation of the multiple sequence alignment of Figure 18.

During the tblastn searches ESTs and ORFs from non-Hog genes such as inteins were discovered, usually in the non-significant zone at the bottom of the results lists. One group of genes attracted our attention, because upon closer inspection it became apparent that these genes had an amino-terminal domain comprised of a VWA domain followed by a region that has good similarity to the first part of the Hint domain, i.e. in particular motifs A and B (Figure 17, 18, 19, Additional file 15). This observation was intriguing given that in Nematostella Nv 200640 a Hedge domain is followed by a VWA domain. Further blast searches revealed the presence of these VWA-Hint proteins in Tetrahymena, several fungal species, the Heterolobosea *Naegleria gruberi*, the parabasilid *Tritrichomonas foetus*, dinoflagellates, the slime mold *Physarum polycephalum*, rice and the chanoflagellate *Monosiga brevicollis*. Additional matches in other species, for example, pine tree, were found in the EST database, but not included here, because the fragmentary nature of the sequence information made it impossible to determine, whether the VWA domain resides in the same transcript as the Hint domain (data not shown). No match could be found for the cDNA sequence from rice (pOs AK110392) in the genomic sequence, but ESTs recovered from other plants support the notion that VWA-Hint proteins exist in plants.

The VWA-Hint proteins do not seem to have a signal peptide for secretion. The VWA domain is located at the N-terminus of the proteins, although in four cases a Ubox precedes the VWA domain (Figure 17, 18, 19). A region of around 300 residues separates the VWA domain from the Hint domain. This region has several small patches of conservation and one large region, that we propose to call Vwaint domain. At the C-terminus a Hint-like domain follows, which is of similar size as a Hog domain. However, the best conserved features are only motifs A and B, i.e. the N-terminal region of the Hint-like domain. One region shares a little similarity with motif F of inteins and BIL-Bs, but motifs J, K and L are lacking (Figure 17, 18, 19). The Hint-like domain is also rather different from inteins or Hog domains, the best blast matches of aTt 00471620 are to honeybee Hh with a probability 0.013. Therefore, these sequences cannot be classified as intein, Hog or Bil domains, and we refer to these genes as Vint genes. Vint genes are apparently so wide spread in eukaryotes that we have to assume that a common ancestor was present in early eukaryotes. However, Vint genes seem to be lacking in Arabidopsis, many fungi (for example, *Saccharomyces cerevisiae*), and in Metazoa. Multiple independent losses in different lineages seem the most likely explanation for this absence.

Our searches also revealed a group of proteins from bacteria that had a Hint-like domain at their C-terminus and shared some weak sequence similarity in their N-terminal region (Additional files 16, 17). At least some of these proteins are predicated to have signal peptides for secretion, and the upstream region has two cysteine residues conserved between all sequences. The Hint-like domains of these bacterial proteins are also quite divergent from inteins, Hog and BIL domains, and represent yet another subgroup. This subgroup has previously also been detected by Dassa and Pietrokovski [21]. The new members we retrieved here support the notion that this is yet another new type of Hint protein.

## Discussion

### Hh and hh-related proteins in nematodes

**Figure 20:**
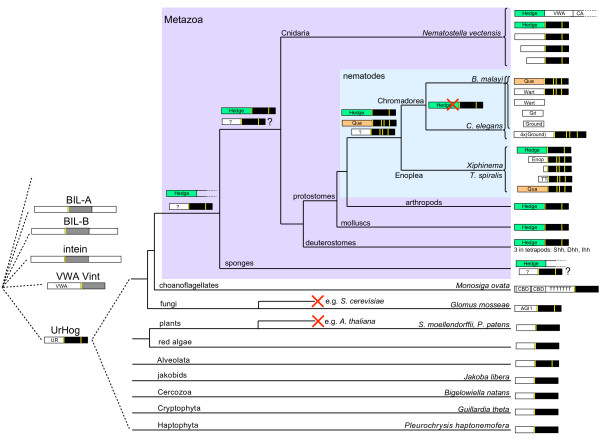
**Summary of the evolution of *hh *and *hh*-related genes**. For detailed discussion of the evolution of the Hog proteins see text. The right side shows the different types of ORFs found in different organisms. The sizes are not to scale. The "Hedge" domain is marked in green, the Qua domain in orange, and the Hog domain in black, with yellow bars representing the conserved cysteine residues. T stands for poly-threonine repeats. Red 'X's mark branches where a gene loss occurred.

Hh genes are present in deuterostomes as well as in several different protostome phyla such as molluscs, annelids, and arthropods (Figure 20). However, in nematodes the situation is more complex. In *C. elegans*, *C. briggsae *and *B. malayi *we find no *hh *gene but instead many *hh*-related genes. We recovered 19 *hh*-related genes from the nematode *B. malayi*. Based on empirical evidence from other gene families we estimate that the genome of *B. malayi *is around 80% complete (K. Mukherjee and T. B., unpublished). Therefore, some additional *hh*-related genes might still be forthcoming. But the present survey shows that members of the *qua*, *wrt*, *grd *and *grl *gene families are all present in *B. malayi*. Only a representative of a *grd *gene with a Ground domain has so far not been found. The phylogenetic analyses show that while there are some instances of direct orthology between *B. malayi *and Caenorhabditis genes, in many instances, in particular for the *grl *genes, the relationship is not clear and in fact suggests that independent diversification occurred in these two Chromadorea branches. This shows that these gene families have been actively evolving in nematodes.

In the more distantly related Enoplea nematodes *Xiphinema sp*. and *T. spiralis *a strikingly different pictures emerges. In both species we find both a *hh *gene as well as several *hh*-related genes. In *T. spiralis *we also find a *quahog *gene, and – based on the phylogenetic analyses – some of the Xiphinema genes could also be *quahog *genes. Two *T. spiralis *and one Xiphinema protein share a new motif (Enop motif) upstream of the Hog domain that appears to be specific to Enoplea nematodes. However, there are also a number of instances of N-terminal sequences that are very short. Several of these proteins cluster with the "Enop" proteins in the phylogenetic analyses, suggesting that they diverged from a common ancestor. However, two proteins with short N-terminal regions (Ts Xhog3 and XC Shog2) are rather divergent and do not reliably fall within the clade of nematode-specific Hog proteins ("Nema-Hog" proteins) in phylogenetic analyses. In particular Ts Xhog3 lacks the conserved cysteine pair usually found in Nema-Hog domains, and it shares motifs K and L with Hh Hog domains, indicating it could be derived from a Hh protein. Therefore, while these genes could have diverged from *hh *or Nema-Hog genes, it may also be possible that the represent ancestral genes that were lost in Chromadorea. In conclusion, we think that there were probably at least three different types of Hog genes in the common ancestor of Enoplea and Chromadorea, one *hh *gene, one *quahog *gene and one gene which give rise to the *wrt/grd *branch in Chromadorea and the Ts Xhog1/2 branch in Enoplea. But possibly up to five Hog genes could have existed in the common ancestor. The proliferation into further distinct groups such as *wrt*, *ground *and *ground-like *appears to have happened later in a branch specific manner.

Many different N-termini now exist in Nema-Hog proteins. Two possible mechanisms can explain this diversity: Either acquisition of new N-terminal domains, or divergent evolution of existing N-terminal domains. A relatively good case can be made that all Wart, Ground and Ground-like domains arose from a single common ancestor based on weak sequence similarities between the motifs [17]. This relationship is also supported by the phylogenetic analyses of the Hog domains. Therefore, multiple loss of the Hog domain must have occurred secondarily within the *wrt *and *ground *families. The presence of the rather short N-termini in Enoplea suggests that these regions have evolved and diverged through mutations, rather than by acquisition of a new domain. The threonine-rich stretch in XC Thog is very likely the result of polymerase slippage, though it is striking that this feature has evolved separately also in the choanoflagellate Hoglet protein [22]. It is also worth mentioning that some of the Caenorhabditis N-terminal domains have repetitive regions outside of the conserved Ground and Ground-like domains, mainly proline, glycine and serine. For example, Ce *grl-23 *has a 176 residue long stretch upstream of the Ground-like domain containing 125 glycine residues. In conclusion, most of the observed variability in the N-terminal domains of nematode Hh-related proteins is probably the result of sequence divergence from a progenitor, rather than acquisition of new domains. Loss of N-terminal domains in the case of *C. elegans *Hog-1, as well as loss of Hog domains did occur however.

A surprising observation is the fact that *T. spiralis *has a *hh *gene, but apparently lacks several components of the Hh pathway, such as Smoothened. Particularly noteworthy is that the components that appear to be missing are the same as in *C. elegans*. This would suggest that the signaling pathway was modified by loss already before the split of Enoplea and Chromadorea, even though *hh *was maintained in Enoplea. While one could imagine that Hh could be maintained in an animal parasite such as *T. spiralis *to affect host cells, this is very unlikely in the case of the plant nematode Xiphinema. It implies that Hh has an important function even in the absence of Smoothened, and it refutes the hypothesis that the Nema-Hog genes evolved directly from *hh *concomitantly with the other changes in the Hh pathway.

### Hog proteins in Cnidaria

In Cnidaria we also encounter a complex situation with both Hh and Hh-related proteins. Both in *N. vectensis *and *A. millepora *we find bona-fide *hh *genes that have a Hedge and a Hog domain. Another gene is well conserved between *N. vectensis *and *A. millepora *and has a distinct, novel secreted N-terminal domain. Two further Hh-related proteins in *N. vectensis *have yet other, distinct N-termini. The upstream region of the two closely related genes retrieved from Hydra do not share any similarity with those in Nematostella, indicating divergent evolution. No sequence similarity of these new N-terminal motifs has been found outside Cnidaria.

The *hh*-related genes from Cnidaria are however distinct from those in nematodes, since the phylogenetic analyses of the Hog domains does not show them to be closely related. Therefore, we would like to suggest that – as in the case of the nematode *hh*-related genes – the Cnidarian N-terminal domains have evolved from common ancestors by divergent evolution rather than by domain acquisition.

The case of Nv 200640 is perhaps a special exception. In this protein we find an N-terminal Hedge domain fused to a large extracellular protein that contains a VWA domain as well as CA and EGF repeats, but it clearly lacks a Hog domain. The VWA domain is a 200 residue long domain first identified in von Willebrand Factor [44,45]. VWA domains are found both in extracellular and intracellular proteins, such as non-fibrillar collagens, plasma proteins such as complement factors and integrins, and they mediate adhesion via metal ion-dependent adhesion sites. Likewise, the CA repeats also mediate adhesion in a Ca2+ dependent fashion. Therefore, the Nv 200640 protein is probably involved in cell adhesion. This shows that the Hedge domain can also evolve in a modular fashion and separate from the Hog domain. The EST recovered from sponges also has a Hedge domain that lacks the immediately following Hog domain, and may perhaps represent also a protein lacking a Hog domain.

### Hog proteins in lower eukaryotes

We have recovered a substantial number of Hog domain proteins from many diverse groups of eukaryotes, mostly protists, such as red algae, moss, alveolates (ciliates, dinoflagellates, apicomplexans), cryptophytes, jakobids, haptophytes, cercozoa and Glomeromycota fungi. While some of these Hog sequences are quite divergent, they are invariably most closely related to Hog domain proteins from animals, and not to inteins, such as those found in fungi, or to BIL or Vint domains. Given the widespread occurrence in many of the major groups of eukaryotes ([46], we must conclude that Hog proteins were present already in the earliest eukaryotes. We find diverse N-termini associated with the Hog domain that are only conserved to limited extends within groups (case in point are the various conserved N-termini in nematodes). Many of these limited conserved N-termini have conserved cysteine residues, and in cases, where one can be quite confident of the start methionine, they start with a good signal peptide for secretion. Only in the case of the fungal protein GmGIN1 and the choanoflagellate Hoglet are distinct other N-terminal domains fused to the Hog domain. Therefore, we postulate that an ancestral Ur-Hog gene existed, with a secreted N-terminal domain and an autoprocessing Hog domain, that may have added a sterol or similar moiety to its secreted N-terminus. This gene evolved in concert with eukaryote evolution and was lost in several branches. In animals, the question arises about the origin of the Hedge domain. Both in sponge and in Nematostella we find a Hedge gene that lacks a Hog domain. Perhaps such a gene merged with a Hog domain in early metazoans. However, the reverse process is also possible: the Hedge domain evolved as an N-terminal variant of a Hog protein in early metazoans, and in the two Hedge genes in sponge and Nematostella the Hog domain was lost later. Both in Cnidaria and nematodes we find both *hh *and *hh*-related genes. Did the *hh*-related genes evolve twice independently from a *hh *precursor in each lineage? This is certainly the most parsimonious hypothesis. Nonetheless, in an alternative scenario, a *hh *and a *hh*-related gene could have been present in the common ancestor of eumetazoa, and the *hh*-related gene would have given rise to the cnidarian and nematode *hh*-related genes. For this hypothesis to be true, we would have to postulate three separate losses of *hh*-related genes: in deuterostomes, in lophotrochozoa, and in arthropods. While this seems rather unlikely, we do observe many losses of Hog genes in various branches of eukaryotes, as well as loss of the Hog domain only in a number of nematode genes so that such a series of losses may not be totally impossible.

### Novel Hint genes

Our searches revealed new genes with Hint motifs merged to VWA domains. Given that a Hedge domain was found fused to a VWA domain in Nematostella we investigated this further and recovered a novel gene family. The well-conserved gene structure consists of a VWA followed by a new domain, termed Vwaint, followed by the "Vint"-type Hint domain. Unlike the Hog proteins, these proteins are most likely not secreted and instead are processed inside the cell. The Vint genes are present in many eukaryotic groups, but must have been lost multiple times, in particular in multicellular eukaryotes. Multiple loss seems to be a common theme also in Hog proteins and especially inteins [21,32]. Inteins may be subject to special selective pressure for loss [21,32], and this pressure may also extend to Hog and Vint proteins. However, gene loss is not uncommon. The *N. vectensis *genome contains a remarkable complexity of highly conserved gene families [25], and several instances of later gene loss in the protostome or deuterostome lineage, for example in the homeobox gene family, have been found [47,48], indicating gene loss later in evolution is feasible.

## Conclusion

We find that the evolution of Hh is more complex than anticipated, and that this gene family is not simply derived from an intein in early metazoan evolution. Both in Cnidaria and nematodes parallel evolution between *hh *and *hh*-related genes occurred. Given that the nematode-specific Hog domain (Nema-Hog) with its distinct features was already present in the progenitor of two very different nematode branches it may be possible that both Hh and some other Hog domain protein was already present in protostomes before the emergence of nematodes and was lost in other lineages such as arthropods. The finding of multiple Hog domain proteins in Cnidaria raises the possibility that multiple distinct types of Hog domain proteins also existed in ancestral Eumetazoa. Snell et al. (2006) suggested that a precursor of a Hedge domain fused to a Hog domain in early Metazoan evolution. However, our discovery that an Ur-Hog gene probably existed in the progenitor of eukaryotes makes if feasible that Hh evolved from an ancestral Hog gene without domain shuffling. In eukaryotes, we now know that at least three different types of Hint domains evolved in parallel: Hog, Vint, and inteins. At present we do not know the origin of the Hog and Vint domains, but perhaps new Hint domains from bacteria, such as described here and by Dassa and Pietrokovski [21] will shed light on that issue in the future.

## Methods

Procedures for retrieving and analyzing sequences have been detailed in Hao et al. 2006 and Mukherjee and Bürglin 2007 [38,48]. Briefly, *B. malayi *sequences were searched at TIGR [49]. Preliminary sequence data for *B. malayi *is deposited regularly into the GSS division of GenBank. This sequencing effort is part of the International Brugia Genome Sequencing Project and is supported by an award from the National Institute of Allergy and Infectious Diseases, National Institutes of Health. ESTs, in particular nematode ESTs, were searched at NCBI [50]. The nematode ESTs are generated by the Washington University Parasitic Nematode EST sequencing project [51]. Many of the protist ESTs were generated by the Protist EST program [52]. The *T. spiralis *genome was searched using the GSC blast server at The Genome Sequencing Center of the Washington University School of Medicine [53]. *N. vectensis *sequences were searched at Stellabase [54,28], and at the DOE Joint Genome Institute (JGI) [55]. Additional genome sequences such as for *Naegleria gruberi*, *Physcomitrella patens *and *Monosiga brevicollis *were searched at the JGI [55]. Zebrafish sequences were retrieved from ZFIN [56,57]. The intein database was checked at New England Biolabs InBase [30,58]. Manual sequence corrections were performed with the help of FGENESH and FGENESH+ at Softberry [59] and PPCMatrix [60]. ESTs representing the same locus were assembled using the CAP3 server at Iowa State University [61].

Sequences were added to an existing database of Hh and Hh-related proteins [38], and are shown in Additional file 9. Protist sequences were arbitrarily named Hog, Hog2, etc. (Additional file 9). For identification and tagging of sequences in the figures the species names were reduced to two and three letter codes and prefixed to sequence names (Table 3). Multiple sequence alignment and phylogenetic analyses using Neighbor joining were carried using Clustal_X [62] and MUSCLE [63,64]. Manual correction of alignments was carried out using SeaView [65]. For Maximum likelihood analysis PHYML was employed [66]. Signal peptide predication was carried out at the SignalP 3.0 server [67,68]. Protein sequence logos were generated using LogoBar [69,70]. Some protein motifs were also identified using the SMART server [71].

**Table 3:** Species abbreviations. Fungi are prefixed with 'f', red algae with 'r', plants with 'p", Alveolata (ciliates, dinoflagellates, Apicomplexa) with 'a', jakobids with 'j', Cercozoa with 'c', Cryptophyta with 'cr', excavates with 'e', haptophytes with 'h', heterolobosea with 'l', and slime molds with 's'.

Codes	Species names
Acm	*Acropora millepora *(Cnidaria)
Ag	*Anopheles gambiae *(malaria mosquito)
At	*Achaearanea tepidariorum *(common house spider)
Bf	*Branchiostoma floridae *(Florida lancelet, Amphioxus)
Bm	*Brugia malayi *(nematode, Chromadorea)
Cap	*Capitella *sp. I ECS-2004 (polychaete)
Cb	*Caenorhabditis briggsae *(nematode, Chromadorea)
Ce	*Caenorhabditis elegans *(nematode, Chromadorea)
Cr	*Caenorhabditis remanei *(nematode, Chromadorea)
Dm	*Drosophila melanogaster *(fruitfly)
Dh	*Drosophila hydei*
Dr	*Danio rerio *(zebrafish)
Gb	*Gryllus bimaculatus *(two-spotted cricket)
Lv	*Lytechinus variegatus *(green sea urchin)
Hm	*Hydra magnipapillata *(Cnidaria)
Mb	*Monosiga brevicollis *(choanoflagellate)
Mi	*Meloidogyne incognita *(southern root-knot nematode, Chromadorea)
Mm	*Mus musculus *(mouse)
Mo	*Monosiga ovata *(choanoflagellate)
Nv	*Nematostella vectensis *(Cnidaria, starlet sea anemone)
Ob	*Octopus bimaculoides *(mollusc)
Oc	*Oscarella carmela *(sponge)
Pt	*Parastrongyloides trichosuri *(nematode, Chromadorea)
Pv	*Patella vulgata *(common limpet, mollusc)
Sp	*Strongylocentrotus purpuratus *(sea urchin)
Tr	*Takifugu rubripes *(fugu)
Ts	*Trichinella spiralis *(nematode, Enoplea)
XC	*Xiphinema *index CSEQDL01 (nematode, Enoplea)
	
aAc	*Amphidinium carterae *(dinoflagellate, Alveolata)
aAt	*Alexandrium tamarense *(dinoflagellate, Alveolata)
aCm	*Cryptosporidium muris *(Apicomplexa, Alveolata)
aCp	*Cryptosporidium parvum *(Apicomplexa, Alveolata)
aKb	*Karenia brevis *(dinoflagellate, Alveolata)
aKm	*Karlodinium micrum *(dinoflagellate, Alveolata)
aTt	*Tetrahymena thermophila *(ciliate, Alveolata)
cBn	*Bigelowiella natans *(Cercozoa)
crGt	*Guillardia theta *(Cryptophyta)
eTf	*Tritrichomonas foetus *(Parabasalidea, excavates)
fAc	*Ajellomyces capsulatus *(ascomycetes, fungus)
fCg	*Chaetomium globosum *(ascomycetes, fungus)
fCt	*Candida tropicalis *(ascomycetes, fungus)
fGm	*Glomus mosseae *(Glomeromycota, fungus)
fGz	*Gibberella zeae *(ascomycetes, fungus)
fMg	*Magnaporthe grisea *(ascomycetes, rice blast fungus)
fNc	*Neurospora crassa *(ascomycetes, fungus)
hPh	*Pleurochrysis haptonemofera *(haptophytes)
jJl	*Jakoba libera *(jakobids)
lNg	*Naegleria gruberi *(heterolobosea)
pAt	*Arabidopsis thaliana *(plants)
pOs	*Oryza sativa *(rice, plants)
pPp	*Physcomitrella patens *(moss, plants)
pSl	*Selaginella lepidophylla *(club moss, plants)
pSm	*Selaginella moellendorffii *(club moss, plants)
RCc	*Chondrus crispus *(carragheen, red algae)
RGc	*Gracilaria changii *(red algae)
RGj	*Griffithsia japonica *(red algae)
RPh	*Porphyra haitanensis *(red algae)
RPy	*Porphyra yezoensis *(red algae)
SPp	*Physarum polycephalum *(slime mold, amoebozoa)

## Authors' contributions

All research was carried out by TRB and the manuscript was written by TRB.

## Supplementary Material

Additional file 1 Multiple sequence alignment of Hog domains used for the protein sequence logos. Multiple sequence alignment in this and subsequent figures was carried out using first MUSCLE and imported subsequently into Clustal_X. Color coding was modified from default Clustal_X color coding by marking all cysteine residues in yellow, small hydrophobic residues in light blue and large hydrophobic residues in cyan blue. The conserved motifs, as well as the C-terminal SRR or ARR region are indicated in the alignment. The two conserved cysteine residues found in the Hog domain of nematode Hh-related proteins are indicated with red arrows. Click here for file

Additional file 2 Full image of the protein sequence logo of aligned Hog domains shown in Figure 1. The color scheme is similar to the one used in the multiple sequence alignments (N,Q,S,T: green; C: yellow; P: pink; G: orange; K,R: red; A,I,L,M,V: blue; F,W,Y: cyan blue; H, purple, D,E: magenta; gaps: white). The extend of the Hint domain and the SRR region are indicated above the logo with a red line. Red boxes underneath the logo indicate the different motifs A, B, F, J, K, L. Click here for file

Additional file 3 Protein sequence logo of nematode Hog domains. Protein sequence logo generated from nematode Hog domains shown in the multiple sequence aligment of Additional file 1. This logo is in register with the Hh Hog domain logo of Additional file 2. The two conserved cysteine residues specific to nematode Hh-related proteins are indicated with red arrows. Click here for file

Additional file 4 Multiple sequence alignment of Wart domains. Wart domains were aligned and visualized in Clustal_X as described in Figure 2. Click here for file

Additional file 5 Phylogenetic tree analysis of Hog domains using Neighbor joining. Neighbor joining tree without protist sequences. The Hog domain of the fungal gene GmGIN1 was used as outgroup. Click here for file

Additional file 6 Phylogenetic tree analysis of Hog domains using Maximum likelihood. Maximum likelihood tree of the same sequences as in Additional file 5 with GmGIN1 as outgroup. Click here for file

Additional file 7 Neighbor joining tree of Hog sequences which were truncated at the N-terminus. Hog sequences were truncated at the N-terminus to have the same size as the Pt wrt sequence fragment. This analysis shows that Pt wrt clusters with the *wrt *genes (arrow). GmGIN1 was used as outgroup. Note: Apart from Figure 5 and 6, and Additional files 5-7 further phylogenetic analysis were carried out that are not shown here. For example, the intein from vacuolar ATPase from *C. tropicalis *was used as outgroup [22] and gave comparable results to the tree analyses shown here. Click here for file

Additional file 8 Multiple sequence alignment of Ground and Ground-like domains. Alignment of nematode Ground and Ground-like domains. Click here for file

Additional file 9 Sequences used in the analysis. List of sequences, accession numbers, notes, predicted signal peptide cleavage sites and protein sequences used in this analysis. Click here for file

Additional file 10 Multiple sequence alignment of "Hedge" domain containing proteins and Hedgehog proteins. Note that Os hhlike and NV 200640 do not line up in the Hog domain region. Click here for file

Additional file 11 Multiple sequence alignment of Quahog proteins. Alignment of nematode Quahog proteins. Click here for file

Additional file 12 Structure of the Nematostella vectensis genomic assembly around Nv 239508. Current assembly of the genomic region around Nv 239508. Color arrows indicate duplicated regions. N gap indicateds two regions with unknown sequence. The green area shows the ESTs found mapping to this region. The CAGN20453 correpsonds to Nv 239508. The yellow area shows regions of sequence similarity, i.e. hydrolase domain, Hog domain, and Reverse transcriptase. CAGN20453 is not sequenced fully, but the 3' read has been mapped to the right side, since the 3' untranslated region matches better to the 2. repeat of the duplication due to some indel differences. However, as will be noted, the final resulting transcript (shown at bottom) would be rather unusual, as it would splice over another gene, i.e. the hydrolase, which is also supported by an EST. Hence, the genomic organization and gene structure in this region could be subject to change, especially given the unsequenced areas. Click here for file

Additional file 13 Predicted protein structure of Nv 200640. Protein motif prediction of the SMART server was used to analyse the ORF Nv 200640, and the different types of conserved motifs found are indicated. Click here for file

Additional file 14 Multiple sequence alignment of non-metazoan Hog proteins. Alignment of non-metazoan Hog proteins including also the ones which are only based on EST fragments. Click here for file

Additional file 15 Multiple sequence alignment of the Hint region of VWA-Vint proteins with Hog domains of Hh proteins. Click here for file

Additional file 16 Multiple sequence alignment of bacterial proteins with a novel type of Hint-like region. Full length multiple sequence alignment of bacterial proteins. Motifs A and B are marked, as well as a small region with some similarity to the beginning of motif F in BIL-As. Note: the conserved upstream regions may be secreted, since some of the sequences have bacterial signal peptides for secretion. Species and the accession number for the sequences are: bSa_STIAU_1829: Stigmatella aurantiaca DW4/3-1 (ZP_01466308); bMx_MXAN_6253: Myxococcus xanthus DK 1622 (YP_634382); bRM_MED297_11140: Reinekea sp. MED297 (ZP_01113290); bPl_plu1731: Photorhabdus luminescens subsp. laumondii TTO1 (NP_929012); bSp_Draf4685: Serratia proteamaculans 568 (ZP_01534811); bYi_YintA_01002283: Yersinia intermedia ATCC 29909 (ZP_00833384). Click here for file

Additional file 17 Multiple sequence alignment of the Hint region of the bacterial proteins with a novel Hint-like domain with Hog domains. Multiple sequence alignment of the bacterial Hint domains from Additional file 16 with Hog domains from animals. Note the roughly similar length. Click here for file

## References

[B1] Cohen MM (2003). The hedgehog signaling network. Am J Med Genet A.

[B2] Hooper JE, Scott MP (2005). Communicating with Hedgehogs. Nature reviews.

[B3] Huangfu D, Anderson KV (2006). Signaling from Smo to Ci/Gli: conservation and divergence of Hedgehog pathways from Drosophila to vertebrates. Development (Cambridge, England).

[B4] Wang Y, McMahon AP, Allen BL (2007). Shifting paradigms in Hedgehog signaling. Current opinion in cell biology.

[B5] Beachy PA, Karhadkar SS, Berman DM (2004). Tissue repair and stem cell renewal in carcinogenesis. Nature.

[B6] Briscoe J, Thérond P (2005). Hedgehog Signaling: From the Drosphila cuticle to anti-cancer drugs. Dev Cell.

[B7] McMahon AP, Ingham PW, Tabin CJ (2003). Developmental roles and clinical significance of hedgehog signaling. Current topics in developmental biology.

[B8] Rubin LL, de Sauvage FJ (2006). Targeting the Hedgehog pathway in cancer. Nature reviews.

[B9] Zardoya R, Abouheif E, Meyer A (1996). Evolution and orthology of hedgehog genes.. Trends Genet.

[B10] Avaron F, Hoffman L, Guay D, Akimenko MA (2006). Characterization of two new zebrafish members of the hedgehog family: atypical expression of a zebrafish indian hedgehog gene in skeletal elements of both endochondral and dermal origins. Dev Dyn.

[B11] Meyer A, Schartl M (1999). Gene and genome duplications in vertebrates: the one-to-four (-to-eight in fish) rule and the evolution of novel gene functions. Current opinion in cell biology.

[B12] Hall TMT, Porter JA, Young KE, Koonin EV, Beachy PA, Leahy DJ (1997). Crystal structure of a Hedgehog autoprocessing domain: Homology between Hedgehog and self-splicing proteins.. Cell.

[B13] Porter JA, Young KE, Beachy PA (1996). Cholesterol modification of Hedgehog signaling proteins in animal development.. Science.

[B14] Bijlsma MF, Spek CA, Peppelenbosch MP (2004). Hedgehog: an unusual signal transducer.. Bioessays.

[B15] Gallet A, Rodriguez R, Ruel L, Therond PP (2003). Cholesterol modification of hedgehog is required for trafficking and movement, revealing an asymmetric cellular response to hedgehog. Dev Cell.

[B16] Gallet A, Ruel L, Staccini-Lavenant L, Therond PP (2006). Cholesterol modification is necessary for controlled planar long-range activity of Hedgehog in Drosophila epithelia. Development (Cambridge, England).

[B17] Aspöck G, Kagoshima H, Niklaus G, Bürglin TR (1999). Caenorhabditis elegans has scores of hedgehog-related genes: sequence and expression analysis.. Genome Res.

[B18] Bürglin TR, Kuwabara PE (2006). Homologs of the Hh signalling network in C. elegans. WormBook.

[B19] Hao L, Mukherjee K, Liegeois S, Baillie D, Labouesse M, Bürglin TR (2006). The hedgehog-related gene qua-1 is required for molting in Caenorhabditis elegans. Dev Dyn.

[B20] Beachy PA, Cooper MK, Young KE, von Kessler DP, Park WJ, Hall TMT, Leahy DJ, Porter JA (1997). Multiple roles of cholesterol in hedgehog protein biogenesis and signaling.. Cold Spring Harb Symp Quant Biol.

[B21] Dassa B, Pietrokovski S, Belfort M, Stoddard BL, Wood DW, Derbyshire V (2005). Origin and evolution of inteins and other Hint domains. Homing Endonucleases and Inteins.

[B22] Snell EA, Brooke NM, Taylor WR, Casane D, Philippe H, Holland PW (2006). An unusual choanoflagellate protein released by Hedgehog autocatalytic processing. Proc Biol Sci.

[B23] Philippe H, Snell EA, Bapteste E, Lopez P, Holland PW, Casane D (2004). Phylogenomics of eukaryotes: impact of missing data on large alignments. Mol Biol Evol.

[B24] James TY, Kauff F, Schoch CL, Matheny PB, Hofstetter V, Cox CJ, Celio G, Gueidan C, Fraker E, Miadlikowska J, Lumbsch HT, Rauhut A, Reeb V, Arnold AE, Amtoft A, Stajich JE, Hosaka K, Sung GH, Johnson D, O'Rourke B, Crockett M, Binder M, Curtis JM, Slot JC, Powell MJ, Taylor JW, McLaughlin DJ, Spatafora JW, Vilgalys R (2006). Reconstructing the early evolution of Fungi using a six-gene phylogeny. Nature.

[B25] Technau U, Rudd S, Maxwell P, Gordon PM, Saina M, Grasso LC, Hayward DC, Sensen CW, Saint R, Holstein TW, Ball EE, Miller DJ (2005). Maintenance of ancestral complexity and non-metazoan genes in two basal cnidarians. Trends Genet.

[B26] Walton KD, Croce JC, Glenn TD, Wu SY, McClay DR (2006). Genomics and expression profiles of the Hedgehog and Notch signaling pathways in sea urchin development. Developmental biology.

[B27] Nichols SA, Dirks W, Pearse JS, King N (2006). Early evolution of animal cell signaling and adhesion genes. Proceedings of the National Academy of Sciences of the United States of America.

[B28] Sullivan JC, Ryan JF, Watson JA, Webb J, Mullikin JC, Rokhsar D, Finnerty JR (2006). StellaBase: the Nematostella vectensis Genomics Database. Nucleic Acids Res.

[B29] Requena N, Mann P, Hampp R, Franken P (2002). Early developmentally regulated genes in the arbuscular mycorrhizal fungus Glomus mosseae: identification of GmGIN1, a novel gene with homology to the C-terminus of metazoan hedgehog proteins. Plant Soil.

[B30] Perler FB (2002). InBase: the Intein Database. Nucleic Acids Res.

[B31] Poulter RT, Goodwin TJ, Butler MI (2007). The nuclear-encoded inteins of fungi. Fungal Genet Biol.

[B32] Pietrokovski S (2001). Intein spread and extinction in evolution. Trends Genet.

[B33] Amitai G, Belenkiy O, Dassa B, Shainskaya A, Pietrokovski S (2003). Distribution and function of new bacterial intein-like protein domains. Molecular microbiology.

[B34] Pietrokovski S (1994). Conserved sequence features of inteins (protein introns) and their use in identifying new inteins and related proteins.. Protein Science.

[B35] Dalgaard JZ, Moser MJ, Hughey R, Mian IS (1997). Statistical modeling, phylogenetic analysis and structure prediction of a protein splicing domain common to Inteins and Hedgehog proteins.. Journal of Computational Biology.

[B36] Perler FB, Olsen GJ, Adam E (1997). Compilation and analysis of intein sequences.. Nucl Acids Res.

[B37] Saleh L, Perler FB (2006). Protein splicing in cis and in trans. Chemical record.

[B38] Hao L, Johnsen R, Lauter G, Baillie D, Bürglin TR (2006). Comprehensive analysis of gene expression patterns of hedgehog-related genes. BMC Genomics.

[B39] De Ley P, Community TCR (2006). A quick tour of nematode diversity and the backbone of nematode phylogeny. WormBook.

[B40] Huang G, Gao B, Maier T, Allen R, Davis EL, Baum TJ, Hussey RS (2003). A profile of putative parasitism genes expressed in the esophageal gland cells of the root-knot nematode Meloidogyne incognita. Mol Plant Microbe Interact.

[B41] Genome Sequencing Center, Washington University School of Medicine. http://genome.wustl.edu/.

[B42] Varjosalo M, Li SP, Taipale J (2006). Divergence of hedgehog signal transduction mechanism between Drosophila and mammals. Dev Cell.

[B43] Svard J, Heby-Henricson K, Persson-Lek M, Rozell B, Lauth M, Bergstrom A, Ericson J, Toftgard R, Teglund S (2006). Genetic elimination of Suppressor of fused reveals an essential repressor function in the mammalian Hedgehog signaling pathway. Dev Cell.

[B44] Colombatti A, Bonaldo P, Doliana R (1993). Type A modules: interacting domains found in several non-fibrillar collagens and in other extracellular matrix proteins. Matrix.

[B45] Perkins SJ, Smith KF, Williams SC, Haris PI, Chapman D, Sim RB (1994). The secondary structure of the von Willebrand factor type A domain in factor B of human complement by Fourier transform infrared spectroscopy. Its occurrence in collagen types VI, VII, XII and XIV, the integrins and other proteins by averaged structure predictions. J Mol Biol.

[B46] Baldauf SL (2003). The deep roots of eukaryotes. Science.

[B47] Ryan JF, Burton PM, Mazza ME, Kwong GK, Mullikin JC, Finnerty JR (2006). The cnidarian-bilaterian ancestor possessed at least 56 homeoboxes. Evidence from the starlet sea anemone, Nematostella vectensis. Genome Biol.

[B48] Mukherjee K, Bürglin TR (2007). Comprehensive Analysis of Animal TALE Homeobox Genes: New Conserved Motifs and Cases of Accelerated Evolution. Journal of molecular evolution.

[B49] J. Craig Venter Institute. http://www.tigr.org.

[B50] BLAST: Basic Local Alignment and Search Tool. http://www.ncbi.nlm.nih.gov/blast/.

[B51] Wylie T, Martin JC, Dante M, Mitreva MD, Clifton SW, Chinwalla A, Waterston RH, Wilson RK, McCarter JP (2004). Nematode.net: a tool for navigating sequences from parasitic and free-living nematodes. Nucleic Acids Res.

[B52] O'Brien EA, Koski LB, Zhang Y, Yang L, Wang E, Gray MW, Burger G, Lang BF (2007). TBestDB: a taxonomically broad database of expressed sequence tags (ESTs). Nucleic Acids Res.

[B53] GSC: BLAST Server. http://genome.wustl.edu/tools/blast/.

[B54] StellaBase: Nematostella vectensis Database. http://evodevo.bu.edu/stellabase/.

[B55] DOE Joint Genome Institute. http://www.jgi.doe.gov/.

[B56] ZFIN: The Zebrafish Model Organism Database. http://zfin.org/.

[B57] Sprague J, Bayraktaroglu L, Clements D, Conlin T, Fashena D, Frazer K, Haendel M, Howe DG, Mani P, Ramachandran S, Schaper K, Segerdell E, Song P, Sprunger B, Taylor S, Van Slyke CE, Westerfield M (2006). The Zebrafish Information Network: the zebrafish model organism database. Nucleic Acids Res.

[B58] NEB Intein Database. http://www.neb.com/neb/inteins.html.

[B59] SoftBerry. http://www.softberry.com.

[B60] Bürglin TR (1998). PPCMatrix: a PowerPC dotmatrix program to compare large genomic sequences against protein sequences.. Bioinformatics.

[B61] Sequence Assembly at Iowa State University. http://deepc2.psi.iastate.edu/aat/cap/cap.html.

[B62] Thompson JD, Gibson TJ, Plewniak F, Jeanmougin F, Higgins DG (1997). The CLUSTAL_X windows interface: flexible strategies for multiple sequence alignment aided by quality analysis tools.. Nucl Acids Res.

[B63] MUSCLE. http://phylogenomics.berkeley.edu/cgi-bin/muscle/input_muscle.py.

[B64] Edgar RC (2004). MUSCLE: a multiple sequence alignment method with reduced time and space complexity. BMC Bioinformatics.

[B65] Galtier N, Gouy M, Gautier C (1996). SEAVIEW and PHYLO_WIN: two graphic tools for sequence alignment and molecular phylogeny.. Comput Appl Biosci.

[B66] Guindon S, Gascuel O (2003). A simple, fast, and accurate algorithm to estimate large phylogenies by maximum likelihood. Syst Biol.

[B67] SignalP 3.0 Server. http://www.cbs.dtu.dk/services/SignalP/.

[B68] Bendtsen JD, Nielsen H, von Heijne G, Brunak S (2004). Improved prediction of signal peptides: SignalP 3.0. J Mol Biol.

[B69] LogoBar - Java application for protein sequence Logos. http://www.biosci.ki.se/groups/tbu/logobar/.

[B70] Pérez-Bercoff, Koch J, Bürglin TR (2006). LogoBar: bar graph visualization of protein logos with gaps. Bioinformatics.

[B71] Letunic I, Copley RR, Schmidt S, Ciccarelli FD, Doerks T, Schultz J, Ponting CP, Bork P (2004). SMART 4.0: towards genomic data integration. Nucleic Acids Res.

