# Supplementary material for: Evolution of hedgehog and hedgehog-related genes, their origin from Hog proteins in ancestral eukaryotes and discovery of a novel Hint motif
Source: BMC Genomics. 2008 Mar 11;9:127. doi: 10.1186/1471-2164-9-127 (PMC2362128; doi:10.1186/1471-2164-9-127)
Supplement: Additional file 1 — Multiple sequence alignment of Hog domains used for the protein sequence logos. Multiple sequence alignment in this and subsequent figures was carried out using first MUSCLE and imported subsequently into Clustal_X. Color coding was modified from default Clustal_X color coding by marking all cysteine residues in yellow, small hydrophobic residues in light blue and large hydrophobic residues in cyan blue. The conserved motifs, as well as the C-terminal SRR or ARR region are indicated in the alignment. The two conserved cysteine residues found in the Hog domain of nematode Hh-related proteins are indicated with red arrows. [file 1471-2164-9-127-S1.pdf]

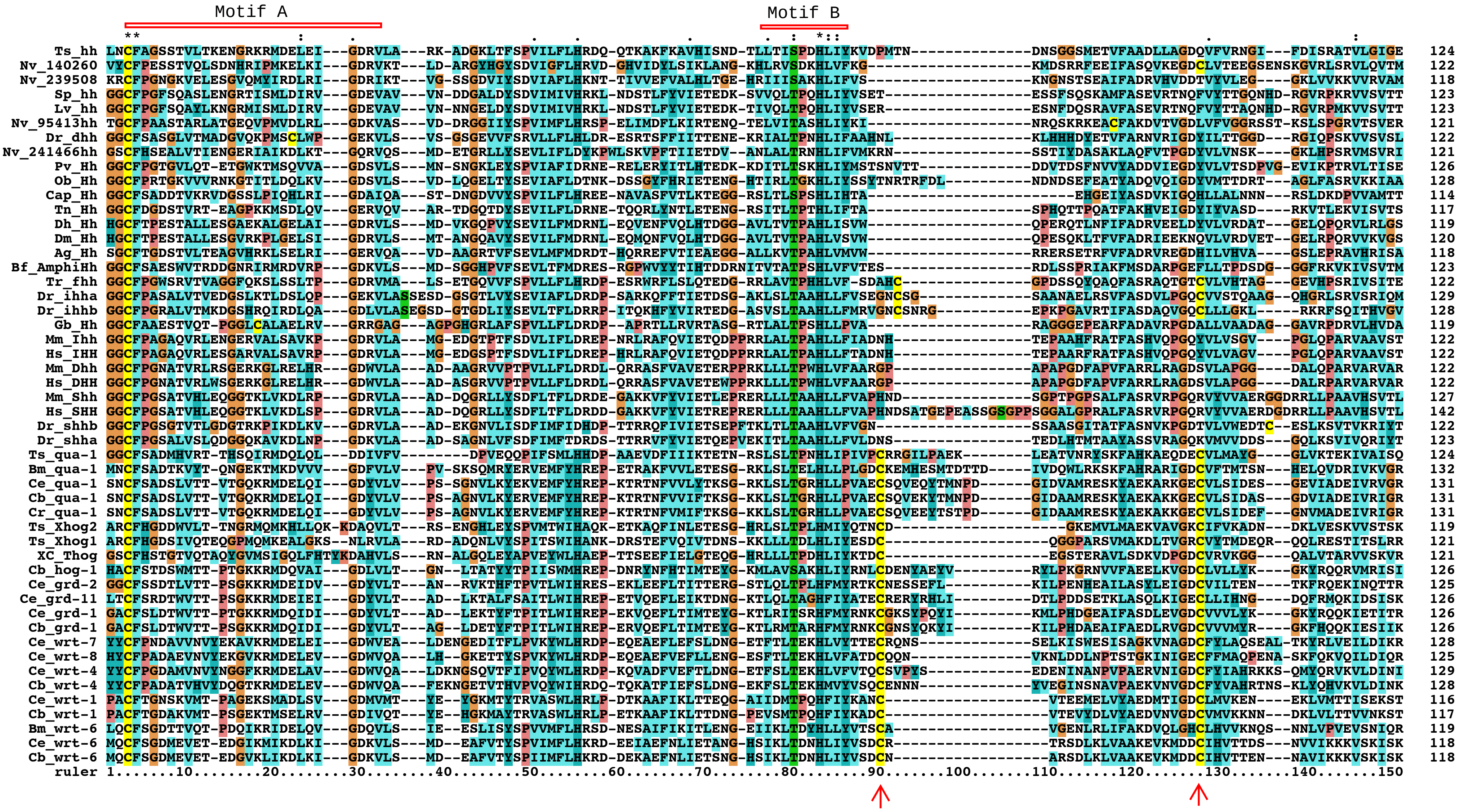
Motif A
Motif B
Ts_hh 124
Nv_140260 122
Nv_239508 118
Sp_hh 123
Lv_hh 123
Nv_95413hh 121
Dr_dhh 122
Nv_241466hh 121
Pv_Hh 126
Ob_Hh 128
Cap_Hh 114
Tn_Hh 117
Dh_Hh 119
Dm_Hh 120
Ag_Hh 118
Bf_AmphiHh 123
Tr_fhh 122
Dr_ihha 129
Dr_ihhb 128
Gb_Hh 119
Mm_Ihh 122
Hs_IHH 122
Mm_Dhh 122
Hs_DHH 122
Mm_Shh 127
Hs_SHH 142
Dr_shhb 122
Dr_shha 123
Ts_qua-1 124
Bm_qua-1 132
Ce_qua-1 131
Cb_qua-1 131
Cr_qua-1 131
Ts_Xhog2 119
Ts_Xhog1 121
XC_Thog 121
Cb_hog-1 126
Ce_grd-2 125
Ce_grd-11 126
Ce_grd-1 126
Cb_grd-1 126
Ce_wrt-7 128
Ce_wrt-8 125
Ce_wrt-4 129
Cb_wrt-4 128
Ce_wrt-1 116
Cb_wrt-1 117
Bm_wrt-6 119
Ce_wrt-6 118
Cb_wrt-6 118
ruler 1.......10.......20.......30.......40.......50.......60.......70.......80.......90.......100.......110.......120.......130.......140.......150

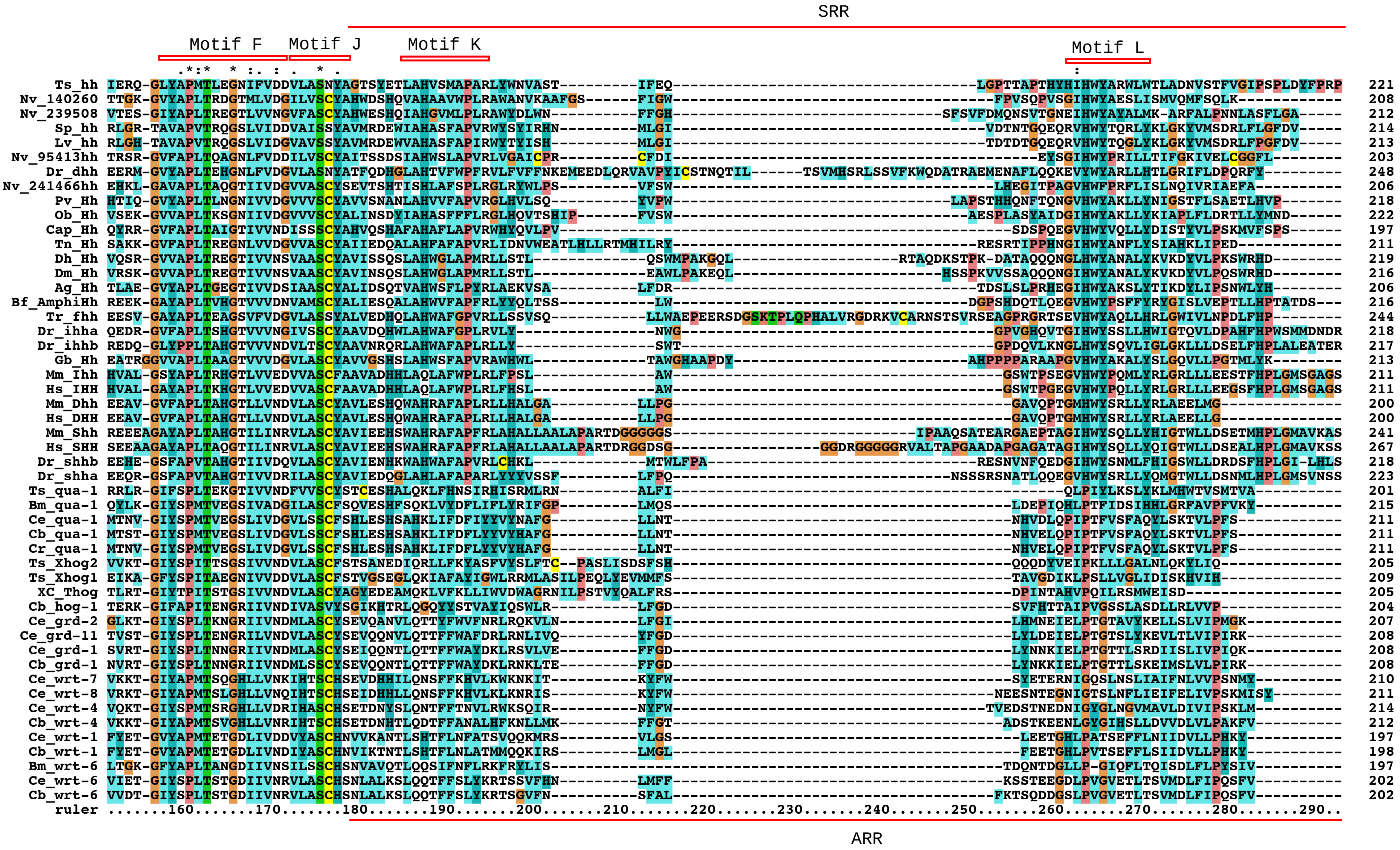
SRR
Motif F
Motif J
Motif K
Motif L
ARR
Ts_hh
Nv_140260
Nv_239508
Sp_hh
Lv_hh
Nv_95413hh
Dr_dhh
Nv_241466hh
Pv_Hh
Ob_Hh
Cap_Hh
Tn_Hh
Dh_Hh
Dm_Hh
Ag_Hh
Bf_AmphiHh
Tr_fhh
Dr_ihha
Dr_ihhb
Gb_Hh
Mm_Ihh
Hs_IHH
Mm_Dhh
Hs_DHH
Mm_Shh
Hs_SHH
Dr_shhb
Dr_shha
Ts_qua-1
Bm_qua-1
Ce_qua-1
Cb_qua-1
Cr_qua-1
Ts_Xhog2
Ts_Xhog1
XC_Thog
Cb_hog-1
Ce_grd-2
Ce_grd-11
Ce_grd-1
Cb_grd-1
Ce_wrt-7
Ce_wrt-8
Ce_wrt-4
Cb_wrt-4
Ce_wrt-1
Cb_wrt-1
Bm_wrt-6
Ce_wrt-6
Cb_wrt-6
ruler
