# Supplementary material for: Evolution of hedgehog and hedgehog-related genes, their origin from Hog proteins in ancestral eukaryotes and discovery of a novel Hint motif
Source: BMC Genomics. 2008 Mar 11;9:127. doi: 10.1186/1471-2164-9-127 (PMC2362128; doi:10.1186/1471-2164-9-127)
Supplement: Additional file 4 — Multiple sequence alignment of Wart domains. Wart domains were aligned and visualized in Clustal_X as described in Figure 2. [file 1471-2164-9-127-S4.pdf]

```
                                       *    :   :     .     *    *                                                   *
    Ce_wrt-3 ----MLYHVEMFTIILLF---GFSLADYCGSDQVPYGMEVHHSGVVRLMCSKPNCY--------------------------------------------DKNYSDCPERAESRH    64
    Cb_wrt-3 ---MWWQAVTIILVAFAI----ICRSDYCGSDQIAYGMEVHHSGVIRLLCSKPNCF--------------------------------------------DKNYSDCPERAESPQ    64
    Ce_wrt-5 --MCSMWLMASWLMAFVA---GSTLADYCGDHKVPFGMEVHKNGNVNILCSRPSCH--------------------------------------------EKKYAECPERATS-T    65
    Cr_wrt-5 --MRRLFLPTVTFILLLILQTTVTRADYCGEHKVPFGMEVHKNGNVNILCSRPNCH--------------------------------------------EKKYAECPERSTS-T    68
    Cb_wrt-5 ---MSNLHKLLYLLLPTV-----ILGDYCGEHKVPFGMEVHKNGNVNILCSRPNCH--------------------------------------------EKKYAECPERAMS-S    62
  Bm_wrt-5-3 ---MPVMISVIISLLIPI---VLYKGEYCGENKIPFGIEVHPNAQPLLHCSRPSCF--------------------------------------------ERRYADCDDRAQR-K    64
    Ce_wrt-9 -MRHRAFSFQIALVLALL---QPFGASYCGSNGVPYSLEILSDGSPVLGCAQPTCM--AEPQEDEEDSVFIANTAGQEDGFFREGDRQKKSYTQS-----YKPKAECPGEFSD-F   103
    Cb_wrt-9 -MRHGAFILSSFLLLLQL---YVIETSYCGENGVPYSLEILSDGSPVLGCAQPTCM--SEPLEDNEDSIFIANAAGQEDGYFREGDRQRRSYSQS-----YKPKAECPGHFSE-F   103
    Ce_wrt-2 ------MHTPIIFLLALV---PVALASYCGQSAIPYTFQVLRSGYPVLGCARPKCFGWTANGTRAGETAQFYRVAGKDDGYLRRSDQFIKSPSKNPNF--VPQLAICTDEYKS-K   103
    Cb_wrt-2 ------MHTPIFYLLALL---PVALASYCGQSAIPYTFQVLRSGFPVLGCARPKCFGWTANGTRAGETAQFYRVAGKDDGYLRRSDQFIKNPSKNPNF--VPQLAICTDEYKS-N   103
    Bm_wrt-4 --MLLLTSLSVCLIIIQN---AYIFASYCGEDAIPFSLQALQSGQPVLGCARPSCFGW---GVKTDKGARFYRIHRKNDGFMRRTDLKKYDKAKTMAR--ESQLAFCEKNYAS-S   104
    Ce_wrt-4 ---MRFSLLALVLLSSSY---KFTYGSECGDSTIPYSLEVLSSGQPILGCARPTCFGWHSNGHQLPTNAKFFRIDQQSDGFLRDDPLAIHTFDAADPRVYAQQQASCEQEFQS-L   108
    Cb_wrt-4 ----MRSLIWIVLLSISY---EAAFGSECGESTIPYSLEVLPSGQPILGCARPTCFGWHPNGHQLPTTAKFFRINQQSDGFLRDDPLAIHTFDAADPRVYSQQHATCEHEFQS-F   107
    Ce_wrt-8 ----MNYLLLVSGLLSVW---QPVFGSRCGESTIPFSLEILPSGHPVLGCARPTCFGWHPKGYQLPTTAKFSRLNRKLDGFLRDDSLFTYPFETDSSKIYKVQNSTCEPGFQS-S   107
    Ce_wrt-7 ----MNISKCVLVVALLSLCCKLSFGSSCGETTIPFSFEILPTGQPVLGCARPTCFGWDPKGYHLPTDARFVRIDRKRDGFLRDDPIYTYPFTPDGSKMYLQQNSTCEPAFQS-A   110
    Ce_wrt-1 ---MVMNPLTATFLAALI---GTAASASCGSSGIPFRFEVLPSGQPVLGCGSPTCFGAENGGRDLRHDSSFMAGADGDDGFFRDGDLARVRVRDPDA---PAQMANCPREFSS-S   105
    Cb_wrt-1 ---MVMNPLTATLLAALI---GTAASASCGSSGIPFRFEVLPSGQPVLGCGSPTCFGSENGGKDLRHDSNFMAGPDGDDGFFREGDLARVRVRHSDA---PAQMANCPREFSS-S   105
    Ce_wrt-6 -MTLLNLFYCFCLLFGAVLADSIHDGGSCGTNSIPYKMEVDSEGKPVISCEAPSCLGVSSSAARR----------------------------------PRVLDVSCDPFKE--I    78
    Cb_wrt-6 MRLLLNLCLPFCILFASAIADSYHDGGSCGTNSIPYKMEVDSDGKPVISCEAPSCLGLTSSLKSR----------------------------------PRELSVSCDPFKE--V    79
    Bm_wrt-6 ----MLLPVMLFLMEAVSGQQQSAIDSSCSEYTIPFSFQSDKTGNPTLLCTSPACFD-EKRLYELKRDDVIPEIETETRSLLGSKN--------------HIHKAQCHNYYQN-I    95
   Ce_wrt-10 -MLLVSVISCLLISVLAKDAVTPRVGSQCTKNQVVRKLTVYEDGALEAECGPVPC---------------------------------------------GEVGKRCIDDQT---    66
   Cb_wrt-10 -MLLISIVSCLFITVLAKDA-NVRMGSQCSKNQVVRKLTVYEDGALEAECGPVPC---------------------------------------------GEVGRRCIDDQT---    65
   Bm_wrt-10 ----------------------------CLLKVIT-KLTVYEDGAIEAECNKLPC---------------------------------------------GISGTQCKDNQA---    38
       ruler 1.......10........20........30........40........50........60........70........80........90........100.......110.....
                  signal peptide
                                                                                                  W A R T   d o m a i n

              *        *.               **                 :  ..   *                ** : :                  :    *
    Ce_wrt-3 GCQKSNQWVGGFEKNIEG----DLYTMCCEFEGLEKYAKVRYSDVRIRRGEFFEGEEKENDD--GDV--VKFDVIKDIRMHKDDEG--QAYYNLTVLSFNCESIPDVKPAWYQ--   167
    Cb_wrt-3 GCKKSNDWVGGFDKNIEG----DLSVMCCEYEGLEKYAKIRYTDVRIRRGEFFEGEEKENAD--GDV--IKFDVIKDIRMHRDSDG--HAYYNLTVLSFDCESIPDVKPAWFQ--   167
    Ce_wrt-5 TCSTNSSWVGGVTQHSDG----SLRLMCCEYDLLPTYSTIQYEKLTIRTGEYFEGDKQMEG---DVV--TAFDLIGNIEQVKEPDG--KYSYNLLIYRYHCGNIPDTPPAWYM--   167
    Cr_wrt-5 SCSTNSSWVGGVTQHSDG----GLRLMCCEYDLLPVYSTIQYQKLQIRRGEYFEGDEQMDG---DTV--ISFDLIGDIEQIKEPDG--NFSYNLLIYRYHCGNIPDTPPAWYM--   170
    Cb_wrt-5 ACTTNSSWVGGVTQHVDG----SLKLMCCEYDLLPIYSTVQYEKLQIRPGEYFEGDEQMDG---DTV--TAFDLIGDIQQVRDAAS-GNFTYNLLIYRYHCGKIPDSPPAWYM--   165
  Bm_wrt-5-3 SCESNDSWVGGFEKGYGN--HQPLYVQCCTFEGLAD-HSSPLYHTIIKPGQYFEGEEQVEE---ETDTVISFDVITDFKMIRSTNL--SIFYEMAVRRLRCYELPPVDRIKR---   168
    Ce_wrt-9 ACTKKNQWVGGIDFIDHP--RQPLVLQCCTFEGLR--FSQDVGVTTISAGEAVTGGEVVRD---NRQ--ISFDVIANVRKLVDPDDPKRTYFEVTVRRMNCLPDPPEFEVAYDDD   209
    Cb_wrt-9 ACTKKNQWVGGIDFIDHP--RQPLVLQCCTFEGLR--FSQDVGVTTISSGEAVTGGEVVRD---GRQ--ISFDVIANARKLVDPDDPKRTYYEVTVRRMNCLPDPPEFEVAYDDD   209
    Ce_wrt-2 TC-EEGEWVGGLSPQSDP-FADQLEMKCCSYQVLI--SAEDRGNAIVKQGQLVVGGEVLDG---SRL--VAFDYISNLSKSVSENG--TVVYVASIKRMPCFDEEQAVENKKREN   207
    Cb_wrt-2 TC-EEGEWVGGLSPQSDP-FTDKLEMKCCSYQVLI--NSQDRGNAVVKQGQLVVGGEVLDD---GKL--VAFDYISNLSKTVSKNG--TVVYVATIKRMPCFDEEQTETKKQEN-   206
    Bm_wrt-4 SCDENMQWVGGLSPSSNI-TTQPLLLKCCTFDNLK--NSWDRGIADVNPGQIVVGGEVMKD---ERQ--YAFDYIANIKKYSKKNG--SITYSVTIRRFWCLP-----------   197
    Ce_wrt-4 SCNPEDQWVGGIAPVMNASTTKIVAYKCCTYAPLR--ASIDRGVATVSGGQIVVGGEIFAD---NKP--YAFDYISNVEKKIDSEG--EIFYEVNIKRFSCLDLQKVDRS-VPE-   212
    Cb_wrt-4 SCNQEDQWVGGISPVMNATNTQVIAYQCCTYPPLR--ASTDRGLATVSGGQIVVGGEVTEN---NKQ--YAFDYISNVEKKLSAEG--EVFYEVNIRRFSCLDLQKADRS-VQE-   211
    Ce_wrt-8 KCDSKDQWVGGIEPETDA--FQDVAYQCCTYAPLR--ESTDRNIATVSAGEIVIGGEVYQN---ESQ--YAFDYISNIEKSMDENG--EVYYEVNIRRFACLDPHNADRR-IDE-   209
    Ce_wrt-7 MCDSKIQWVGGVEPVQDVNSTHDIAYQCCTYPPLR--ESTDRGMTLVAAGQIVIGGEVFKN---GSQ--YAFDYISNIAKNIDEYG--KIFYEVNVRRLACLDPHNADRS-VDE-   214
    Ce_wrt-1 SCSNPMTWVGGFKASDNG----DLSLQCCHYEGLR--FAQEVGRPVVHPGEVYSGGEVLRD---GRQ--TGFDAISNVRKITSGDG--TVAYEVTVTRMNCLPNPGEESNEVSFD   207
    Cb_wrt-1 TCSNPMTWVGGFKASENG----DLSLQCCHYEGLR--FAQEVGRPVVHPGEVYSGGEVLRD---GRQ--TGFDAISNVKKITSGDG--TVAYELTVTRMNCLPNPAEDTNEVSFD   207
    Ce_wrt-6 VCVKDLQWTSGLVEINNG-THRTLKTECCSYEGMS--DAKTIKSIFLGPGQSFVGGMVEKD---GEQ--SGFDLIKEIRKTVNADN--QVQYIVGVYRMPCEATSDSSEEALPL-   182
    Cb_wrt-6 VCVDELQWTSGLVEINNG-THRTLKTECCSYEEMA--TAKNVKSIFLGPGQSYVGGLVEKD---GEE--SGFDLIKEIRKTVNADN--QVQYIVGVYRMVCNARSDSSEE-LPV-   182
    Bm_wrt-6 SCTGEIQWTVGLLLEDDG-NNIKAKWKCCNYEGLR--HARAMKTVIVKADESYAGGEVYQD---GRR--VAFDLIKEVYLLFDEQH--RPRYELKIMRLACIPKPKSNKIIWVI-   199
   Ce_wrt-10 SCRAETDVFSGMRWAPNG---ESILLRCCTMHAK---NKIYVGTDVVAAGSFYEGGEVAEKDLYGDKGGAEYDFVANARTEQGGVR-------VWVYRMICAKGEKPVDFDPIT-   167
   Cb_wrt-10 SCRAETDVFSGMRWAPNG---ESILLRCCTMQAK---NKIYVGTDVVSAGSFYEGGEVAEKDLYGDKGGAEYDFVANARTEQGGVR-------VWVYRMICAKGEKPVDFEPIT-   166
   Bm_wrt-10 SCRAETDTFSGMKWASNG---QSILQRCCIISVPR---KLYIGTDLVSLGSYYTGGMVDPKDLYSKE-GPEFDFISNVRTEQGGVR-------IWVYRVVCPKTTGISDR-----   134
       ruler ..120.......130.......140.......150.......160.......170.......180.......190.......200.......210.......220.......230
```
