# Supplementary figures and images for: Evolution of hedgehog and hedgehog-related genes, their origin from Hog proteins in ancestral eukaryotes and discovery of a novel Hint motif
Source: BMC Genomics. 2008 Mar 11;9:127. doi: 10.1186/1471-2164-9-127 (PMC2362128; doi:10.1186/1471-2164-9-127)

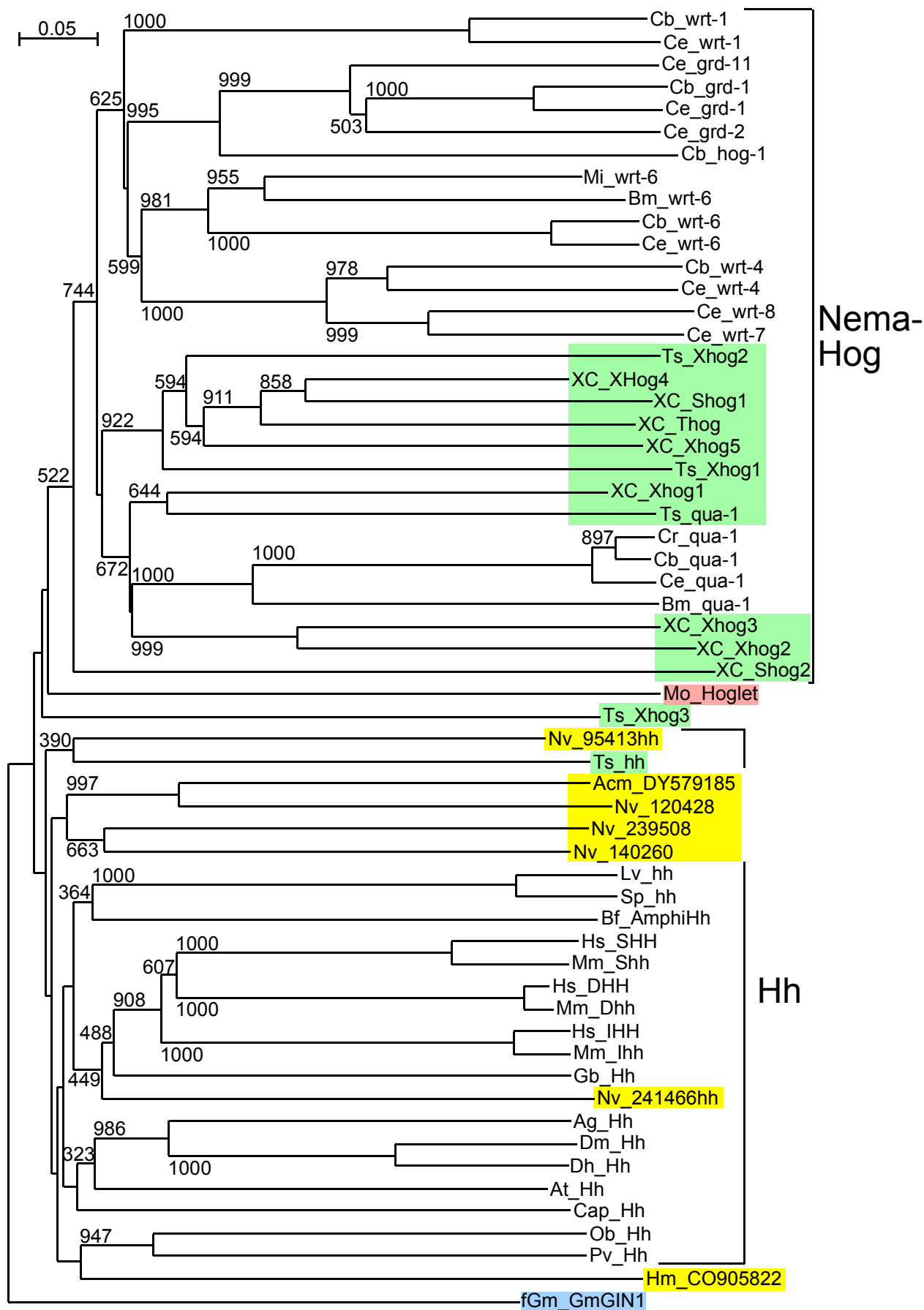

Supplement: Additional file 5 — Phylogenetic tree analysis of Hog domains using Neighbor joining. Neighbor joining tree without protist sequences. The Hog domain of the fungal gene GmGIN1 was used as outgroup. [file 1471-2164-9-127-S5.pdf]

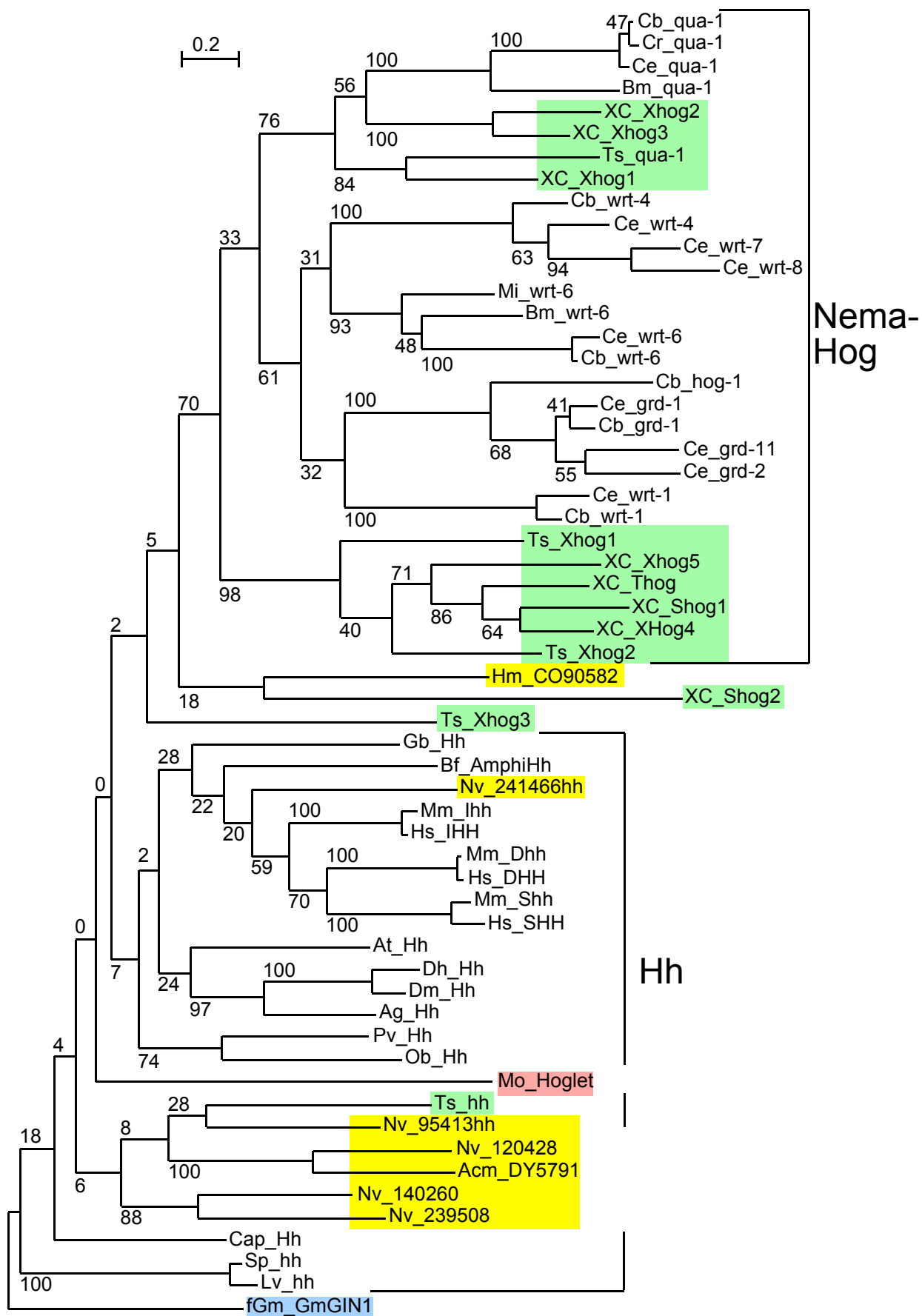

Supplement: Additional file 6 — Phylogenetic tree analysis of Hog domains using Maximum likelihood. Maximum likelihood tree of the same sequences as in Additional file 5 with GmGIN1 as outgroup. [file 1471-2164-9-127-S6.pdf]

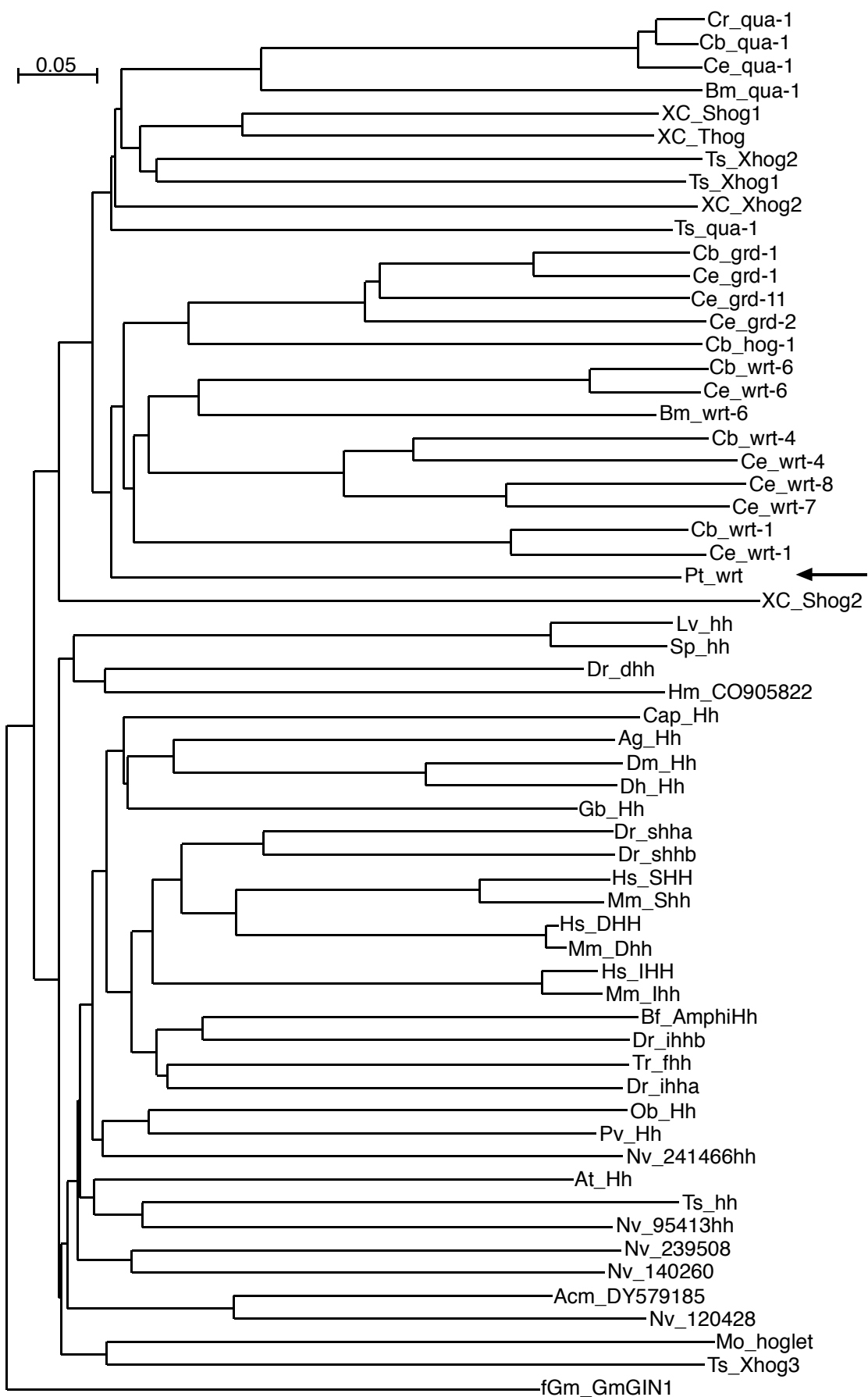

Supplement: Additional file 7 — Neighbor joining tree of Hog sequences which were truncated at the N-terminus. Hog sequences were truncated at the N-terminus to have the same size as the Pt wrt sequence fragment. This analysis shows that Pt wrt clusters with the wrt genes (arrow). GmGIN1 was used as outgroup. Note: Apart from Figure 5 and 6, and Additional files 5-7 further phylogenetic analysis were carried out that are not shown here. For example, the intein from vacuolar ATPase from C. tropicalis was used as outgroup [22] and gave comparable results to the tree analyses shown here. [file 1471-2164-9-127-S7.pdf]

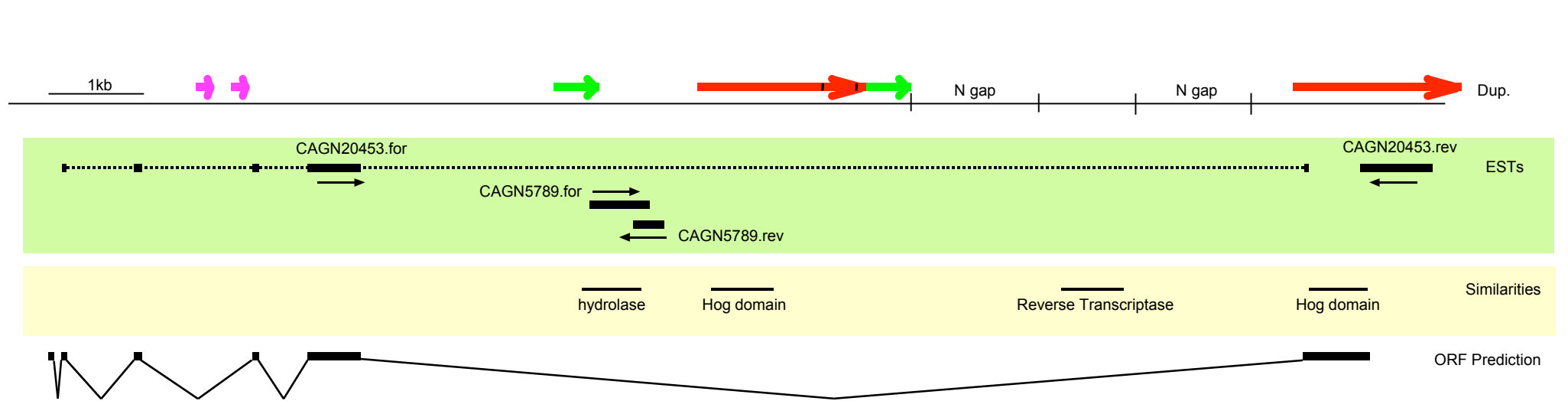

Supplement: Additional file 12 — Structure of the Nematostella vectensis genomic assembly around Nv 239508. Current assembly of the genomic region around Nv 239508. Color arrows indicate duplicated regions. N gap indicateds two regions with unknown sequence. The green area shows the ESTs found mapping to this region. The CAGN20453 correpsonds to Nv 239508. The yellow area shows regions of sequence similarity, i.e. hydrolase domain, Hog domain, and Reverse transcriptase. CAGN20453 is not sequenced fully, but the 3' read has been mapped to the right side, since the 3' untranslated region matches better to the 2. repeat of the duplication due to some indel differences. However, as will be noted, the final resulting transcript (shown at bottom) would be rather unusual, as it would splice over another gene, i.e. the hydrolase, which is also supported by an EST. Hence, the genomic organization and gene structure in this region could be subject to change, especially given the unsequenced areas. [file 1471-2164-9-127-S12.pdf]

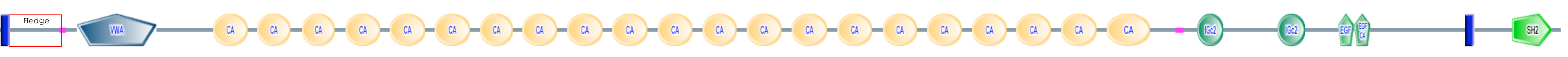

Supplement: Additional file 13 — Predicted protein structure of Nv 200640. Protein motif prediction of the SMART server was used to analyse the ORF Nv 200640, and the different types of conserved motifs found are indicated. [file 1471-2164-9-127-S13.pdf]
