# Supplementary material for: Evolution of hedgehog and hedgehog-related genes, their origin from Hog proteins in ancestral eukaryotes and discovery of a novel Hint motif
Source: BMC Genomics. 2008 Mar 11;9:127. doi: 10.1186/1471-2164-9-127 (PMC2362128; doi:10.1186/1471-2164-9-127)
Supplement: Additional file 8 — Multiple sequence alignment of Ground and Ground-like domains. Alignment of nematode Ground and Ground-like domains. [file 1471-2164-9-127-S8.pdf]

```
    Cegrl-2 KPAFVPSASYLEDVSEEGQLVEKQGHWSKGSD-SQTSSKYGQFESRHNILKRMKTESIPRTNNTCNSIKLANVMMRAIV---DDVS-VS------KRM   87
    Cbgrl-2 KPAIVPSMSYMEDSSEEGQLVEKQGYWMKGHPVSQTSSKYGQFEARHNILKRMKTEAIPRTNNTCNSIKLANVMMRAIV---DDVS-VS------KRM   88
   Bmgrl-x7 DNMRCKCISESKSKCK-------------LTCLDGTNKTDTN--HNIERREIDYEEVEIIDERKCNSKSLQNIMLQNMA---GQTAKQA------KTK   74
   Cegrl-28 ----------CGCG----GRKKRSLPEKP----TFYKISASG------------------EDLVCNTPELKKIIVENME---SSAI-ES------SKS   52
   Cegrl-30 ----------CGCG----GKKKRSLPEKP----TFHGIAASD------------------EDVMCNTPELKKIINENMQ---ASAV-DS------SKA   52
   Cbgrl-27 ----------CGCG----GRKKRSLPEKP----TFFGIAAGD------------------DDMMCNNTELKKIILENMQ---STSV-DS------SKA   52
   Cegrl-27 ----------CGCG----GRKKRSLPEKP----EFFGIAAGD------------------QDVMCNTPELKKIILENTQ---TTAV-DS------SKA   52
   Cegrl-31 ----------CGGGCGGCGRKKRSLPEKP----TFVGIADSG------------------DDVMCNTPALKKIILENIQ---TTAS-DS------SKA   56
   Cegrl-29 ----------CGC-----GRKKRSLPEKP----TFFGISASG------------------DDVMCNTPELKKIIARNMQ---DSAM-DS------SKT   51
   Cegrl-26 ----------CQSAC---GRKKRSIDEDERREFEFRGIASKN------------------EDLLCNSPEMKAMIQAHMK---SNPQ-AS------SKS   57
   Cbgrl-26 ----------CQSGC---GRKKRSVDDEEAPGFQFRGIASKA------------------DDMLCNSPDMKALIQAHMK---SSPQ-AS------SKS   57
  Cegrl-32p ----------------------------------------------------------------------------NIQ---TTAV-DS------SKA   12
   Cegrl-20 ----------CGGGCGCGGRKKR----------------AAS------------------EDSKCTDPELRKIILNGVR---TTTS-ES------RDN   44
   Cbgrl-20 ----------CGGGCGC-GRKKR----------------AAS------------------EDSKCTDPELRKIILNGVR---TTTT-ES------RDN   43
   Cbgrl-23 ------------------GRKKR----------EAVNVIAHD------------------DLNKCNNEELRIILNENTK---ETVA-ES------IK-   41
   Cegrl-23 ------------------GRKKR----------EAINVVGHD------------------DLNKCNNEELRIILNENTK---ETVA-ES------IK-   41
   Cegrl-25 ----------PSGGGYSGSGGAGGAPNGANYDEAQEDVEEYEGGAAGFRNRRFSKGGTADDTPICNSVRLRKLIRQSLT---EDPE-SS------MQR   78
   Cbgrl-25 ----------AQQGGYS---GAGGAPNGANYDEAQEDVEEYEGGSAGFRNRRFSKGGAADDTPICNSVRLRKLIRQSIT---EDPE-SS------MQR   75
   Cegrl-15 ----------CCPTCACGGRKKREIDAGVQEEES-----VST------------------KDVSCNDDSLLAIMKKEMT---TGES-SA------VKI   55
   Cbgrl-15 ----------CCPTCAC-GRKKREIDTGV-AEDT-----VST------------------KDVSCNDDGLLAIMKKEMT---TGES-SS------VKI   53
   Cegrl-17 -------VEATANPLDAQLGEDIAAQQQAAT---EDDGDDES-ASSGDFKARAAK--TAVTDEKCNSKILQKLVLTNIA---ANDALAS------KKA   76
   Cbgrl-17 -------VEATANPLDTQLGEEIAAQEQAATGNSEDTEDAES-ASSGEFKARAAK--TAVTDEKCNSKILQKLVLSNIA---TNDALAS------KKA   79
   Cbgrl-22 ----------MC-GCGC-GRKKRSVEGEPAVEG----------------------IINMDSILECNNQDLKGILEKNMK-TPSDSLLSV------R--   55
   Cegrl-22 ----------MC-GCGC-GRKKRSVDD---VEG----------------------VINMDSDVECNNEELREVLENNMKSTPSDSLVSV------R--   53
   Cbgrl-19 ----------VCGGC-CGGRKKREIGH---VKGHIVHRD-EQ-----------------EWSNQCNSQEFSEVILKHIE---MSSLKSS------REA   57
   Cegrl-19 ----------VCGGCGCGGRKKREIGH---VKGHLAHRDNDQ-----------------EWNNQCNSLEFSDVIVKHLR---TKSLSTS------RDF   59
    Cegrl-7 DEGKTTHMKTVKAPA---AASS---TTAAAASTSTEFPVGEEIVADTNEKEVDISELHLTDDPLCNSDDLRKVVIDNID---DQLN-SS------KRM   82
    Cbgrl-7 DEGKTTKVKTVKTPAASPASST---TTAASSSTSTEFPVGEEIVPDTNEKEVDISELHLTDDPLCNSDDLRKVVIDNID---DQLN-SS------KRM   85
    Cegrl-5 --------QAYAAP-----------HKRHVDVMDIDTGVDQS-EG---YVARVKR-EENVFDPKCNSEDLKAIIVANIH---ESTAVA-------KRQ   64
    Cbgrl-5 --------QGYAAP-----------HKRHIINNMEDTGVDQS-EG---YVARVKRDEEAVFDPKCNSEDLKAIIIANIN---ESTAVA-------KRQ   65
   Cegrl-13 ----------IQKSKPETTDMKNDLDVDVNALSNFETSLQAYKSMNR-NGIRRAQAAKTEKAGKCSSERLQQIMEEAMS---SN-VSVS------KLK   77
   Cbgrl-13 ----------IQKSRTDTSPAKNDLDVD-NALSNFETSLQAYKSMNGIRGIRRAQGAKIEKPEKCSSTRLQQIMEESMS---SS-VSVS------KLK   77
   Cegrl-21 ----------SYAGPSYAGSYG-PGPY-RAKSKRSVDMKASS------------------GDLFCNSVEVRDIIKKGMT---SGEK-ES------RET   58
   Cbgrl-21 ----------YSTGPSYAGSYGAAGPYKRARSKRSMELKASS------------------GDVLCNSVQVRDIIEKGMT---SDEK-ES------RET   60
   Cegrl-18 FYKSSTSSPAQTFEYGAVRDARDSTGKDETSTPKFPFDRDENV---NKWTSKRKIREKEEINSKCNNPILKDLMEMKMT---TSPS-IS------KQM   85
   Cbgrl-18 FYKSSTSRPIETFEYGAVRDARDSSDK-ETSTPKFPFDREEEVSPKSKWTSKRKTREKED-SSKCNNSILKDLMELKMT---GSPS-IS------KQM   86
    Cegrl-4 ------CCCRCGSPCR-FMARH---RTHGSKLFTTE---DPE--ED----------------PTCNSKKLRRVMERNMN---GDPS-IS------KRA   57
    Cbgrl-4 ------CCCRCGSPCR-FMARH---RTHGSKLFTTE---EQE--ED----------------PTCNSKKLRRVMENNMN---RDPT-IS------KRA   57
    Bmgrl-4 ------CCCTCVAPCM-Y-SQM---RIHGAKIFSASLVADLE--DH----------------SKCNDPVLKSIMEENMT---EDAT-TS------KRS   59
    Cegrl-6 ------CCCGCSSPCK-YKSVR---RA----AFAAKTI-------D----------------PSCNSSELKNIILDNIS---EDAS-ES------KRN   51
    Cbgrl-6 ------CCCGCSSPCK-YKSVR---RA----AFAAKTV-------D----------------PSCNSSELKNIILDNII---EDAS-ES------KRT   51
   Bmgrl-Ax CKTDSEVCTKCNQQSKPSKSPN---RSMAGTYFRTNKRLSRK--SERLNRRNKREETKIVMSDTCNSHDLMEIMDQNLS---TNLA-IS------KHL   83
    Cegrl-9 -------IQAQTEEP-ATTQKATDAPPPPILQNKIERVFESE---PNSGYRKPVRRQAPPLSEKCNDDRLRKIIEQNVD---DNPS-TS------KRK   77
    Cbgrl-9 -------VQKNPEEARNNVEKVTDEPPPPVLQNKVERVFESE---PNNGYRQPVRRQAPPLNDKCNDDRLRRIIESNVD---DNPS-TS------KRK   78
    Cegrl-1 ------CCGKCGAPCK----FR---SKKNVIALASKIFTAQF--VP----RRDGEDEEEPKDPKCSSEKLKDLMNKYIT---RTVA-LS------KRL   69
    Cbgrl-1 ------CCGKCGAPCK----FR---SKKNVIALASKIFTAEY--VP----RRDADNEDEPKDPKCSSEKLKDLMNKYIT---RTVA-LS------KRL   69
   Cegrl-12 -------CCNTCGTCN---GKR---RRRHLTMLSNATYVADH--EKVNRVKRQDDQETTVTSGNCNSVELQRIIESKID---RVTA-IA------KRR   73
   Cbgrl-12 -------CCNTCGTCK---GRR---KRRHL-MLSNATYVASH--DPARRVKREDDTE-AASDGRCNSEELRKIIENKID---RVTA-IA------KRR   71
   Cegrl-14 PSSQPIQLHILPPIPS---PQPQIIYQQTTPSSQFVAYSTFAPPIEPSNECGNGQCKPESDEDKCNSQRLRDIIFNNIVSGDAEAS---------KRA   86
   Cbgrl-14 PSPQPIQLHILPPIPSPPAPQPQIIYQQTTQAPQFFTFSTFAPTTDNQNDCSNGQCRPESDDEKCNSQRLRTIIFNNIVNGDAEAS---------KRA   89
   Cegrl-10 DGGLYRSKNIRRAPVEPTVHVHRDSSVETTTISSEISGMETD---------------ETLDKNKCSSSVLRKLMIENIS---DSSS-ES------KRN   73
   Cbgrl-10 SGGWYRAKEIRRAPAEPTVHVHRDGSVESTTLGS----VATE---------------ESFDKNKCSSAVLRKLMLEEIT---DSSA-ES------KRN   69
   Cegrl-11 -----------------------------DYTDSYSTSLIQNPNI-RTKPIPRSR-----KDDDCSDELLRKVMNEHIS---DEGMTES------KRA   54
   Cbgrl-11 -----------------------------DYIEPYPGSLIQNPNV-RTKPVVRPR-----KDEDCSDEILRKVMNEHIS---DEGMTES------KRA   54
    Cegrl-3 ISHMMKEMEGMLMLMRDEISIQIENSSPVKLLEEAATRTEIDSSLHASPMTRAIRHKRAETE-NCNDEKLRKLIKENIR---RDAK-SS------KRE   87
    Cbgrl-3 ISHLMKEMEGILSLMRDEVTTQIENSSPVKLLEEAATRTEVDSSLHATPMTRGMRVKRAETD-NCNDEKLRKLIKDNIK---KDAK-SS------KRE   87
    Cegrl-8 ----------RPKTESDSEEEQ-------PKPTRNRVRLHSHNS--KVSKVSRTREVEV-LSEKCNDDKLEQIMQDAMT---PS-LSTS------KMV   68
    Cbgrl-8 ----------------DQQENE-------RKVSRNRVRLHSHNS--KISKLSRTREVEV-LSEKCNDDELQQIMQDAMT---PS-LSTS------KMV   62
   Cegrl-16 TPAPVVEETQAPVAAAKEESTYDDIVEEQPSTATETATEQVDTVEET--NEAVEYADEEGDEGNCDDPELRAIVEAALK--NEKDNLEA------ARK   88
   Cbgrl-16 TPAPAPVE-QAPVAAAKEESTYDDIVEEQPATATEAATEQVDTVEET--NEAVEYADEEGDEGNCDDPELRAIVENALK--NEKDNLEA------ARK   87
   Bmgrl-16 SAAESDHGATTALATAMRSAEFTSLESSGLDAKNNAISEAIAGYDKSNGNAIIESRSIETDDSTCNDPVLKAIIESTLK--EHRDNLDA------ARS   90
   Cegrl-24 ------------------------------------------------------------EGERCNDVILYDIIKKASK-KTDDPVIIR------RTS   31
   Cbgrl-24 ------------------------------------------------------------DGEKCNDVILYDIIKRASK-TTSDPAEIR------KTS   31
    Bmgrl-7 AQSSSSVLPYKQQRPNFAKEENIKDKEVADLLDAIEEFHNQTLQIQEPIKPIPISSLITIGDSECNDNKLKIIMLENIG----KDLNAT------KKM   88
   Bmgrl-14 NRIDQKFGDRYGGRANFEQPLTQLMSNDYNIDTASYIPEVNPNCINDPCEIDNGNPFGFIEDERCNSPRLKEIILQNIV---ERDAEAS------KQA   89
   Bmgrl-17 GTETDIQNTSDIQQFPSIQNEAEKLNDPTFLNPDITHQLSPLSSTAASITERSAHGVVIISENKCNSLVLRELLKKNMD---ETDPVIS------KRS   89
    Bmgrl-2 LNDLTTISATSMYPLTTTDIEDTLEDSKNNLQRTSFNVLKRQKKTKDAXKRNYPAKRAIFPSAVCNSRRLKKVILQAIT----DDVSES------KRR   88
   Bmgrl-x1 NTVIQQPVLVTYRVIVPVVRKIPIIENTCCSTCAIPCIARKKRELLAEDELLINNSTKLPVNPVCNSKSLQEIMSENIS----TTAAES------QRL   88
   Bmgrl-x3 TVPIPVVKPIYKIINKPIILN------ECCKTCGKQCIKRMKRNISNDTIITV--------NSVCNNKILGEIISKMMT----SNLIVS------QKL   74
   Bmgrl-x2 PPLPPLPPLPSLPCPCLPCPCMPMLLNSCCNTCQRPCSFRSRRRRSLNARALARGMQILNDDPVCNNAELQSTIKQNLV----DDIWLT------KEM   88
    Mi_Msp3 PQPCIPQPPPIALPSLCFPQIQLPCPPPSCGCCGRRKRESGASALLTAVSTKSGIKRIGEEKNHCNNPHIKRIILKNLIIGDWVGT---------RNA   89
   Bmgrl-x4 LCLPCPPPIPMQPLCPPSLQ---HLLLPCPLPQPSNSPCKRKKRSTPITSVAS--DYGTNIHASCTSNYIRKIILKNLS----TDAKIS------KAA   83
   Bmgrl-x5 IKPPSVKKSLAQSNIQEQIVNNFPSH-DCCTRCNGAN-CMPRNGNAI-LLAANTFQNRHDHDTKCKSKELAKLMMANMKR---DPTRS-------KRA   85
    Cegrd-3 --------------------------------------------QSDTCPSLKDRQLAKPGQLFCCDSTIKTVVETGMQTLDLFGAGGPQTLGPIVQA   54
  Cegrd-17p --------------------------------------------QSDTCPALKDRQLAKPGKLFCCDRIIKTVAETGMKTLDLYGPNGPRTLGPIVQA   54
    Cbgrd-4 --------------------------------------------QGDTCPSWKDRQHAKPGKLFCCDSTIKTVVETGMKTLDLFGAGGPQTLGPIVQS   54
    Cegrd-4 --------------------------------------------QDDNCPSMKSRKHAKSGDLYCCDSTIKTVIKRGIRTLSYFGDDGPQTLGPIVQG   54
   Cegrd-14 -------------------------------------------DLPKPADPYEPPGYEPE---ACPDKYSKIITQLRSD----LGKDVSS--IKFTNQ   46
   Cbgrd-14 -------------------------------------------DLPKPADPYEPPGYEPEPDKPCPDKYVKIINDLRAE----LGKDVSS--IKFTNT   49
   Cegrd-13 -------------------------------------------DLPKPADPYQPPGTEPKTESTCPDPYKKIITQLRAE----LGKDVSS--IKFTNQ   49
    Cegrd-5 ------------------------------------------------------NCYINDSGFTCCNKELESAMKGAMGGD--------D-LLGSADS   35
    Cbgrd-5 ------------------------------------------------------NCYINDSGFTCCNKELEASMKGAMGGG--------D-LLGSADA   35
   Cegrd-10 ------------------------------------------------------NCYINEGGFTCCNKELESVMKSSLGGS--------D-LVGSAGD   35
    Bmgrd-5 -------------------------------MCWWGNVLINFVFLFASLNAYREDCFINVGGYVCCDMDMVKVMQNVMEED--------DNLLNVAKK   59
    Cegrd-8 YQSLLGGKGGPGGFSPLSFFLNGGLGGTGGGGNNGFFVPVPVVIPPPPPPPPGPNCFTNPSGFLCCNVTLEKTMEDAYLAAK-ADGASLCNVQKMATA   97
    Cbgrd-8 YQSLLGGK---GSFSPLSFFLNGGL---GGGHNNGFFVPVPVVIPPPPPPPPGPKCFTNPSGFLCCNVTLEKTMEEAYIAAR-DGGASSCNVQKMASA   91
   Cegrd-15 ---------------------KP---------KYAEH-----K-------YPLKQCYTETSGFMCCNPKLEKVMSETALKMK---SSKSCNLQKMSSM   53
   Cbgrd-15 ------------TNSYQNGYKQPA-------DEYADEYPWPGK-------YPLRQCYTETSGYMCCNLHLEKVIHNATQKMK---ESKACNLQKMATM   69
    Cegrd-6 RPKTTTAAPTTTTEEVTQGYEEEAVELPKYDETFVGQYYYGRRGEGGNNTFPLPSCFYNPSGYVCCNLMLNELMSTSFEEVK--VATNLCNVHKFATK   96
    Cbgrd-6 RPTTTTAAPTTTT-EVTQGY-EEAVETPQYDETFVGQYYYGRRGEGGNNTFPLPSCFYNPSGYVCCNLMLNELMTTSFEEVR--VATNLCNVHRFAIK   94
    Cegrd-7 ------------------GWKGDT------------EYNYPER------NLPLKGCFYNNHGYACCNLKLQNKMEELADELLNNGTFHRCNVQKLAND   62
    Cbgrd-7 ------------------GW-GDT------------EYNYPER------QLPLKGCFYNNHGYACCNLRLQNKMEELADQLLNNGTFHRCNVQKLAND   61
    Cegrd-9 AYEDGDDNGEHNREEEEESSQYSSNIRDMEQPTKYSSKPNVKYSYPPKDTLPLQTCFHNPSGYVCCNLELNNVVESTYKEVRELPNFNPCNLQLIANK   98
    Cbgrd-9 SGEHGDGDG---HREEEEKSEYSSNIREGEKQTKYSSKPNVKYSYPPKDTLPLQTCFHNPSGYVCCNLELNNVVESTYKEVKELPNFNPCNLQLIANK   95
   Cegrd-12 ----------------YPTPPAYVR-PPPVYVTPPPVYITPPP--TTTTTIPPPKCFQNTQGYKCCNRQLDQFLEQKVGEM-LKPEWQRCNLQRFATQ   78
   Cbgrd-12 ---------------AYPTPPAYVRPPPPTYVRPPPVYVTIPPTTTTTTTLPPPKCFLNNQGFKCCNRLLDQYLDQVVTNM-QRPSWQRCNLQRFATQ   82
   Cegrd-16 -SKSSSE--SMFRTNVFRGNINRQIPAIPLPSPFSVAYGKPSFAIAREED---GKCYTNRFGYRCCDEALEALILKSYEKLRRKSNDLEDNLSKIAST   92
   Cbgrd-16 ISSGTGEVGSVFRTNVFRGNINRQIPAIPFPSPFSAAYGKPSFAIQREED---GTCYTNRFGYRCCDEALESLILKSYEKMRRRSDSLEENLAKIAST   95
  Cegrd-1R2 SLNITEIEL-------------------PNPTPIPRNFANLREKDRLPENTHCDKE--RKDGNRCCDGRLASTMRDAMRQMATSPDFGQGKEGIIAAE   77
  Cbgrd-1R2 -LNITEIEL-------------------PNPSPIPRNFANLREKDRLPENTHCDKE--QKDGNKCCDGRLASTMRDAMRHMATSPDFGKGKEGIIASE   76
  Cegrd-2R2 --FNTDLDR-------------------PGPQPVPRNITNWRKKERYPKGTHCESG--KRDGNKCCDARLAATMRDAMRQMATSPEFGPGKEGIVAAE   75
 Cegrd-11R2 ------------------------------------------IRDRLPENTHCEKE--RRDGNKCCSGPLASTMRDAMSQMASSPDFGPGGEGIIANR   54
  Cegrd-1R3 -----------------------------G------------DRAGYPVGSHCT-Q--ARTGSKCCSLILFNAMKSGYDSHVATSNFDPYDIRNISKA   54
  Cbgrd-1R3 WHIPLQLER-------------------SG------------ERAGYPVGSHCT-E--ARTGSKCCSLILFNAMKTGYDNHVATSNFDAYDIRNISKA   64
  Cegrd-2R3 ----LQLEE-------------------SG------------DRAGYPVGSHCSND--TRTGSKCCNLRLFNAMKDGYDRHKASPNFDRYNIRNISKA   61
 Cegrd-11R3 -NVQLLLES-------------------SG------------DRVGFPVGSHCLEE--VRTGSKCCSIDMFNAMNAAYNTHMASSYFNAYDIRNISKV   64
  Cegrd-1R4 -------------------------------------------------PFNCPADLSGLSGMACCDGGLQFEANKVIDQAKQEPDFDKHNTRNLAKL   49
  Cbgrd-1R4 -------------------------------------------------PFNCPAGLSGLSGMACCDGGLQFEANKVIDQAKQAPDFDKHNTRNLAKL   49
  Cegrd-2R4 -------------------------------------------------PFHCPPDLSGLDGTACCNSGLQYEINRILDEARYSDSYHKHDTRYLAKM   49
 Cegrd-11R4 -------------------------------------------------PIFCPSTLTGLFGSIICDGGVAYDMIRIVEETRQQPGFDKHNSRAMAKM   49
  Cegrd-1R1 ----------------------------------GRSADINHDSPCIWNKTQSWVHPYQSITVECCDEELSKLIHKTILDAGNNAKL--GN---LAKF   59
  Cbgrd-1R1 ------------------------------F---GTSTDTKHQSPCIWNQTQSWIHPYQSITVECCDEELGDLIRKTINDAGRNAKL--GN---LAKF   60
  Cegrd-2R1 ---------------------------------------YQVDAVCIWNRTQSWTVPYQSILVECCDKPLKQLILDTIHG-TNFESLANGD---FAKI   55
 Cegrd-11R1 ----------------------------KNI---GLSTDNKHQSPCIWNQTHAWSHPYQSLIVECCDESLKDLLQNFIRQ-GKAGSL--GD---LAKV   61
      ruler 1.......10........20........30........40........50........60........70........80........90.......1
```

Cegrl-2	IQHATKSAFDGAKFDVFCAIGEF-SYS--IHSRKYCEVTKQ-EVTCFAFR--	133
Cbgrl-2	IQHATKLAFDGAKFDVFCAIGEF-SYS--VHSRKYCEVTKQ-DVTCFAFR--	134
Bmgrl-x7	IMRAAEALL-GGYFNVICASGDF-SYI--TTTSLYCLQSLG-NINCYAFLTGNTRPRL------------------------------------	127
Cegrl-28	VNAVLETRQLN-RFVVVCSENPF-AFTA-RADSAYCGAAKN-GHNCHAFAM---	99
Cegrl-30	INGALESKELN-RFTVVCSENQF-VFTI-RADTAYCGAKNN-GHTCNVFSM---	99
Cbgrl-27	VNGALEAKQLN-RFVVVCSENPF-VFTV-RADTSYCVARKN-EHNCHAFAM---	99
Cegrl-27	INTVLESRQLQ-RFVVVCSENPF-VFTI-RADTAYCGASKN-GHNCHAFAM---	99
Cegrl-31	INTVLESRQIQ-RFVVVCSEKPF-VFTF-RADAAYCGATKN-GHNCHAFAM---	103
Cegrl-29	VNAALENKKLQ-RFTVICSENPF-VFSI-RADTAYCGVQRN-GHNCHVFAM---	98
Cegrl-26	LQSALEDHDSH-RYVVVCSENPF-HYSI-KHDSAYCGARNG-SHYCQAFAI---	104
Cbgrl-26	LQVALEDHDQH-RYVVVCSENRF-HYSV-KHDSAYCGSRNG-THYCQAFAI---	104
Cegrl-32p	INTVLESRQLQ-RFVVVCSENPF-VFTI-RADTAYCGASKN-GHNCHAFAM---	59
Cegrl-20	IVASLKEKYAGVRYLVTCIEGDH-DFA--SSSTDYCADGSQ-QQTCIVAKATDE--	94
Cbgrl-20	IVASLKEKYAGVRYLVTCIEGEN-DFA--SSSSDYCADGSQ-QQTCIVAKATD---	92
Cbgrl-23	---ALKEKVAGQDYIVVCNEKPA-PFT--AETDDFCQLQKE-NVHCTILRINHKEIVE------------------------------------	92
Cegrl-23	---TLKEKVAGQDYIVVCNEKPA-PFT--AETDDFCSLQKE-NVHCTILRINHKEVAEKNEE--------------------------------	96
Cegrl-25	LAQRVKSRLVSGEFYVACGEQGL-LPLA-GENREHCFIKSG-KFACYVLRKA--	127
Cbgrl-25	LAQRVKSRLVSGEFYVACGEQGL-SPIE-GEHREHCFIKTS-TFACYVLRKA--	124
Cegrl-15	ALVEAAEQELGGRFTVVCSQGAF-SFV--TSTTSYCLHSQA-GLNCYLFKTQ--	103
Cbgrl-15	ALVEAAEQELGGRFTVVCSQGAF-SFV--TSTTSYCLHSQS-GLNCYLFRTQ--	101
Cegrl-17	IHDNALQQFPDSSVDVICSTTGF-TYL--VSTTEHCEAQKD-GVICFVYKRPL---	125
Cbgrl-17	IHENALQQFPDSSVDVICSTTGF-TYL--VSTTEHCEAQKD-GVICFVYKRPL---	128
Cbgrl-22	------SSLNSDEYFVMCTHGLT-AYSA-PAGTKNCAVXKE-NHFCQVFSLNESH---------------------------------------	101
Cegrl-22	------SNLPTDQYFVTCTHGLT-AYSA-PAGTKSCAVRKE-SHFCQIFSLTNNSSNL------------------------------------	102
Cbgrl-19	IYKELDAAYPDSMFTVFCLKNSTSSYQA-DA-KRYCMEKSK-DRMCYVFEF---	105
Cegrl-19	IYKELDTAFPDSMFTVFCLQNSTVSYQA-DA-KRYCMEKTA-DRSCYVFEF---	107
Cegrl-7	IQLAAEAQF-GGRFDVICANGDF-SYV--TNTELYCQETKG-DISCYTYRQL--	129
Cbgrl-7	IQLAAEAQF-GGRFDVICANGDF-SYV--TNTELYCQETKG-DISCYTYRQL--	132
Cegrl-5	IQTAAADAI-GGRVDVICSKGTF-SYI--VNTELYCETEKD-GTTCFAFKQSS---	112
Cbgrl-5	IQSAAADAI-GGRVDVICSKGTF-SYI--VNTELYCETEKD-GTTCFAFKQSS---	113
Cegrl-13	ISRGAKKEF-GYNFDVICSQFDF-SYL--ISSNIFCRVELD-GQICLAYEN---	123
Cbgrl-13	ISQGAKKEF-GYNFDVVCSQFDF-SYL--ISSNIFCRVELD-GQICLAYEN---	123
Cegrl-21	ITALLKTEM-NREYVVICTKQPF-EYLA-SSDSEFCSVTNDSGITCSSFVF---	106
Cbgrl-21	VVALLKAEM-EREYVVICSKQHF-DYLA-SSDSDFCSVTNDAGITCSSFVF---	108
Cegrl-18	IYSAATEMWMGRNVNVICSKHSF-SYVV-VTSPIFCEHRKK-ALTCFVFFQP--	134
Cbgrl-18	IYSAATEMWMGRNVNVICSKHSF-SYVV-VTSPIFCEHRKK-ALTCFVFFQPD---	135
Cegrl-4	IQKAVEEKM-FGKFNVICARGDF-SYV--AYTETYCQVAND-DVTCYAFRPM--	104
Cbgrl-4	IQKAAEEKM-FGKFNVICAKGDF-SYV--AYTETYCQVAND-DVTCYAFRPM--	104
Bmgrl-4	IQRAAEEKL-FKKFNVICSESDF-SYI--AYTDTFCQHSND-DITCYAFSPYSGI---------------------------------------	109
Cegrl-6	IQKIAEETL-GHEVNVICGTGEF-SYI--AHTDTFCQAFKE-DVTCYAFKPLQ---	99
Cbgrl-6	IQKIAEEQL-GHEVNVICGTGEF-SYI--AHTDTFCQAFKE-NVTCYAFKPL--	98
Bmgrl-Ax	IQKYAELKL-KRDFNVICSNNSF-TFI--THATLYCQTVKF-NVSCYAFTAD--	130
Cegrl-9	IQKAAAEEI-GGLFDVICSAHDF-SYL--ANTQLFCESGND-DVTCFAFLHSLIQ---------------------------------------	127
Cbgrl-9	IQKAATEEI-GGLFDVICSAHDF-SYL--ANTQLFCESGND-DVTCFAFLHSLIQ---------------------------------------	128
Cegrl-1	IQKNAESEL-GGYFSVFCSIDDF-SYV--ARSEMFCQLQKN-DITCYAFKHK--	116
Cbgrl-1	IQKNAESEL-GGYFSVFCSIDDF-SYV--ARSEMFCQLQKN-DITCYAFKHK--	116
Cegrl-12	IQEEAEATM-GGRFNVICARGDF-SYV--ANTELFCQHSVG-DVTCFLFKQLSDVVRRRLM---------------------------------	129
Cbgrl-12	IQEEAESTM-GGRFNVICARGDF-SYV--ANTELYCQHSVG-DVTCFLFKQLSDVVRRRLM---------------------------------	127
Cegrl-14	VQSAAEAET-GLFFDAICGTGFF-SYI--AHTDEFCLASSG-GVNCYVFAPICQIDSQNQQKRTSKKLVKLSKN--------------------	155
Cbgrl-14	VQSAAEADT-GLFFDAICGTGFF-SYI--AHTDEFCLASSG-GVNCYVFAPICQGGDEQRKK-------KLSKN--------------------	151
Cegrl-10	INLAAEGKF-GGNVDVICSRGHF-SYI--FTSNLYCEATRG-LTTCIAFRQSDKSRRRR-----------------------------------	127
Cbgrl-10	INVAAEGKF-GGNVDVICSRGHF-SYI--FTSNLYCEVSKG-LTTCIAFRQSDKVRRRK-----------------------------------	123
Cegrl-11	IHEAARRDF-EGTWSIICAPCAF-SYL--AHAQDYCIHSRH-GITCLLYRDG--	101
Cbgrl-11	IHAAARRDF-EGTWSVICAPCAF-SYL--AHAQEYCIHSRH-GITCLLYRDG--	101
Cegrl-3	IQKAANKEF-GGHFNVICSPCEF-SFV--VASQKYCDGFKD-DVACFAFLQPPTKLKLDDE---------------------------------	143
Cbgrl-3	IQKAANKEF-GGHFNVICSPCEF-SFV--VASQKYCDGFKD-DVACFAFLQPPTKLKLDDE---------------------------------	143
Cegrl-8	ISERATRDF-GANFDVICARGHF-SYY--VEAASYCEVTMN-DVTCLAYKPGPQTDDGSK--NDFIDIKDELIKNKNTREKDEAESKKNR----	151
Cbgrl-8	ISERAARDF-GANFDVICARGHF-SYY--VEAESYCEVTLN-DITCLAFKPTSENSESKQETTDFANIREQL--NGKLREKGEDEAKLNR----	145
Cegrl-16	IEGDASAKF-GGRFNAIVSDAEF-AYVN-WYGKRNCQLRYE-NRHSLTWED---	135
Cbgrl-16	IEGDASAKF-GGRFNAIVSDAEF-AYVN-WYGKRNCQLRYE-NRHSLTWED---	134
Bmgrl-16	IESKASKRF-GGRFNSIGSDSEF-AYVN-WYGKRNCQLQFN-GRHSLTWED---	137
Cegrl-24	MDTMQNVFPLARSMGCICTDRNF-QFPD-FTNHRYCSVRVS-NFRCHAIVF---	79
Cbgrl-24	MDTMQRVFPLARSMGCICTDHEF-QFPN-FTNHRYCSVRVS-NLKCHSIVF---	79
Bmgrl-7	IQLAAEAQF-DGHFNVICSKDDFSFLTN---TELFCQATKG-DISCYAYRLYL---	136
Bmgrl-14	IYNVCETEM-EIPCNVICGTGFYSYLAR---ATNFCLVSMM-DISCYAFLPACNFNLNLNQKQWIRRHRTKV----------------------	156
Bmgrl-17	IHKAFQESIKENDLDIICSDAGFTYIVS---TTEYCEAQKE-KVICFVYKKT--	137
Bmgrl-2	VTEAAEEYAYQGIKFDVICAEGDFSYTIH---AKKYCEVTKK-DITCFAFR---	134
Bmgrl-x1	IQKVSETQL-GGHFNVLCSNSDLSYSVL--TTSSFCQYQKD-NIICYAFKMP--	136
Bmgrl-x3	IMKKVTKLL--GEYNIFCSTGDLTYSAY---TVDFCQVNKA-GIVCYAFKNL--	120
Bmgrl-x2	IQKAVTEKYPSNRYSIICSTGDLTYTAR---TNDFCLVQQN-NISCYVFRPL--	136
Mi_Msp3	IYSELRAKL-GGNYIINCAHAPSFAYSG-DSVIDYCVDGHQ-AITCAVFKIQ--	138
Bmgrl-x4	IYSELKAKQ-KGDYVVLCSQSSLSFTSD---STNYCVGGNT-NHLCYVFEL---	129
Bmgrl-x5	IQRAAEQNF-ATQFNVICSKGDFSYVTH---AIEYCEVSNR-GTTCYAFRIS--	132
Cegrd-3	LSTFVQQHF-KTAYEIVMAPKDFVLNTH-YNGTSLCKFQSN-SYTLAIYETPAKYDINSSREKYFNKFALNDKLRLPSVSKHLKQFSGLAN---	142
Cegrd-17p	LSSLVQKHF-QVAYEIVMAPNGFVLNTN-YNGTRLCKFQTN-SYTLAIYETPAKYDINSAR-KYFNKFALHDKLRLLPVSKHLRQLSRLAHTSH	144
Cbgrd-4	LSSFIQRHF-KVAYEVVMAPKEFVLNTN-YNGTKLCKFQSN-SYTLAVYETPEYYDINGPGEAYFYNFAANDKLNIPSVSEHLKSFGGLANSAS	145
Cegrd-4	LSTYVQRHY-GVAYEIVLAPKGFILNSN-YNGSSVCKFETN-SYTMAVYETPEHYDVNGPGEAYYYHFAANDKLRIPSVSSHLKKFSGLVNTVT	145
Cegrd-14	LGSRVQKAF-GSAHEIMMGPSAPTLKTN-FNGTI-CQHAGDDGFNYVVYPSPGKYDINNVAVEEYFEKFAEFAALGKGANIADLPKDPRAV---	134
Cbgrd-14	LGSRVQKAF-GSAHEVMMAPSAPTLKTN-FNGTI-CRQAADDGNHYVVYPSPGQYDLNNLAQEEYFEKFAEFAALGKNANIADLPKDPRSI---	137
Cegrd-13	LGSRVQKAF-GSAHEITMGPSEATLKTN-FNGTI-CRHASTDGFHYIVYPTPGQYNINNAAVEEYFEKFAEFAALGKSANIADLPKDPRNV---	137
Cegrd-5	IQKGAEGSL-GGKFETVVALDDFAYKSH-FKEGKSCKIEKN-GQYALAWQP---	83
Cbgrd-5	IQKGAEGSL-GGKFETVVALDDFAYKSH-FKEGKSCKIEKN-GQYALAWQP---	83
Cegrd-10	IQKGAEGSL-GGKFETVVAHDDFAYKSH-FQEGKSCKVEKD-GQYALAWQP---	83
Bmgrd-5	IQDDAWWRN--AKFETVVAYDDFAYKTL-FKAGKACKVSRN-GMQAIN--	103
Cegrd-8	VQAQAEKKF-GTTFESVAAHSDFVAKIN-FAGDLNCKIEID-GKFILAYATPIAEQEVNIVDASSFFSGAADKDLDGVNGTKPTYIVYGPIK--	186
Cbgrd-8	VQAVAEKKF-GTTFESVAAHKDFVAKIN-FAGDLNCKIEID-GKFIMAYATPMSEQEVNIVDASSFFSGAADPDLEGVNGTKPTYIVYGPIK--	180
Cegrd-15	LQAASEKAF-GTDFEAIAGTGDFASKIH-FYSDFVCKMERE-GRTMLVYATPSR---HN------------------------------YAMPY	111
Cbgrd-15	LADVTEDVF-GTDFEAVAAVGDFASKIH-FYSDYVCKMQRD-GRTILVYATPSR---HNGTGKY------SNDRNGNGGNGNGKNGGNYLVTPY	151
Cegrd-6	LQKHSEKIF-STQFETIVSYQDFSQKIH-FKKDLVCKIEVE-GRFILAYATPED-----VEQEKIIPTVPSQDVQKDSDVLKQEVKSKIRQIER	182
Cbgrd-6	LQKHSEKIF-DTQFETIVSYEDFSQKIH-FKKDLVCKVEIE-GRFILAYATPED-----VEQEKIIPTVPSKEIQKDSFILKEEVKAKIRQIER	180
Cegrd-7	LQDKVESAF-KEDFETVVGLSDFAERIH-FREHYVCKIEVN-GRYMLAWATPDD---IGTRRKRGANSTSD-DIH-----------------EY	132
Cbgrd-7	LQDTVETAF-NEDFETIVGLSDFAERIH-FREHYVCKIEVA-GRYMLAWATPDD---HPARNKRGVNSTTPVEIH-----------------EY	132
Cegrd-9	VQRASERMF-GHPFESIVSHADFAQNIN-FSGDLVCKLEID-GKYMIAYGTPYHADDAVGPQGPDGKPLPVRSLKL------------------	171
Cbgrd-9	LQRATEKMF-GHPFESVVSHADFAQNIN-FSGDLVCKLEID-GKYMIVYGTPYHADDAVGPQGPDGKPLPVRSLKL------------------	168
Cegrd-12	LQHETQQMF-NHSMEAIVASGEVQNLSN-YRGDLYCKKRSRDGKIVVIYGSAVPYSLDTGVTRPMNDDELRTQMYPAKYDEIGVHDGHEENIWF	170
Cbgrd-12	LQDEAQGLF-NHSMETVVASGHMENRAQ-YRGDLYCKKRSMDGKLVVLYGSAVPYALENGVTRPMNEDELRMANYPAKYDEIGTYDG-TENIWT	173
Cegrd-16	LRRDSRQVF-AKNLEAIVSTSNFGTSIP---SDFSCKVELGPNRFVAQVFVPELGLDAKTTTRRHRIPYHELSREDLSDAADLVVSRNGIIVSK	182
Cbgrd-16	LRRDSRQVF-AKNFEAIVSSSNFGTSIP---SDFSCKVELGPSRFVAQVFVPELGFDAKPTISRHRIPYHELSREDLSDSTDLVVSRNGIIVSQ	185
Cegrd-1R2	LQQKVQQRF-QKSYEIIVSQSDFVISTY-TAGDNFCKFDNK-GFYILAYVSPKQ---YDIDEKEDEMKLAATSNKDPL---------------	149
Cbgrd-1R2	LQQKVQQRF-KKSYEVIVSRSDFVVSSY-NGGDTFCKFENK-GFYILAYSTPKQ---YDIDEKEDEMELAATSN-------------------	144
Cegrd-2R2	LQQSVQTRF-TKSYEIVVSRSDFVATTY-TAGEKFCKFKTK-NFVILAYVTPRQ---YDIDKKDDE--------------------------	135
Cegrd-11R2	LQHSVQHRF-KSSYEVIVSRSDFMIST--RSGEKICKFQSK-GFYILAYATPKQ---YDIEKVEEERKLAD----------------------	118
Cegrd-1R3	VQWSVEEIL-QHSAEVIVSLDDFAYATY-NNNSYTCKYRVD-KYHILAYTTPN----HDLDN-YDEMA-----TS-------------------	116
Cbgrd-1R3	VQWNVEEVL-QHSAEVIVSLDDFAYATY-NNNSYICKYRVD-KYYMLAYTTPN----HDLDD-YDEMALTKIETE-------------------	131
Cegrd-2R3	IQWSVEEVF-QHSAEVIVSLDDFAYATN-EKNEESCKYRVD-KYYVYVYTTPVPYPIHQ-PT-Y---------ND-------------------	122
Cegrd-11R3	VQWNVEEVF-QHSAEVIVALDDFVYASH-YNDSFTCKYRVD-RYHILAYLTPD----HNLDKRISEVEIPNQITP------------------	132
Cegrd-1R4	MTRAVQKRF-GTTFESVVAEADFSWGTNKFNGRT-CKIDSQ-GYNALTYQSSSKPPPPSDFIDIPNDPTLGGPT-------------------	120
Cbgrd-1R4	MTRAVQKRF-GTTFESIVAEADFSWGTNKFNGRT-CKIDND-GYTALTYQSSSKPPPPSDFLDIPGDPTLGG----------------------	118
Cegrd-2R4	ITRAVQRTF-NTTFETIVAEADFSWSTNKFTGRM-CKIDHE-GYNALTYQSSLPRAIDFIDFPNDVTLDGPTFVENGWFGNGN----------	131
Cegrd-11R4	ITRTMERRF-DTTFDSIAAEADFAWKTQKFNGRV-SKLYLG-GYSALSFQSSPDPPPASDFVDVANDPT------------------------	115
Cegrd-1R1	IQRRSQLFY-HMSFETIVSRENFAISTH-YHGTHSCRVHDN-NLYYLVYETPVQ---YDPFNMKTEDYLAS----------------------	124
Cbgrd-1R1	IQRRAQLHY-HISFESIISKSNFAISTH-YHGTHSCRVHDN-NHYYLVYETPIQ---YDPFNMRTEDYLSSIDSADPLGSTKPP----------	138
Cegrd-2R1	VQRRAQMEF-HSSFETIVSKTNFAISSH-YHGPHTCRVHKK-GHYLLVYETPVQ---YDPYNLPIENYLASID--------------------	122
Cegrd-11R1	IQRKAQLEF-HASFEAIICKSNFAISTH-YHGSQACKVHVE-NQYFLIYETPVQ---YDPFNMHMENYLSS----------------------	126
ruler	00.......110.......120.......130.......140.......150.......160.......170.......180.......190..	
