# Supplementary material for: Evolution of hedgehog and hedgehog-related genes, their origin from Hog proteins in ancestral eukaryotes and discovery of a novel Hint motif
Source: BMC Genomics. 2008 Mar 11;9:127. doi: 10.1186/1471-2164-9-127 (PMC2362128; doi:10.1186/1471-2164-9-127)
Supplement: Additional file 9 — Sequences used in the analysis. List of sequences, accession numbers, notes, predicted signal peptide cleavage sites and protein sequences used in this analysis. [file 1471-2164-9-127-S9.html]

| species | gene | other gene name | type domain | Notes | Accession Nr | cDNA list | Accession Nr cDNAs | predicted signal cleavage site | protein sequence |
| --- | --- | --- | --- | --- | --- | --- | --- | --- | --- |
| Ce | wrt-1 | ZK1290.12 | wart hog |  | ZK1290: U21308 | yk141h4, yk423f10, yk494b6 | yk423f10:C46697, | AAS-AS | MVMNPLTATFLAALIGTAASASCGSSGIPFRFEVLPSGQPVLGCGSPTCFGAENGGRDLRHDSSFMAGADGDDGFFRDGDLARVRVRDPDAPAQMANCPREFSSSSCSNPMTWVGGFKASDNGDLSLQCCHYEGLRFAQEVGRPVVHPGEVYSGGEVLRDGRQTGFDAISNVRKITSGDGTVAYEVTVTRMNCLPNPGEESNEVSFDIQRDIGRILDKVGETAASGVQTNHIEADQRLSPSTDVQSDSYVSPTEADPQEPVEQFVQVGEQVVPVTSAGYYYPVASGVPACFTGNSKVMTPAGEKSMADLSVGDMVMTYEYGKMTYTRVASWLHRLPDTKAAFIKLTTEQGAIIDMTPQHFIYKANCVTEEMELVYAEDMTIGDCLMVKENEKLVMTTISEKSTFYETGVYAPMTETGDLIVDDVYASCHNVVKANTLSHTFLNFATSVQQKMRSVLGSLEETGHLPATSEFFLNIIDVLLPHKY |
| Cb | wrt-1 | CBP09220, CBG12986 | wart hog |  |  |  |  | AAS-AS | MVMNPLTATLLAALIGTAASASCGSSGIPFRFEVLPSGQPVLGCGSPTCFGSENGGKDLRHDSNFMAGPDGDDGFFREGDLARVRVRHSDAPAQMANCPREFSSSTCSNPMTWVGGFKASENGDLSLQCCHYEGLRFAQEVGRPVVHPGEVYSGGEVLRDGRQTGFDAISNVKKITSGDGTVAYELTVTRMNCLPNPAEDTNEVSFDIQRDIGRILEKVGETAASGVQTNQIEADQRLSPSTDVQSDSYKSDSYVAPPESDQQETVEQFVQVGEQVVPVTSAGYYYPVASGVPACFTGDAKVMTPSGEKTMSELRVGDIVQTYEHGKMAYTRVASWLHRLPETKAAFIKLTTDNGPEVSMTPQHFIYKADCVTEVYDLVYAEDVNVGDCVMVKKNNDKLVLTTVVNKSTFYETGVYAPMTETGDLIVNDIYASCHNVIKTNTLSHTFLNLATMMQQKIRSLMGLFEETGHLPVTSEFFLSIIDVLLPHKY |
| Ce | wrt-2 | F52E4.6 | wart only |  | F52E4: U56964 | yk145f9, yk164g4, yk289b8, yk467f9 | yk467f9: C49132 | ALA-SY | MHTPIIFLLALVPVALASYCGQSAIPYTFQVLRSGYPVLGCARPKCFGWTANGTRAGETAQFYRVAGKDDGYLRRSDQFIKSPSKNPNFVPQLAICTDEYKSKTCEEGEWVGGLSPQSDPFADQLEMKCCSYQVLISAEDRGNAIVKQGQLVVGGEVLDGSRLVAFDYISNLSKSVSENGTVVYVASIKRMPCFDEEQAVENKKRENTIVEAAAVVEQPATTPTASSVVTHQPSVTVPQQGAPLNIQPYAPQQQQVQQPAQVAYPGVVNPATNYGQFSQQAYGVAQNGQYSGYPQYAQQQPQPQYYQDPFAAMLQQQHQAFQQQVYANQQQQQQQMQQQQQQQQAQPQQQQQPAQPQQQLGFAPLPQFPQPMMFQAPQVVAQPGVAAAAGVPQMPIGALPQMPVQMTSTLPPMTLPKLEDLPKLQIPSVEDVEQVIPPVQRAILTSVAKFFGVL |
| Cb | wrt-2 | CBP03528, CBG14131 | wart only |  |  |  |  | ALA-SY | MHTPIFYLLALLPVALASYCGQSAIPYTFQVLRSGFPVLGCARPKCFGWTANGTRAGETAQFYRVAGKDDGYLRRSDQFIKNPSKNPNFVPQLAICTDEYKSNTCEEGEWVGGLSPQSDPFTDKLEMKCCSYQVLINSQDRGNAVVKQGQLVVGGEVLDDGKLVAFDYISNLSKTVSKNGTVVYVATIKRMPCFDEEQTETKKQENTIVEAAAAVEPPATSTTATQQPAATVPQVQGTQTLNIQPYAPQQPQALPKNSVPTATYGQFSQQAYGVAPNGQYSGYPQYAQPQAQPQQQQQQQPQPQYYQDPFAAMIQQQQAALQQQQALANQQFQQQQQQQQQQMAFAPLPPFPQPMMFQQPQIVQVAAPGTAGAAGATSPQVVATGVLPVQMTSTMAPMTLPKLEDLPKLQIPSVEDVEQVIPPVQRAILTSVAKFFGVL |
| Ce | wrt-3 | F38E11.7 | wart only |  | F38E11: Z68342 | yk348a9 |  | SLA-DY | MLYHVEMFTIILLFGFSLADYCGSDQVPYGMEVHHSGVVRLMCSKPNCYDKNYSDCPERAESRHGCQKSNQWVGGFEKNIEGDLYTMCCEFEGLEKYAKVRYSDVRIRRGEFFEGEEKENDDGDVVKFDVIKDIRMHKDDEGQAYYNLTVLSFNCESIPDVKPAWYQKSQWPYFQFAKN |
| Cb | wrt-3 | CBP05141, CBG21671 | wart only |  |  |  |  | CRS-DY | MWWQAVTIILVAFAIICRSDYCGSDQIAYGMEVHHSGVIRLLCSKPNCFDKNYSDCPERAESPQGCKKSNDWVGGFDKNIEGDLSVMCCEYEGLEKYAKIRYTDVRIRRGEFFEGEEKENADGDVIKFDVIKDIRMHRDSDGHAYYNLTVLSFDCESIPDVKPAWFQKSQWPYFQYKK |
| Ce | wrt-4 | ZK678.5 | wart hog |  | Y7A5: AL021576, ZK678: Z79605 | yk105d8, cm02c8, yk175d1, yk296a11 | yk105d8: D75507; cm02d8: M75796; yk296a11: C62844 | TYG-SE | MRFSLLALVLLSSSYKFTYGSECGDSTIPYSLEVLSSGQPILGCARPTCFGWHSNGHQLPTNAKFFRIDQQSDGFLRDDPLAIHTFDAADPRVYAQQQASCEQEFQSLSCNPEDQWVGGIAPVMNASTTKIVAYKCCTYAPLRASIDRGVATVSGGQIVVGGEIFADNKPYAFDYISNVEKKIDSEGEIFYEVNIKRFSCLDLQKVDRSVPEILNSENTIRHVNGHRFVVHQAPTVDVETPVETGQLVVPQGVQNGQEVIIEEIVAQEGFVQETNPQPPPPPGQQGGFVQPQGFQPQGGFQPQGFQPQGFQPQAFQPQVVQNPVPAAPAGYAPMGFAPSGLQLYYCFPGDAMVNVYNGGFKRMDELAVGDWVQALDKNGSQVTFIPVQYWLHRDPKQVADFVEFTLDNGETFSLTEKHLVFVTQCSVPYSEDENINANPVPAERVNIGDCFYIAHRKKSQMYQRVKVLDINIVQKTGIYSPMTSRGHLLVDRIHASCHSETDNYSLQNTFFTNVLRWKSQIRNYFWTVEDSTNEDNIGYGLNGVMAVLDIVIPSKLM |
| Cb | wrt-4 | CBP15980, CBG07767 | wart hog |  |  |  |  | AFG-SE | MRSLIWIVLLSISYEAAFGSECGESTIPYSLEVLPSGQPILGCARPTCFGWHPNGHQLPTTAKFFRINQQSDGFLRDDPLAIHTFDAADPRVYSQQHATCEHEFQSFSCNQEDQWVGGISPVMNATNTQVIAYQCCTYPPLRASTDRGLATVSGGQIVVGGEVTENNKQYAFDYISNVEKKLSAEGEVFYEVNIRRFSCLDLQKADRSVQEILNAENIIRQVNNQKAVAHQAPIAAVDTPIEAGELVVPEGVVNGQAVIIEEIVAQEGFIEEVTTPVPPPPFQPQGFQQPPPQPFQPQPIPQGFQQPVQPQVFQPVPVQQPVPVPAQPAGYFPYFSAPAGLELYYCFPADATVHVYDQGTKRMDELEVGDWVQAFEKNGETVTHVPVQYWIHRDQTQKATFIEFSLDNGEKFSLTEKHMVYVSQCENNNYVEGINSNAVPAEKVNVGDCFYVAHRTNSKLYQHVKVLDINKVKKTGIYAPMTSVGHLLVNRIHTSCHSETDNHTLQDTFFANALHFKNLLMKFFGTADSTKEENLGYGIHSLLDVVDLVLPAKFV |
| Ce | wrt-5 | W03D2.5 | wart only |  | W03D2: AF000298 |  |  | TLA-DY | MCSMWLMASWLMAFVAGSTLADYCGDHKVPFGMEVHKNGNVNILCSRPSCHEKKYAECPERATSTTCSTNSSWVGGVTQHSDGSLRLMCCEYDLLPTYSTIQYEKLTIRTGEYFEGDKQMEGDVVTAFDLIGNIEQVKEPDGKYSYNLLIYRYHCGNIPDTPPAWYMKKQWPYWEK |
| Cb | wrt-5 | CBP11846, CBG21493 | wart only |  |  |  |  | ILG-DY | MSNLHKLLYLLLPTVILGDYCGEHKVPFGMEVHKNGNVNILCSRPNCHEKKYAECPERAMSSACTTNSSWVGGVTQHVDGSLKLMCCEYDLLPIYSTVQYEKLQIRPGEYFEGDEQMDGDTVTAFDLIGDIQQVRDAASGNFTYNLLIYRYHCGKIPDSPPAWYMKKQWPYWTPVA |
| Cr | wrt-5 |  | wart |  |  |  |  | TRA-DY | MRRLFLPTVTFILLLILQTTVTRADYCGEHKVPFGMEVHKNGNVNILCSRPNCHEKKYAECPERSTSTSCSTNSSWVGGVTQHSDGGLRLMCCEYDLLPVYSTIQYQKLQIRRGEYFEGDEQMDGDTVISFDLIGDIEQIKEPDGNFSYNLLIYRYHCGNIPDTPPAWYMKKQWPYWDKEQ |
| Ce | wrt-6 | ZK377.1 | wart hog |  | ZK377: U88183 |  |  | VLA-DS | MTLLNLFYCFCLLFGAVLADSIHDGGSCGTNSIPYKMEVDSEGKPVISCEAPSCLGVSSSAARRPRVLDVSCDPFKEIVCVKDLQWTSGLVEINNGTHRTLKTECCSYEGMSDAKTIKSIFLGPGQSFVGGMVEKDGEQSGFDLIKEIRKTVNADNQVQYIVGVYRMPCEATSDSSEEALPLLSRNRRKLRDRVGKYDDYEEDRNYRSERRRPFAMRRRALLQRLEDMYDDYDYEFRVVRRPFRKSRLPYNENALWPLQYSSPQRSRTFADNTYNKETGLLTTMQDGTAALSNYGDSSVESGPLPPPPSSNYIDSQNVAPASPVVQSPAYPQTPAEMPLPPQSGSYSGSYSGYPTADASQYNAYPAMQQPAYQPAYQPAYQPAYQPAYQPAYQPAYSARGYSPNLNGLFGGTGMQCFSGDMEVETEDGIKMIKDLKIGDKVLSMDEAFVTYSPVIMFLHKRDEEIAEFNLIETANGHSIKLTDNHLIYVSDCRTRSDLKLVAAKEVKMDDCIHVTTDSNVVIKKKVSKISKVIETGIYSPLTSTGDIIVNRVLASCHSNLALKSLQQTFFSLYKRTSSVFHNLMFFKSSTEEGDLPVGVETLTSVMDLFIPQSFV |
| Cb | wrt-6 | CBP09579, CBG14222 | wart hog |  |  |  |  | AIA-DS | MRLLLNLCLPFCILFASAIADSYHDGGSCGTNSIPYKMEVDSDGKPVISCEAPSCLGLTSSLKSRPRELSVSCDPFKEVVCVDELQWTSGLVEINNGTHRTLKTECCSYEEMATAKNVKSIFLGPGQSYVGGLVEKDGEESGFDLIKEIRKTVNADNQVQYIVGVYRMVCNARSDSSEELPVLSRNRRKLRERVGKYDDYEEDRHYRRRRPFAMRRRALLQRLEDYYDDYEYDFRPMRRPFRKSRLPYNENALWPLQYSAPQRSRVFAENTYNEKTGLLETMQDGTAALSNYGDSSVESGPLPPPPSSSYIDSQNIAPASPSVQSPAYPQNSQMPQPPPSDSYAGSYQQQNSYTSYNGYPTADNSQYNGYPAMQQPAYQPAYQPAYQPAYQPAYSPSSYSGYSPNLNGYFTKMQCFSGDMEVETEDGVKLIKDLKIGDKVLSMDEAFVTYSPIIMFLHKRDDEKAEFNLIETSNGHSIKLTDNHLIYVSDCNARSDLKLVAAKEVKMDDCIHVTTENNAVIKKKVSKISKVVDTGIYSPLTSTGDIIVNRVLASCHSNLALKSLQQTFFSLYKRTSGVFNSFALFKTSQDDGSLPVGVETLTSVMDLFIPQSFV |
| Ce | wrt-7 | ZK1037.10 | wart hog |  | ZK1037: Z81142 | - |  | SFG-SS | MNISKCVLVVALLSLCCKLSFGSSCGETTIPFSFEILPTGQPVLGCARPTCFGWDPKGYHLPTDARFVRIDRKRDGFLRDDPIYTYPFTPDGSKMYLQQNSTCEPAFQSAMCDSKIQWVGGVEPVQDVNSTHDIAYQCCTYPPLRESTDRGMTLVAAGQIVIGGEVFKNGSQYAFDYISNIAKNIDEYGKIFYEVNVRRLACLDPHNADRSVDEIWDSENTIRKVNGKKAMAHQVPNVAVGNAVPQYNVYPAVAQQPAPYSYLPAAPQCQQYYCFPNDAVVNVYEKAVKRMDELEIGDWVEALDENGEDITFLPVKYWLHRDPEQEAEFLEFSLDNGETFTLTEKHLVYTTECRQNSSELKISWESISAGKVNAGDCFYLAQSEALTKYRLVEILDIKRVKKTGIYAPMTSQGHLLVNKIHTSCHSEVDHHILQNSFFKHVLKWKNKITKYFWSYETERNIGQSLNSLIAIFNLVVPSNMY |
| Ce | wrt-8 | C29F3.2 | wart hog |  | C29F3: Z81043; | cm20f10 | cm20f10: M89293 | VFG-SR | MNYLLLVSGLLSVWQPVFGSRCGESTIPFSLEILPSGHPVLGCARPTCFGWHPKGYQLPTTAKFSRLNRKLDGFLRDDSLFTYPFETDSSKIYKVQNSTCEPGFQSSKCDSKDQWVGGIEPETDAFQDVAYQCCTYAPLRESTDRNIATVSAGEIVIGGEVYQNESQYAFDYISNIEKSMDENGEVYYEVNIRRFACLDPHNADRRIDEVWSSENTIRKVNGQKPIAQQAPNVAVNAPIEAGTFDGEVVDGQTVVIEEIIAQQGFIVENETTVAPFAGPFQAQGFQPRFGAPQGFQPAFQQPPPQQFFPQNFQPVVQQPVQFPAQPVGYAPYAPAGWQLHYCFPADAEVNVYEKGVKRMDELEVGDWVQALHGKETTYSPVKYWLHRDPEQEAEFVEFLLENGESFTLTEKHLVFATDCQQNVKNLDDLNPTSTGKINIGECFFMAQPENASKFQKVQILDIQRVRKTGIYAPMTSLGHLLVNQIHTSCHSEIDHHLLQNSFFKHVLKLKNRISKYFWNEESNTEGNIGTSLNFLIEIFELIVPSKMISY |
| Ce | wrt-9 | H02F09.1,B0344.2 | wart only |  | H02F09: AF077538, B0344: AF067209 | - |  | GAS-YC or SYC-GS | MRHRAFSFQIALVLALLQPFGASYCGSNGVPYSLEILSDGSPVLGCAQPTCMAEPQEDEEDSVFIANTAGQEDGFFREGDRQKKSYTQSYKPKAECPGEFSDFACTKKNQWVGGIDFIDHPRQPLVLQCCTFEGLRFSQDVGVTTISAGEAVTGGEVVRDNRQISFDVIANVRKLVDPDDPKRTYFEVTVRRMNCLPDPPEFEVAYDDDVEPEIQRVLGNATNSAMNMGHENHPHMAQEKKVSKNRPYKRKPKTSSTNNVVSPFVEHHEGEKKSREPFIGSFGATIFNPTAENNKEQETPRAPPRRVHQPYTRRILTPKPRIVHTTTVMPTTHAPPVQVQVEPVTQPTPPPNPFVFAPLPPFPQFGLPQPNLFQFPAPPAPPPFGVPQAPQLAPLPQHQHQFGFFQPPPQPQPQIFGGYGLNNGGGYQQPSYGLQGVQTVQQNQLPEMDVYTRSLLTNPNSPFALQLPTFGAAPPAPQFFAPQQQIQDQQKNLQRVNIQAPQREQSNQPAQLMLQPQPDPNQAPNHDTLGALYRPPPFAALGTTLYNFQSHNG |
| Cb | wrt-9 | CBP18687, CBG16423 | wart only |  |  |  |  | IET-SY | MRHGAFILSSFLLLLQLYVIETSYCGENGVPYSLEILSDGSPVLGCAQPTCMSEPLEDNEDSIFIANAAGQEDGYFREGDRQRRSYSQSYKPKAECPGHFSEFACTKKNQWVGGIDFIDHPRQPLVLQCCTFEGLRFSQDVGVTTISSGEAVTGGEVVRDGRQISFDVIANARKLVDPDDPKRTYYEVTVRRMNCLPDPPEFEVAYDDDVESEIRRVLGNATNSAMNIGHDNHPHIAREKKVPINRPYKRKPKTSSTHNVVSPFVDGHDGEKKPRSEPFIGSFGATIFDPTAEHNAQEESAKSATPPPKREHVPYTRRILTPKPRIIPTTTQTPTTIAPPVQVQVEPVSPMPAAPLNPFAFAPLPPFPQFGLPQPNLFQFPAAPPAPAPLPPAPGPTISGVLQAPQLTPLQPYNHQFAFFQPQPPIGQYGLNGFHQPAPVYGLHGVQNVPSDQFNNLDVYTKSLLTNPQSPFALQMPTFGAAPPAPQFFPQQQPTIEQNASLQRIDIQAPQREKIAQTAAQFSLQPQAANQIPKKDTLGALYRPPPFAALGTTLYNFQAHNGK |
| Ce | wrt-10 | ZK1290.8 | wart only |  | ZK1290: U21308 | yk474b9, yk326b11, yk247g3, yk332f9, yk426a6, yk199d10, yk106h11, yk502c8, yk818g05, yk811a05, yk819d04, yk821g11, yk661h3, OSTR115H5, yk632h4 |  | VLA-KD | MLLVSVISCLLISVLAKDAVTPRVGSQCTKNQVVRKLTVYEDGALEAECGPVPCGEVGKRCIDDQTSCRAETDVFSGMRWAPNGESILLRCCTMHAKNKIYVGTDVVAAGSFYEGGEVAEKDLYGDKGGAEYDFVANARTEQGGVRVWVYRMICAKGEKPVDFDPITTTSAPRVIKTTPAPTTTPTVEEEAEEAEQLEEDQPNDNEAEIVESNDEEEVVEEETEEEEEVTTTPAPKPFNPLRYRPPHFPRQSTGIRRA |
| Cb | wrt-10 | CBP09221, CBG12987 | wart only |  |  |  |  | VLA-KD | MLLISIVSCLFITVLAKDANVRMGSQCSKNQVVRKLTVYEDGALEAECGPVPCGEVGRRCIDDQTSCRAETDVFSGMRWAPNGESILLRCCTMQAKNKIYVGTDVVSAGSFYEGGEVAEKDLYGDKGGAEYDFVANARTEQGGVRVWVYRMICAKGEKPVDFEPITTTAVPKVVKTTTPEPTTPVVEEEEDENEQLEGEEQTNENVAEVVESNDGEEEVEEEDEEEEVTTTPEPKAFNPLRYRPPHFPRQSTGVRRS |
| Ce | grd-1 | R08B4.1 | ground hog 4rp |  | R08B4: Z68008 | yk42d3, CEESI75R=wEST01483=wEST01484 | yk42d3.5: D37242, yk42d3.3: D34193, CEESI75: T00762,T00763 | SEA-NK | MNLLIFLLCYFLGSPHYSEANKIKLKSSGRSADINHDSPCIWNKTQSWVHPYQSITVECCDEELSKLIHKTILDAGNNAKLGNLAKFIQRRSQLFYHMSFETIVSRENFAISTHYHGTHSCRVHDNNLYYLVYETPVQYDPFNMKTEDYLASIDSADPLGSTKPANLRGDFPDVREDSTAGEDLSVQPSIGNILQYPNKFAWPDPDEEVSKKIMLAEQDAIRSINELMESVTEISVMNSGAVVNSTDSLNITEIELPNPTPIPRNFANLREKDRLPENTHCDKERKDGNRCCDGRLASTMRDAMRQMATSPDFGQGKEGIIAAELQQKVQQRFQKSYEIIVSQSDFVISTYTAGDNFCKFDNKGFYILAYVSPKQYDIDEKEDEMKLAATSNKDPLGANTTMFENEAPWHVALKLDTYGDRAGYPVGSHCTQARTGSKCCSLILFNAMKSGYDSHVATSNFDPYDIRNISKAVQWSVEEILQHSAEVIVSLDDFAYATYNNNSYTCKYRVDKYHILAYTTPNHDLDNYDEMATSIIDSEPEAQIVYPAVDRTANQIPFQNTPSPYWTTPVPIPTQVYMPMQYTQQPMQPMYNQQPFFNQQPMFNQQPMYLQPAFNQAPLPYQYPAQPSQFNPFLASFRRFKRQIGRTPYPIHQNVYNDIGSAKPFNCPADLSGLSGMACCDGGLQFEANKVIDQAKQEPDFDKHNTRNLAKLMTRAVQKRFGTTFESVVAEADFSWGTNKFNGRTCKIDSQGYNALTYQSSSKPPPPSDFIDIPNDPTLGGPTGSSGGGGGGGNGGSGGGGGGGGGGSGGSGGGGSNSNSGGGGGNGGGGNGGGGNGNGGGAGDGNGGAGAGNGNGAGAGNGNGAGAGNGNGAGAGNGNGAGAGNGNGAGAGDASAAAAAAQAQAAAAAQAQAAAAAAAQQAAAAAAAANAQQAAAAAAAAANPLSALVAATGACFSLDTWVTTPTGKKRMDQIDIGDYVLTADLEKTYFTPITLWIHREPEKVQEFLTIMTEYGKTLRITSRHFMYRNKCGKSYPQYIKMLPHDGEAIFASDLEVGDCVVVLYKGKYRQQKIETITRSVRTGIYSPLTNNGRIIVNDMLASCYSEIQQNTLQTTFFWAYDKLRSVLVEFFGDLYNNKIELPTGTTLSRDIISLIVPIQK |
| Cb | grd-1 | CBP10540, CBG17433 | ground hog 4rp |  |  |  |  | VDA-EE | MNLPIFLLCYFWGSPLFVDAEEELKYKTFGTSTDTKHQSPCIWNQTQSWIHPYQSITVECCDEELGDLIRKTINDAGRNAKLGNLAKFIQRRAQLHYHISFESIISKSNFAISTHYHGTHSCRVHDNNHYYLVYETPIQYDPFNMRTEDYLSSIDSADPLGSTKPPNLRGDFPDIREDSTAGEDLSIQPPIDSILQYPNKFAWPDPDEEISKKIMKAEEEAIQSMNELMESVTEISIMNSMVNATDGLNITEIELPNPSPIPRNFANLREKDRLPENTHCDKEQKDGNKCCDGRLASTMRDAMRHMATSPDFGKGKEGIIASELQQKVQQRFKKSYEVIVSRSDFVVSSYNGGDTFCKFENKGFYILAYSTPKQYDIDEKEDEMELAATSNKEPLGSNETMFENEAPWHIPLQLERSGERAGYPVGSHCTEARTGSKCCSLILFNAMKTGYDNHVATSNFDAYDIRNISKAVQWNVEEVLQHSAEVIVSLDDFAYATYNNNSYICKYRVDKYYMLAYTTPNHDLDDYDEMALTKIETEPEAQLVFPSVDRSTNQVPFQNTPAPYWTTPRPIPTQVFQPIQYTQQPMYHQQQQQPVYNQQQQFFNQQPMYHLQPSFNQAPLPYHYPGQASQFNPFLANSFHRSKRQIGASPYPIHQNVYNDIGSAKPFNCPAGLSGLSGMACCDGGLQFEANKVIDQAKQAPDFDKHNTRNLAKLMTRAVQKRFGTTFESIVAEADFSWGTNKFNGRTCKIDNDGYTALTYQSSSKPPPPSDFLDIPGDPTLGGPTGSSGGGGGGGAGGNGGGGGGGSGGSGGAASADASSGGGGNGGGGNGGGGNGGGGNGGAAGNGNGAAGGNGNGAAAGNGNGGGAGNGNGAGAGNGNGAGAGDASAAAAAAHAQAAAAAQAQAAASAAAQQAAAAAAANAAQQAAAAAAAANSNDAFSALAAAAGGACFSLDTWVTTPSGKKRMDQIDIGDYVLTAGLDETYFTPITLWIHREPERVQEFLTIMTEYGKTLRMTARHFMYRNKCGNSYQKYIKILPHDAEAIFAEDLRVGDCVVVMYRGKFHQQKIESIIKNVRTGIYSPLTNNGRIIVNDMLSSCYSEVQQNTLQTTFFWAYDKLRNKLTEFFGDLYNKKIELPTGTTLSKEIMSLVLPIRK |
| Ce | grd-2 | F46B3.5 | ground hog 4rp |  | F46B3: Z81540; Y113G7: AL031113; Y44A6:Z98863 | yk87f2, yk317c5 |  | VDA-VC | MKFPTFLLFYFTGFLGGYQVDAVCIWNRTQSWTVPYQSILVECCDKPLKQLILDTIHGTNFESLANGDFAKIVQRRAQMEFHSSFETIVSKTNFAISSHYHGPHTCRVHKKGHYLLVYETPVQYDPYNLPIENYLASIDSSDPLGSTKPFGLRGDFPDISDDPTAGDDLSIQPPSDQILQFPNKFAWPDPDDGTHEPSNKIKRADFDEEVDDLMEISVMDSNSNSTHLEEDLKKEFASKFNTDLDRPGPQPVPRNITNWRKKERYPKGTHCESGKRDGNKCCDARLAATMRDAMRQMATSPEFGPGKEGIVAAELQQSVQTRFKKSYEIIVSRSDFVATTYTAGEKFCKFKTKNFVILAYVTPRQYDIDKKDDERILADVSNRDPLGSSETKFPDEEPFHKELQLEESGDRAGYPVGSHCSNDTRTGSKCCNLRLFNAMKDGYDRHKASPNFDRYNIRNISKAIQWSVEEVFQHSAEVFVSLDDFAYATNEKNEESCKYRVDKYYVYVYTTPVPYPIHQPTYNDIGSAKPFHCPPDLSGLDGTACCNSGLQYEINRILDEARYSDSYHKHDTRYLAKMITRAVQRRFNTTFETIVAEADFSWSTNKFTGRMCKIDHEGYNALTYQSSLRPPRAIDFIDFPNDVTLDGPTFVENGWFGNGNGALSAQAQVVVQAQIAAQAVAPPQPPVFVQQFVAPQFVQFVAPQPQFVAVAPQPQFVAVAPVPPPVAPAVAFAGGGGGGGGCFSSDTLVTTPSGKKRMDEIDVGDYVLTANRVKTHFTPVTLWIHRESEKLEEFLTITTERGSTLQLTPLHFMYRTKCNESSEFLKILPENHEAILASYLEIGDCVILTENTKFRQEKINQTTRGLKTGIYSPLTKNGRIIVNDMLASCYSEVQANVLQTTYFWVFNRLRQKVLNLFGILHMNEIELPTGTAVYKELLSLVIPMGK |
| Ce | grd-3 | W05E7.1 | ground only |  | W05E7:AF100672 | yk66e9 |  | VRA-QS | MKFLLLLGAVIAVRAQSDTCPSLKDRQLAKPGQLFCCDSTIKTVVETGMQTLDLFGAGGPQTLGPIVQALSTFVQQHFKTAYEIVMAPKDFVLNTHYNGTSLCKFQSNSYTLAIYETPAKYDINSSREKYFNKFALNDKLRLPSVSKHLKQFSGLAN |
| Ce | grd-4 | T01B10.1 | ground only |  | T01B10: U97551 | - |  | VQG-QD | MKLILLLIGFAAVAVQGQDDNCPSMKSRKHAKSGDLYCCDSTIKTVIKRGIRTLSYFGDDGPQTLGPIVQGLSTYVQRHYGVAYEIVLAPKGFILNSNYNGSSVCKFETNSYTMAVYETPEHYDVNGPGEAYYYHFAANDKLRIPSVSSHLKKFSGLVNTVTERDVLRN |
| Cb | grd-4 | CBP05429, CBG22788 | ground only |  |  |  |  | IQG-QG | MKFLLVLIGCIFVAIQGQGDTCPSWKDRQHAKPGKLFCCDSTIKTVVETGMKTLDLFGAGGPQTLGPIVQSLSSFIQRHFKVAYEVVMAPKEFVLNTNYNGTKLCKFQSNSYTLAVYETPEYYDINGPGEAYFYNFAANDKLNIPSVSEHLKSFGGLANSASGGLTGSASSLAGSLGG |
| Ce | grd-5 | F41E6.2 | ground only |  | F41E6: AF016448 | yk256h6 | C40963, C30673 | CFA-QD | MRSLIVLAALAVTVCFAQDNCYINDSGFTCCNKELESAMKGAMGGDDLLGSADSIQKGAEGSLGGKFETVVALDDFAYKSHFKEGKSCKIEKNGQYALAWQP |
| Cb | grd-5 | CBP00356, CBG01473 | ground only |  |  |  |  | AFA-QD | MRSLIVLAALAVTVAFAQDNCYINDSGFTCCNKELEASMKGAMGGGDLLGSADAIQKGAEGSLGGKFETVVALDDFAYKSHFKEGKSCKIEKNGQYALAWQP |
| Ce | grd-6 | T18H9.1 | ground only |  | T18H9: U41746, F07C3: U50308 | yk68a8 |  | VYG-QQ | MPSIRYRLVALVVFISSVYGQQEAAVVQTPPVQPTPNLIRFGSGEPVRAIRPNQFQIQLPDIYDPLRDERPKRNNHAGYIRHPTFQVPQQQVQQFRPAGNVYIAQPNPRVVSQYPFRSAPVVRPYVIPQQQFQRQGQSQYIQQQYRPQQQQYSQYPRFDLRPQPVRPVRPVYIASTPASRLSYTERPQTSYGDEIEDTNYLSGRLTPGPIAPVTQGYSERPPATVAPYIERPVPARPTPYIERPVPARPAPYIERPEPARPAPYIERPVPARPAPYIEPTPARPAPYIEPSTAKPQPRPQPPRTRPYVAPSTTTQRYIEPTTQRPTTRRATTKRITTTTTAAPTTPRLTTARATTPLATTSRPTTPSPTTPRATTPLATTPLATTRAPLPPSPPPRTSKRPVTQAPTTPRATTTRRPTTTTPRPTPRRTRRPKTTTAAPTTTTEEVTQGYEEEAVELPKYDETFVGQYYYGRRGEGGNNTFPLPSCFYNPSGYVCCNLMLNELMSTSFEEVKVATNLCNVHKFATKLQKHSEKIFSTQFETIVSYQDFSQKIHFKKDLVCKIEVEGRFILAYATPEDVEQEKIIPTVPSQDVQKDSDVLKQEVKSKIRQIEREL |
| Cb | grd-6 | CBP11104, CBG19232 | ground only |  |  |  |  | IYG-QQ | MSPIRYALVALLTFTASIYGQQDFSTQVQPNNAQPEIIRFGSGEPVRAIKPNQFQIELPNIYDPLREDRPKRNNNHAGYIRHPTFQVPPQQPIQQFRPAGNVYIAQPNPAVVRQYPFRPAPVFRQYVIPQQQQQFQPQFQHQGQPQYVSQQKFRPQQPQQYIQYPRFDLRPQPVRPVRPVYIQSTPAPSRPFYTERPQAAYGDEIEDSNYLNSRITPGPVAPKYSERPPAPQGYSERPPVPQTYSERPPVTQGYSERPYSERPATPQPYIEKPVPARPAPYIEPTPVRPAPYVEPTTARPQPRPQPPRTRPYVVPTTRRPAPPRPAPTAAAYIEPTTTTPRPTTRRATVSRLSDPPTKRITTTTAAPTTTTPAPTTPAPTTTPRTTPRTTPRTTPRTTTPRPTTRAPLPPSPPPRTTRRTTTTQATTTPRPTTTPRPTTTTPRPTPPRTTRRRTTTTPRPTTTTAAPTTTTEVTQGYEEAVETPQYDETFVGQYYYGRRGEGGNNTFPLPSCFYNPSGYVCCNLMLNELMTTSFEEVRVATNLCNVHRFAIKLQKHSEKIFDTQFETIVSYEDFSQKIHFKKDLVCKVEIEGRFILAYATPEDVEQEKIIPTVPSKEIQKDSFILKEEVKAKIRQIEREL |
| Ce | grd-7 | F46H5.6 | ground only |  | F46H5: U41543 | - |  | ATA-AE | MMPSFVFIFLLVSSQLATAAEEYKSQTTYPESAPAATTPQPPPPPPPPKPAPYVEQSAQPQQTAPPPPPAPYPQQAVPAPAPPPAPYPQHAVPAPAPPLASYPQNAVPVPAPPPAPYPQHAVPAPAPPPAPYPQHAVPAPAPYQQQPPPPPPPPHYPPPPPHYPPPPPAPHSAYIDHSAPRPAYIEHSAPPPQPQYPQQQPPQYPQQRQQHSYNAGPRTYHEEPQPYPAKFEYVDYPAPTTRRPYPYPSFEVLPHHEEYEPRSTRRPTTTEEPTTTSRKPRPGWKGDTEYNYPERNLPLKGCFYNNHGYACCNLKLQNKMEELADELLNNGTFHRCNVQKLANDLQDKVESAFKEDFETVVGLSDFAERIHFREHYVCKIEVNGRYMLAWATPDDIGTRRKRGANSTSDDIHEYNF |
| Cb | grd-7 | CBP17047, CBG11008 | ground only |  |  |  |  | VLA-AE | MVPKSVFILLLTGVQLVLAAEEYKSQTTYPESAPPAATTPAPPPPPPPPKPYVEHSAPPPPPAPAPSLAPYPQQAPPPPPPTAAPYPQQAPTQAPYPQHAVPAPAPYHEQPPPPPPPAHYPPPPHQYPPPPHPAPHPAYIDHSAPRPAYPEQAPPPQPYPQQQHTYNGGPRTYHEQPPPAYPSKFQYVDYPAPTTRRPYPYPSFEVLPHHEEYQPKPTRRPTTTEKSTTTHKPRPGWGDTEYNYPERQLPLKGCFYNNHGYACCNLRLQNKMEELADQLLNNGTFHRCNVQKLANDLQDTVETAFNEDFETIVGLSDFAERIHFREHYVCKIEVAGRYMLAWATPDDHPARNKRGVNSTTPVEIHEYNF |
| Ce | grd-8 | C37C3.4 | ground only |  | C37C3: U64857 | - |  | FPA-QR or 22 and 23 | MKSLRIIFCLTLLIASFPAQRSRRKQKFVRLPSGFTFPADAASNFQRDAYIPATFAPPSEKILQAPPRYLTGEHNPAYAHNTDVQGMNYAEYKQAMAPQPHPVDAYSPPPPAPMVPPVTVVEPPAMPYEMTTIASVGPLTTPASVGLKKGNIGGIAQNLNDRYTSLTPEAQRAQKGHTYTALGGGQFYQSLLGGKGGPGGFSPLSFFLNGGLGGTGGGGNNGFFVPVPVVIPPPPPPPPGPNCFTNPSGFLCCNVTLEKTMEDAYLAAKADGASLCNVQKMATAVQAQAEKKFGTTFESVAAHSDFVAKINFAGDLNCKIEIDGKFILAYATPIAEQEVNIVDASSFFSGAADKDLDGVNGTKPTYIVYGPIK |
| Cb | grd-8 | CBP11554, CBG20565 | ground only |  |  |  |  | SIA-QR | MFLLKVIVLLSLLNSSIAQRSRRKQKFVRLPSGFTFPADAASNFQRDSYIPATFAPPNEKILQAPPRYVTGENYLSSGSPSPTSEPHGMDYTEYKKAMQAYASTPVVTPHPPESPSIYQASPPPAATAPTIPPVTVVESPGSEIATTIAPGPATTAATMGLKKENIGGIAQNLNNRFSSLSSEAKQSQRGHTYTALGGGQFYQSLLGGKGSFSPLSFFLNGGLGGGHNNGFFVPVPVVIPPPPPPPPGPKCFTNPSGFLCCNVTLEKTMEEAYIAARDGGASSCNVQKMASAVQAVAEKKFGTTFESVAAHKDFVAKINFAGDLNCKIEIDGKFIMAYATPMSEQEVNIVDASSFFSGAADPDLEGVNGTKPTYIVYGPIK |
| Ce | grd-9 | C04E6.6 | ground only |  | C04E6: U97012 | - |  | AEG-NC | MILPLLLSLFIFLPDVAEGNCHDHRYGVPSKSCKRVKDPTFQIRGDIRIVPMWEFILMKEPSEKRVKRSTSFRNGTLRSVHSGGNHGLPVDSLPTSNKNLNQANSFIKTPVLHQLLIQPIDSSAPASPVQRHIIQSQGKPTIGQLPIHVNKSKINTNHKTKRQGVYSALAQPPPAAKWGNNRPSYQNPTPSYQQGYPQQFPAPQRQQQYQPQPQTTNYNQAPQRPLYIPPPPPKLQVYPPQQLAENPYQTRNGVNNYRSRNSYGTNNRKHGRNGKKGKSSKMCKMCRKLSEEDESEEKEMACDLCSKSRRKGKKKGKSLRDGMVGGRRKSSKSSEDSDDHDDSMEGEGGGTENFTNDDENGADDYEDDGESTTIKPNAVSQPTILDFVERSKQQNKLMRIPVYRGKKLEAESNGTSGAYEDGDDNGEHNREEEEESSQYSSNIRDMEQPTKYSSKPNVKYSYPPKDTLPLQTCFHNPSGYVCCNLELNNVVESTYKEVRELPNFNPCNLQLIANKVQRASERMFGHPFESIVSHADFAQNINFSGDLVCKLEIDGKYMIAYGTPYHADDAVGPQGPDGKPLPVRSLKL |
| Cb | grd-9 | CBP16567, CBG09297 | ground only |  |  |  |  | VES-NC | MILSLLLSLFIFLPDLVESNCHDNRYGVPNNSCKRIKDPKFQIRGQIKIVPMWEFILMNEPTGKRVKRASTTHQNRTIHAAHSGGKTFLQPLPLPQRWIPNASHNPPPRHHTLIQPVDSNAPRLPPPQRHVVERQVVPILNPAQIYNANPYSNQPILAQNSKQNYSAYPPPAPPPRNPLPNLYHQNLQQNATGQPAQYQQQSVNTNYNQPPQRRPPQLQVSPPNQAIETNYYTDRNVGKYQSPKNSGRNRNNRRKGKKKKGRANSKMCRLCREMSEEEDSEEKEVACDLCSKSSNRGRGRKKGRKQKTRTKDSSEEDDSDRDEDSPEGEGAQIDDITEDGDGDYYDDEDRTTQKPTTVSQPTIIDFAKRAEQNKLMRIPVYRGKKLENKDAYRTGSGEHGDGDGHREEEEKSEYSSNIREGEKQTKYSSKPNVKYSYPPKDTLPLQTCFHNPSGYVCCNLELNNVVESTYKEVKELPNFNPCNLQLIANKLQRATEKMFGHPFESVVSHADFAQNINFSGDLVCKLEIDGKYMIVYGTPYHADDAVGPQGPDGKPLPVRSLKL |
| Ce | grd-10 | F09D12.1 | ground only |  | F09D12: AF038610, Y55H10: | - |  | CFA-QD | MRSLVVLAALAVTVCFAQDNCYINEGGFTCCNKELESVMKSSLGGSDLVGSAGDIQKGAEGSLGGKFETVVAHDDFAYKSHFQEGKSCKVEKDGQYALAWQP |
| Ce | grd-11 | K02E2.2 | ground hog 4rp |  | K02E2: Z81560; Y113G7: AL031113; Y102G3:AL020985 | - |  | VEA-GS | MIFLLFTLSILSHVEAGSFKNIGLSTDNKHQSPCIWNQTHAWSHPYQSLIVECCDESLKDLLQNFIRQGKAGSLGDLAKVIQRKAQLEFHASFEAIICKSNFAISTHYHGSQACKVHVENQYFLIYETPVQYDPFNMHMENYLSSIDSSDPLGSTKPQGLRGGFKDHRDDVTAGENLSVQPDADNILQYPNRWSQQDAKEEIFTTEASIENAFVLGTNMSHLPDIAEIDLVGPVPVPKNFTNLRIRDRLPENTHCEKERRDGNKCCSGPLASTMRDAMSQMASSPDFGPGGEGIIANRIQHSVQHRFKSSYEVIVSRSDFMISTRSGEKICKFQSKGFYILAYATPKQYDIEKVEEERKLADISILDPLGSMESNFPHEAPQNVQLLLESSGDRVGFPVGSHCLEEVRTGSKCCSIDMFNAMNAAYNTHMASSYFNAYDIRNISKVVQWNVEEVFQHSAEVIVALDDFVYASHYNDSFTCKYRVDRYHILAYLTPDHNLDKRISEVEIPNQITPSPYWITPVPMTVHQQPMFNLQQNLLPYQLPTHFSSPFTYAFGRMKRQIGTSPLPWLIKKLSFFKSGPRVGDLTPTGLCRRRRRSPRCAIWRELVCVYPIHLSIFHDIGYAIPIFCPSTLTGLFGSIICDGGVAYDMIRIVEETRQQPGFDKHNSRAMAKMITRTMERRFDTTFDSIAAEADFAWKTQKFNGRVSKLYLGGYSALSFQSSPDPPPASDFVDVANDPTVDGPVYTDREQEDDDNDDDDGGDDMSSTSTTTTTTIPPPFFALPVPPPPPVPPPPPPPFLTCFSRDTWVTTPSGKKRMDEIEIGDYVLTADLKTALFSAITLWIHREPETVQEFLEIKTDNGKTLQLTAGHFIYATECRYLPSKNSSLLNSTPERYRHLIDTLPDDSETKLASQLKIGECLLIHNGDQFRMQKIDSISKTVSTGIYSPLTENGRILVNDVLASCYSEVQQNVLQTTFFWAFDRLRNLIVQYFGDLYLDEIELPTGTSLYKEVLTLVIPIRK |
| Ce | grd-12 | F02D8.2 | ground only |  | F02D8: Z78411 | - |  | ADC-WF AGG-CQ | MLHRPKLTVILSVLLTFRLADCWFLSMLGGGAGGCQNQCPPAYSGYYQSRNYNPPQQRFNYGLPPPTPPANSYATAPANYAAPSNAYPFAPQYSIPMNSYAMPKYAVAPQYAMVPYPTPPAYVRPPPVYVTPPPVYITPPPTTTTTIPPPKCFQNTQGYKCCNRQLDQFLEQKVGEMLKPEWQRCNLQRFATQLQHETQQMFNHSMEAIVASGEVQNLSNYRGDLYCKKRSRDGKIVVIYGSAVPYSLDTGVTRPMNDDELRTQMYPAKYDEIGVHDGHEENIWF |
| Cb | grd-12 | CBP17234, CBG11599 | ground only |  |  |  |  | ADG-WF | MSTLLLFLLLTCRLADGWFLGMMGGGCQNRCPTYSGYYQNRSPNPYAQQPAQYALPQTPPPNQYATAPANYGQSPQAYPVAPQHAIPMNSYAGGQYAVAPSYPTPPQYPVAYPTPPAYVRPPPPTYVRPPPVYVTIPPTTTTTTTLPPPKCFLNNQGFKCCNRLLDQYLDQVVTNMQRPSWQRCNLQRFATQLQDEAQGLFNHSMETVVASGHMENRAQYRGDLYCKKRSMDGKLVVLYGSAVPYALENGVTRPMNEDELRMANYPAKYDEIGTYDGTENIWT |
| Ce | grd-13 | W05E7.3 | ground only |  | W05E7:AF100672 | - |  | VLA-AY | MSRFLLLTVLVASVLAAYDDLPKPADPYQPPGTEPKTESTCPDPYKKIITQLRAELGKDVSSIKFTNQLGSRVQKAFGSAHEITMGPSEATLKTNFNGTICRHASTDGFHYIVYPTPGQYNINNAAVEEYFEKFAEFAALGKSANIADLPKDPRNV |
| Ce | grd-14 | T01B10.2 | ground only |  | T01B10: U97551 | - |  | VFA-AY | MVKLLLFVAISSATVFAAYDDLPKPADPYEPPGYEPEACPDKYSKIITQLRSDLGKDVSSIKFTNQLGSRVQKAFGSAHEIMMGPSAPTLKTNFNGTICQHAGDDGFNYVVYPSPGKYDINNVAVEEYFEKFAEFAALGKGANIADLPKDPRAV |
| Cb | grd-14 | CBP05429, CBG22788 & CBP12267, CBG22789 | ground only |  |  |  |  | VFA-AY | MKLLLVLAISAATVFAAYDDLPKPADPYEPPGYEPEPDKPCPDKYVKIINDLRAELGKDVSSIKFTNTLGSRVQKAFGSAHEVMMAPSAPTLKTNFNGTICRQAADDGNHYVVYPSPGQYDLNNLAQEEYFEKFAEFAALGKNANIADLPKDPRSI |
| Ce | grd-15 | Y87G2A.15,Y87G2A.M,Y87G2A.j | ground only |  | Y87G2: AL022597 | - |  | AFS-VP | MHEVTVICLLVFLYSTCQIAFSVPIVVSELNGEKFSAAPNNTPITKVYIFGRPVYIREPFVIPQRDEQIDFPKLIEDASRQRKHRAPYVDEPVLAAKPKYAEHKYPLKQCYTETSGFMCCNPKLEKVMSETALKMKSSKSCNLQKMSSMLQAASEKAFGTDFEAIAGTGDFASKIHFYSDFVCKMEREGRTMLVYATPSRHNYAMPYQL |
| Cb | grd-15 | CBP07824, CBG08040 | ground only |  |  |  |  | VVS-IP | MAATAFAENILVISSIFLISTVYLVVSIPVTHVDSAGEEKYSAAPNNTPISKVYIFGRPIYIREPFVIPQRNVSENIDFSKLFDQSKLRKNRASYTEEQGRQGNQPEQGRYNTNSYQNGYKQPADEYADEYPWPGKYPLRQCYTETSGYMCCNLHLEKVIHNATQKMKESKACNLQKMATMLADVTEDVFGTDFEAVAAVGDFASKIHFYSDYVCKMQRDGRTILVYATPSRHNGTGKYSNDRNGNGGNGNGKNGGNYLVTPYTL |
| Ce | grd-16 | Y69A2AL.1, Y69A2AL.x, Y69A2A\_6076.A, Y69A2A\_1798.a | ground only |  | AC006892 | - |  | GSA-QK | MRCIQLYVFYLISQLFTTGSAQKTFHYGVTGGEQFGRHEPSTRLSRFQSTPVSLNPLEDTLRVQQILKKLEDPGGLEIRRRSTSRRLHGVLTEPFGTPLVISGNSSEKKKKKPLKIKKRNGRRNPKSPEVNEYELQLAKQIEEDQLKFVEDEKNPMKYVVENGLLFRQSRYSPLARVEIPPVSGMSEISGISQRTSHSVGQTMQNPPRKRRRKQSYKLKNSKSSSESMFRTNVFRGNINRQIPAIPLPSPFSVAYGKPSFAIAREEDGKCYTNRFGYRCCDEALEALILKSYEKLRRKSNDLEDNLSKIASTLRRDSRQVFAKNLEAIVSTSNFGTSIPSDFSCKVELGPNRFVAQVFVPELGLDAKTTTRRHRIPYHELSREDLSDAADLVVSRNGIIVSKLL |
| Cb | grd-16 |  | ground only |  |  |  |  | VSS-QK | MQLSGIPVIYYLIYQLFSTVSSQKTYSYGVTGREFIHEPSSRLSRYRSTSVSLNPLEDNLRVQQILKKLEDPGGIEIRRKSTSRKLHGVISEPLGRPLVLSGGLSNDERAVTKKVRKAPVSRRKEKSPEVNQFEMDLARQIEEDQLKFVEDEKNPMKYVVENGLLFKQSRYSPLARVEIPQMVPPKFVLKKRKRKQRKVVKNKRIRTISSGTGEVGSVFRTNVFRGNINRQIPAIPFPSPFSAAYGKPSFAIQREEDGTCYTNRFGYRCCDEALESLILKSYEKMRRRSDSLEENLAKIASTLRRDSRQVFAKNFEAIVSSSNFGTSIPSDFSCKVELGPSRFVAQVFVPELGFDAKPTISRHRIPYHELSREDLSDSTDLVVSRNGIIVSQVL |
| Ce | grd-17p | Y102A5c.34,Y102A5.W | ground only |  | Z99711 | - |  | VRA-QS | MKLLLLWSFSIGVLTAVRAQSDTCPALKDRQLAKPGKLFCCDRIIKTVAETGMKTLDLYGPNGPRTLGPIVQALSSLVQKHFQVAYEIVMAPNGFVLNTNYNGTRLCKFQTNSYTLAIYETPAKYDINSAR\*KYFNKFALHDKLRLLPVSKHLRQLSRLAHTSHRGLLLPIPPPEHSLVGRLFGR |
| Ce | hog-1 | W06B11.4 | hog only |  | W06B11: U39854; Y23B4: | - |  | no | TNGHACFSTDSWMTTPSGKKR MTTPSGKKRMDQVGIGDLVLTGNLTATYYTPIITWMHREPENRYNFYTIMTEYGKMLAVSAKHLIYRNLCDENYAEYVKYLPKGRNVVYAEELKVGDCLVLLYKGKFRQQRVMRISITERKGIYAPITKNGRIIVNDIVASVFSGIKHTRLQSDYYSTIAYAQSWLWIFGETVFHKATIPIGSALASDVLRLVIP |
| Cb | hog-1 |  | hog only |  |  |  |  | no | MFLTETDGHACFSTDSWMTTPTGKKRMDQVAIGDLVLTGNLTATYYTPIISWMHREPDNRYNFHTIMTEYGKMLAVSAKHLIYRNLCDENYAEYVRYLPKGRNVVFAEELKVGDCLVLLYKGKYRQQRVMRISITERKGIFAPITENGRIIVNDIVASVYSGIKHTRLQGQYYSTVAYIQSWLRLFGDSVFHTTAIPVGSSLASDLLRLVVP |
| Ce | qua-1 | T05C12.10, M110 | qua hog |  | M110:Z49968, T05C12:Z66500, Y1E3:AL021388 | cm14h1, cm08e10, yk480e12, yk125e5, yk424b7 | cm14h1:M89067, cm08e10:M89431; yk480e12:C50338; yk125e5:C11824; yk424b7:C46745 | VES-LN | MRRLSAILPILLLSNFWPTVESLNYKCHNDQILVVQSFGNDTIRMHCQRLDLCGYQKLKCDYDELQPQCGGKLNFVSHVNQKGSTAPVEHTCCNLFNPRSHHSIPTHIGNDCFIYELPDGSSNGKKVDPAPADDAPYAVLKNPAEIPEQFDGVTGYRLRLFLLKNKSPPTLLVKGIERRLDGYRVTICRPRCTSYDKVVNDNEGAEDGEWKAISWSSWSSSSWSTWARHAFNKAAAEGGEAAERIRTRMPIGEKTVAGAAGATGAAGSDKSNINIHVESNGNNNNSFEGGRSSSEKSDGQLNREISGSSEAGAGGKGGAGADGAAGSGAGAGAGAGTNGNINITVHTDGKSGGNAVAVANANVTVNGAGGVSTTGTGAQTGNESGLGGSAGTDKAGGKKGGHGDSGDSGNNKNKDNGKGKGKGKNDEEDEEDNGDEDGNGKGGNGGNPKGEWDDGDGDEDDDGTDGGSKESGNNGKGKGKGSGDGDGNRNGNGDGNGRPKGDGNIKINIHSPDDNDLLEKDENGPNGKGGAGNGNGDGDKDNNGKGNGTGDGDGDGNGNGNGLTGDGNGTGDGDNNESGNGNGDGSDKNSGAGAGTKPENREGGDGNGNGTGDGNGDGNDNGNGSKGLGTGSGDGKGEGNKSGTPGKSDGKEDGAGSNGSGNGKEGDGNKSGGSGKGGAGNGKSGDGSGDGKNNGNGGTGDGKDKNGKGSGSGDNDKSGTRAAGKGNAEGNGKGNGNDGKGSGSGDGSGAGGKGDKSDSESGNEADGKDGKKNEGAGGEAAAGSGGANKGGSDGDDDDVDVTDVEVGTKPLTGTKLEELLAKLPNETADGNATGDGNEFGTVQTGAKHNAESSASGIPLVQARSNTVNGGAPVPPAPGSGATGSGTSGSGTSESVTNGSGATESGSTGSGTTGTGTSGTGSSGTGASAARTSSIAGDAPQAAVLADTPGAAGAAGGGRSNCFSADSLVTTVTGQKRMDELQIGDYVLVPSSGNVLKYEKVEMFYHREPKTRTNFVVLYTKSGRKLSLTGRHLLPVAECSQVEQYTMNPDGIDVAMRESKYAEKARKGECVLSIDESGEVIADEIVRVGRMTNVGIYSPMTVEGSLIVDGVLSSCFSHLESHSAHKLIFDFIYYVYNAFGLLNTNHVDLQPIPTFVSFAQYLSKTVLPFS |
| Cb | qua-1 | CBP00142 | qua hog |  |  |  |  | VDG-LN | MRRLCAILPILLLSNFWPTVDGLNYKCHNDQVLVVQSFGNDTIRMHCQRLDLCGYQKLKCDYDELQPQCGGKLNFVAHVNQKGSTAPVEHTCCNLFNPRSHGSIPTHIGNDCFIYELPDGSSNGKKVDPAPADDAPYAVLKNPAEIPEQFDGVTGYRLRLFLLKNKSPPTLLVKGIERRLEGYRVTICRPRCTSYDKVVNDNEGTPESEWKAISWSSWSSSSWSTWARHAFNKAAAESGGSADRIRTRMPIGEKATAAGVPTGAAGSDKNNINIHVESNGNNNNSFGGNGSGEKNGSGENGLNREGSGANGGAGADGAGAGRSGAGAGADGAAGAGAGTHGNINITVHTDGKSGGNAVAVANANVTVNGANGKVDTTGTGASGNGAAGGNGGNGGHGDSGDSRDAKKDKDHGKGKGKGDGDGDSDGGAGNGSGNGSGKDKGDGNGDSDGNGNGSGGSGGSGDKKPKPAGEWDDGDGDDDESGKGTGSNEAGNGKGGDGDGDGSKGAGGSGGKPDGNIKINIHSPDDNDLLEKDENGPGGNGKGDGDKDGGAGGAGGSDKDGGDGNGGSDKDGGAGGDGNGDKDGNGNGNGNGLTDGGDGNGGSKNGPRDNDGNGDGNKETGAGGNGNGDENGNGAGAGGNGNGDGNGGSNRPNGNGAGGDGNGTEAREGGDGNGGSNGGSNGSGDKGAGAGGKGKGDGSVGDKYDKGSGGAGGDKDGKGAGGSGADKDGNGGNGKSNKGDKDGNGKGSDKDKNGKDGAGKAGNGDKDGKGGKKDDSGKDGAGSGKDKDAGKGKGAGDKDKKGGAGAGGKGAGGAGAGGKGAGAGAAAAGKGTGGGDDDDDVDVTDVEVGTKPLTGNTLKELLAKLPNETADKDLGNGDELTNASLNRNQHAEGSTGTAPGTSAAAASAPDTTGGTGGTGTTANAANTGGGTTGSGATSSGTTESGSTGSTGGSGSTGGSGSTGSGSTGTGASTAQRSAVAADTPAAAAADAVAADAAGGGGAGGGGRSNCFSADSLVTTVTGQKRMDELQIGDYVLVPSAGNVLKYERVEMFYHREPKTRTNFVVIFTKSGKKLSLTGRHLLPVAECSQVEKYTMNPDGIDAAMRESKYAEKAKKGECVLSIDASGDVIADEIVRIGRMTSTGIYSPMTVEGSLIVDGVLSSCFSHLESHSAHKLIFDFLYYVYHAFGLLNTNHVELQPIPTFVSFAQYLSKTVLPF |
| Ce | grl-1 | C24G6.7 | grl, ground-like |  | C24G6: AF067936 | - |  | AAA-NN | MLAPILLLLYFSVNSAAANNCGGYMCGSANYYQAYAQQQPATATRYQASYIPQQMPQHTSQPMIQYYYPANTYQIAPQQPTLQKVSPPVISSSVDKESLVTTGKMNYDDFEKELEKLKPASAVTTDEKVDSKLDLNVLNASIPTQSITYIREFPSGYSPEAFITRPLPYRPVPYMPVQLPAACQQYFLPPQRPQPMPQVTPQIIQLPSAYVTAPPVTIRQQPLSILPPPINDCCGKCGAPCKFRSKKNVIALASKIFTAQFVPRRDGEDEEEPKDPKCSSEKLKDLMNKYITRTVALSKRLIQKNAESELGGYFSVFCSIDDFSYVARSEMFCQLQKNDITCYAFKHK |
| Cb | grl-1 | CBP08308, CBG09376,  CbC24G6.7 | grl ground-like |  |  | - |  | SDA-NC | MLAPILLLLYFSVNPSDANCGGYLCGSASYYQQQPQQIPRYQPSYTPVQHPSQQQVLQYYYPANTYQIAPQPMPQKVTPPVISPSLDKETPVSTGKMNYDEFEKELEKLKPASAVTSEDKVDNKLDLALLNASIPTQSITYIREFPAGYSPEAFMTRPLPYRPVPFVPVQLPAACQQYFMPRPMPMQPQVVQLPSAYVTAPPISVRQQPVSILPPPINDCCGKCGAPCKFRSKKNVIALASKIFTAEYVPRRDADNEDEPKDPKCSSEKLKDLMNKYITRTVALSKRLIQKNAESELGGYFSVFCSIDDFSYVARSEMFCQLQKNDITCYAFKHK |
| Ce | grl-2 | T16G1.8, T16G1.H | grl ground-like |  | T16G1: Z81592 | yk27a8 |  | SFQ-FM | MLTALILLQLLIPPSFQFMFGGSGGGGCGCPCPVPPPVPICAPQTICAQQAPCSSSSSYSPSYSASYASVPAPVPSPFYQSGWSNPIPSYASAPALPSYSTAYSMAPPPLILPSSPSYVAPVSAPSYSLTPSISIPGPFPPAPLYVPPAPPMPIVTDGYDKISIVTSIATTPSYLQSGYAPATLAYEKQYDEESSLEGMAPPPPPPPPIDIPKDVKTYDYRTSEVVSHSENYKPAFVPSASYLEDVSEEGQLVEKQGHWSKGSDSQTSSKYGQFESRHNILKRMKTESIPRTNNTCNSIKLANVMMRAIVDDVSVSKRMIQHATKSAFDGAKFDVFCAIGEFSYSIHSRKYCEVTKQEVTCFAFR |
| Cb | grl-2 | CBP02375, CBG09498, CbT16G1.8 | grl ground-like |  |  |  |  | SLQ-FF | MLTALILLQLLIPPSLQFFFGSSGGGCGCPCPVPPPIPICAPQPICAQPAPCSSSPSYPSYSPSYSSYASAPAPVPSPFYQTGWSPAIQPYASAPTIPSYSTSYSAAPPPLVIPSGPSYVAPVSAPSYSLTPSISIPGPLPPSPIYMPALAMPIVTNGYDQISIVTSIATTPSYLQSGYVPAASKGYETKYDEENSVEGMAPPPPPPPIGIPKEPSTYDYRTSEVVTPSDYYKPAIVPSMSYMEDSSEEGQLVEKQGYWMKGHPVSQTSSKYGQFEARHNILKRMKTEAIPRTNNTCNSIKLANVMMRAIVDDVSVSKRMIQHATKLAFDGAKFDVFCAIGEFSYSVHSRKYCEVTKQDVTCFAFR |
| Ce | grl-3 | K03B8.7 | grl ground-like |  | Z74039 | - | - | VLT-AP | MTVKYFVILLLLIVFIIPSVLTAPVRTELCCCGCIEEPCPLVGPSCPAPRAPCNASVETVKCSALQRLFEISQHKLFADEDDEETSTFQPTPSFDHQKTMKLLPTLNAKEVQRMKNEIMEISTNKTRKSEIRFSPPIESDRVNGLHRISHMMKEMEGMLMLMRDEISIQIENSSPVKLLEEAATRTEIDSSLHASPMTRAIRHKRAETENCNDEKLRKLIKENIRRDAKSSKREIQKAANKEFGGHFNVICSPCEFSFVVASQKYCDGFKDDVACFAFLQPPTKLKLDDE |
| Cb | grl-3 | CBP20597, CBG23193 | grl ground-like |  |  |  |  | ILS-AP | MKNDRQNFYILLFLACFAHSILSAPVRTELCCCGCTEEPCPLVGPSCPAPDAPCNKSRETVKCSALQRLFEISQHKILADVGDEEDVTTVRPTLNFEVEHQKTLQLLPTLDAREVQRMKNEILEITTNRTRKAGIRLSPRIESEKVNGLHRISHLMKEMEGILSLMRDEVTTQIENSSPVKLLEEAATRTEVDSSLHATPMTRGMRVKRAETDNCNDEKLRKLIKDNIKKDAKSSKREIQKAANKEFGGHFNVICSPCEFSFVVASQKYCDGFKDDVACFAFLQPPTKLKLDDE |
| Ce | grl-4 | F42C5.7 | grl ground-like |  | F42C5: U40799 | yk304g12, yk437e1 |  | ASS-TF or FG-GC | MLLPISILFSIIPIASSTFGGCCSMGPPPCPPPPPPMCAPPPLPCPPPPICPPQFCPPPPMCPPPPPPPPPPMCPPPPPPMPSYSPCQSYAPAPVFNQYAMQPANDCCCRCGSPCRFMARHRTHGSKLFTTEDPEEDPTCNSKKLRRVMERNMNGDPSISKRAIQKAVEEKMFGKFNVICARGDFSYVAYTETYCQVANDDVTCYAFRPM |
| Cb | grl-4 | CBP21652, CBG05832 | grl ground-like |  |  |  |  | ASS-TF or TFG-GC | MLLPISILLSLVPIASSTFGGCCSMGPPPCPPPPPPMCAPPPLPCPPPPICPPQFCPPPPPCPPPPPPPPPPMCPPPPPPMYSPCQSYAPAPVFNQYAMQPANDCCCRCGSPCRFMARHRTHGSKLFTTEEQEEDPTCNSKKLRRVMENNMNRDPTISKRAIQKAAEEKMFGKFNVICAKGDFSYVAYTETYCQVANDDVTCYAFRPM |
| Ce | grl-5 | Y47D7A.5,Y47D7A.x, Y47D7A\_146.b | grl ground-like |  |  | yk94d4 | D66255 | AQA-FF | MRTAVLLIVGFATAQAFFFGGGSSCGCSAPPACPPPPPPSPCGGGGYARAYSAPTFTAGGAGYQQQPQYQPQQSSFGSSYAAPAPFAAQPAQFAVQSAPPQPIVAPPPQAYAAPHKRHVDVMDIDTGVDQSEGYVARVKREENVFDPKCNSEDLKAIIVANIHESTAVAKRQIQTAAADAIGGRVDVICSKGTFSYIVNTELYCETEKDGTTCFAFKQSS |
| Cb | grl-5 | CBP12513, CBG23612 | grl ground-like |  |  |  |  | SQA-FF | MLRTTLLLAIGVVASQAFFFGGGGGAACGCSAPPACPPPPSPSPCGGGGGGYARAYSAPTFAAGGGSYPQQPLYQPQPSYQPAAAPAAFGSSYAAQPQPFAAAPAPFASQPQSFAAQPQSFAAAPQPIVSPPQGYAAPHKRHIINNMEDTGVDQSEGYVARVKRDEEAVFDPKCNSEDLKAIIIANINESTAVAKRQIQSAAADAIGGRVDVICSKGTFSYIVNTELYCETEKDGTTCFAFKQSS |
| Ce | grl-6 | K10C2.5 | grl ground-like |  | U39852 | - | - | AFA-QY | MSTLSVAVFLISFHFAFAQYPFGRGGCGGCPTPMCQPRMPCAAPMPMPMPMPVCPPPPPCPAQFCPPPPICPPPPPPPMPCPPPPPPMPRPSCPCMMQRPSFYPSYVPQYYQPMMPQPMPTAGGCGGGAVVPSVRIPAQNDCCCGCSSPCKYKSVRRAAFAAKTIDPSCNSSELKNIILDNISEDASESKRNIQKIAEETLGHEVNVICGTGEFSYIAHTDTFCQAFKEDVTCYAFKPLQ |
| Cb | grl-6 | CBP01297, CBG05066 | grl ground-like |  |  |  |  | VYA-QY | MSTSSLAIFLFALSCVYAQYPFGRGGCGGCPTPLCQPRMPCAAPMPMPMPMPVCPPPPPCPAQFCPPPPICPPPPPAPMPCPPPPPPMPRPSCPCMQQRPFYPSYVPQYYPQPMYQQPMPMPMGGGCSGGAVVPAVRIPAQNDCCCGCSSPCKYKSVRRAAFAAKTVDPSCNSSELKNIILDNIIEDASESKRTIQKIAEEQLGHEVNVICGTGEFSYIAHTDTFCQAFKENVTCYAFKPL |
| Ce | grl-7 | T02E9.2 | grl ground-like |  | Z93387 | yk217h6, yk282e7, yk475h3, yk61a4, yk80d11 |  | TSA-FF | MLFRLVSTLLLAQTTSAFFFGGGGGGCGCQQAPACAPHPAPAPCSGGNIVQGYVGAPQQGGYAAAPQYPQQGGYGGAQQGFQAAPAPYQPQGGYQAGPAPVQGYQPQAQVPAGGYQAPVQPAPGPAEVAPAQVQAGGNYQDAPAQVAEVATAQESAPPPSEAAYTGEQEVVASLAREEPNYQNTGTNVIEAAQHASELGNQAAAAAAKVAEVVEEEEEPIKKKDEGKTTHMKTVKAPAAASSTTAAAASTSTEFPVGEEIVADTNEKEVDISELHLTDDPLCNSDDLRKVVIDNIDDQLNSSKRMIQLAAEAQFGGRFDVICANGDFSYVTNTELYCQETKGDISCYTYRQL |
| Cb | grl-7 | CBP05493, CBG23176 | grl ground-like |  |  |  |  | TSA-FF | MLFRLVSTLLLAQTTSAFFFGGGGGGCGCQQAPACAPPPAPAPCSGGNIVQGYVGAPQQGGYAAAPQYQQGGYAGAPQQFQQGPGPAPYQPQGGYQAGPAPVQAPAGGYQEPVPQAPQAPQPQPAEVAPAQVQAGGNYQETPAQTAGVDPAQQQSAVPENEAAYTGEEQEVVASLAREEPNYQNTGTNVVEADQHASELGNQAAAAAAKVAEVVEEDDEPIKKKDEGKTTKVKTVKTPAASPASSTTTAASSSTSTEFPVGEEIVPDTNEKEVDISELHLTDDPLCNSDDLRKVVIDNIDDQLNSSKRMIQLAAEAQFGGRFDVICANGDFSYVTNTELYCQETKGDISCYTYRQL |
| Ce | grl-8 | ZC487.5 | grl ground-like |  | ZC487: U50310 | - |  | ASS-AI | MCHYYLLRGFLLISVASSAIIPLPSPPNPLPEPPTVDASPSLTSEKRTVAMEVTSSAHHLLRPKTESDSEEEQPKPTRNRVRLHSHNSKVSKVSRTREVEVLSEKCNDDKLEQIMQDAMTPSLSTSKMVISERATRDFGANFDVICARGHFSYYVEAASYCEVTMNDVTCLAYKPGPQTDDGSKNDFIDIKDELIKNKNTREKDEAESKKNR |
| Cb | grl-8 | CBP11197, CBG19006 | grl ground-like |  |  |  |  | VLS-AI | MAAEFLFLLSTIPVVLSAIVPLPNPPNPIPEPPTVDTFPSLTSEKQTPIALDITSSADQQENERKVSRNRVRLHSHNSKISKLSRTREVEVLSEKCNDDELQQIMQDAMTPSLSTSKMVISERAARDFGANFDVICARGHFSYYVEAESYCEVTLNDITCLAFKPTSENSESKQETTDFANIREQLNGKLREKGEDEAKLNR |
| Ce | grl-9 | ZC487.4 | grl ground-like |  | ZC487: U50310 | yk409f10 |  | TQA-SP | MTRSWLLVAALLAFTQASPPMCHCPNGQVGMQCPSPINFNCPPQAPCPPQQGCNMFGGLPTLPTLAPQQFQTLAPFTLAPLPGSPPGMAGPPLLPPAVAPPTNGQETLVGINNPVQPPPPVQYQNQGPQYIEAPPPPPSPPPPPPPPPPQLQQQQVQLPLQDATTLRPSTLPSETYVELENPEAVVNEPQPIQAQTEEPATTQKATDAPPPPILQNKIERVFESEPNSGYRKPVRRQAPPLSEKCNDDRLRKIIEQNVDDNPSTSKRKIQKAAAEEIGGLFDVICSAHDFSYLANTQLFCESGNDDVTCFAFLHSLIQ |
| Cb | grl-9 | CBP11196, CBG19005 | grl ground-like |  |  |  |  | VNA-SP | MTISWLLAVVLLSSVNASPPMCHCPNGQVGMQCPAPINFNCPPQAPCPASPPCNMFGGLQPLPTMAPQQFPTFAPFTLSPLPGSPPGASGPALLPPAVSPPTNGQETLVGINNQNQPPPPPVQYNQGPQFVEAPPPPPPPPPPPPPPPPPLQQQQIPVQDATTLRPSTLPSETYVELENPEAIINENAAQVQKNPEEARNNVEKVTDEPPPPVLQNKVERVFESEPNNGYRQPVRRQAPPLNDKCNDDRLRRIIESNVDDNPSTSKRKIQKAATEEIGGLFDVICSAHDFSYLANTQLFCESGNDDVTCFAFLHSLIQ |
| Ce | grl-10 | C26F1.5 | grl ground-like |  | U53148 | yk416b9, yk442a9, yk459g2 |  | VNG-FF | MRHSLTLVVVILSLSLYSVNGFFFGAAGGGGACGCAPRPACPPPPPSGCSGSGVRAVARGAKTMTFDQPIYSPTPQSYAQPPRVYSVPDELDLRAAAFGVPIQPQPQYTIFDILHNLGESNDNPAQQLVVADGSTNTNYFPKENDEDLTVSDMTGDGGLYRSKNIRRAPVEPTVHVHRDSSVETTTISSEISGMETDETLDKNKCSSSVLRKLMIENISDSSSESKRNINLAAEGKFGGNVDVICSRGHFSYIFTSNLYCEATRGLTTCIAFRQSDKSRRRR |
| Cb | grl-10 | CBP04840, CBG20585 | grl ground-like |  |  |  |  | VNG-FF | MRHSVTLFILLTSSSVNGFFFGAAGGGGACGCSQPPACPPPPPPCGAHRAVARGAKTRSFDQPITAGYQPNYATNPGPQVYTVPDELDLRAAAFGVPIQPQPQYSIFDVLHNLGESRDSPAQQLVVADGSTNTDYFPKENDEDLTVSEMTGSGGWYRAKEIRRAPAEPTVHVHRDGSVESTTLGSVATEESFDKNKCSSAVLRKLMLEEITDSSAESKRNINVAAEGKFGGNVDVICSRGHFSYIFTSNLYCEVSKGLTTCIAFRQSDKVRRRK |
| Ce | grl-11 | ZK512.9, ZK512.x | grl ground-like |  | Z22177 | - |  | ISG-IQ | MYFSIFSVFIGLQITVEISGIQLIDGSTNVTVFHNDYTDSYSTSLIQNPNIRTKPIPRSRKDDDCSDELLRKVMNEHISDEGMTESKRAIHEAARRDFEGTWSIICAPCAFSYLAHAQDYCIHSRHGITCLLYRDG |
| Cb | grl-11 | CBP07471, CBG06938 | grl ground-like |  |  |  |  | VRG-QK | MALSCYRILLVLIGLRVTIEVRGQKTMIDGNTNLTSSRQYDYIEPYPGSLIQNPNVRTKPVVRPRKDEDCSDEILRKVMNEHISDEGMTESKRAIHAAARRDFEGTWSVICAPCAFSYLAHAQEYCIHSRHGITCLLYRDG |
| Ce | grl-12 | F28A12.2 | grl ground-like |  | U64851 | - |  | ASA-FF | MLSPFLLLFLLFSAPASAFFNSLFGGSNCGCSCTPPPSTCPTTCLPIMTCTPPSPPACCNTCGTCNGKRRRRHLTMLSNATYVADHEKVNRVKRQDDQETTVTSGNCNSVELQRIIESKIDRVTAIAKRRIQEEAEATMGGRFNVICARGDFSYVANTELFCQHSVGDVTCFLFKQLSDVVRRRLM |
| Cb | grl-12 | CBP00353, CBG01466 | grl ground-like |  |  |  |  | ISS-FF | MLTLPFFLFLLLQPISSFFFPSGGGSNCGCTCTPPPSTCPTVTPCAPIMTCSPPPVQACCNTCGTCKGRRKRRHLMLSNATYVASHDPARRVKREDDTEAASDGRCNSEELRKIIENKIDRVTAIAKRRIQEEAESTMGGRFNVICARGDFSYVANTELYCQHSVGDVTCFLFKQLSDVVRRRLM |
| Ce | grl-13 | F32D1.4 | grl ground-like |  | F32D1: AF016427 | - |  | ANS-LF | MVCVLVLILMASLTANSLFFNGGGGCGCRPQQCGCAAASLPVRSYCPPSPCGRQFQSFPSYTQPYSPVSFRPPPPTIIQQQPYYSVPHPIPIQTQQFHNSYQQTPVFTSSYNAPPFASSSSYSNAPNSFQNAPIQVASAKETSADTFDTLIQKSKPETTDMKNDLDVDVNALSNFETSLQAYKSMNRNGIRRAQAAKTEKAGKCSSERLQQIMEEAMSSNVSVSKLKISRGAKKEFGYNFDVICSQFDFSYLISSNIFCRVELDGQICLAYEN |
| Cb | grl-13 | CBP12521, CBG23622 | grl ground-like |  |  |  |  | SQG-LF | MVHILLLSMTTIGISQGLFFGGGGGCGCRPQTCGCAAPLPVARSYCPPTPCGPRPFPVYPSYQPVSFRPPPPTLIQQPYYAQPQQIPVPIQAQFHNSYQQTPVFTSSYNAPFNNNQLPSYINSPTQNAPIQVTSAKETTADSFDTVIQKSRTDTSPAKNDLDVDNALSNFETSLQAYKSMNGIRGIRRAQGAKIEKPEKCSSTRLQQIMEESMSSSVSVSKLKISQGAKKEFGYNFDVVCSQFDFSYLISSNIFCRVELDGQICLAYEN |
| Ce | grl-14 | T03D8.4 | grl ground-like |  | Z92838 | - |  | ISA-KS | MILILITLVFVFQKTQISAKSDFEILKNDSIFDGGTQGNKAFLKTQLLRNLNNFYPSINNGEKLEMLTGRKPEFEDDRERNQNFDGNSVTEPFPTQYPTLIPYARESNPEEELQLATAPTSKIRSEGRITSEQKMDSIRDFRMKLYKAFKNRPKLSRMIRKSNVNDVVEMNDGFPTIMDKNRQIILSRTEPNWQSLNTRKPNQTYGRDQNGNLIPLLGPYPPREIRYAYRTTNRQPVVYVPSASPVNQPSHASNTVPVAVASYLVTANPSSQPIQLHILPPIPSPQPQIIYQQTTPSSQFVAYSTFAPPIEPSNECGNGQCKPESDEDKCNSQRLRDIIFNNIVSGDAEASKRAVQSAAEAETGLFFDAICGTGFFSYIAHTDEFCLASSGGVNCYVFAPICQIDSQNQQKRTSKKLVKLSKN |
| Cb | grl-14 | CBP01428, CBG05569 | grl ground-like |  |  |  |  | VDS-KQ | MKVTRRVTVDGVSTVTLLLALYTLVDSKQSDFGILKNDSIFDGGTQGNKAFLKTRLLENLNNFFPSINNEERLEMLTGSSKSKEEDLDDSNQELTDNSVTAPPPPEFPTLIPSSEPEEEFPTSSVESSKIRSEGRITSETKLDSIRDWRKRLYKAFKNRSKMSRIIRKSPSEEVVEMNDASPTIMDKNRQIILSRTEPNWQSLSSGKHIQTYGRDPNGKLIPLPYPPREIRYAYRTTHRQPVVYVPAAPAPAPPQNTIPVPVASYLVTANPSPQPIQLHILPPIPSPPAPQPQIIYQQTTQAPQFFTFSTFAPTTDNQNDCSNGQCRPESDDEKCNSQRLRTIIFNNIVNGDAEASKRAVQSAAEADTGLFFDAICGTGFFSYIAHTDEFCLASSGGVNCYVFAPICQGGDEQRKKKLSKN |
| Ce | grl-15 | Y75B8A.20, Y75B8A.W | grl ground-like |  | AL033514, Y79H2:AL022287 | - |  | VSC-TF | MQSFLQIFLIFSVISTVSCTFFNGGCGCQPPPICLPPPPPMCFTEIQLPPIRIPIIPLPRIELPQPCCPTCACGGRKKREIDAGVQEEESVSTKDVSCNDDSLLAIMKKEMTTGESSAVKIALVEAAEQELGGRFTVVCSQGAFSFVTSTTSYCLHSQAGLNCYLFKTQ |
| Cb | grl-15 | CBP12825, CBG24285 | grl ground-like |  |  |  |  | VSS-TF | MQNLARILTISAVFGSVSSTFFNGGCGCPPPPLCLPPPPPMCFTEIQLPPIRVPVIPLPRIELPQPCCPTCACGRKKREIDTGVAEDTVSTKDVSCNDDGLLAIMKKEMTTGESSSVKIALVEAAEQELGGRFTVVCSQGAFSFVTSTTSYCLHSQSGLNCYLFRTQ |
| Ce | grl-16 | Y65B4BR.6, Y65B4.x, Y65B4B\_137.B Y65B4B\_137.A | grl ground-like |  | Y65B4: ?; Y50C1: ? | yk434g6, yk206a2, yk376g8, yk409f5 | C47287, C66265 | ALA-CI | MRVLVAVLLAVSPALACIGGAGSAGGCCPPSQPSCGGGAPPCSSSSYATGGGGGSYAAAPALPPPPPPPPAPIGGGAGYAAPFGGPGPVGGASYSQGPQGFGGQVGGGAYQAGPGPIGGGASSHAGSQAGPSYAGASQAVGASSYARSQGPIGGGPSYAGASQPAPVAPQGGYNAGPSAPVEQVQQGGYQSGPVSSIQVTQVSQGGNYQAPPEHQAVQVVEQVAPVATYTQGPVPTPVEQVVETVVEPQQAPEPEQAPEPQQAPEPTVVETVQEEIVPEIVAETEAPVVVAETEAPAVVETEAPVVIEETEAPVVVEETPAPVVEETQAPVAAAKEESTYDDIVEEQPSTATETATEQVDTVEETNEAVEYADEEGDEGNCDDPELRAIVEAALKNEKDNLEAARKIEGDASAKFGGRFNAIVSDAEFAYVNWYGKRNCQLRYENRHSLTWED |
| Cb | grl-16 | CBP01307, CBG05117 | grl ground-like |  |  |  |  | ALA-CI | MRVVLAVLLAVSPALACIGGAGSAGGCCPPSQPSCAPATPPCSSSSYASGGGAYAAAPAALPPPPPPPPSAGYAGAAGPIAGGAYSQGPQGGFGGAPQGGYQSGPIGGGQQGYAGPGPIGGGAPQGPSYAAGPAQGPIGGGQQQGPSYAAGPAQGIQGGGQEGYAGQGAAQGGQGYQAGPAQVAPVEQPAPQQGGYQSGPVNSIQVTQVSHGGLYQAPPEQPAAPQETVTEAATVVVEETQAPIIEETQAPVVEETQAPVVEETPAPAPVEQAPVAAAKEESTYDDIVEEQPATATEAATEQVDTVEETNEAVEYADEEGDEGNCDDPELRAIVENALKNEKDNLEAARKIEGDASAKFGGRFNAIVSDAEFAYVNWYGKRNCQLRYENRHSLTWED |
| Ce | grl-17 | C56A3.1 | grl ground-like |  | Z77655 | - |  | CHA-IF | MMLRLLLLSVSTLTLCHAIFFGMGGGGGGGGGGCCCGCGTPQPASCGCAPACPQAPSCPVCPPPQPCPAPPAAYCPQVQPVYVQSGGGGCGGGGCGGGGGGCGGGGGCGGGGGGGCGGGGGGGCGGGGGGGGGGYASGGSGGFASAPVSLPAPSYGGPPPPAPSFSHAPSGGYSSGGSSGGGYSSGGSSGGGGYAGGAAAAGATAAQVDEASETSAGEPPVDQVFHSPKEPCTQQVKYIMLRSRKVPGGGATELVEEELEQVENPPPPVEATANPLDAQLGEDIAAQQQAATEDDGDDESASSGDFKARAAKTAVTDEKCNSKILQKLVLTNIAANDALASKKAIHDNALQQFPDSSVDVICSTTGFTYLVSTTEHCEAQKDGVICFVYKRPL |
| Cb | grl-17 | CBP02782, CBG11540 | grl ground-like |  |  |  |  | CHA-IF | MMLRLLILSVSGLTLCHAIFFGMGGGGGGGCCCGCGTPQPPSCGCAPACPQAPACPVCPPPQPCPAPPAAYCPQVQPVYVGGGGGGCGGGGGGCGGGGGGGCGGGGGGENCSEGGCVGGSGGGGYASGGSGSSGGGYGRAPVSLPAPSAGYSGPPPPIPSSGSGYSSRYSSGGSSGGSGYSSGGSSGGGYGAAAAGATAAQVDEATESDGEPPADQVFHSPKEPCTQTVKYIMLRSRKVPGTETTELVEEELEQVANPTHVEATANPLDTQLGEEIAAQEQAATGNSEDTEDAESASSGEFKARAAKTAVTDEKCNSKILQKLVLSNIATNDALASKKAIHENALQQFPDSSVDVICSTTGFTYLVSTTEHCEAQKDGVICFVYKRPL |
| Ce | grl-18 | T05C3.4 | grl ground-like |  | AF016428 | - |  | TTA-IF | MAKYLFFYLSLVLYFHETTAIFFPQLGFGSMNCQCQNSCSSPPAQLGCLCPPPMPCYQQPTQNYYTQPPSVIYPSYQNQQPLQPSSSYLANIPPFMQSYPQPPLFTTPPPLPQDNSILIWPIDQKIRKLIYKINYPTPSQTFIPQQTLKTSYSTSFGSLGPPAPSAEKLNLQVPPALKQKEETSDQQVNEKNYVFGAHQPLSDIFYETGASEADNEIDQNYSPIITTTEAVYRPIETTAKRNYETSTHRTSSSSRATKQESRRYETELMRGEGNSDISSSGYGFESASTTSAPILLEFLEQPTKYENRSPNNTELADVTAFTQEVDEFYKSSTSSPAQTFEYGAVRDARDSTGKDETSTPKFPFDRDENVNKWTSKRKIREKEEINSKCNNPILKDLMEMKMTTSPSISKQMIYSAATEMWMGRNVNVICSKHSFSYVVVTSPIFCEHRKKALTCFVFFQP |
| Cb | grl-18 | CBP16517, CBG09384 | grl ground-like |  |  |  |  | SGA-IF | MAKNLFFFLSLVFYLHESGAIFFPQLGFGSMNCQCQSSCNSPPSQLGCLCPPPMPCYQQPAQTFYTPPPPPVIYPSYQNQQQPQPSSSYLANIPPFMQQYAQPSFFSTPPPIQVQQLPPGTSSLNYPSASQMFIAEQSLKPSYSFPGSAGPPASSDSQETLNLQPPAIKQKEETFERPVSEKNYVNTADRPLSDIFYETGASEDDKEIERNHLPILTSTEAVYRNTDTATRTNSESSTYPSSTIAVTTKRYETALKRGEGNSDISQSGYGFEAASTSEPIMLEFLEHPRKYENQSPNNTDITDVPDFTKEVDAFYKSSTSRPIETFEYGAVRDARDSSDKETSTPKFPFDREEEVSPKSKWTSKRKTREKEDSSKCNNSILKDLMELKMTGSPSISKQMIYSAATEMWMGRNVNVICSKHSFSYVVVTSPIFCEHRKKALTCFVFFQP |
| Ce | grl-19 | R02D3.6 | grl ground-like |  | AF038615 | yk150a2 |  | AFA-FF | MFLKLLLFLSICHVAFAFFFPRVTPNDGCGCGCGGGGGGGGCCAPPPPPPVCGGCGCGGRKKREIGHVKGHLAHRDNDQEWNNQCNSLEFSDVIVKHLRTKSLSTSRDFIYKELDTAFPDSMFTVFCLQNSTVSYQADAKRYCMEKTADRSCYVFEF |
| Cb | grl-19 | CBP05381, CBG22548 | grl ground-like |  |  |  |  | ASS-LF | MLKLLILLSLCHVASSLFFPSVSPAGGGCGCGCGGGGGGGCCAPPPPPPVCGGCCGGRKKREIGHVKGHIVHRDEQEWSNQCNSQEFSEVILKHIEMSSLKSSREAIYKELDAAYPDSMFTVFCLKNSTSSYQADAKRYCMEKSKDRMCYVFEF |
| Ce | grl-20 | C23H5.9 | grl ground-like |  | AF067609 | yk144e8, yk363f10, yk442g11, yk122d4, yk364h4, yk262g4, yk365h4, yk124c7, yk189c9, yk303h7, yk86g6, yk445d1, yk445c12 |  | TQA-FL | MRVLVVVSLIVVSTQAFLFPGGGGGGGCGGGCGGGCGGGGGGGCLPTLGCSVISFNVPTLKLPPPPPPPCGGGCGCGGRKKRAASEDSKCTDPELRKIILNGVRTTTSESRDNIVASLKEKYAGVRYLVTCIEGDHDFASSSTDYCADGSQQQTCIVAKATDE |
| Cb | grl-20 | CBP02581, CBG10552 | grl ground-like |  |  |  |  | TQA-FL | MRFVVTLALIIVSTQAFLFPGGGGGGGCGGGCGGGCGGGGGGGCLPTLGCSVISFNVPTLKLPPPPPPPCGGGCGCGRKKRAASEDSKCTDPELRKIILNGVRTTTTESRDNIVASLKEKYAGVRYLVTCIEGENDFASSSSDYCADGSQQQTCIVAKATD |
| Ce | grl-21 | ZC168.5 | grl ground-like |  | Z70312 | yk712h4 |  | SSA-FL | MVHSSVLIVLSLTASSSAFLLEGLGLGLGPQRPQQCCCPPPPPPPPCGGGYEAPPPPPPPSYAGGPSYAGPSYAGSYGPGPYRAKSKRSVDMKASSGDLFCNSVEVRDIIKKGMTSGEKESRETITALLKTEMNREYVVICTKQPFEYLASSDSEFCSVTNDSGITCSSFVF |
| Cb | grl-21 | CBP10608, CBG17648 | grl ground-like |  |  |  |  | TSA-FL | MVRNSVLVILSLTTATSAFLLEGLGLGLAPPRPQQCCCPPPPPPPPCGGGYEAPPPPPAYAPPPPPPPQPYSTGPSYAGSYGAAGPYKRARSKRSMELKASSGDVLCNSVQVRDIIEKGMTSDEKESRETVVALLKAEMEREYVVICSKQHFDYLASSDSDFCSVTNDAGITCSSFVF |
| Ce | grl-22 | W03A5.3 | grl ground-like |  | U39850 | OSTR047B2\_1 |  | TSG-FL | MLRLALVLSVFIVSTSGFLFPMAGGGGGGGGCAPAAPACAPPPPPMCGCAPPPPPPPPPPMCGCGCGRKKRSVDDVEGVINMDSDVECNNEELREVLENNMKSTPSDSLVSVRSNLPTDQYFVTCTHGLTAYSAPAGTKSCAVRKESHFCQIFSLTNNSSNL |
| Cb | grl-22 | CBP07014, CBG05173 | grl ground-like |  |  |  |  | SSA-FL | MLRFTLLSIFIASSSAFLFPMAGGGGGGGXXXPPPPACGCGAPPPPPPPMCCAPPPPPVCAPPLPPPPPPPMCGCGCGRKKRSVEGEPAVEGIINMDSILECNNQDLKGILEKNMKTPSDSLLSVRSSLNSDEYFVMCTHGLTAYSAPAGTKNCAVXKENHFCQVFSLNESH |
| Ce | grl-23 | E02A10.2 | grl ground-like |  | Z81053 | - |  | ATA-FL | MRKSIIILLLGLSVATAFLFPSGGGGGGGGGCGGGCGGGGGCGGGGGCGGGCAPPPPPPACGGGCGGGGGGCGGGGGGCGGGGGCGGGGGGCGGGGGGCGGGGGCGGGCAPPPPPPACGGGCGGGGGGCGGGCGGGGGGGCGGGGGGGCGGGGGGCGGGGGGCGGGGGGCGGGGGGGCGGGGGGGCGGGCGRKKREAINVVGHDDLNKCNNEELRIILNENTKETVAESIKTLKEKVAGQDYIVVCNEKPAPFTAETDDFCSLQKENVHCTILRINHKEVAEKNEEDKKEEEPKKEEEKKEEVEKKEEDEKKDEEPKKEEEKKEEEQKEEVEKKEEEEKKDEEPKKEEEKKEEEEKKEDEVEEKSEKVEEKELEPKKDEEETKKN |
| Cb | grl-23 | CBP12461, CBG23530 | grl ground-like |  |  |  |  | ATA-FL | MRKSIIILLLGLSVATAFLFPSAGGGGGGCGGGCGGGGGCGGGCGPPPPPPCGGGCGGGGGCGGGGGCAPPPPPPCGGGGGGCGGGCGGGGGGCGGGGGCGGGGGCAPPPPPCGGGGGGCGGGGGGCGGGGGGCGGGGGGCGGGGGGCGGGGGGCGGGCGRKKREAVNVIAHDDLNKCNNEELRIILNENTKETVAESIKALKEKVAGQDYIVVCNEKPAPFTAETDDFCQLQKENVHCTILRINHKEIVEEKKEEEKKVEEEPKKEEEKKEEEKKEEGSKEESKEEKKEEESKAEKKEEEPKKDEVEEKSEKAEEKELEPKKEEEETKKN |
| Ce | grl-24 | F11E6.2 | grl ground-like |  |  | yk123d11 |  | ASC-QD | MSSLLLLLVLVMFFTLASCQDDNFEGERCNDVILYDIIKKASKKTDDPVIIRRTSMDTMQNVFPLARSMGCICTDRNFQFPDFTNHRYCSVRVSNFRCHAIVF |
| Cb | grl-24 | CBP05755, CBG00435 | grl ground-like |  |  |  |  | VSG-QE | MCTFHILPAILLFVVLVSGQEEMMDGEKCNDVILYDIIKRASKTTSDPAEIRKTSMDTMQRVFPLARSMGCICTDHEFQFPNFTNHRYCSVRVSNLKCHSIVF |
| Ce | grl-25 | ZK643.8 | grl ground-like |  |  | yk207e6 |  | VSS-FL | MATYLRLVVLFLLTIHVSSFLFPSAGGGGGGGGCGGGCGGGCGGGGGCAPPPAPCGGGCGGGGGGCGGGGGCAPPPAPCGGGCGGGGGGCGGGGGGCGGGGGCGGGGGGCGGGGGGCGGGGGGCGGGGGGCGGGGGGGCGGGSSGGCGGGSSGGCGGGGGGGCGGGGGGGCGGGSSGGGGYAVAPSGGGGCGGGGSSGGGGYAVAPSGGGGCGGGGSSGGGGYASAPSGGGGYATSGGGGSGGYATGGSSGGGYSSGGSSGGGYSTGGGGGYAGGGGGGGGSSGGYAGSSGGGGYSAPAAAPPPPPPPPPPPAPAPVSSGGGYSEQSSGGGGGSSYSGGGEASSSSGGGYSGGGESSSSGGSSYSSGGDSSSSSGGGYSSGGDSSSSSSSSGGYSGGSDSSSSSSSSSGGYSSGGGDAGASSGGESSSAGGYSGSSSSGGEASSGGYSGGSSEPAPAPEAAPASSGGYSGGSEAAPEAAPAAPSGGYSGSEAAPEAAPAAPSGGYSGSEAAPEAAPAAPSGGYSGSEAAPEAAPAAPSGGYSGSEAAPEAAPAAPSGGYSGSEAAPEAAPAAPSGGYSGSESSAPAAPEPAPSSGGYSGGGGDAGSAAGGSNYSGGGETAPAAPPPAPEPAQTYSGAGGESAAAAPAPSGGGYSGSGGAGGAPNGANYDEAQEDVEEYEGGAAGFRNRRFSKGGTADDTPICNSVRLRKLIRQSLTEDPESSMQRLAQRVKSRLVSGEFYVACGEQGLLPLAGENREHCFIKSGKFACYVLRKA |
| Cb | grl-25 | CBP21805, CBG06865 | grl ground-like |  |  |  |  | VSS-FL | MATYLRLVVVLILTVHVSSFLFPSLGGGGGGGCGGGCGGGGGCGGGCGPPPPPPCGGGCGGGGVCGGGGVCAPALPPPPPCGGGGGGCGGGCGGGGGGCGGGGGGCGGGGGACGGGGGGCGGGGGGCGGGGGGCGGGGGGGCGGGGGGCGGGGGGCGGGSSGGCGGGGGGCGGGVKDTSRTFWNLLEVTHININEVMSSGGSSYGGASSSSGGTSYSSGGDTASASSYSSGGESSSSSSSGGYSGASTGGESSSGGYSSQGSTGGESSSSGGYSSGSSTGGESSLSGGYSSGGSESVSTGGESSSSGGYSGGSESTGSESTSSGGYSGGSDSSATSSSSSSGGYSGGGDAGTSTGGESSSSGGYSGSSSSSSGGESSSAGGYSGSSSSSESIPEPAPEAAAPAPEAAPAASYGGSESSAPAESAPVESAPAASSYGSSESAAPAAPSGGDYSSSGSSESAAPAAPEPAPSAGGYSGGGSDAGATSGGGSNYSGGGDASAAAPAPAPAQTYSGGGDAAVAVPAAQQGGYSGAGGAPNGANYDEAQEDVEEYEGGSAGFRNRRFSKGGAADDTPICNSVRLRKLIRQSITEDPESSMQRLAQRVKSRLVSGEFYVACGEQGLSPIEGEHREHCFIKTSTFACYVLRKA |
| Ce | grl-26 | K02D7.6 | grl ground-like |  |  | yk506e1 |  | HG-CAG or CAG-LF | MFSNSAKIVLIFAILFFDLCHGCAGLFGGGGGGGCCCQSACGRKKRSIDEDERREFEFRGIASKNEDLLCNSPEMKAMIQAHMKSNPQASSKSLQSALEDHDSHRYVVVCSENPFHYSIKHDSAYCGARNGSHYCQAFAI |
| Cb | grl-26 | CBP12179, CBG22544 | grl ground-like |  |  |  |  | CNG-CA | MSKIATLCLFLVLLDVCNGCAGLFGGGGGGCCCQSGCGRKKRSVDDEEAPGFQFRGIASKADDMLCNSPDMKALIQAHMKSSPQASSKSLQVALEDHDQHRYVVVCSENRFHYSVKHDSAYCGSRNGTHYCQAFAI |
| Ce | grl-27 | F40C5.3 | grl ground-like |  |  | - |  | TAA-CP | MQSSLLILLAIVSITAACPGLFGMGGGGGCGCGAPPPPPACGCGGRKKRSLPEKPEFFGIAAGDQDVMCNTPELKKIILENTQTTAVDSSKAINTVLESRQLQRFVVVCSENPFVFTIRADTAYCGASKNGHNCHAFAM |
| Cb | grl-27 | CBP05966, CBG01299 | grl ground-like |  |  |  |  | CAA-CP | MQSSILILLCLVSICAACPGLFGMMGGGGGCGCSAPPPPSSCGCGGRKKRSLPEKPTFFGIAAGDDDMMCNNTELKKIILENMQSTSVDSSKAVNGALEAKQLNRFVVVCSENPFVFTVRADTSYCVARKNEHNCHAFAM |
| Ce | grl-28 | T24A6.15 | grl ground-like |  | AF068713 | - |  | TAA-CP | MHSSILLLLAFTSITAACPGLFGMMGGGGGCGCGAPPPPSCGCGGRKKRSLPEKPTFYKISASGEDLVCNTPELKKIIVENMESSAIESSKSVNAVLETRQLNRFVVVCSENPFAFTARADSAYCGAAKNGHNCHAFAM |
| Ce | grl-29 | T24A6.18 | grl ground-like |  | AF068713 | - |  | TTA-CP | MISSLLFLLVFSSITTACPGLFNMLGGAGGCGGGGGCGGGGGCGGGCGYGGGGGCGYGGGYGCGIPPPPPPPPQCGCGRKKRSLPEKPTFFGISASGDDVMCNTPELKKIIARNMQDSAMDSSKTVNAALENKKLQRFTVICSENPFVFSIRADTAYCGVQRNGHNCHVFAM |
| Ce | grl-30p | T24A6.3 | grl ground-like |  | AF068713 | - |  | TAA-CP | MHSSILLLLAFTSITAACPGLFGMMGGGGGCGCGAXPPPSSCGCGGKKKRSLPEKPTFHGIAASDEDVMCNTPELKKIINENMQASAVDSSKAINGALESKELNRFTVVCSENQFVFTIRADTAYCGAKNNGHTCNVFSM |
| Ce | grl-31 | T24A6.19, T24A6.y | grl ground-like |  | AF068713 | - |  | TTA-CP | MHSSILLLLSFASITTACPGLLNIFGGAGGGGCGGCGGGCGGGCGCAPPPPPCCGGGCGGCGRKKRSLPEKPTFVGIADSGDDVMCNTPALKKIILENIQTTASDSSKAINTVLESRQIQRFVVVCSEKPFVFTFRADAAYCGATKNGHNCHAFAM |
| Ce | grl-32p | T24A6.x | grl ground-like |  | AF068713 | - |  |  | NIQTTAVDSSKAINTVLESRQLQRFVVVCSENPFVFTIRADTAYCGASKNGHNCHAFAM |
| Mi | qua-1 |  | qua hog |  |  | rd30d09.y1 | BQ613344 | TIA-IS | FRLITSLFIQLFNILAILNSIPQTIAISYRCEGDQVLVVQSFGNDTIRMHCQRLNVCGDVDVHCHYEKNQPACGGKANFVAHVDQPTPLAPVSHTCCEAIPPFEEKMNEIPSHEGNDCFIYELPDPNAPPPEEGEQNNKKEEEEEENEHFTLLNSIDQLPGHINPEGYHYRMRLFLLRYKSPPALLVKGIRRLREGYRVTICRPRCRRVIIEEDERQEK |
| Ss | qua-1 |  | qua hog |  |  | kq58h02.y1, kq19a09.y1, kq23h04.y1 | BF014893, BG226231, BE579200 | IFT-TQ / IKG-LS | MRWRSMLFNILIITFLTIIFTTQIKGLSYRCENDQILIVQSFGNDTIRMHCQKLQLCGYSNLKCTYDREQPACGGKTNFVSHVNQLTPTGKVLHTCCDMTFKNNKSKHIIEHDGNDCFVYELPDGTTDITPGSEADIIKKNIDVVTNGFTVLKDASQIPEDFGGYTGYRLRLFMLRNKSPPLLIVKAIERTSGGYRVTICRPRCGKFNREGIISQGESISTFDKDISIPKNKIIHSNENHCEKHTTEEGHSKKNENNSNA |
| Mc | qua-1 |  | qua hog |  |  | ri62g01.y1 | CB931749 |  | CGGKANFVSQVDQPSPLAPITHTCCEAMPSLEEKITEISSHEGNDCFIYELPDPNAPPPDSTEKENNNKNDDDENEHFTLLNSIDQLPKHLNPEGYHYRMRLFLLRYKSPPALLVKGIKRLREGYRVTICRPRCRRVVIEEDEPQEKEEDVGLKEWITQKLTGIESQ |
| Ov | qua-1 |  | qua hog |  |  | SWOv3MCAM30H08, SWOv3MCAM21D03 | AW257707, AI444860 | SAT-IT /KHS-AT | MFPRSMIFFLLLSLGMKHSATITYRCDDDQIIVVQNFGNDTIRMHCLKPTLCGFQFLKCHYNHLQSYCGGKTNFVAHLQQSTPISPVIHTCCNLTINEDVQIQAHIGNDCFLYDLPDGSDGTTGEDLEKEDKEGYMLLRNINKLPGQFANFSGYHLRFYLLRNKGMSQNVVKGVERNELGYRITICSIQCRDSVGRRKGMIYENDEKNEEKQVIRDDKHIDLNKKEQIFNDNNLNQVKLTIRNLTDDGQWLFVTWAEWSYKQWSEWSTIHKIELNELDGTKRRNRCGIHETDTEIKTNTEKGQEKSERIKMFMQKLNRVREGFRDCDDDEDYTDELKKNNVEQEEQKLGEDSKELGYDYSKKELAIKSAEKQNFSELDVEENLKSELNSHKINETEEEMDLFVNPVTRREEIENQTKDHFEKMKENTLSDDGNNLKTPTIRATIHCNISITKEVRSGHDDISSIKKTSESEEKDISTIKHRDDVDLMNTSQSYLNEKQLLAEKKKK |
| Cr | qua-1 |  | qua hog |  |  |  |  | VES-LN | MRRLCAILPILLLSSFWHTVESLNYKCHNDQVLVVQSFGNDTIRMHCQRLDLCGYQKLKCDYDELQPQCGGKLNFVAHVNQKGSTAPVEHTCCNLFNPRSHHSIPTHIGNDCFIYELPDGSSNGKKVDPAPAEDTPYAVLKNPAEIPEQFDGVTGYRLRLFLLKNKSPPTLLVKGIERRLEGYRVTICRPRCTSYDKVANDNEGAEESEWKAISWSSWSSSSWSTWARHAFNKAAAESGSADRIRTRMPIGEKAGTGAGGATGAAGSDKNNINIHVESNGNNNNSFGGGSSEKTDSQLNREVSGSSEASNGDGAGSGGGAGAGKAGADGEVGSGAGAGAGTHGNINITVHTDGAGKAGGNAVAVANANVTVNGVNGGVSTAGTGASTNGNGGAGGADGGNGGAGGKGGNGGGHGDSGDDGDKDGKDHGKGKGKGKGKNGGDDGDDDNGDGNNGGDGDGDDNGKGSGKGSSGKGSGKESGKDKGSGGNKKPAGEWDDGDGDDEDGGAGNGGSNESDGDGKGGKGGDGDGKGNIKINIHSPDDNDLLEKDENGPGRKGKGDGDGAGAGGAGKDNGNGNGNGNGKDNGDGNGNGTGNGDNDGNGNGNGVTGDGDGNGNGGSKGPNGGDGNGTGARETGDGDGDGKGPNGSGTGDGGAGGAGGKGKGGGKGDKDGKGKGSGSGDGNGDGKGDGKGKGKGKGDKDGNGKDKNGGAGGAGAGKDNGKGAGKGDGKDKAGAGAGKDGAGAGNGKDAGKGKGAGDKDKNAGGAGGAGAGAGGKKDGGAGGAKAGTGAGVGAGAGGNGANGGKNGKGKGGDDDDDVDVTDVEVGTKPLTGSKLQELLAKLPNETADKAAAKSDEDALTPKTLKRKQNAEAAAPGTTSVSSGTSGTSGTSGASSNAAAADTSGTSGTAGSGTTGTSGTTATGTSGTGTASTGTASTGTGTAATGTGTTGTGSTGTSAVAADTAAADPAAAVQADAAGATGGGGGGRSNCFSADSLVTTVTGQKRMDELQIGDYVLVPSAGNVLKYERVEMFYHREPKTRTNFVMIFTKSGKKLSLTGRHLLPVAECSQVEEYTSTPDGIDAAMRESKYAEKAKKGECVLSIDEFGNVMADEIVRIGRMTNVGIYSPMTVEGSLIVDGVLSSCFSHLESHSAHKLIFDFLYYVYHAFGLLNTNHVELQPIPTFVSFAQYLSKTVLPFS |
| Bm | qua-1 |  | qua hog |  |  | kb60c04.y1 | CB338757 | SAA-IT | MRSVMLFVFLSLLIKHSAAITYRCDDDQIVVVQSFGNDTIRMHCQKPILCGLQFLKCHYNHLQTYCGGKTNFVAHIQQLMPIAPVMHTCCNLTINEDVQIRAHMGNDCFLYDLPDGSNGTTAEELEKKDKEGYALLKDINKIPEQFTDFSGYRLRLYLLRKKEPSQFVIKGVERNEVGYRVTICTIQCQNDNHKVKAVTSDQKHADLNKQEPIINDNNLNQINFALRNLTDDGQWIIATWAEWSYKKWSEWSTERRIEFNDLDRTKGRGHRYRIHHSNSESGTATGKGQQKSEGIKTSIHESSKGHRRIHKKKHSSDCDDDDDDESYGSKEVGKIDVTKEGGKIKERFGNIPDNDSKEKLNQEKRKFPALDIQENMKSELDPHKTNGTGKKEELFTVRFGEIGNQTKDGTKKIEEGTEKNQGDSSGNNANATKATESERKDHSNANKEGGGDDLTGKKITLTQKTDKSAGNQQKEKKHGELKDQLKSGSTQSSKPVNQTRKQELGHDPPAIPVAAGHHVGANPMPAMNCFSADTKVYTQNGEKTMKDVVVGDFVLVPVSKSQMRYERVEMFYHREPETRAKFVVLETESGRKLSLTELHLLPLGDCKEMHESMTDTTDIVDQWLRKSKFAHRARIGDCVFTMTSNHELQVDRIVKVGRQYLKGIYSPMTVEGSIVADGILASCFSQVESHFSQKLVYDFLIFLYRIFGPLMQSLDEPIQHLPTFIDSIHHLGRFAVPFVKY |
| Bm | wrt-4 |  | wart |  |  |  |  | IFA-SY | MLLLTSLSVCLIIIQNAYIFASYCGEDAIPFSLQALQSGQPVLGCARPSCFGWGVKTDKGARFYRIHRKNDGFMRRTDLKKYDKAKTMARESQLAFCEKNYASSSCDENMQWVGGLSPSSNITTQPLLLKCCTFDNLKNSWDRGIADVNPGQIVVGGEVMKDERQYAFDYIANIKKYSKKNGSITYSVTIRRFWCLPYPTKSNLYVEENTLPYIMDRMTRNYETNRQPETNSKQFTSQVIINHQLIIVLLQR |
| Bm | wrt-5-3 |  | wart |  |  |  |  | VLY-KG | MPVMISVIISLLIPIVLYKGEYCGENKIPFGIEVHPNAQPLLHCSRPSCFERRYADCDDRAQRKSCESNDSWVGGFEKGYGNHQPLYVQCCTFEGLADHSSPLYHTIIKPGQYFEGEEQVEEETDTVISFDVITDFKMIRSTNLSIFYEMAVRRLRCYELPPVDRIKRTRRWP |
| Bm | wrt-6wa |  | wa hog wrt wart hog |  | AA842729 | AFCZ9H04 |  | VSG-QQ | MLLPVMLFLMEAVSGQQQSAIDSSCSEYTIPFSFQSDKTGNPTLLCTSPACFDEKRLYELKRDDVIPEIETETRSLLGSKNHIHKAQCHNYYNGYFETLQCFSGDTTVQTPDQIKRIDELQVGDQVLSIEESLISYSPVVMFLHRSDNESAIFIKITLENGEIIKLTD |
| Bm | wrt-6 |  | wart hog |  |  |  |  | VSG-QQ | MLLPVMLFLMEAVSGQQQSAIDSSCSEYTIPFSFQSDKTGNPTLLCTSPACFDEKRLYELKRDDVIPEIETETRSLLGSKNHIHKAQCHNYYQNISCTGEIQWTVGLLLEDDGNNIKAKWKCCNYEGLRHARAMKTVIVKADESYAGGEVYQDGRRVAFDLIKEVYLLFDEQHRPRYELKIMRLACIPKPKSNKIIWVIIVFPVLTDKNRKSNSPTMDVSSEYDTEEYIFTNQQHKKYSRLSTILEDDNFDDLFLPRHRMTHRRMFSRRRRPFYRPVAEDYDYYDYDPVILKRPSNAIEGDYHQFVDSDSTVNPITSAQAEVYNQLSATVEFATLVGLFLQQTVEQLLPTYSSLQYLPQRAEISSEPKTIVSAQRAVPTYSGGNVPYYNGYFETLQCFSGDTTVQTPDQIKRIDELQVGDQVLSIEESLISYSPVVMFLHRSDNESAIFIKITLENGEIIKLTDYHLLYVTSCAVGENLRLIFAKDVQLGHCLHVVKNQSNNLVPVEVSNIQRLTGKGFYAPLTANGDIIVNSILSSCHSNVAVQTLQQSIFNFLRKFRYLISTDQNTDGLLPGIQFLTQISDLFLPYSIV |
| Bm | wrt-10 |  | wart |  | AA114395 | AFCE4A12 | AA114395 |  | CLLKVITKLTVYEDGAIEAECNKLPCGISGTQCKDNQASCRAETDTFSGMKWASNGQSILQRCCIISVPRKLYIGTDLVSLGSYYTGGMVDPKDLYSKEGPEFDFISNVRTEQGGVRIWVYRVVCPKTTGISDRSTATSQIQRSATVQKQVSSNGNTQFGRAVAEGAPTAYRPLNPLQYRPANWRMLQRDSPIHAKSRDLFERLI |
| Bm | grd-5 | grd-A | ground only |  |  | SW3ICA1195 | N44358 | LNA-YR | MCWWGNVLINFVFLFASLNAYREDCFINVGGYVCCDMDMVKVMQNVMEEDDNLLNVAKKIQDDAWWRNAKFETVVAYDDFAYKTLFKAGKACKVSRNGMQAIN |
| Bm | grl-2 |  | grl, ground-like |  |  |  |  |  | MSNQYDMDYLLEALNDLTTISATSMYPLTTTDIEDTLEDSKNNLQRTSFNVLKRQKKTKDAXKRNYPAKRAIFPSAVCNSRRLKKVILQAITDDVSESKRRVTEAAEYAYQGIKFDVICAEGDFSYTIHAKKYCEVTKKDITCFAFR |
| Bm | grl-4 | 1133771 | grl ground-like |  |  |  |  | CNT-FG | MELLMLTIVFMQIWDVCNTFGCCPVAVPCMPYRCKPCIPVPCPPPLPPPTCPPPLPPCPPPPVCPLPPPPVICPTLPPPCPPPPICPPPPPPVICPPLPPPCPPPPICPPPVVCPRPIICPPPPPPPPPPPPCPPSPPPCPPSPPMPICPILAPKLTPTYTIPVINDCCCTCVAPCMYSQMRIHGAKIFSASLVADLEDHSKCNDPVLKSIMEENMTEDATTSKRSIQRAAEEKLFKKFNVICSESDFSYIAYTDTFCQHSNDDITCYAFSPYSGI |
| Bm | grl-7 |  | grl, ground-like |  |  |  |  | YDT-XF | MKQIVILFECILLFPSYDTXFFDCCFMLKFPELCETLCNSHANLFSLQPAISSPTYATPPFPFAPTTTTQIYQINSLSEXISPVYAMQQLPPHYASQPLQQPYLPPLSQHGRLPAQQSNVRAQSSSSVLPYKQQRPNFAKEENIKDKEVADLLDAIEEFHNQTLQIQEPIKPIPISSLITIGDSECNDNKLKIIMLENIGKDLNATKKMIQLAAEAQFDGHFNVICSKDDFSFLTNTELFCQATKGDISCYAYRLYL |
| Bm | grl-14 |  | grl, ground-like |  |  |  |  |  | AFRSIQNFHHQRQKHKEDNSATNANQIVVDNNRTILLLNDQRKKQRESLIDPINVTFNDWETFITDYDKVMPQYNRRHAISNEYGATNLNSMSNPFIPVNESSNNNAFEYTPQIRQMQSSGIAPNSYDYQQFARSLNSAQQMLSNGAEATEHLPPPPNPKSTEASVHTVQVYPPSQQPDYYQENRMYQNNRIDQKFGDRYGGRANFEQPLTQLMSNDYNIDTASYIPEVNPNCINDPCEIDNGNPFGFIEDERCNSPRLKEIILQNIVERDAEASKQAIYNVCETEMEIPCNVICGTGFYSYLARATNFCLVSMMDISCYAFLPACNFNLNLNQKQWIRRHRTKV |
| Bm | grl-16 |  | grl, ground-like |  |  | SWAMCAC34C05 | AI919632 |  | GDKAVADNIAPGYGGGMRGSEDGIVGFTPPANIAPPPPPPPLELPLPLPKQYAYNNDMMQPEEQLAPAAMTQESRAPEGSMVTSDSNNKYDQTESHTSINDGTNYKTNSAYQSDTSAAESDHGATTALATAMRSAEFTSLESSGLDAKNNAISEAIAGYDKSNGNAIIESRSIETDDSTCNDPVLKAIIESTLKEHRDNLDAARSIESKASKRFGGRFNSIGSDSEFAYVNWYGKRNCQLQFNGRHSLTWED |
| Bm | grl-17 | grl-2 | grl ground-like |  | AA161640 | AFCG0B02 |  | VQA-FL | MTLILISVILLKISVPVQAFLLGTGNNCCCECGIPMPQSCGCATPAPLPICPPPIPCPPPICPICNQCPLPTPCPPQPIALCPPSYPVYLPQSSCNCGAGNTAAGTYTSPARGQNFGPTQKGIYDSVRNVAQSAGLAVAHGASPPSVNPQNLLGTETDIQNTSDIQQFPSIQNEAEKLNDPTFLNPDITHQLSPLSSTAASITERSAHGVVIISENKCNSLVLRELLKKNMDETDPVISKRSIHKAFQESIKENDLDIICSDAGFTYIVSTTEYCEAQKEKVICFVYKKT |
| Bm | grl-Ax | grl-1 13970.m00031 | grl ground-like |  | AA841818, AA841945 | 3D6V7F09, 3D6V9C09 |  | TFA-GC | MMAYFRLLAIILSHSIVNITFAGCGHHGRSHQPYPRPLYQPYPRPQPPQRPCPPPPPSPPCPPQPCPSLPPPTPCPPQPCPPPPPPVPCPKPPPCPPPPPPIPCPPPLPPKPCPPPPAPPPCPPQSCPPPPLPPPCPKPPPPIPCPPPPPPKPCPPPPSPPPCPPPPPPQPCPPPPLPPPCPPTYCTPPPPSQPPVTYSPPPKPYPYVPECPSLPPPSPSDCKTDSEVCTKCNQQSKPSKSPNRSMAGTYFRTNKRLSRKSERLNRRNKREETKIVMSDTCNSHDLMEIMDQNLSTNLAISKHLIQKYAELKLKRDFNVICSNNSFTFITHATLYCQTVKFNVSCYAFTAD |
| Bm | grl-Axb |  | grl, ground-like |  | AW347982 | SWYD25CAU10A10 |  |  | MMAYFRLLAIILSHSIVNITFAGCGHHGRSHQPYPRPLYQPYPRPQPPQRPCPPPPPSPPCPPQPCPSLPPPTPCPPQPCPPPPPPVPCPKPPPCPPPPPPIPCPPPLPPKPCPPPPAPPPCPPQSCPPPPLPPPCPKPPPPIPCPPPPPPKPCPPPPSPPPCPPPPPPQPCPPPPLPPPCPPTYCTPPPPSQPPVTYSPPPKPYPYVPECPSLPPPSPSDCKTDSEVCTKCNQQSKPSKSPNRSMAGTYFRTNKRLSRKSERLNRRNKREETKIVMSDTCNSHDLMEIMDQNLSTNLAISKHLIQKYAELKLKRDFNVICSNNSFTFITHATLYCQTVKFNVSCYAFTAD |
| Bm | grl-x1 |  | grl, ground-like |  |  |  |  |  | MTEMFRQCGIMFLVTVHVYVIEASPCRCSSCQTSGVSCSKPVVCPTPVSCPLQTCPVPSPCPQPPPCPPCLNTVIQQPVLVTYRVIVPVVRKIPIIENTCCSTCAIPCIARKKRELLAEDELLINNSTKLPVNPVCNSKSLQEIMSENISTTAAESQRLIQKVSETQLGGHFNVLCSNSDLSYSVLTTSSFCQYQKDNIICYAFKMP |
| Bm | grl-x2 |  | grl, ground-like |  |  |  |  | IES-FV | MGQLSSATISLIFYITTIESFVSGHGCGQGPPAPCGAPPPPPPPPPPPPPPPPPPQPPCSCPQLCLPCPPPLPPLPPLPSLPCPCLPCPCMPMLLNSCCNTCQRPCSFRSRRRRSLNARALARGMQILNDDPVCNNAELQSTIKQNLVDDIWLTKEMIQKAVTEKYPSNRYSIICSTGDLTYTARTNDFCLVQQNNISCYVFRPL |
| Bm | grl-x3 |  | grl, ground-like |  |  |  |  | LRA-SC | MFNYLFLLSTTQFVLFGLLRASCNCPSCPSPQPCSSCPPPVICQPKICPPVPVCPILLPQSCPVCIKPTIKTVPIPVVKPIYKIINKPIILNECCKTCGKQCIKRMKRNISNDTIITVNSVCNNKILGEIISKMMTSNLIVSQKLIMKKVTKLLGEYNIFCSTGDLTYSAYTVDFCQVNKAGIVCYAFKNL |
| Bm | grl-x4 |  | grl, ground-like |  |  |  |  | YQA-TF | MVVLFFLLALIPQYQATFLGPSQDCNCPQTSLCSVCPISCPSLFPPLCPPQPPCPPQLPPPPPPPPPPPPPPQASAPCPQQPLCLPCPPPIPMQPLCPPSLQHLLLPCPLPQPSNSPCKRKKRSTPITSVASDYGTNIHASCTSNYIRKIILKNLSTDAKISKAAIYSELKAKQKGDYVVLCSQSSLSFTSDSTNYCVGGNTNHLCYVFEL |
| Bm | grl-x5 |  | grl, ground-like |  |  |  |  | IQG-RC | MFHQLLIIIISYGLFQSVIQGRCIGAGCDKHQSLKSLRFAPVNCHNQHYHITKFCSDCXADKNVISNNCCNCCNCCCNQISSHXXTNDNDDSTGDATVTATGCGGPSTDCNSRDATESDTSKDDDESPPTSVTSISGKASSEFKSNYPSVPSSCPTSSSCKTISNPQRFSRNSDTGIFKKNITLPLLPAGVPIIPSPQLDLLTAGGNVKFSKIRDLHLSTIKPPSVKKSLAQSNIQEQIVNNFPSHDCCTRCNGANCMPRNGNAILLAANTFQNRHDHDTKCKSKELAKLMMANMKRDPTRSKRAIQRAAEQNFATQFNVICSKGDFSYVTHAIEYCEVSNRGTTCYAFRIS |
| Bm | grl-x7 | 1133571 | grl ground-like |  |  |  |  | TDS-DN | MSHDDGYYISKRSLLLIIFVLNLSSFNATDSDNMRCKCISESKSKCKLTCLDGTNKTDTNHNIERREIDYEEVEIIDERKCNSKSLQNIMLQNMAGQTAKQAKTKIMRAAEALLGGYFNVICASGDFSYITTTSLYCLQSLGNINCYAFLTGNTRPRL |
| Ov | Onchowrt |  | wart like |  | AA293990, AA294416, AA294323, AA618812, AI053015 | SWmL3CO1348, SWmL3CO732, SWmL3CO622, SWmL3CO1769, SWOv3MCAM04D09 |  |  |  |
| Mi | Msp3 |  | grl, ground-like |  | AAQ10017 |  |  | SEA-FG | MPKLILLFYLIIYGILLLISLSEAFGFGGGCGCPCMPQPCIPQPPPIALPSLCFPQIQLPCPPPSCGCCGRRKRESGASALLTAVSTKSGIKRIGEEKNHCNNPHIKRIILKNLIIGDWVGTRNAIYSELRAKLGGNYIINCAHAPSFAYSGDSVIDYCVDGHQAITCAVFKIQ |
| Mi | wrt-6 |  | wart hog | clone rb25a03.y1, EST covering Hog, lacks wrt domain |  |  | BM882030 |  | FTTLQCFSGDQLVNTPKGEKRMDELKIGDLVLSVDESLVAYSPILMFLHRLPNEKAIFKIITTLEGQQLKLTEFHLVWAGCSNLRLIRAKDLNNGECLYTVKNKKNNITSLIELNKEMMGLSSTKIIKINEIEENGIYAPLTSNGNILVNGLLASCHSNIAAQTLQQTF |
| Pt | wrt |  | wart hog | clone kx48g02.y1 |  |  | BI863815 |  | NVYVGECVNKVGDDGELKPIKLINSTVKKVIGAYSPLTKNGNILVNNISASCYSVVKSNSLAHSFFHYINEINKIISSIATSFATKTGVVSEEETVELPAVAKYLYDTLNLIIPETIFDGETRNMFMKH\* |
| XC | Thog |  | T hog | clones kc92h04.y2, kc49d12.y1, kc51c05.y1, kc97a08.y2, kc71b07.y1 |  | kc92h04.y2, kc49d12.y1, kc51c05.y1, kc97a08.y2, kc71b07.y1 | CV579829 CV511906 CV512053 CV511906 CV580091 CV512053 CV508357 CV511906 | GQA-TT | MFKLITLVSCILLLHFHGLLIVNGQATTTTTATTTTTTPTTTTLTTTTPTTTTSTTSTSTTSTSSTSSTTSTSTTSTVSTSTTSTTSTASTTTSPGQTTTKKKGS CFHSTGTVQTAQYGVMSIGQLFHTYKDAHVLSRNALGQLEYAPVEYWLHAEPTTSEEFIELGTEQGHRLLLTPDHLIYKTDCEGSTERAVLSDKVDPGDCVRVKGGQALVTARVVSKVRTLRTGIYTPITSTGSIVVNDVLASCYAGYEDEAMQKLVFKLLIWVDWAGRNILPSTVYQALFRSDPINTAHVPQILRSMWEISD |
| XC | Hh |  | hedge hog | clone kd05d04.y2 |  | kd05d04.y2 | CV580690 |  | RCKAKLDTLAMLVTNFWPGVKLRVTEAWDQDGFHSADSLHYEGRAVDITTSDRDRNKYGLLARLAHQAGFDWVHYENRMHVHASVKSDSVTRHKRVLK CFPGHATVTTANGIRKFMSDLSVGDMVAVSTGHVNSDQYYQPIIAFLHRNTSMPAKFVQIRTSPSGKVFTASSEHLIYVWNGQNGSRS |
| XC | Xhog1 |  | Enop hog | clone kc65e04.y1 |  | kc65e04.y1 | CV510963 |  | KSGGHNGDNGGDCGVTGCHNNPPLEGGSPEHYQKQNVPLPGKPPASASDPKAWGIDNSPRGAGGAG CFSADTIVHTPHGEKRMDQLRVGDHVLSTASWDGNSAISYQPITQFIHNDPLVVAGYTVISTVSGRSLSLTPYHLLPQFECHHLHHQSISVRQYELLFNQWARFAKRARPGQCVLMVNSEGHFSAERISNVTRTVGRGVFSPITSSGIIVTNGFQASCYSSVENHAIQHTFFTCV |
| XC | Xhog2 |  | some hog | clones kc79a04.y1, kc73b11.y1, 1 frameshift introduced to maintain similarity |  | kc79a04.y1, kc73b11.y1 | CV509027, CV508530 |  | SIDSKPIYTAVEWLYHKDPEMQADFISISTKSQKTIRMSPNHLIPVVSCNTTDFQKMGLAHFTNSHSIFAGRLKMGDCIATLVNNELIADKVISMKTETKKGIFSPITSHGTIIVNDIYASCFSTFENHMLQQSVHSILINIRNRMAKMWSGVFGENVLAPEDKGIPLPLQMLLKMAQFMLPANVFTV |
| XC | Shog1 |  | X hog | clone kd05a07.y2 |  | kd05a07.y2 | CV580660 | LKA-NA | MLTLSTLLIIIVLIDMDDRLKANALGATLAPPPMMAS CFHGSGSVQTEQYGRITMSQVFHSHQDARVLTRNDKGQLEYTSIVYWLHAEPDRNQSFLEITTDTSHRLLLTAGHLIYRTDCTGGPARAVLAQKLKPGQCVLVKKEQSLIQVKISSIQNRFLPGIYAPITETGTIIVDDVLASCYVVYEDEAVQKMIFEHFLAVEKTLRGILPSSLYKIIFRNQDLNRANVPR |
| XC | Shog1b |  | X hog | clone kc91d03.y2 |  | kc91d03.y2 | CV579699 |  | MLTLSTLIIFIVFVDMDDRLKVNGLGATLAPPPMMAS CFHGSGSVQTEQYGRITMSQIFHSHXDARVLTRNDQGQLEYTSIVYWLHAEPDRNQSFLEITTDTSHRLLLTPGHLIYQTDCTGGPARAVLAQKLKPGRCVLVKMEQSLIRVKISSIQN |
| XC | Xhog3 |  | X hog | clone kd04b04.y2 |  | kd04b04.y2 | CV580586 |  | GGPR CFSGNMMVSTPNGQKRMDRIRAGEEVLVTLTDSKPIYEPIEWLYHMNPDIEADLITLRTRSGRVLQLSHNHLIPVVPCSNDHSHKLDVVQFASNQSIFAGRLKLGDCVATLIDNEFRSDKIVSMVQERHRGIFSPIPRQGTFVVN |
| XC | XHog4 |  | X hog | clone kd28a06.y3, 1 frameshift introduced to maintain similarity |  | kd28a06.y3 | CV568345 |  | SATVVPFTRTNTMAAAPPPPPPR CFSGESTVFTEAYGRLTMGMLFSVYQDARPLARNDQGQLEYTDVAYWLHAEPNVRSPFLEIWTNSSHRLQLTPEHLIYQTDCQGGPERAVFAEKVKEGDCVLVKQGQALVQARVIAKKKRHLTGIYAPITNTGNILVDDVLASCFAVYEN |
| XC | Xhog5 |  | Enop hog | clone kd06f09.y2, manual 1 frameshift |  | kd06f09.y2 | CV580800 |  | GQVSKNDCKFIHDAIACCKSDCGNGGGQQTACQGKLTRRGRCRRRRG CFHGADIVQTKEYGNMTIHELSKHRDGRVLTRNDDGQLDYTPVRYWLHAQPTTSMKFLILHTESGHRLSITADHLIYQTECRGGPGRAIFAKNVEIGRCLYINEKWGFEGNSSISEGSKKMTGIY |
| XC | Shog2 |  | short hog | clone kd20b11.y3, made manual frameshifts to retain similarity |  | kd20b11.y3 | CV568678 | GHC-CG | MATLWLLVTLAYVSITHIPSGHCCGAGVGGAAAGRAVGGSIVGTFGGK CLYIDANVRMSDGSMKRVKDLQIGDEVLAYNEAQGIHPSRIFGELHYDNETMMKLVEIETSSGRRIPVTPAHTVFVRQCLSDGTNWLTKSAYEVEVGECVPRYYTDDGDVVEESLVNVRLFDGVSVTQPVTETGTILLDDVVLSCYNPVINQKPTYFKIIPLLMNRMYIATTNIPHIQSIIQPIHAPTRIVTPTQHSPSIYDSYILRICLLQPPI |
| Ts | Xhog1 |  | Enop hog | Contig 13, pos ~208674 rev.comp.; clones pt19g01.y1, pt19e07.y1, pt18c09.y1, pt34f11.y1, pt14h06.y1 |  | pt19g01.y1, pt19e07.y1, pt18c09.y1, pt34f11.y1, pt14h06.y1 | BQ738709, BQ738691, BQ738588, BQ693402, BQ692434 | IVA-VH, VHG-LT | MRSISKLPFPMFSLIFCIISCWWGGIVAVHGLTCPDETDAKSFIQPQLVKKSSCQEIMRHECLFIHEARDKCPCLCERLIQSVDTAEQTVVAAESENGRAR CFHGDSIVQTEQGPMQMKEALGKSNLRVLARDADQNLVYSPITSWIHANKDRSTEFVQIVTDNSKKLLLTDLHLIYESDCQGGPARSVMAKDLTVGRCVYTMDEQRQQLRESTITSLRREIKAGFYSPITAEGNIVVDDVLASCFSTVGSEGLQKIAFAYIGWLRRMLASILPEQLYEVMMFSTAVGDIKLPSLLVGLIDISKHVIH |
| Ts | hh |  | hedge hog | Contig 14, pos ~870498 |  |  |  | VHS-AK | MLSWCDLLFFLSVTFSSNVHSAKVYCWRWCSSGRMAGMAYKKYYPLIYNERFPNEPENAPGSAGAAELRIRRSDPGFKRLVQNNNPDIIFRDEENTGADRMMTYRCKQKLDMLAILTMNYWPNVKLRVIDAWYEQNRYSRSALHYEGRAVDITTSDRDRNKLGMLARLAIQAGFDWVYYESHLHVHASVQPDSVFRTKRMLN CFAGSSTVLTKENGRKRMDELEIGDRVLARKADGKLTFSPVILFLHRDQQTKAKFKAVHISNDTLLTISPDHLIYKVDPMTNDNSGGSMETVFAADLLAGDQVFVRNGIFDISRATVLGIGEIERQGLYAPMTLEGNIFVDDVLASNYAGTSYETLAHVSMAPARLYWNVASTIFEQLGPTTAPTHYHIHWYARWLWTLADNVSTFVGIPSPLDYFPRP |
| Ts | Xhog2 |  | Enop hog | Contig 13, pos ~203768 rev.comp. dup xhog1 |  |  |  | ANG-AA | MHKLLLELGLTLMLIATVANGAAMCPGDAGERTFIQRQLSSKRSCTELTRTDCLFIHEAREKCTCTCDNLIASNVQTEAIAEPAVEMVENGGRAR CFHGDDWVLTTNGRMQMKHLLQKKDAQVLTRSENGHLEYSPVMTWIHAQKETKAQFINLETESGHRLSLTPLHMIYQTNCDGKEMVLMAEKVAVGKCIFVKADNDKLVESKVVSTSKVVKTGIYSPITTSGSIVVNDVLASCFSTSANEDIQRLLFKYASFVYSLFTCPASLISDSFSHQQQDYVEIPKLLLGALNLQKYLIQ |
| Ts | qua-1 |  | qua hog | N-terminus FGENESH prediction, one manual intron in Hog, Contig2, pos ~1157554 rev.comp. |  |  |  | ALA-ST | MMFIVVCVCFLINQALASTFDPAEFSIFQCQSHEALSIQGRGTGFVKLSCAKPVACSKTQNCKMTTFNATCSGPNEYVHGIIKLPEHKINQICCQLADVPSSLTINERCFTDSITNQTLTEVQSALNEKERLPYSYLARDDYAPAFVKLFRLAHQSDGVRIVKKIKAVEHGYLITSCQLDCIDGTLPEGSVPPEEGILCDEACQRLLEKNRENSAVNSVEVSPTSLPEKQLTGEPVIADNALVPRISSAGAGGGGAVQADGNLQDRHATNLASSASSSEHSAPGQWQPCSYCGPFIPRAAVNSAINPIPSATNPIGGG CFSADMHVRTTHSQIRMDQLQLDDIVFVDPVEQQPIFSMLHHDPAAEVDFIIIKTETNRSLSLTPNHLIPIVPCRRGILPAEKLEATVNRYSKFAHKAEQDECVLMAYGGLVKTEKIVAISQRRLRGIFSPLTEKGTIVVNDFVVSCYSTCESHALQKLFHNSIRHISRMLRNALFIQLPIYLKSLYKLMHWTVSMTVA |
| Ts | Xhog3 |  | X hog | contig 6, pos ~2212436. N-terminus a bit uncertain, but must be in small region, since 600~ bp upstream of Hog is another gene. |  |  |  | IMT-VV | MRTTSKTWRNVAIASVIILILAVVIMTVVIVILIQSQSQANSSDPSPTAQPPEEDGEDIPG CFPSDALVKTRSRWKTIQELHIGDEVLDLDERGRPVYTEIFAWLRKDMLGHSFVKITTADGISLTLTPNHLIFKLIPDCTTPDNRTVQLSDYEAVLAGEIQKGDVLVQMDDAWNGARHATVTEIHNIRSRGAFAPATLSGTLIVDGTLVSSYTSYWFFDFIGHRFVHEIIFAPLRFFYAFFKLVVKHQPTVDALFSPTYSGTHWYVEIWKPFRSAIS |
| Sp | hh |  | hedge hog |  | NP\_001012720 |  |  | TQA-CH | MVRADMVKWLTVQITTVLCLIALTQACHPGRSGKTSHRPRNRTPLQYKQRVPNISEDTFGASGPPEGRINRNDERFNTLSPNNNDDIVFKDKEGTGADRLMTQRCKDKLNTLAISVMNEWPGIKLRVVEAWDEDQPNVEPLHAEGRAVDITTSDRDKNKYGALARLAVEAGFDWVNYESKAWVHCSVKSESAAAKNSGGCFPGFSQASLENGRTISMLDIRVGDEVAVVNDDGALDYSDVIMIVHRKLNDSTLFYVIETEDKSVVQLTPQHLIYVSETESSFSQSKAMFASEVRTNQFVYTTGQNHDRGVRPKRVVSVTTRLGRTAVAPVTRQGSLVIDDVAISSYAVMRDEWIAHASFAPVRWYSYIRHNMLGIVDTNTGQEQRVHWYTQRLYKLGKYVMSDRLFLGFDV |
| Lv | hh |  | hedge hog |  | AAC15065 |  |  | TQA-CH | MVHADMVKWLTVQITTALCLIALTQACHPGRSGKTSHRPRNRTPLQYKQKVPNISEDTFGASGPPEGRIDRDDERFSKLSPNNNDDIVFKDEEGTGADRLMTQRCKDKLNTLAISVMNEWPGIKLRVVEAWDEDQPNVEPLHAEGRAVDITTSDRDKNKYGALARLAVEAGFDWVSYESKAWVHCSVKSESAAAKNSGGCFPGFSQAYLKNGRMISMLDIRVGDEVAVVNNNGELDYSDVIMIVHRKLNDSTLFYVIETEDKTIVQLTPQHLIYVSERESNFDQSRAVFASEVRTNQFVYTTAQNHDRGVRPMKVVSVTTRLGHTAVAPVTRQGSLVIDGVAVSSYAVMRDEWVAHASFAPIRWYTYISHMLGITDTDTGQEQRVHWYTQGLYKLGKYVMSDRLFPGFDV |
| Tn | Hh | CAG07416 | hedge hog | c-terminus fishy, too long | CAG07416 |  |  | VQG-CG | MKQSWWARLAQVSLLAAWSCIWMVQGCGPGPGYGIRSRPRKLKAMHYKQFFPNLSENNLGASGRAEGKITRNSEHFNELVCNYNPDIVFKDEENTNADRFMTKRCKDCLNRLAIAVMNQWPGVHLRVTEAWDEDGHHPPGSLHYEGRAVDITTDDRETEKYGLLAQLAVEAGFDWVHYESKYHIHCSVKADHSVAVEKGG CFPGWARVTLPGGFQKSLSSLTPGDRVMALSETGQVVFSPVLMFLHRDPESKSRFLSLQTEDGHRLAVTPHHLVFSDAYCRPDSRQYRAQFASRARTGTCIVVLAAEGQLRPSRIVSVSEEENTGVYAPLTEAGNVLVDGVLASSYALVEDHRLAHWAFGPVRLLFSFSRLLWAEPGQQSDRGTAPLGSCTMAACGSGACVRNSTSAGSRADGQGHMSQVHWRSKSAQGMDEQKSNCEENDSEPTADDNAPSQQLVLSEEESKTCTAAEDVSGFTVASSSTHIESVQVCALCNCVEWSLHGQRELRYFGPFSEWRTLQPSSTPLPQPGNDDLSSIGFSVSPCLAALLDDSG |
| At | Hh | BAD01490 | hedge hog |  | BAD01490 |  |  | ASS-CG | MFHTLAAVLCLAALVNSASSCGPLRGGGRRRPPRKLTPLVFKQHVPNVNEFTLGASGQSEGKLTRDHPKFKSVLVPNYNSDIIFRDEEGTGADRLMTQRCKEKLNTLAILVMNQYPGVKLRVTEGFDEESYHSTQSLHYEGRAVDVTTSDRDRSKYGMLARLAVEAGFDFVYYESRSHIHCSVKSESADAGRSGG CFDGDSTVRTEAGPKKMSDLQVGERVQVARTDGQTDYSEVILFLDRNETQQRLYNTLETENGRSITLTPTHLIFTASPHQTTPQATFAKHVEIGDYIYVASDRKVTLEKVISVTSSAKKGVFAPLTREGNLVVDGVVASCYAIIEDQALAHFAFAPVRLIDNVWEATLHLLRTMHILRYRESRTIPPHNGIHWYANFLYSIAHKLIPED |
| Pv | Hh | AAM60752 | hedge hog |  | AAM60752 |  | AF435840 | THA-CG | MKLLPPFSSICSFAFIILFITSLTHACGPGRGSGSRRKAKKHTPLVFKQNVPNVSENSLGASGMSEGRIKRDDAKFKDLVRNHNADIVFKNEEGDGSDYHMTRRCQDKLNSLAVSVMNNWKGVMLRVTEAWNDNNSHAKDSLHYEGRAVDITTSDKDRAKYGMLARLAVEAGFDWVYYESRGHIHCSVKSDSSVAIKIGGCFPGTGVLQTETGWKTMSQVVAGDSVLSMNSNGKLEYSPVIAFIDRNERELERYITLHTEDKKDITLTSKHLIYMSTSNVTTDDVTDSFNVVYADDVIEGDYVLVTSDPVGEVIKPTRVLTISEHTIQGVYAPLTLNGNIVVDGVVVSCYAVVSNANLAHVVFAPVRGLHVLSQYVPWLAPSTHHQNFTQNGVHWYAKLLYNIGSTFLSAETLHVP |
| Gb | Hh | BAB19658 | hedge hog | N-terminal residues removed to give nice Met with signal sequence. Frameshift introduced at very C-terminus | BAB19658 |  | AB044709 | ARA-CG | MPLLLRWLLLLLLMQGAARACGPGRGAGRRRASRRLTPLVFKQHVPNVSEHTLGAAGPAERRVARDDPRFRDLVPNYNADIVFKDEEGTGADRLMTQRCKEKLNTLAISVMNQWPGVRLRVIEGWDEEGGHAADSLHYEGRAVDVTTSDRDRSKYGMLARLAVEAGFDWVYYETRGHIHCSVKSESSQAAKSGG CFAAESTVQTPGGLCALAELRVGRRGAGAGPGHGRLAFSPVLLFLDRDPAPRTLLRVRTASGRTLALTPSHLLPVARAGGGEPEARFADAVRPGDALLVAADAGGAVRPDRVLHVDAEATRGGVVAPLTAAGTVVVDGVLASCYAVVGSHSLAHWSFAPVRAWHWLTAWGHAAPDYAHPPPPARAAPGVHWYAKALYSLGQVLLPGTMLYK |
| Dh | Hh |  | hedge hog |  | P56674 |  |  | AHS-CG | MRHIAHTPRGSCFMALLLLLLLALNFRHAHSCGPGRGLGRRRERNLFPLVLKQTVPNLSEYHNSASGPLEGAIQRDSPKFKNLVLNYNRDIEFRDEEGTGADRVMSKRCREKLNMLAYSVMNEWPGVRLRVTESWDEDRQHGQESLHYEGRAVTIATSDHDQSKYGMLARLAVEAGFDWVSYVSRRHIYCSVKSDSSPSISHMHGCFTPESTALLESGAEKALGELAIGDRVLSMDVKGQPVYSEVILFMDRNLEQVENFVQLHTDGGAVLTVTPAHLISVWQPERQTLNFIFADRVEELDYVLVRDATGELQPQRVLRLGSVQSRGVVAPLTREGTIVVNSVAASCYAVISSQSLAHWGLAPMRLLSTLQSWMPAKGQLRTAQDKSTPKDATAQQQNGLHWYANALYKVKDYVLPKSWRHD |
| Dm | Hh | CG4637-PA | hedge hog |  | NP\_524459 |  |  |  | MDNHSSVPWASAASVTCLSLDAKCHSSSSSSSSKSAASSISAIPQEETQTMRHIAHTQRCLSRLTSLVALLLIVLPMVFSPAHSCGPGRGLGRHRARNLYPLVLKQTIPNLSEYTNSASGPLEGVIRRDSPKFKDLVPNYNRDILFRDEEGTGADRLMSKRCKEKLNVLAYSVMNEWPGIRLLVTESWDEDYHHGQESLHYEGRAVTIATSDRDQSKYGMLARLAVEAGFDWVSYVSRRHIYCSVKSDSSISSHVHGCFTPESTALLESGVRKPLGELSIGDRVLSMTANGQAVYSEVILFMDRNLEQMQNFVQLHTDGGAVLTVTPAHLVSVWQPESQKLTFVFADRIEEKNQVLVRDVETGELRPQRVVKVGSVRSKGVVAPLTREGTIVVNSVAASCYAVINSQSLAHWGLAPMRLLSTLEAWLPAKEQLHSSPKVVSSAQQQNGIHWYANALYKVKDYVLPQSWRHD |
| Ag | Hh | ENSANGP00000015828 | hedge hog |  | XM\_321721 |  |  |  | MVWLMVALLALVQLTAGCGPGRGIGGPRRTRKLLPLVFKQHVPNVSENSLSASGMQEGPISRNDSKFRSLETNYNKDIIFKDEEGTGADRVMTQRCKEKLNILAVSVMNQWPGLRLMVTEGWDEDHMHAPESLHYEGRAVDIMTSDKDRSKIGMLARLAVEAGFDWVFYESRSHIHCSVKSDSSQSSHTSGCFTGDSTVLTEAGVHRKLSELRIGERVQAVDAAGRTVFSEVLMFMDRDTHQRREFVTIEAEGGALLKVTPAHLVMVWRRERSETRFVFADRVREGDHILVHVAGSLEPRAVHRISATLAEGVYAPLTGEGTIVVDSIAASCYALIDSQTVAHWSFLPYRLAEKVSALFDRTDSLSLPRHEGIHWYAKSLYTIKDYLIPSNWLYH |
| Bf | AmphiHh |  | hedge hog |  | CAA74169 |  |  |  | MAGVLARWMVTLVAISALGTHWGPSEACGPGGRFGRRRHPRKLTPFVYKQQMPAVSENTFGASGLFNGRITRDSERFHTLKQNFNTDIIFKDEEKTGADRFMTQRCKDKLNALAISVMNQWEGVKLRVTEGWDEDGFHTEESLHYEGRAVDITTSDRDRTKYGMLARLAVEAGFDWVYYESKAHIHCSVKAESDTTATQGGCFSAESWVTRDDGNRIRMRDVRPGDKVLSMDSGGHPVFSEVLTFMDRESRGPWVYYTIHTDDRNITVTATPSHLVFVTESRDLSSPRIAKFMSDARPGEFLLTPDSDGGGFRKVKIVSVTMREEKGAYAPLTVHGTVVVDNVAMSCYALIESQALAHWVFAPFRLYYQLTSSLWDGPSHDQTLQEGVHWYPSFFYRYGISLVEPTLLHPTATDS |
| Ob | Hh |  | hedge hog |  | AAZ99217 |  |  | SLA-CG | MPQRSLRHQLGMILVFFLLVTSHSLACGPGRGPGKRRGPRKRTPLVFKQHIPNVSENTVGASGIHEGKITKPDPRFKEMVTNLNPNIVFRDEEENNEDRVMSKRCKDKLNTLAIAVMNEWPGVKLRVTEAWDTQGHHAPTSLHYEGRAVDITTSDRVRSRYGMLARLAVEAGFDWVYYESRSHIHCSVRSDSLDTTHYGGCFPRTGKVVVRNKGTITLDQLKVGDSVLSVDLQGELTYSEVIAFLDTNKDSSGYFHRIETENGHTIRLTGKHLIYSSYTNRTRFDLNDNDSEFEATYADQVQIGDYVMTTDRTAGLFASRVKKIAAVSEKGVVAPLTKSGNIIVDGVVVSCYALINSDYIAHASFFFLRGLHQVTSHIPFVSWAESPLASYAIDGIHWYAKLLYKIAPLFLDRTLLYMND |
| Cap | Hh |  | hedge hog |  | AAZ04357 |  |  | AWA-CG | MHFHDHLFFVFCLLALSRPAWACGPGRASGRRRGARKMTPLVFKQHVPNISENTLGASGLNEGRITRDDPRFKDLVENYNPDVVFKDEEGTGADRIMSQRCKDKINTLAISVMNQWPGVKLKVTEAWDEDGFHAKDSLHYEGRAVDITTDDRDRSKYGMLARLAVEAGFDWVYYENRGHIHCSVKSDSSITAKTGGCFSADDTVKRVDGSSLPIQHLRIGDAIQASTDNGDVVYSPVILFLHREENAVASFVTLKTEGGRSLTLSPSHLIHTAEHGEIYASDVKIGQHLLALNNNRSLDKDPVVAMTTQYRRGVFAPLTAIGTIVVNDISSSCYAHVQSHAFAHAFLAPVRWHYQVLPVSDSPQEGVHWYVQLLYDISTYVLPSKMVFSPS |
| Mm | Shh |  | sonic hedge hog |  | A49425, Q62226 |  |  |  | MLLLLARCFLVILASSLLVCPGLACGPGRGFGKRRHPKKLTPLAYKQFIPNVAEKTLGASGRYEGKITRNSERFKELTPNYNPDIIFKDEENTGADRLMTQRCKDKLNALAISVMNQWPGVRLRVTEGWDEDGHHSEESLHYEGRAVDITTSDRDRSKYGMLARLAVEAGFDWVYYESKAHIHCSVKAENSVAAKSGGCFPGSATVHLEQGGTKLVKDLRPGDRVLAADDQGRLLYSDFLTFLDRDEGAKKVFYVIETLEPRERLLLTAAHLLFVAPHNDSGPTPGPSALFASRVRPGQRVYVVAERGGDRRLLPAAVHSVTLREEEAGAYAPLTAHGTILINRVLASCYAVIEEHSWAHRAFAPFRLAHALLAALAPARTDGGGGGSIPAAQSATEARGAEPTAGIHWYSQLLYHIGTWLLDSETMHPLGMAVKAS |
| Mm | Dhh |  | desert hedge hog |  | NP\_031883 |  |  |  | MALPASLLPLCCLALLALSAQSCGPGRGPVGRRRYVRKQLVPLLYKQFVPSMPERTLGASGPAEGRVTRGSERFRDLVPNYNPDIIFKDEENSGADRLMTERCKERVNALAIAVMNMWPGVRLRVTEGWDEDGHHAQDSLHYEGRALDITTSDRDRNKYGLLARLAVEAGFDWVYYESRNHIHVSVKADNSLAVRAGGCFPGNATVRLRSGERKGLRELHRGDWVLAADAAGRVVPTPVLLFLDRDLQRRASFVAVETERPPRKLLLTPWHLVFAARGPAPAPGDFAPVFARRLRAGDSVLAPGGDALQPARVARVAREEAVGVFAPLTAHGTLLVNDVLASCYAVLESHQWAHRAFAPLRLLHALGALLPGGAVQPTGMHWYSRLLYRLAEELMG |
| Tr | fhh |  | hedge hog |  | AAC34384 |  |  | VQG-CG | MKQCWWARLAQVSLLAAWSCVWLVQGCGPGPGYGIRTRPRKLKAMYYKQFFPNLSENNLGASGRAEGKITRNSERFNELVCNYNPDIVFKDEENTNADRFMTKRCKDCLNRLALAVMNQWPGVHLRVTEAWDEDGHHPPGSLHYEGRAVDITTDDRETEKYGLLAQLAVEAGFDWVHYESKYHIHCSVKADHSVAVEKGGCFPGWSRVTVAGGFQKSLSSLTPGDRVMALSETGQVVFSPVLLFLHRDPESRWRFLSLQTEDGRRLAVTPHHLVFSDAHCGPDSSQYQAQFASRAQTGTCVLVHTAGGEVHPSRIVSITEEESVGAYAPLTEAGSVFVDGVLASSYALVEDHQLAHWAFGPVRLLSSVSQLLWAEPEERSDGSKTPLQPHALVRGDRKVCARNSTSVRSEAGPRGRTSEVHWYAQLLHRLGWIVLNPDLFHP |
| Mm | Ihh |  | Indian hedge hog |  | P97812 |  |  |  | MSPAWLRPRLRFCLFLLLLLLVPAARGCGPGRVVGSRRRPPRKLVPLAYKQFSPNVPEKTLGASGRYEGKIARSSERFKELTPNYNPDIIFKDEENTGADRLMTQRCKDRLNSLAISVMNQWPGVKLRVTEGWDEDGHHSEESLHYEGRAVDITTSDRDRNKYGLLARLAVEAGFDWVYYESKAHVHCSVKSEHSAAAKTGGCFPAGAQVRLENGERVALSAVKPGDRVLAMGEDGTPTFSDVLIFLDREPNRLRAFQVIETQDPPRRLALTPAHLLFIADNHTEPAAHFRATFASHVQPGQYVLVSGVPGLQPARVAAVSTHVALGSYAPLTRHGTLVVEDVVASCFAAVADHHLAQLAFWPLRLFPSLAWGSWTPSEGVHWYPQMLYRLGRLLLEESTFHPLGMSGAGS |
| Hs | SHH |  | hedge hog |  | NP\_000184 |  |  | GLA-CG | MLLLARCLLLVLVSSLLVCSGLACGPGRGFGKRRHPKKLTPLAYKQFIPNVAEKTLGASGRYEGKISRNSERFKELTPNYNPDIIFKDEENTGADRLMTQRCKDKLNALAISVMNQWPGVKLRVTEGWDEDGHHSEESLHYEGRAVDITTSDRDRSKYGMLARLAVEAGFDWVYYESKAHIHCSVKAENSVAAKSGGCFPGSATVHLEQGGTKLVKDLSPGDRVLAADDQGRLLYSDFLTFLDRDDGAKKVFYVIETREPRERLLLTAAHLLFVAPHNDSATGEPEASSGSGPPSGGALGPRALFASRVRPGQRVYVVAERDGDRRLLPAAVHSVTLSEEAAGAYAPLTAQGTILINRVLASCYAVIEEHSWAHRAFAPFRLAHALLAALAPARTDRGGDSGGGDRGGGGGRVALTAPGAADAPGAGATAGIHWYSQLLYQIGTWLLDSEALHPLGMAVKSS |
| Hs | DHH |  | hedge hog |  |  |  |  | AQS-CG | MALLTNLLPLCCLALLALPAQSCGPGRGPVGRRRYARKQLVPLLYKQFVPGVPERTLGASGPAEGRVARGSERFRDLVPNYNPDIIFKDEENSGADRLMTERCKERVNALAIAVMNMWPGVRLRVTEGWDEDGHHAQDSLHYEGRALDITTSDRDRNKYGLLARLAVEAGFDWVYYESRNHVHVSVKADNSLAVRAGGCFPGNATVRLWSGERKGLRELHRGDWVLAADASGRVVPTPVLLFLDRDLQRRASFVAVETEWPPRKLLLTPWHLVFAARGPAPAPGDFAPVFARRLRAGDSVLAPGGDALRPARVARVAREEAVGVFAPLTAHGTLLVNDVLASCYAVLESHQWAHRAFAPLRLLHALGALLPGGAVQPTGMHWYSRLLYRLAEELLG |
| Hs | IHH |  | hedge hog |  | NP\_002172 |  |  | AWG-CG | MSPARLRPRLHFCLVLLLLLVVPAAWGCGPGRVVGSRRRPPRKLVPLAYKQFSPNVPEKTLGASGRYEGKIARSSERFKELTPNYNPDIIFKDEENTGADRLMTQRCKDRLNSLAISVMNQWPGVKLRVTEGWDEDGHHSEESLHYEGRAVDITTSDRDRNKYGLLARLAVEAGFDWVYYESKAHVHCSVKSEHSAAAKTGGCFPAGAQVRLESGARVALSAVRPGDRVLAMGEDGSPTFSDVLIFLDREPHRLRAFQVIETQDPPRRLALTPAHLLFTADNHTEPAARFRATFASHVQPGQYVLVAGVPGLQPARVAAVSTHVALGAYAPLTKHGTLVVEDVVASCFAAVADHHLAQLAFWPLRLFHSLAWGSWTPGEGVHWYPQLLYRLGRLLLEEGSFHPLGMSGAGS |
| Dr | shha |  | hedge hog |  | Q1MTB5 |  |  | GLA-CG | MRLLTRVLLVSLLTLSLVVSGLACGPGRGYGRRRHPKKLTPLAYKQFIPNVAEKTLGASGRYEGKITRNSERFKELTPNYNPDIIFKDEENTGADRLMTQRCKDKLNSLAISVMNHWPGVKLRVTEGWDEDGHHFEESLHYEGRAVDITTSDRDKSKYGTLSRLAVEAGFDWVYYESKAHIHCSVKAENSVAAKSGG CFPGSALVSLQDGGQKAVKDLNPGDKVLAADSAGNLVFSDFIMFTDRDSTTRRVFYVIETQEPVEKITLTAAHLLFVLDNSTEDLHTMTAAYASSVRAGQKVMVVDDSGQLKSVIVQRIYTEEQRGSFAPVTAHGTIVVDRILASCYAVIEDQGLAHLAFAPARLYYYVSSFLFPQNSSSRSNATLQQEGVHWYSRLLYQMGTWLLDSNMLHPLGMSVNSS |
| Dr | shhb |  | hedge hog |  | Q6DBX7 |  |  | GLA-CG | MDVRLHLKQFALLCFISLLLTPCGLACGPGRGYGKRRHPKKLTPLAYKQFIPNVAEKTLGASGKYEGKITRNSERFKELIPNYNPDIIFKDEENTNADRLMTKRCKDKLNSLAISVMNHWPGVKLRVTEGWDEDGRHLEESLHYEGRAVDITTSDRDKSKYGMLSRLAVEAGFDWVYYESKAHIHCSVKAENSVAAKSGG CFPGSGTVTLGDGTRKPIKDLKVGDRVLAADEKGNVLISDFIMFIDHDPTTRRQFIVIETSEPFTKLTLTAAHLVFVGNSSAASGITATFASNVKPGDTVLVWEDTCESLKSVTVKRIYTEEHEGSFAPVTAHGTIIVDQVLASCYAVIENHKWAHWAFAPVRLCHKLMTWLFPARESNVNFQEDGIHWYSNMLFHIGSWLLDRDSFHPLGILHLS |
| Dr | dhh |  | hedge hog |  | Q4PLX3 |  |  | VDG-RG | MTLAPWLRLARLGLLTVCLYSWLVVDGRGPGPGYGGRHRQRKLTPMSYKQYVPGVSENNLGASGRAEGRITRSSERFNELVCNYNTDIDFKDEERSNADRFMTKRCKDCLNKLAIAVMNQWPGVRLRVTEAWDEDGHHPPGSLHYEGRAVDITTSDRDTKKYGLLAQLAVEAGFDWVHYESKYHVHCSVKADHSVAVEKGGCFSASGLVTMADGVQKPMSCLWPGEKVLSVSGSGEVVFSRVLLFLHLDRESRTSFFIITTENEKRIALTPNHLIFAAHNLKLHHHDYETVFARNVRIGDYILTTGGDRGIQPSKVVSVSLEERMGVYAPLTEHGNLFVDGVLASNYATFQDHGLAHTVFWPFRVLFVFFNKEMEEDLQRVAVPYICSTNQTILTSVMHSRLSSVFKWQDATRAEMENAFLQQKEVYWYARLLHTLGRIFLDPQRFY |
| Dr | ihha |  | hedge hog |  | Q4PLX4 |  |  | NEG-CG | MRLPVVFGLLVGCALIFAPVNEGCGPGRGHGKRRPPKKLTPLNYKQFSPNVAEKTLGASGRIEGKITRNSERFKELTPNYNPDIIFKDEENTGADRLMTQRCKDKLNSLAISVMNMWPGVKLRVTEGWDEDGNHFEDSLHYEGRAVDITTSDRDRNKYGMLARLAVEAGFDWVYYESKAHIHCSVKSEHSVAAKTGG CFPASALVTVEDGSLKTLDSLQPGEKVLASSESDGSGTLVYSEVIAFLDRDPSARKQFFTIETDSGAKLSLTAAHLLFVSEGNCSGSAANAELRSVFASDVLPGQCVVSTQAAGQHGRLSRVSRIQMQEDRGVFAPLTSHGTVVVNGIVSSCYAAVDQHWLAHWAFGPLRVLYNWGGPVGHQVTGIHWYSSLLHWIGTQVLDPAHFHPWSMMDNDR |
| Dr | ihhb | Echidna | hedge hog |  | Q98862 |  |  | SPA-YD | MRLSTAAALLTGFILAFSPAYDGCGPGRGYGKRRTPRKLTPLAYKQFSPNVAEKTLGASGRYEGKVTPSSERFKELTPNYNPDIIFKDEENTGADRMMTQRCKDKLNSLAISVMNLWPGVRLRVTEGWDEDGLHSEESLHYEGRAVDITTSDRDRNKYRMLARLAVEAGFDWVYYESKGHVHCSVKSEHSVAAKTGG CFPGRALVTMKDGSHRQIRDLQAGDLVLASEGSDGTGDLIYSEVLTFLDRRPITQKHFYVIRTEDGASVSLTAAHLLFMRVGNCSNRGEPKPGAVRTIFASDAQVGQCLLLGKLRKRFSQITHVGVREDQGLYPPLTAHGTVVVNDVLTSCYAAVNRQRLAHWAFAPLRLLYSWTGPDQVLKNGLHWYSQVLIGLGKLLLDSELFHPLALEATER |
| Nv | 241466hh |  | hedge hog | has EST covering Hog domain; Stellabase: 14141 |  |  | 2672324\_2 | ANS-CS | MRLHLFLFYPLAFALVLFQFSLDFANSCSSRPSLSRRGRRPLYFKQRVPDVDEFSLGASGRPQGKITRNSSKFNKLVACYNTDIVFKDEERTGADRLMSKRCREKLRNLATKVKQKWKGVKLRVTEAWDEDGQHSLDSLHYEGRAVDISTSDKDPKKLPDLGSLAVDAGFDWVYYDRRSSIHASVRSDDDRKDSWGS CFHSEALVTIENGERIAIKDLKTGQRVQSMDETGRLLYSEVLIFLDYKPWLSKVPFTIIETDVANLALTRNHLIFVMKRNSSTIYDASAKLAQFVTPGDYVLVNSKGKLHPSRVMSVRIEHKLGAVAPLTAQGTIIVDGVVASCYSEVTSHTISHLAFSPLRGLRYWLPSVFSWLHEGITPAGVHWFPRFLISLNQIVRIAEFA |
| Nv | 95413hh |  | hedge hog | N-terminus missing in prediction; Stellabase: 9851 |  |  |  | AGT-CG | MGQQFPCLLSCGIALFIFQLLIHGAGTCGPGKAAFDRDNRAIPMKIREHIPDTSETSLQASGPSRKIKRGSNGYKELITNADPNIVFREDKAGNNRRMSKRCERKLKILSSLVRKEWIGDVKVRVIRAYDDGTSKKRHHGPHSLHFSGRALDITTSDEKRDKLPMLGRLAYRAGFDWVYRAKAYIHASVKSDDEDDYRENTG CFPAASTARLATGEQVPMVDLRLGDKVASVDDRGGIIYSPVIMFLHRSPELIMDFLKIRTENQTELVITASHLIYKINRQSKRKEACFAKDVTVGDLVFVGGRSSTKSLSPGRVTSVERTRSRGVFAPLTQAGNLFVDDILVSCYAITSSDSIAHWSLAPVRLVGAICPRCFDIEYSGIHWYPRILLTIFGKIVELCGGFL |
| Nv | 120428 | 9703 | Y hog | Predictions cover only Hog domain, EST covers N-terminus; Stellabase: 11525, 11524, 2031; cDNA 327-384-29\_H09\_T7 |  |  | 2674792\_2 | AFA-RN | MRGSMEVAAVFLAAWANVAFARNLPPFSALKSSYPGHASQGGLYTDDNVMLSVKAENRTINNTCALRISIMLNFIPFHQISSLPKGLNDSQYHEMIITSAQFNTSMYFMLDNEALMAHMNRVYGQPLVSSRVEDFKNKLGIMLISMSDGRDNQCGAVLWDGYGFFQARAFLRYTNMSAIYFWQTPDEPCSIVQGEKSESLL CFPSAAQVELDSGERVAMSELRIGDAVKTTDKQGEAFYTKVVTFLQREPSHLAKYYTLMTESGAQVTMTESHLIFTKNLQVSSNGLHRRDAVYAARVRPGDYVYVQTPGEDTTHAEKVVGVALGREVGAFAPVTAEGTMLVNGVLVSCFADISDHDLANSLMSPLRSFYSMAPHYLGSGGTYLHKYLKVVLRPVGIRVFGEEKFYKGPFEEYAKKN |
| Nv | 140260 |  | X hog | N-terminus manually fixed, has ESTs; Stellabase: 23561 |  | 327-384-32\_O04\_KS, 327-384-29\_H09\_T7 | DV090341, DV089255 | CRA-KP | MGSWLLLAVFVAAVFIDKGSCRAKPEVTWYDQPKVSNIKLPYLQGMSINATADKQVPQHDTTTSMPPTPLKTITDVINERKLLKSEDSSAVANKSHEVPVIPMKRPSVY CFPESSTVQLSDNHRIPMKELKIGDRVKTLDARGYHGYSDVIGFLHRVDGHVIDYLSIKLANGKHLRVSDKHLVFKGKMDSRRFEEIFASQVKEGDCLVTEEGSENSKGVRLSRVLQVTMTTGKGVYAPLTRDGTMLVDGILVSCYAHWDSHQVAHAAVWPLRAWANVKAAFGSFIGWFPVSQPVSGIHWYAESLISMVQMFSQLK |
| Nv | 87496 |  | hog | scaffold\_16:50982-71258, Hog domain 1, 87496 prediction just Hog, lacks C-term.; manual prediction from scaffold. |  |  |  |  | KRCFPGNGKVELESGVQMYIRDLRIGDRIKTVGSSGDVIYSDVIAFLHKNTTIVVEFVALHLTGEHRIIISAKHLIFVSKNGNSTSEAIFADRVHVDDTVYVLEGGKLVVKKVVRVAMVTESGIYAPLTREGTLVVNGVFASCYAHWESHQIAHGVMLPLRAWYDLWNFFGHSFSVFDMQNSVTGNEIHWYAYALMKARFALPNNLASFLGA |
| Nv | 239508 |  | p-rich hog | scaffold\_16:50982-71258; confirmed by EST JGI\_CAGN20453; Manual reassembled from 239508 & 87421; Two Hogs, duplicated cluster, transposon insertion? Stellabase predication parts: 28512, 15895, 4947, 42335 |  |  | JGI\_CAGN20453 | LES-HP | MSLHYSSLFWITLFWRFLESHPFKFNFRVKAPYFFKSNKQAVIMSIVLVEGRNQCLQSFLVFALFFIHSVAAKPSAEWWSYGPKGSYSVEELGTLPPTTRSTPVKPAVAPSPPPPGIPVGSKPGAGLPVAPQTGAGKPVAPPGTTIRIAVTVNRTGQPEIHPEVHPGPLPPPPAATARPGNQASTANPLSTPVSTTSTKATQGLLPSSTPEMPETAKPTTVVTQVTTMPNTSVTPLTSPSTSVTQRTRPTTPVTQEATPSQTSSASTSHGKEEDQTIETG KRCFPGNGKVELESGVQMYIRDLRIGDRIKTVGSSGDVIYSDVIAFLHKNTTIVVEFVALHLTGEHRIIISAKHLIFVSKNGNSTSEAIFADRVHVDDTVYVLEGGKLVVKKVVRVAMVTESGIYAPLTREGTLVVNGVFASCYAHWESHQIAHGVMLPLRAWYDLWNFFGHSFSVFDMQNSVTGNEIHWYAYALMKARFALPNNLASFLGA |
| Nv | 200640 |  | hedge VWA | scaffold\_23:25000-60000; Stellabase: 13976 but wrong. EST just upstream limits N-terminus |  |  |  | TLAVA-LP, TLA-VA | MGLLAIALILQLCVASFGTLAVALPSSLSQGSSYPYSKTEVSVCGGIRDVILRNSARFRKILVRNADTEVVFENDDCRRTTARAKSKLDVLASRVRQEWAGRKLKVIKAWTDQRTAQDPASLHYEGRALRLQLDNNDRSMLSRLAGLALASGFDWVSYPLNSDYIHASVIRDVCQTSVDLVFILDTSGSVGSYNFEKMKTFVKNVVDFFNIGPKGTHVAVITYSTWAQVEFNLKAHHSSKAALKNAVNAIYYRSGWTYTADALDLAGRNIFQVANGMRPDKGIPKIAVLLTDGYSNGNNPLGPANDLRAAGVNVFCVGIGNYYERELNDIATDPDKDHVFKLENFNDLNSWVDTLSAVSCDEGAMIRACDDTISTVEAGSFLYFRTEFASANDGISVEVKDISGVSHLYVSLSSKNPGPLDPQSYKNESSISPRSLRLNFPSRGSKLVFVAVQGQHSSNSFKLSMWDTLFAKDSYSASVKEELQATQNVITVSSSVSPNLRYSIMDGNDQGMFTIDSSTGSITTTGRKFDRESESRYRLTVLAQDAANRCHKGRTVVVIDVKDENDNAPEFPLNSYVFNIRENTPAAQVAIVTATDRDSGTNAAITYSLFSGENKFMMNSKTGEIRTVTPLDHETKDSYTMRISAFDGKHTTFVPLVINILDVNEPPRFQDDCTRRGDCSATVLESAPLNTRIVTLAATDPDGDSLLYSVRIRDSQEKIFTIDNSGRLTLNKMLDREAKSAYDLVVEASDGNIIAIAALKITVDDVNDNAPFFALSVYKTGVYEDVAVGSIVRQLTAVDLDVGINARLSYSLVSGDTSYFTVDSQGVIRTKGAFDRETKASYQVALRAQDGGSPSRSGQATVEIEILDVNDNRPVFSSAQYTASVDEDVAIGAAMVTVTASDKDNGNNADLRYSFTSGNTNHAFTLDAVTGVVTVSRSLDFEQRSSYALGLSVTDRGSPPLTDTSHLLINVNDINDNPPVFSPSAYQSRVKENTPAGTQVSATDRDSSTNGALVFGIIKGNDDGVFTIDGSTGIISIAKSPDYENKTRYDLTVQARDRGVPVKYDTAQVTIFIEDVNDHSPVFSPSNYSKQIPESTVIGSTVVTVTATDLDSGPRGRLEYRLISGNVKGAFNIDANSELDRETTPSYSLQVEARDGGSTSRSSSTRVTIALQDINDNSPIFQGPYVFRVSESSSPGHQIGQVAATDADDSSNGDVRYALSDNTDVFSINTSTGLITLKTQLDYEKKTQYMVTVTAKDGGTPSRSSLTVVKVMVDDVNDNPPQFAKSLYTCTVGENLAAGVAVCYVTATDADSGANGKISYSILSGNENMAFAMDRFTGEITTRVPLDRETIASYSLTINADDGSLVRFMAATTVNISIADENDNQPTFTGPSRFNISEDSPTGTRVGQLTATDRDIGPNGAVTYAIISGNQGNSFQLDPRSGILSVRSTLDRETIPSYTLVVKASDAGSPQQSITASIHVSVLDVNDNAPRFEKSLFSGEIREDASVHSTVLQVKVEDKDEGSNGAITLSLSGEGSDNFTIDSTGFVLSKTPLDYETTAAYQLTVTARDGGSPSKSATAQVKITIINVNDNVPSFVSPAQVTSIPEDVAINTHVVKLNATDSDGTPLTFDIVEGNAGSSFKIDQSSGLISVAKRLDRETTANYTLVVRATETGSQGESVYNNATIRVLDVNDNAPVFNPGSYAKSLHENLPIGQTVAKVTATDRDEGENAKVTYELSVGDTSKFEVNPATGLVTTKRPLDREDQASYSLRVTAMDHGKPSQSAVAAITLTINDLNDNSPQFSQSKYTLTVTENTANGSNILRVLASDPDAGANGRVTYSIISGNHGNAFRIDSTTGRITVVGVVDREALASYNLTISAKDSGVPPWVSTAFVAITVADENDNSPIFASNETSYGIDEGVSIGSRVAVVTATDRDEGSNAQVFYSIVRGDTSAFAINAMNGVLTVKKEINRETVPTYTLTIRASDRGSPMMTSDKELTVVVNDVNDNPPVFNSQSYIGSVRENSAQSTSVLTVAADDSDVGANAVLRYSIISGNDEKRFKINSTSGVIMTTTPLDFEEKSQYGLEVTATDSKYTAKTNVTIRVINLNDITPAFTQQNYTASVRENSPTSTSVVKVSASDRDSFGSLSYSIVGGKDSDKFSIDQRSGIISTADVLDRELVSLYAIQVRVTDAGNPPLSNLTWVTIKILDENDNSPKFDSSSYSSSILENSAPSSFSFKLAATDADEGTNARITYNLSRNDAFMIGASSGVITNVRKLDREQVSIYTLYVQAVDQGNPRLASEWVPVHIRVQDENDNAPRFVSSGNCNVSENAVVGAIIAELLAVDPDLGTGGVVSYRILTRQTEFTIDNTTGVITLAQPLDRENKSTYDLAIQARDHGTPPQASSITIRVTVIDINDHRPLINSSSLRGSVRENQPKETHVMRILASDADTGLNAELTFSLLSNEHKSVFTLDNTTGDISTKVPLDREQQAVYTLRIGVADKGNPSLSSVADVIIDVIDEDDNCPRFQPTEYNVTISESLPRGASIVQVTARDIDGDKDTSYAIKSMTPSGGFTIDSKTGVVMVASSGVDREIADVFKLVIRAGKEHCGATNDTGGGEGGRFLGVQQATYPDSLATVTIQVTDVNDNAPSFSRDSYEYDFNDVTTVNLLSVRATDLDAGVNGVVKFRLAEQTELGGNRVVKVEAYDQGSPSLSSQVSVIVQTSVSCEAMVFSITEDGAISAKTICSFDVPPRSGTVVAGNPWTLKCSAKGNTSPQYRWMRNGLYLNSFSNQSDYVIAKATREDAGVYACLATSEAGLIQSSAATLIVNGKPSRSSPPIPYACLDTSEAGLIQSSAATLIVNGKPSRSSPPIPYACLDTSEAGLIQSSAATSIVNGKPSRSSPPIPYACLDTSEAGLIQSSAVTLIVNVKPEITHHPEDSSVELGGTATLECRASGVPEPSYRWFKNNEVIPSVGGIQSDRPVLVLRDVIAQDESPYYCQASNDAGTAKSNIATLKVYGKARLGVPLRGGESLVALEVSVSNSNRAKKCNIFDIQAFKAALVKAIEAKVTIANYTAERMCEANPCNSNPCMHGGVCELHSSVRYTCLCPQGWYGDNCQFNLNECKGDPCFNNGTCQDRNGTYQCTCRPGSTGKQCELTEGACAKSNCTADELCVLSESSLTGYECKNNDHVISMAHDRDIHGDQTKIYDFEREIQGMIQSAPDVNNASSVTGRFRRSARRNTNDYGACVVRIITPLQPGNVRFVLLCPIAVEPQSTRAWVCGLLFNAGKARACGQSESMATPVPATTLAPVEPTRVVIYLFARDDKGRNLPAEEAIDLFNNDDFDIAMKQQGVKFVAAKRVKPMAQTAPKETNTGLIAGLVVGIISLCLVAVAGAWYYTRKRKGSRHVQLKDRAQMNRSSCKKSSVERNVSVKDITRSEKRFINQAYTDSVYDDEAAFMVEVDLGKKRTSKPARKNGAAKIEHAPWYYGNMNTTEAEELLEVHALQPGTFILHDGIATDEYTLTVKAPEGGPPYRHLTIKLTDTGTVTAQFGTMISRMEFGDVDDVIRHFQVTPIEFGETFPDVVLAHGWNKTLV |
| Acm | hh |  | hedge hog | B035-A4, B037-B9 clones | DY581614 | B035-A4, B037-B9 | DY581614, DY581885 | SLS-CG | MAIAKSNILCQLIALFVAAQLRCSLSCGSRTGHGARNPGSPLMQYQCVPDLSENSQGASGPAKGKITRNSPEFEKLEPCYNTAIIFKDEEGTGADRLMSKRCKEKLIELASLVKNQWPSLKLVVTEAWDEQGQHSKNSLHYEGRAVDLRLSDTYKSNPKLALLGRLAVNAGFDYVLYESKTHIHASVREDSYVDKTKRTGCFSSESTVRLENGAVLRVDQLKISDRVQVMMQDGTIGYSEVIMFADYLPNI |
| Acm | DY579185 |  | Y hog | A050-B6 clone |  | A050-B6 | DY579185 |  | ARGTTCVLRMSTALTLNPGHQVREPYEGSVEGTANNYYFYDQRGFLSYLEKMYGFPISSTTTDTFRGIPGIMFINVTGTIRSDDVCSILLWDGAGFHQGKGYLHHYAIEKVFLWPAPSGGCDINLNVKQS CFPSTSKVELKSGKKIAMNKLEIGDLVKTYSNDGEIVFSPVITFLDQDIDYKGHYYTISTACGFKITLSEAHLIFKITQTPDAIKSDFAHSSLVRPGDHIAVHSRYGRFHEQVTSVSVAERQGAFAPVTEEGTMLVDNVWVSCYADIADHDLAHTLMTPLKRLY |
| Hm | CO905822 |  | hog | tah92d08, genomic traces, N-terminus ? |  | tah92d08.x2 | CO905822 | RWG-FY | MPVNSNYPILLWKLLLLLIFQPRWGFYIVMSANLGGSVELRKIHSNQIDKEAIKDQLKYGLKILKDGFQKSQDCGDIFDFNLSLTDIELNWIGGVCEPQISHLKNIQTIGSSSPFKSWKASLTFKPSVLKTKLKLQSIADFVSRINKKKGETCQTALEHMLGISKRKQSSIKRNTSSIAVTPDTRISAANDAEPDNNS CFSGNGKVIVMKSYPVTKLIKDINVGDKVLSFETKTKKFIYSTVYMLAHKNDTKKTIFLKISCKNGSFVTLSSKHLVYTDCYKVKHADQIKIGDKIWTTSIDDSEKMNLCKVDSIKITKSVGFYAPLTMSGNIIVDGVLSSCYANVKDVSLPGFGRISGQVIAHFGTAPLRVACLVFRKKFQTNEEMPKYIIALNQLGKRANLVSKP |
| Hm | CO9para |  | hog | close paralog to CO905822 |  |  |  |  | QWGHYVVTSAYLGGSIEIKKIFTNKVEQDLVEAQLKCNLNVLENGFQNSLDCGTILGLDPSLTDIELNWIGGVSTTQVSNLKYIETIDSSNPFKIWKASLSSKPSVLKTKMKLQSIADFVSSIDKRKGETCQIAFKHLLGMSKTRSFIRINTNNSAKAPDTRDRAANDAEPDNNS CFSGNGKIIVMKSYPEIKLIKELNVGDKVLSFESKTKKFIYSTVYMFAHKNDTKKTSFLKISCKNGNSVTLSPKHLVYTDCYKVKHADQIKIGDGIWTTCANDSKSMNLCKVDAIEITES |
| Os | hhlike |  | hedge ? | EST G840P38RM19.T0 |  |  | EB741380 |  | GRVRVSEAWEEPTSTDPAGIHPLASLHYEGRSARLDISIDILPGISVPDRDTDVSLLTRLGGLAVCAGFDHVYHSPDGHLTVSVRQQKDRHGNIIVYPSGATFLQVEIPTGYENDYTISGKEASLLVDSDGRLEDELSGHFKVKDVLSLHSDTGKAFRYFRLSPGVLYCLESVYNEVEEGRLEIVSAYRTKTDNDGRRL |
| Mo | hoglet |  | CBD T hog | blast 4e-10 to hh | ABA55664 |  |  | VLA-TP | MAPCGIVAALLVLSASAVLATPPACSDVTYTQTARWQGGFVGTFTINNAGTGFQFEWDFSDFGYPVTLASGDVWNANLDSYSSDHATVTLDADQNSFGFVAGTEAPPGAVRAADVGGFSIAGTPCGGVATTPAPTACSNLNYQVTASWEDGFVIRVDLQGGAQNSIIEWTWDSSVSIQGSVWGADVLSSSGNTVKFGVAGESFGMLLSGSSTTINSIQINGNPCTGGGSTTTSTSSTTTTTTTTSSTTSTTTTTTTSTTTTTTTTTTTTTTTTTTTTTTTTTTTTGTTTTTTTGSTTTTTTTTGSTTTTTTTTTTSTTTTTTTTTTTTTTTTTTTTTTTTTTTTTTTTTTTTTTTTTTTTTTTTTTTTTTTTTTTTTTTTTTTTTTTTTTTTTTTTTTTTTTTTTTTTTTTTTTTTTTTTTTTTTTTTTTTTTTTTTTTTTTTTPTTTFGCFSQSSTVVVEGRGRISITDVQPGDMLVDGSAAGTSQFVSFMHVGEQLAPFVAIQAGNNTIKLTALHLLYVVDAKQGAELVKASEVRVGNTVILADGEHAIVDAVSIVEEEGFISVLTASGQLAVDGIVASSYTASKVYPGEPISLRHALNQPLIWLAQAAPSLAAPMLSSEYHWYQQVTRAPRAMAHAFRSIAKAVEARMPALATVSCTGSL |
| fGm | GmGIN1 |  | AIG1 hog | clone GM1132; blast 7e-18 to hh | AJ315709 |  |  | - | MSNCPSILLIGKTGVGKSTLGNLLLGRDVFDVSDSAASLTQGYQTAPIEINEKTFNVVDTHGFFDTNRTNQEILKEVTQEILQCENGIQAFVFVIEATRFTKGQRDTINQIINFLGEDSLNNMIAVFSKCRKAPTINPDRRLFNSFSQEEKDFLNRIGNRFTISPNLEIFDEPNDPIVVRHMTKLKEYIVNFPDLYTTAVFEKVLMARENEYKRKRVNAGLKGFLTRKPVELINDFEG CFAADSKVILKNGKVTKISELVIGDYVCCGFEDGKQVYSEVFLMIHADPNAVTKFQSIDFVKQDGSQGNLHITPKHHIFVNNGETDFANNVTTNTKLFVSDGEKFVTVLPIRVTKERRKGYYSPLTRSGTILVDEVLCSCYASAPPYQALLNFVLVPLRMYTKIFPSNYLDKEIHPYVKFLYKGRWIMGCL\* |
| fGi | Hog |  | Hog | clone MtBC40C12, blast best match to Glomus mossae Hog, this EST may be of fungal origin |  |  | AL387088 |  | REAAEERYMKSLEMMRQEHRDQQERAETMFNQRIQDEERRRERDEEARREAENTYRRRIEESEKNLKNRENNETMRLMKEMQDREKKANLDFAKQIEDLKKKNTLSQQELEALKKKKG CFSLDTKVQLANGKFVEMAELQVGDRICSNVRNGELEFSEVYLISH |
| aKm | Hog |  | some hog | clones KME00015150, KME00013914, KME00002051, KME00006039; cluster KML00001775 |  |  | EC158739, EC162269, EC156565, EC155085 |  | TIGTLLQGLHQNHHRDVVSFSEFLTPRIQTGFAKTAAQAGQDNFKGANDNLEEKIGIKEAGKLKYSLRTGRTVYLCGDNADCCKESCKKLAHPNPRRDESRASQGSAELASLGATDNNEQADCDKFCTAEFSLL CFPGDSTVVVRDRGRVPLAELKVGDAVLSVRRRHAPTKEVDEISCDGWELYFDEVLAFLHSDASMEAEFLQVRHEAGQLHLTPNHLLFARSRTASASSAPAPVFARDVCAGDHLLAPWIDGSFSEPEVLEVTKVRRRGVYAPLLESGAFIVDGTVVSCYAIPDNITESLVFRKLAQITSARSMQAAYHALFLPLRVACRLRCTLPISD |
| aAc | Hog |  | Hog | clone Ac6884 |  |  | CF067845 |  | LQDLRVGDRILGLDHSTGRQSFSEVRGWLHRNPMQTMSMMELHTEDGLAVVTSPGHMLALSNDSSAIDGREALSYRRASDIRPGDTLVSANGSSIAVEETMSTKMEGLFAPLTMHSNYFVGSGEVDASKLVLAHSFANIADPQRYERFWHGLLSVIQTIAPXFNXTRWKLTATTCIP\* |
| aAt | Hog |  | some hog | clones UI-D-GC1-aam-p-24-0-UI, UI-D-GC1-aax-l-16-0-UI  hedgehog [Aedes aegypti] 61.6 3e-08 |  |  | CK784493, CK432388 |  | SDAVGQWELLXDTVINWLHHSPDREGEVLQIRHEAGQVQLTASHLLXVRKPGRAGAVPCRADEVRLGDRLLVPWIDGSIAEPAVLRIDHTCKRGLYAPLVSCGTVFVDGTAASCYGLPDRLHASPLVQRVARLADGKGFHAAAHAVFLPLRLLHAACGERGGGPRHAKAAALPGAGEERKQLEPSSRKEAGAYPPLCLGSVHPGILLPDLSAGFRVVAVA |
| aAt | Hog2 |  | hog? | clone UI-D-GC1-aal-d-21-0-UI, C-terminus finishes too early? |  |  | CK432474 |  | GTRNYELEITYSCSAYDATDKVCKGWTQRGTMSSAPA CFPANATVITRGGPKPMSKVALGEELMGLDHRTGLVTFSPVRAWLHRDTASEVAMVRIRTDAGDFIASPFHSIASGEDGAYTFATGLKPGDAVVTAKGPATVQSISAGCRARMPHSYPSGCLGCLYLCDVGCQ |
| aAt | Hog3 |  | hog | clone UI-D-GC1-abd-g-12-0-UI |  |  | CK785366 |  | HEGRAGLRGAHAPLTATGTLLVDGVLCSCYAPPAAWAVPHEACHAALLPLRLLDSLRAAAERWSRPAGAKEPLLTVEAVWLLPRSADETVHPYASGLLRTAQAAKAAAACCRALLLSCRLAAVAEPGPAASAARPPAAAAAAAVR |
| aCp | Hog |  | Hog | full length | XP\_628672 |  |  | VEL-FW | MKVNNIKIICFLWLTILVELFWFNKESRKNVDLFHSNLSFIRVRTKSIFDTFSGMSAGGTGFRSGWGNVISNAISLAFPSSCVGQLINIGYLSNELDEDDYPYNPNCGAQSQSNNL CFPGNSLVITRERGEIKLEDLRIGEYVLIRDLNTMKFKYSKVEIMLHKDKNIYLDDEWIQVEYLGMEKPLVLSPNHLIFIQYLGEKPHEGNCYYPSQIEKAIGIEVTPQLEKKKKLTSIQAKDIRVGDSVIIYSKERVGWVTQVSIISNVNSNQKNYEYVGRYAPLTTDGYLIVNGVLVSSYSKPFPWPMELLNPSHNLIDLLARPIVNIEIHFKQIFDYTRNGVVKILRYISNISSKYTFDQSIALSLEIIRAISLFVK\* |
| aCm | Hog |  | Hog | genomic shotgun ctg\_1104857754489, full length, C-terminus too long? | AAZY01000565 |  |  | IFP-KL | MEFQKFLLIYYIYLWEIIYRIFPKLIIDQQIIINYSFVRLKVKGILSSLQDFDSQSFRSDWGNVISASLQVSFPTSCIGDIINMGYVSTELDEDDYGFNTNCYANSETNNL CFPGENIVLSKTRGYIPIKELKIGEHILTFDYKSLKTKYSEVIMMLHNDPNFYPDDNWIIIKYLNVDIPLILSPNHLIFKLDLGDFPQKGDCEINTTIFSHFHNIYINNKIDHFKVVSVLAKDIRIGDALIINSINGIIVSWITDINIYNVESQKYLQLKGRYSPLTKDGHLIVNNVFVSSFSKPYQWPVNIIQPNHLFLFKILKPFIFLQIYIYKISYIRTLIQYIIKFVSYFSNYKYDNSIPEILQLLRIFLAPISKKKYYLTIAYIIKLYLVIFLISNIFTMIFINKIYYNKYSLNYHNC |
| Xx | 104K18 |  | Hog | clone OSIGCRA104K18 | CT850429, CT837019 |  |  |  | MCCCCAAWCAAKCACCACDCLKKLLCVDKEALAIMSDGKLKPLAELQIGDKIKTLDSAGNMVDTEVIMFAHVGNDQKIMYNIITWSGKSIRASPNHLIPVASSNEYKYAKNVTEQDMINTLDLDTKRMCAEQVRAIALEECTGYVAPITMSGDFLANGVLVSCYAEIESQSLAHAAFEPIRWWHSMMNSMAPEFLAVKLKSEKQSEGIHWYPASLFVMAGSLIDILTAKKKETVETEETTGLVAAESEDHLLFLMA\* |
| Xx | 123G06 |  |  | clone OSIGCRN123G06; similar to OSIGCRA104K18 but frameshifts, N-terminus looks wrong. |  |  | CT855150 |  |  |
| rCc | Hog |  | Hog | clone cct14\_B07\_03; blast 1e-10 to hh |  |  | CO652162 |  | PPLPSAAASLLFQQEATSPAVCFPASARVQTADGRAPRLDALAVGDRVLVGVRRGRRVYSEVFAFTHADPHATHAFVELATRPRNHTLRLTEGHWLLADGAWMPARAVRVGMALQDGWTGEARRVSGVRVVGDSGLYSPQTYHGAVVVDGVVA |
| rCc | Hog2 |  | Hog | clone cct03\_E05\_09 |  |  | CO651485 |  | TSGGVPGESEPAASDDLSDLSPSPSPDDEDEDDDHSVCFPANALVELENGATKMMSQVQLGDRVRVGPNDFSDVFMFTHKTAAIKYSFVTLATQSGHTLSLTKGHYLYVNGVVAAAKTVRSGDFITLADGTTSSVTQVGTEIA |
| rPy | Hog |  | hog | clone PS012e10\_r PF006e09\_r |  |  | AV436888, AU186980 |  | SSPGESPSDGSGDGSGDGDGDADESPESTDDDSECFPATASVEVEGGATKAMADLAIGDRVRAADGSFSDVFLFTHADPTAKSTFVTLTTAAGVLTVTPGHYVLVNEGVRTAASAVVVGDVLSYMPPAGCAAAAAATPAVVTATGRVVAKGLYNPQTLVGSIVVDGFAAFTFTTXVE |
| rPy | Hog2 |  | Hog | clones PFL056a11\_r, PM028g03\_r; blast 2e-09 to hh |  |  | AU194160, AV433424, AV433921, AV433866 |  | TAIVQDGARCAGPGQFALVSPAVLENLDDLSGDFVDPLKEYLASEGLGSAFTGFVRTIFNKIVGAPVYMGVATEEDRTCGDREVEGRTFVAAMEVNESVTFAGETVLPEGRHLITYTIESNPLSQADNPLCLYSGSEVTGGAGGEGGGNGTIVDEPA CFPATATVELSTGATVAMADLAVGDRVRVAAGAGAAAFSPVYTFTHRSSGGAHPVVTATTRSGHALTVTPGHLVYINGRAAPLRSVRVGDALDVAADAASSVVTAVSTGTSAGLYNPQTLQGDIV.. |
| rPh | Hog |  | Hog | clone EST02698, ends in stop; blast 4e-07 to hh, linker seq removed |  |  | DN607487 |  | PTPVPKATGGDGGE CFPAATTVRRRYGGVVSTVRMDDLRLGDEVLAAGGVYTRVYLWSHADAAAVTTFVRLVATRADGGLHTLLISAGHLLPTVVRGGSGDRGPTLTAAVSLAVGDTLFAADGSPLVLTAVVPGVAAAGLYHPHTTAGNLVVDGVVASDLTTALPAALASAALAPLRAAGVAGWMAPARVASRLLRRGCAPVVRAVAAARWSVGT\* |
| rPh | Hog2 |  | Hog | clones EST01874, EST01629, EST02640 linker seq removed |  |  | DN606663, DN606418, DN607429 |  | ENLDGLTGPFEEIVRETLGAVDGAASTFSRFVRTVFSAITGAPVYLGVATQVDRTCGDREVEGRTLVAAMSVNQTVQFVGSLSLPAGRHLLTYTIESNPISQVDNPLCVYTGAAIKSGDVAEGANGTAADNNPACFPAAATVELSTGAVVAMADVAVGNPVPVAAGAGPAAFPPVYTFTHRTAGGAHPVVTATTRRGHSLTATPGHLFYFKGHATPMRSVRVGNPLNVAADPTSSVVTAVSVGTAAGLYNPQTLQ.. |
| rGj | Hog |  | hog | ends in stop; blast 3e-08 to hh | AY123134 |  |  |  | LGDRVHVGGGEFSPRVLFSHRDASTRHAFVQVSTACGRSVAATHGHYLYVNGRLAIAGSVKVGDRLQDAHGAPLDVTKVEVVTKTGLYNPQTLHGDIVVDSVRASTFTKAVEIKMAHALMAPLRMMYNAVGLSATFLDKGADTIMSMLPKGRTEL\* |
| rGc | Hog1 |  | Hog |  |  |  | DV963423, DV963204, DV965897, DV969707 |  | DEGTDGGTDEGTTTGGLPISGEQGGPEGDITNIEPPETPEEPTETPEDDDPV CFPADGTVLLEDGSVKRMDEVEIGDSVMVGEKQFSEVFMFSHRLASVKHRFVRMELANGLSIEATTGHYVYVNGRLLAASSVKVGDRMELVSGDRVAVSRVSVVKKSGLFNPQTLHGDIIVNGVRASTFTQAVQVCTASALMAPLRFAYALFGWSASFLNGGMESVANMLPDGAAAL\* |
| rGc | Hog2 |  | Hog |  |  |  | DV964883, DV965371, DV963453, DV965713 |  | MADLQPGQHVHHDEHGASSPVFFFTHRTAKPELWFYSISTASGHAVSMTAKHYLYADGRLTAAHAVQVGQMLRTKAGESAVTSVKRVRDTGLFAPHSMHGDLLVDGIVVSSYSRTIEPRLAHALLMPLRWLARATGSKEVLAGVFYEGGRGLERFLPKGQAQY\* |
| rGc | Hog3 |  | Hog |  |  |  | DV966958,DV964945 |  | EPLAPAETSSPTTQPSSAAETAVSIDDENETVEESPVGEGNGQSEDAAETGDDEDVDDDDDDV CFSASALVELFDGSRKRMDEVEVGDRILVGHEEYSDVFMFTHRSAHVPYQFVQLTTSSGKSMKVTRGHYVYLNGMLKMASTAKPGDTVAVGSGGHEEVVDVRRVWGKGLYNPQTIHGNIVVDDFVCSTYTHTIVPRVAHSLLAPLRLLHKLVHMLTATSQKGFFVDSRAI\* |
| rGc | Hog4 |  | Hog |  |  |  | DV967367, DV967437, DV967343, DV962121 |  | TKDEFSDVYMFSHRDVTAADPSYVRIVTAETSVTMTTGHYVPVMKNVTGRASTFVQAEWVQVGDLVQLASGQWAVVSDVFADEASGLFNPHTLAGTICVDGVVVSTFTRSVSADAAHALLLPIRALYWAGVVREKLAGKWFXAGG\* |
| rGc | Hog5 |  | Hog | c-terminal fragment |  |  | DV969015 |  | PQTVQGDIIVNGVLASTYTTAVEPTMAHALLAPLRAAYSRLGFCMTAFDAGADMLASYMPSGSVVN\* |
| rGc | Hog6 |  | Hog | c-terminal fragment |  |  | DV964087 |  | ANGSELLASRSHYVFAGGALKTAARVQLRERLRVFDARGERAETVVSKRLLLRRGLYNPQTASGALLLFAPRGRAAGVLCSAYTARVPPRAAHALLAPLRWLHAVRAALRHK\* |
| pSm | Hog |  | Hog | clones SmoC-1\_06\_L06\_T7, SmoC-1\_01\_I12\_T7; blast 1e-09 to hh. |  |  | DN839649, DN837769 |  | GIFLGFHSVEFSMAHTFFFATSLFAVLASVVLATNTS CFPGDATVQMYNGDLKLMRDLEVGDKVAVSKNVFSDIYAFGHKDADVISEFIQVHTASDMIELSEGHFIPVDAKGKLVYKRAKDLQVGETLWGSSQITELSKVEKLGLYNPFTLSGNIMVNNVEASSHSEWFLDSLFDAIGATEYLPHAYQAVLAPLRAIYNIVGKEIYAEGYAAMDSCVNFAEFGAKYGGSIALTVGIAGVATAMLATKA\* |
| pPp | Hog |  | Hog | JGI 232119, correction in Hog |  |  |  | LVG-AQ | MIRMLLPLAFLLVLTHQAGVLVGAQLAISVTTFGPPHADGTPNTFIGTGSQIASSLLGSTTKALMWGVVSSSDVNAVCDQIVSTGALTFSATLTATTGETETLQQTITHVGGCRAAADGSGTQAVMVDNNGRQSAVVCPNNAGSTGCGIYLTGPDVTALYNQGARRRLLARNLLARKLLQGDNCQTGAAKGALTGAITSMEACIPFLGPLYPFCMAGVIGVATAGGAAVGCAEANG CFPGDATVLLAGGEVKPMTSLALGDKVAVRRHDGGLDYEDIYAFGHKDALAAANYVQLSLKPVGANMSDPALESTKLELTPLHFTVILSGSEITYKRAQDVRVGDMMWAQASTHAAELSPYLVTDISTVEKQGLYNPFTLGGTIIVNGVAASAHSEWFLDGAFDALGLTHWIPSAYQMVLAPMRLAYYGLGKQAYLDLYLRLDALVDVAQFGTKFGGPVVTAAAGLGSSIIAALLIFKSSRPKTA\* |
| pSl | Hog |  |  | EST, clone: Sl\_SlB\_01O08, upstream sequence only |  |  | EH093067 |  | VPGIPRDLQATTPGLAQKGKPNTFIGTTGDQIASSLLASKTPAMLWGSVPASAITNICQKIVKTGGLQYTAKISGDWGGSESLTLKVTATGGCGAAPDSSGVQFVFTDNYGRKSAIVCPRDAGKSGCGVYMTGSDVDSIYNKKLELEQQTLLNSSDHEAARRILLGDCREEAGKGAIGGAITGVLGTGGCLGL |
| cBn | Hog |  | some Hog |  |  |  | DR038961 |  | GDFDIGIQGPQTMIWATSGSDAVAQHAANDRSAGMVDLSADGMVEDDGDDDGDDV CFSGSSMLTLKSGEKKAIKDITIGESVVVTAEDGSLTHGKVVFLPHTKGNNHKIRFLELTTNKASRIQLSRKHLILANDCGKDSKFELYHASDVTLAMCLSGVDGPEKVIQIGRFRGEGIYSVITDHSHGLIVVDGLRASSFAVNHGIVNAFYHI |
| jJl | Hog1 |  | some hog | clone JLE00003795, several more, C-terminus?, If N-terminus complete, then good signal peptide. Expect = 2e-05 |  |  | EC691609,EC691552, EC691093, EC690824, EC691431,EC690000,EC68974, EC689347, EC689862, EC689862 | VLS-QS | MGKSIILACAVVACLTAAVLSQSNNHIGCNINYSGSMDNGREFNCGDVVLSANDQIQGQGFADSIDGTFSVTLLINGTSVGTPRQIVLDFKPCSGDGCQTPNTGCQRIGIDISGNAILKVTSGSGSVSNVWMNVHPCGRSQGGGPG CFADGQTVQLSTGQSVLVETGKIGDRILTASASGAGAGYSPVTWIMNHLTTKEVLEFSLQARNGTTNASLQVTHFHNMLVEGKGEVQAQDVHQGDTMRVMVENCFVEALVTKIEKLSSQKYVRLVVTESGTIVVNGVLGSSLESSG |
| jJl | Hog2 |  | hog like | clones JLE00002362, JLE00001832, JLE00003705, similar to jJl Hog1 |  |  | EC691842, EC688472, EC688424 |  | LLVTINSSVRIPGIEIRRETKTVWQFVLQEVAGWALCVWLQPLLWWLFCWPFATLALPIRWCAHGHSKATISAGTKAAAALPPSSLDPRIEFACSPTRSCWVGGSASGTFAVLVGSGSSSGQVVGSETVNTNCNRCNLQSGCVQPPVDGNYDLVMSITGVVGSLSAVSVTATLEKCESGGVG CFADGQSVVLSTGESKSIELLQVGDRVLSSLPSSSCQLAFSPVTWIMKHSSPKDVLAFTLESDRGVTAT |
| jJl | Hog3 |  | hog like | clone JLE00003705, very similar to jJl Hog2, Hog lacking |  |  | EC688424 |  | SVRCENSRRRAVCVWLQALVWGVLWWACATVAVPIRGCARGHSKATISAGTKAAGAVPPSSLDPHIEFACSQTRSCWGGGSASGTFGVRVGSRSSSGQVGGSESVNTKCNRCYLQSGCVQP |
| crGt | Hog1 |  | hog | assembled 5 ESTs |  |  | EG727196, EG725553, EG718685, EG725977, EG721300 |  | GLSMLNGSAAGGNSSAGGQNSSSASGGSNDFVCSVANIKALHAAVQPKWDACKSNPNYEEPNKGAKRRLLEWVWEPKAGERMTAKPRYSPDHYRLAIKADRSRRHAAKQSDTSSELGSYISLFSSLAGNSSNSSASQGSANAGSASMSSCEKNALSGACEKLAACKDPVCFSYYNEPEIEQLCGMCSMGSQAWFG CFAEDSKVSVEGKGSVAINEIQIGDMVLSADASGAPQHSRVIFKHDHKDVSSVISISYEDRTLRLTPTHLIPKYSDKCGDSFCHLAKNVPASSIQAGDRIYVHSGEGFQAKIVSAVSKSAAKVRYLLTENDRIVVDGILASVFSTAAEKFETLPFHLLDRFLPGVLQSKAVAATLETILESPILQGFETIVNSLSTLAVPRNANSNALLAATPFKSL\* |
| crGt | Hog2 |  | hog | EST clone GTE00007792 |  |  | EG726097 |  | YRRTYFIHDHEKPSETLKISVGQEEIEMTPSHFLPIYSAECGEKFCQFAKLVPAASVKPGDFLYTQCGFEMVKGVSSSRSFVRYLLVEGGNLMVNGVLASVQSTAAGALETLPFRFLDTIFPGSLEAPAIKQALRTVLESPMLRSFETLIARTHALPITIAPLLVSSSSSS\* |
| hPh | Hog1 |  | Hog | clone: SigC25 | AB258488 |  |  |  | DDGTLIWQCENVTKFVCKPFEVDDDDDDDDDDDRRRRLGKDDDDDKKKKKDDDDDKKKDDDDDDDANNVEVDGDFGSGAKNLTDYEKCELAVADVCDRELNASAALSILEQSLLDNLGVAIDNVEYLPYDTSLSKKKNKKLREKAEKSCKRTCKNECRSAEVLLNEWVADTGLSS CFPAHAQVTVMRDGAQVASPISQVGVGDLVKTTDGFETIYFQGHASKDASPYVRLSLDSTHTLELSPDHYLRLVNLQCELETHVLAKDAAVGMRLAVSAEAEEACQTATVKQVERTVLAGAYNPYTTSGTIIVNGIAASCHSSWFLEGVTSAAATPLLYQQLLAPLRALYAVAPRLVKSFCAKFDGDNRPMSELGLRQIVGSIFTSVSA\* |
| hPh | Hog2 |  | Hog | clone ConC10 | AB258511 |  |  |  | DDDDSPVYDDDGTLIWQCENVTKFVCKPFEEDDNNDDDDDDDRRRRLGKDDDDDKKKKDDDDDKKKKNDDDDKKKKDDDDDEVEVDGDFGSGEKNLTDYEKCELAVADVCGRDLDANEALSILEQSLLDNLGVAIDNVAYLPYDENLSKKKNKKLQKKAEKTCRNVCKDECRSAEVLLNEWVADTGLSS CFPAHAQVTVMRDGAQVASPISQVGVGDLVKTTDGFETIYFQGHASKDASPYVRLSLDSKHTLELSPDHYLRLVNLQCELETHVLAKDAAVGMRLAVSAEAEEACQTATVKQVERTVLAGAYNPYTTSGTIIVNGIAASCHSSWFLEGVTSATATPLLYQQLLAPLRALYAVAPRLVKSFCANFDGDNRPMSELGLRQIVGSIVTSISA\* |
| hPh | Hog3 |  | Hog | clone contig4 | AB258487 |  |  |  | RSDSDGDSSGEDSSGEETSGEKTSGEGGSGSSLTPYDKCRRAVSSVCGRKFQLDEARDKLREALIDELGLAAIDLDSLPSELPYAIKEVIETERFVERCTEECTRQCRRIVVTILQWVNRRS CFPAHAQVSVLRDGAQQNVPISQVGVGDLVMARNGFEPIFFQGHASKDASPYVRLSLESKLTLELSPDHYLRLVNPHGELETHVLAKDAAVGMRLAVSAEAEAEVKTATVLQVERTVLAGAYNPYTTSGTIIVNGVEVSCHSSWFLEGVTSAAATPLLYQQLLAPLRALYSVAPGLVKSFCAKFDGDSRPMSELGLRQIVGSLANIASA\* |
| fGz | FG08272.1 |  | vWA novel hint | N-term Hint detected | XP\_388448 |  |  | - | MVASTVTSYIHIPTNTPQTVPLFRSLVSGSKSTKEEPIKSEDAQEPIAIISDKDATIRLEPVPSRNGLLIKIETPKEPSVNIPHVPCDIVLVIDVSGSMGQPAPVPGEDQESAGLSVLDLTKHAARTIIESMNENDRLSIVTFASKAKVLQPLLPMNQDNKTRAIKNVKSMEPRDATNLWQGMLEAIKQFNTDESSPNVPAIMILTDGMPNHMNPAVGFVPKIRNMGPLPASIHTFGFGYSLKSDLLKSIAEIGNGNYAFIPDAGMIGTVFVHAVANLQSTFATRAVLKLSYSKPLELLETTGPSVEQKPVEFADDSEDSVAELTLMLGNLQFGQSRDIFLRVNNTKELEFLNEQDPTRSVVGAYLTYVKPGNCIKSRKVLEAMMSKDADFIPMATAQRSVLEHSDIPPSEIAYHESRSIICNFLSTIFEIQPSGDRLVTRLIKQRHPDFKTLIETLPAKDFTDDKNKSLMQELSMEEPKGQIYLAIQKNRDFNRWGLHFLPSLCNAHTRQVCNSFKDPGPLQYSTESPLFISCRDSLDQIFDNLPVPEPSITHRSNGFFSASSVPSSMSAYRNSSGVCFAASTEVTLASGRIVQMRKLRRGMKVRTPRGSRRVALVLKTPVEQEVLCRVGDVLVTPWHPISSDSKRWDFPANEATAVVMYTGCIYSILLERDASVAAHAIRVGDMWGVTLGHGLTSGSDVRAHEFFGDYNVVGKSLLGLERRSNGVVVGNGVERDESGLVMGFRQIHLLFVPCKSPIPNLPRTSLGQLFPKLL |
| fNc | NCU05555.1 |  | vWA novel hint | N-term Hint not detected | XP\_960517 |  |  | - | MTAPITIPIVSAPSPSHSTSPSSSSEIRPVAPKLEIHPLPSHTSGLLLRVIPPRSPPNLPDPNFHHVPCDIVLAIDVSGSMSADAPVPTTASADYTNEQPEHNGLSVLDLVKHAARTIVSTLNSSDRLGIVTFSTEAKVLQPLMPMTALNKKKTERNLGGMQPFSATNLWGGIVEGLKLFDGQSGRMPALMVLTDGMPNHMCPAQGYVAKLRAMETLPAAIHTFGFGYSLRSGLLKSVAEIGGGGYSFIPDAGMIGTVFVHSVANLQSTFANNVVLRLTYPKYLGLEETTGESVDKVESVQLEKGDVDPDSSMQLTLNLSTLQYGQSRDIFLRYDSKAQEAIADGFDFESPPSVLATLDYQHFTNITNTVVSECNDIFRPNPQVKQLTPAQTAYHISRSALISFLFSLYTLRPDREHQPRFFKDSFATSLQTFLSTLTAAQPAFASDPHCRSLVQDLIGSTSAINDANQDGQVALALTSEDFYNRWGIHYLLSLADAHARQVCNSFKDPGPLMYGAESPLFVRCRDRLDAAFDSLPAPKPSRTTGFQGEISMRAYNNSGNPCFAGETNVRVGVAVAEDVAADSDQLVTKEIEISKLRRGMMVQTPKGFRKVRAVLKTPVCEERMCLVMARDDEARQLLVTPWHPVSLDGKDWAFPTDVALGDYVSYTGDIYSVLLSADSDVDAHAIMVEGVWAVTLGHGLTGTGEGVVTAAEDLRTHRFFGDYKLVLRSLERLQSSPDGLILGEGVDRDLQTGLVKGFRGKTSVHVDHHDD |
| fCg | CHGG\_08787 |  | vWA novel hint | N-term Hint not detected | XP\_001226714 |  |  | - | MVLTRIVSGCPSHEAGNAAMANGLTLAVGPKKPSNSLQLQLHPFSSEHERGGLIVKIQPPREPENADLHHVPCDLVLSIDISGSMADEAPAPSKPGGEAGEDTGLRVIDLVKHAARTIVATLDSRDRLGIVTFTNRSKVGIPPYENKAKTLENIESMEPFSSTNMWHGIRDGLSLFSEAEGGSTGRVPALLVLTDGMPNYMCPPKGYVPMLRSMEPLPATIHTFGFGYELRSGLLKSIAEVGGGNYSFIPDAGMLGTVFIHAVAHLQSTFANNAKLRLTYPSYLKLEEMTGEAVGRQEPVELEGDVPESMTSLTIPIDNIQYGQSRDICLRYGNLAAAIQKEGGANPPPAITAVLEYQHFSPTVHQAVAQQSPFASTPSLHPDEIAYHLSRAALIAFFATFSPLNVQYEHEPLGSLPTNSTKLLQSLVDSLPASPKFTTTIGGYAGCRSLLIDICGAPVPTTNPSSWTGQVALALLDPTFYRRWGRHYLASLAGAHARQVCNSFKDAGPLQYGVDSPLFQRCRDRLDKAFDSLPPPVPPVRASRRNGRNQNAHTVRAPVSMARYNNAGNGCFAGCSPVLLAGGKGLVRINRLRAGMEVVTPCGPRKVAAVLRMPVRRVEMCLVAAAAGPSGKGKSRLLVTPWHPVELHQGGKWVFPRDHAARSVRYTGAVYSVLLERDEEPDAHAILVGGVWGVTMGHGLTTQVLGRRYDVRVHEFYGAYDTVSKALARLPQRAGGVAMGAGLTRDPVTGKVIGFSAVKPEEVKALGMRQQGRGVFA |
| fMg | MGG\_09762 |  | vWA novel hint | N-term Hint not detected | XP\_364917 |  |  | - | MDVPSDSPQWTKVPPGENDLRVHVYPLASKDGILIKVKPPRVPKTNEPTPTDLVLVIDVSPSMQTEMVVPTEDENQVRERFGFTVLDLVGHACLTILETLTERDRLGIVMFKGRATVLQGLTLQDPQAKERSAKYLGDLRRLSEKWHCNMLGERDVMDGLQVGLQLFNEVRDHSLPYRVPAVMLVTDSHLDSTEYTKPVASLKETNPEKAQIHTFGFGYNSEAGVFKAFSEISGGRYTFIPDSSMIGTAFVHAMANIRNTAAFRVCLNIKGEDLKLVSPFVATLREEETTSSIPEPDTRNIYRIQLTLGSIHFGQSRDVYLAYERPPPEDCVIQAELVAFDAEGYFQTVEDCRADDYSHLSPAEVAYHVSRAQLCRFLSSLFPLDTSFDGLGGFLPKLEMADGRLAELKRTIPARRFPDDDDCASLMTDLEGAPPRGQISLALSRRDYRDRWGGHYLLGLLDAHTGQVSTSHKDEGPLRYGRDSPILKRCRDALNAAFDHIEPPRPSIDVICTPAPPPYRFPSVTLSSARSIADKRAAGEPVDACATVGSRFNQGSNPCFAGTTMVRLAGDGGVVPISWLRAGISVATPAGPRAVRTVLRTRVRSQPMVRLPGGVVVTPWHPVKMGGDGAEWMFPAAAAAASGRDDGSVSTITYSGFIYSLLLEAEALPDGTKQDPVSAAEQHAFCVGSGGVWAVAMGHGLVDGSDVRAHAFYGDRNACLGALATLPAPKNGVATSAGVLRDATGRVIGFRVPDGGGEVRVGTAAQGFDVVVRRSTV |
| aTt | 00471620 |  | vWA novel hint | N-term Hint detected | XP\_001033046 |  |  | - | MESEFVKPISHQLTVKESIKDNKLRISLIPPASIKRRTNSNICCVVDVSGSMGTEANSNSSVSSSENYCLSILDIVQHALKMIVNTLTPDDDLSLVVFSSMAIEVFDTLRMDDANKILAIDKIEKLEASGSTNLQHGIQVGLNILSKSKSQNRNQAMYVLTDGQPDDRNVMQFLKKYKKDNPQLRCTISTFGFGSSCDSELLDEIAREYNGMYSFIPDATLIATAFANALANTLTVFANYVQLRVKSLNSFKFNVPPQQPISSKSISKQQDVIIYGGVVNIQQNRDYILDILEMPQDENLPYAQVEVIYQESFRSDKQKNEKIEDKIVMATLNSREYEQDFDNHIYRYKVISAIDESIKSYKKNQQSSDIKNRLMNLIEKMEEIHDQNDRHFDGIYKDLVRTVSEAFKDAKTFTSWGEHYLTSLLRAHLIQQCNTFKDPGVQHYGKDTFKEIRAVADETFVKMPPPKPYSRANYYDQIQQASQTVQVSQQSNQSSSSYSSAYNMNDYHNYSSGGCMHGDSLVQLSDSSFKMVKEVKKGDKVICPLLENQCVEVECVVLSKCEDGTKEFVQLGTDLWITPKHPIRVNGEWKYPKELGQTVVKTSDYIYQFVLKTGHTMNIGGYECICLGHNFQERVAYHPYLGSQAVVEDLKQMKGWKEGKVIIRSRVRDQITNQVKAFIQ |
| aTt | 0320310 |  | vWA novel hint | N-term Hint detected | XP\_001012930 |  |  | - | MVLIESKFSRNNLNSQRNQTESQKNSSSDSESGNDGQATINLYGLENQVRIQILSPKGKSKVSNSICCVVDVSGSMGSRAVTKQSGGNSELGYSVLDIVKHSLNTIVQNLDEGDEFSMVTFSDNSKLVCNYQQMTESNIKSSVDLINQCQPDASTNIWAGIEQGLEQMQNDSNKNKNQQLIVLTDGQPNVNPPRGILTTLNNFYNKNIISPKPSINTFGFGYYLDSHLLFNIAQDCQGIYSFIPDSSFVGTIFTNSIASMQSTFATNAVLVFKPLNKNAQLNLSQIKSNFKTYLNKEGEYIVELGNLFFDQSKDIIFQQDLHSELLRDFSVEVKYYTKDTNNFQLSHRMHKAEQIHNANKLEFEQEALRLEVVKTVQQCKDSSQKSQKLVEDTINLLKASQFREDEYIQNLCKDMEDQVKQAVSRDDYFTKWGCHYLPSLAMAHNHKICTNFKDPGVQGYGGNLFNKQRDQIEKIVMSIDPPKPSYRSSGNVQINSALYTSYYYDPNNPCFEGNSEVKMANGTIKKVKEIKKGDEVFCPNTGKAEKVKCVIETEVKENLTQLVRLGKGLLITPYHPVRIQGKWLFPTDIAPTKMAQCSSVYSFLLESGHSMMINDIECVTFAHGFQEDKVKHEYFGTNKVSRDLMWMKGYFKGHVKLQNSPSIRDQATGLVVGLRSNRQWISKIQDLKYSSLSNIKQMSLNKLLQISKKLIKI |
| aTt | 00070820 |  | vWA novel hint | N-term Hint detected, Ubox | XP\_001007820 |  |  | - | MEFPDRYFCPLNKQIMYQPAQCEDGYNYEKSCIIKYVMDMGHSPITKQPLKHNQIQDNIALKQEIDQVRHLITDENRFRDLKLVAEEENKNDNNNIQVEGSNNQSNSSQLKLQINRFNDQVKISIKTPEGQQRSACDICCVIDVSGSMSDEAKIKNSKGDIESNGLTILDLVKHSVKTIINNLDERDRLSLVAFHTNAYKITDLTPMNENGRNHAIKELEKLIPLDSTNIWDGIYQALEVVKAGQQQSIQKGEQRVAFSQILLFTDGQPNVIPPRGHLPMLKKYKEENDVNCSISTFGFGYNLDSELLDQLAIEGRGSFAFIPDGQFVGTVFVNALSNLMTTLAVDAVLCIENSNGAQFEEVLIEEEQAKNILNKETVLGNYDYQRCSWGLNINIGTLQYGQSKDIVVTMKNVNNNSNKPYITATLKYRTSSTHKQPEEISASSSDISQQENEVMVDVFRLESVEAIRKAMTLFKTGNRGESQNIIKQHINNISSHQLSKQNKFIQDLVKDLAGQVTEAISVNDYYQKWGRHYLPSLLRAHQIQQCNNFKDPGVQHYGGKLFNQIRDKADEAFLKIPPPKPSIKKQGQAPKSVNMASYYNNSAPCFDGNCLVKMANGDIKKVMDIRKGDLIASPAINGVEAKVSCVVKTPCLNNQAYFVEFEDGLIITPYHPIRVNGIWQFPCQLKPTELRECDFIYSFLLESGHSMEINGIECVTYAHNFQEEIVKHDFFGTEKIITELKKMIGWENGYVTLSTDCMIRDPTTTLVIGLKQNIKNQLSIENIQNYQIIV |
| aTt | 00214760 |  | vWA novel hint | N-term Hint detected | XP\_001007172 |  |  | - | MEKNTKLDLQLSQTKNYVRVSVIPPDDLERHPCNIVCLVDGSLSMGSKLVIHQKNGGKKESDMTTLDLVKHTVKTIASSLNPQDRLALVGFSTHSKIYFELTEMDDQGKNVAFTEIDKMWAGGQTNIWGGLQDSLEVIKKGFRPNQNVCIFLFTDGRPTMIPAIGHVEMLRRWKEQHPAIQFSIFTFGFGNDLDTDLMLELSQEQNGIFSFISDSSMLGTVFSNALANILSTMANNVHLNLQLSEGYTFDGEGVMQSQSFQAKKTNKNTTLDLNLGLIRYGQTKDLILKILPQQNRKLSDVKITYTLKYKLFVGDNPQDIETVSQKDVQVVQNAMIDDECHYVRFLFIEKIKQSILRMNTGFQDRAIKNIDDLQVEIQNKAKENDAIVAFLECILQDLQGEVKLAFSNQDHFKNWGVNYLPSLCQAHFLQQCSNFKDKSLQNYGSILFKKIREITDKIFCGNKFKSQFINDDRQLYAKFYDLVNLNDFYDVSGGCIHGKCKVLLENNQYKNVEEIRKGDIVICPRIGNKAVKVVCVVKTLCTGSNHSFVKLGNLLITPWHPVRLMHEQQWQFPSSLGQTIFEQADAVYSFLLEDGHSMSIEGVECIALAHNISEPVAQHAYYGTHKIVDDLSNLQGFREGYVIVNQQQTKRDKLTHEVIGIQQNLEIKL |
| aTt | 00696950 |  | vWA novel hint | N-term Hint detected, this one brings up Hh quite well. | XP\_001025607 |  |  | - | MDKYNISIQESIKDDFLMISLVPPQNYSSRTNSNICCVVDVSGSMSSEAKIINQSSQKSDENYSLSILDVVKHSIKMIVNTLGSEDYLSIVTFSDSANVLFDLLPMNDSNKTMAIEKIENLSTEGGTELWKGLNSALNILLNNKTPNTNQSIFLLTDGQPTDSGIDTNLVKFKQAYPKLNCTINTFGFSSSSNSELMNKIAMEYNGMFSFIPDASFIATAFANALANTLTVYTNNCLLHITTLEGSNLTLHPAHSFLVKKMNSQGQQEILIDVGVVNTQQTRDFIFKINNLPKQLDRTYAQVKLTYQQSFSLNDYVKNIPSQTIQKSLIQREESEESQIYNHIYRCKVVSAIEESIQQYLQNSKIFEQQIKTKLMNLVDEMEQILPQNDRHFEGIYKDLVRTVSEAFTNQSQFNQWGHHYMLSLKRAHIIQQCNTFKDPGVQHYGQTLFKEIRAVADETFCKMPPPKPYSRASSYTQNNSSSNSSYQSAYNMNDYHNYSSGG CIHGDSLVYLQNGKTKKVSEIRKGDVVQCPELGQLQSTEVVCIVVSKCENNSHSFVQIGENLWITPKHPIRINGEWIYPNKMGEVQQKQSDFIYQFVLKSGHTMNIGGFEVICLGHGFQEPIASHEYLGSQAVINDLMNMKGWKEGKVLITARIKSEITGKVTAFVQQ |
| lNg | 58186 |  | vWA novel hint | JGI 58186, has ESTs, estExt\_fgeneshHS\_pg.C\_270027 |  |  |  |  | MSSSSTQRNYPNEFYCPITQEIMKDPVIGTDGQTYEREAIEHWLTIHATSPLTREAMSKELLVSNIALRNTIEQLVHGNTPIVVNRVTPLVEQDIEDHSLNLTIVSKQVSESKRRIHVKVSPPTGGQRQPCNLVCILDVSGSMGSSAEDLSSSNENTGFSRLDLVKHSVRTLIELMNEKDQISLIPFSDSARMELPLTKMDAVGKKKAIEKLEHLGPEGSTNVWDGLRLGMESSLNNPLCAKTNTCLILFTDGEPNINPPRGIVPTLEKYIKEHPLNSTIHSFGFGYSLDSALLKDIAMNGSGAYSYIPDCSMVGTTFVNMMSNILCTAVRRAELVISSMNGAKISHVYGSSQNGNNSTNEKQFTISMGGVQFQQSRDYIIDVDMHANNLPAIKVDLTYNHHSIEKVEPVNQSLSSEQATDMDANLTRLMFIECVEKCIQTYRNGNSSRDDSSKECKHQLESFLQTIAKLPSFTDERVSALVRDIKSDNTNEGQVSIAFSKQEFMNKWGYHYLPSLLRANWMQQCHNFKDPSVQIFGGSLFEALQSVADDIFCKLPPPKPSNVYSYGGGSYNTPTNMSSYYNAGGS CFDGNSLITMYDNSVKKVSEIKKGDRVKNMNGDSYATILCVLKSKTPSPIVKLCEMNGMYITPYHPVRVKGEWKFPIDIKSPQDVACDYVYNLVVDQGHVVSINGVECITLGHGFTDNSVVSHPYFGTDQIVKDLSSMKGYDLGMIEMNKYDLKRDPNTKMIISLSILE |
| pOs | AK110392 |  | vWA novel hint | clone="002-165-E07" | AK110392 |  |  |  | MSKAKAAAAGAAAPAAAVAVDAKAAAGGAGANAGAGVEVPNTFLCPIRSTVMLDPVTDNEGNSYEREAISEWLTRSAVSPLTNQPLSAADLRPNRALRELIDSWHTERGLPVPGAAEAKAKTDAAAAKPAAAAAADEKKNAPAAVDKPLLTAEVFRAGVAAAETDAKDVDAAAGGGPQVLVTVRAPVDLTNGDAKAAAGSGDRVPTDVCVVIDVSGSMGLEAKIKVAADADGGAGGGVAGQKPAVEEAGGFTTLDLVKHAVRTIIHSLGDADRLAVVTYSAQAQVVVELTAMNGAGKKKAEALVEKLEPDGQTNLWDGIYTGLEVIRKGAQQQAKAAPAPSPRAAKNRKVAAAGAAAGAAAGAAAAPRFTSNAAVLVLTDGQPNVEPPRGHLPSLAKYAADNKGLPAVLHTFGFGYNLDSQLLYDLSVAGKGFYSFIPDGGFVGTIFVNAIANALTTVARNVTVTIPNADANAGAEEAKAVDLGSVVMGQDRCAFVSVPALAANNAADIKSLPLRLQYDSNASAFGARALQATDAPLQVTSKGAAGAAAAVAAAPGGPAAAGAGAGAAVVPETTSVSVAAQRCRLGLVRAILDAVKLADAKDEAKAKAAIARASADIKAALATAPKAASEYDSPHAYLTALQTDADGQIALAVSRTDWYRQWGKHYLLSVARAHTLQSCNNFKDAGVQLYGGEKFRALRDAIDTLFCRLPPPKPSIQPAESFAAAFRAAGAGAAAGGVAVGHARGGRGGGAGAAAAPRKARVANMAAYNNRSNP CFSADSLVAMADGTRKPVCALNAGDVVCASARDAAVTRVVQCVVESRFDAAAPVHLVTLSKGLRVTLYHPVRMTDALSGAWSFPHALAPVQEHSGAEVPAVYSILLTEADAAHSGKGERYDCVLIGGVECVSLGHGYGSGDAGAGVAPHPYFGDRARVLRDLQAMKGFDAGRVVLNAATAARRNSQSGLIEALVQL |
| fAc | HCAG\_03368 |  | vWA novel hint |  | EDN06838 |  |  |  | MDPVQPAASSEDDFEIIDDQIPIRPRLSTSTTIAGERSPNEVGVQLHPLPDTNSMILSVHPPLHPEKEMPHVPCDIVLCIDVSYSMQSSAPLPTTDESGEREETGLSVLDLTKHAARTIIETLNENDRLGIVAFSTEAEVVYEISKMNESSKKAALKAVEALKPLSSTNLWHGLKLGLKAFENERHTPQSVQALYVLTDGMPNHMCPKQGYVTKLRPILQLLGHRMPMIHTFGFGYNIRSGLLQAIAEVGGGTFAFIPDAGMIGTVFVHAIANLYTTFATQAKVTFRTSGSVTLAQDLGSKTGLGLHEESTRDSNLTVAIGTLQYGQSRDLVIRMKNATTAATPSMAQATLTYQFQGCLKSVVADEQVFSQHTSLPVHVSDYHLSRARICAFLRSLYPLGLDKEYTNIDENGLKNARQQLDHIIKDIKQLEHTDEENDSLVRDLAGEEPEGQLRLSILVHANFAKWGKHYLLSLLNAHTHQMCNSFKDPGPLQYGKNSPFFCRCREELDTRFDNLPPPKPSIIEKAPDGTVKIRPSYKMSRYNCRNNP CFAGHCNIRLAERNSSLPIRDIRAGIKVWTPLGPRRVRAVLATAVKNTILCNIGSLSITPWHPIQVAGDWIFPSHVSEQNVPFSGTVYSVLLDPSPVSGAHAIMVEGHVCVSLGHGIQGCNDVRAHPFFGSYPSVVRSLASLSRDANGILRCSGMKRNPFTGLACGFIGEAGFRKSINKRPFKRFAKVGLRYHFQAPITINPRRRTACLAF\* |
| sPp | EC759192 |  | vWA novel Hint | EST |  |  | EC759192 |  | LCFESVKSAQVEERVANCSSFTNGGVDLEVHHLRLAVVSTINHVLELVQSGKDKEAQAALADITELVRQCQDPRAKALLQDLTGQVQEAIQKTPENYFQKWGRHYLPSLARAHLLQQCNNFKDPGIQVYGGEMFQQKRDQIDDIFVKLPPPKPSVRRARPTSHSSSTFSSPSYVNMGSYHNAAGP CFAGDCLVEMANGTMKKVRDIVRGDMVMGHKGPAMVDCVIKTHC |
| aAc | Ac3725 |  | vWA novel Hint | EST clone Ac3725 |  |  | CF066289 |  | AGAARLAELAAVSEEEIRRIPQYATLDQVQLNVACEDMLKQAELTIKDLASLVQRSPLSTQSGHPVAALLEDIVGQTMEAFSCLDYFRKWGRHYVVSIACAHRSQQCTNFKDPGVQVYGGTLFNRLRDDADDTFNTLAAPQPTNGQYRYILGGWRNGRLQPGKVIHNPDFHGGTSVAPSTAAAVINMADFNDRYCG CVHGDSVVHLADGQCIAVASLRKGDIVQSGVGGATARIECIVRSQCPRRRACFVRMAGGLKGTPYHPVLVNGK |
| aKb | CV173950 |  | vWA novel Hint | EST est\_k\_brevis301 |  |  | CV173950 |  | AQQKLDELVAYGGTDIWQGLSHGLDALHTDVEKGRLGHIMLLTDGVSQSADRIIPNLQQYCTKHERLPGTISTFGFGYNLDSRLLVQLAAEGSGSYSFIPDAGFVGTVFVNTVSNLMVTMAHEVYLGLEPGSDSGDELILKDCMLGDLPVTQQDGYTRVKLGTLQYGQTKDILLRMKARSAAAPLLAASVQYEAAAGHSGVPMGSPLVEASCGDLSEEMLQKMERNRCRSIFADAVLRIFKDQLEADTAKALLNQAH |
| Mb | 31857 |  | vWA novel Hint | JGI: 31857|estExt\_fgenesh2\_pg.C\_60157 |  |  |  |  | MEQPPSHLVCQISHELMRDPVAAPDGYVYDRTNILQWIGQGEDGQRNNSPFDRSITISAADLRPAMTIRSALEEYIAQHHLEFEVAPLVTGRPALKLPARLASELELEVALHPVPGETRKAILELIPQRQTATTNIHLNLVLDVSGSMGAAVTARDESNTLIEYNLCVMDLVKFASQVAVKCLAPGDVISIVTFSDAAKIIVEPISVPDPKMGADTTVADVLGKIDAIYHGGSTNLWAGIETGLQLLASCAQPHLHNVCVALTDGEPNRHPEQGYETAHRRFKQMPNFSYVLHTLPFGFGRIDSALLQSLARTGEGIMVNIPDCGMVGTVFTNLIANAKVTSHRGLRLLDLDGVLDRAEPTGATFDYNASKQFLALGTLQAEQPRYLLLHLNQDVHDCTALRLQLAAECLPVAPGAGRTALERPPTGKAGLIAVKPRATCGLESEVLLRVLFRDAACAMLSRVVNLMATGETGAARYALENFLSLHDVLTEAPEMEVDLNEQVRQALVPAAYKRWGHTYVPSLAMAHSAQVRHNFKDPGVQAYGGPLFGTEAGRMSDIFDAMPVPKGTRQVGASLASGAVLNNCYGG CYSADSLVDLASVRAPGTTGDVAQRTGDVRNLAGAWQRRADAIRAGDVLANGAVVDYVVAVACAGGEHDFCQLSPGLRITPWHPVREPSTKRWAFPSELVVPKRHASEYMYSFVLTAGGEVPVDGHTTVGLAHELQEPVAAHPFFGSPALRDRVALLAARAGTARTLWLAPQAFVRDTATGLVKDFNEHAVRSQPSVS |
| eTf | Vwaint |  | vWA novel Hint | clones TfEST035G12, TfEST050F06, TfEST029G11 |  |  | CX154328, CX156329, CX155376 |  | HEVRVKNAVYDVKHKIYIYNGTTTNLMVPLKNVDDLNITLDILTTNQYFVCNDIGPIANSNEHVLFKDQQYRSKLIQLINTHKFGRNCEQAINDIKQLFNELESLTDRSEFLENIMTDLINPSDSCGQIEKSFQKDYYSKWGKYYLRSLLRFHIVEQCGNFKDKSLQLYGGELFHQTRQVANKIFMAIPAPKPIEIPAYGGRGHSRQRGGMPINMARFNDRNAG CFNGDALVSMKNGKEKKVDHLKKGDVLSNGGKVVCVVKTLNQNESKIAKAVKIGNALFTPYHPIKVDGNWIFPIDYAKPELYAVDFWYNLILEGAGSATIGGIEAVTLGHEKKDGVCNHPYFGTNQVLKALMKYDGYNKGEIEININ |
| aPt | 36259001 |  | similar to cTt hint, but internal, intein? |  | XP\_001434522 |  |  |  | MLEYFNKWKKIDDLPPIADEINFQENQNIQLCQQNMNQVIKQQGQEENENNLIFLDLKEFGIQELQTEVIEMSQIIEEFMQRIFQKYLTSIDTNNIYMINEGGLLDKKHQLNFYHIKNNSIIKFIKMKQTYPEKWIIIIKTLTGKTLTLEINNKDMSIEQLKMLIQNREGIPPDQQRLIFEGILLQDGKMLWEYNIGPESTLYLTLRLRGGCFPGNAPIKLFNGRTKIIKEIELGDIVMCYDFEQKQFKSSIVVFKKISSEKQQLIEIISENGSIICTPNHPIYTQTGWKALEPHPNFFIEKLTVTDLIFDSNERLVNILQINQLKDTEIVYNITTSYPNNFIAFDFLVHNMNKIFIQINGFNEEFEILPYFLVQNLKVIIEKKKGIPMIDQQLYYQGILMKDHFSLEDYKLLQNGTEPDLLILKKLQYEINQNTNSETCVSIITHNSQYQLQIKKNQKIGKIKKILEFVESREQIERSFILCNGKPLRSKNIDENKTDLANVQSIFLIDQQSGGISMRFTQEINDQQVQTASQLKDSLQWLGNPQINEFIQANLEEINGNQQKKYYYQQIPLPCMIAVRLYTCDLIYRKLNNDLRTSDYRRWKQYLRYLMEGFRLMKYYKGVAYRGIKDYQNTTLYKKGKIVQWSEVTSVSLNYKIAQHFSNNKGMIFNIQLISGKDISKISIYEGEQELIMYPFSTFVVDEVQIKPNQPHIVTMRELPLPRSHCVLLWVDDNPENNFNYAYEVERQNNNISVIFCTSTKDAILIISKYNWMIYLSESQFRVISDMVRIEDGKLNYNAGIELLIHLYQKMKYKNRTIIFCGDQKRAQEECRSRNIQGNFEITNNEHVLKQFLQFN |
| fCt | PI-CtrI |  | hog intein |  | P38078 |  |  |  | VGCFTKGTQVMMADGADKSIESIEVGDKVMGKDGMPREVVGLPRGYDDMYKVRQLSSTRRNAKSEGLMDFTVSADHKLILKTKQDVKIATRKIGGNTYTGVTFYVLEKTKTGIELVKAKTKVFG--- YYGITLAEETDHQFLLSNMALVHN |
| pAt | At5g60710 |  | vwa |  | AAM53301 |  |  |  | MGSKWRKAKVALGLNLCLYIPKTLEDSSPPRRSDDAVSLSPVIVQRPSTPTPSSSGLRLPRSMSKSSSKKTCAICLTAMKAGQGHAIFTAECSHSFHFQCITTNVKHGNQICPVCRAKWNEIPIQSPNAKPKSGVKPIGRPRDDAWMSIPPRRSSPIQYTSRPDCLRVSSIFNTEPAVFNDDEALEHQDRSAESGLDKPGVTGTLEVKTYPEISEVVRSVSFKDFAVLINLKAPTSSKSSSNPSSSSRAPVDLVTVLDVSGSMAGTKLALLKRAMGFVIQNLGPFDRLSVISFSSTARRNFPLRLMTETGKQEALQAVNSLVSNGGTNIAEGLKKGARVLIDRRFKNPVSSIVLLSDGQDTYTMTSPNGSRGTDYKALLPKEINGNRIPVHAFGFGADHDASLMHSIAENSGGTFSFIESETVIQDAFAQCIGGLLSVVVQELCVTIECMHHLLRIGSVKAGSYRFDNGPNSRTGSIAVGDLYAEEERNFLVNLDIPIVDGVSDVMSLLKVQCVYKDPVTKETVNLNNSGEVKILRPIVMTERRPVVSVEVDRQRIRLRAAEAISEARVLAERGDLTEAVSVLETCRGLLTESVSGRAGDQLCVTLCAELKEMQERMASRQVYEASGRAYVLAGLSSHSWQRATARGDMSDSTTTSYQTQSMVDMVNLSQTMTFGMPIASSNSSPSGQRKLRQALSFPAKPRPR |
