# Supplementary material for: Evolution of hedgehog and hedgehog-related genes, their origin from Hog proteins in ancestral eukaryotes and discovery of a novel Hint motif
Source: BMC Genomics. 2008 Mar 11;9:127. doi: 10.1186/1471-2164-9-127 (PMC2362128; doi:10.1186/1471-2164-9-127)
Supplement: Additional file 10 — Multiple sequence alignment of "Hedge" domain containing proteins and Hedgehog proteins. Note that Os hhlike and NV 200640 do not line up in the Hog domain region. [file 1471-2164-9-127-S10.pdf]

```
Os_hhlike   ----------------------------------------------------------------------------------------------------------------------------------
Nv_200640   ----------------MGLLAIALILQLCVASFG-----------------TLAVALPSSLSQGSSYP-YSKTEVSVCGGIRDVILRNSARFRKILVRNADTEVVFENDDC----RRTTARAKSKLDVLA   92
Nv_95413hh  ---MGQQFPCLL----SCGIALFIFQLLIHGAGT------CGPGKA----AFDRDNRAIPMKIREHIPDTSETSLQASGPSR-KIKRGSNGYKE-LITNADPNIVFR-EDKAGNNRRMSKRCERKLKILS  110
Nv_241466hh ---MRLHLFLFY----PLAFALVLFQFSLDFANS------CSSRPS----LSRRGRR--PLYFKQRVPDVDEFSLGASGRPQGKITRNSSKFNK-LVACYNTDIVFKDEERTGADRLMSKRCREKLRNLA  110
Acm_hh      ------MAIAKS----NILCQLIALFVAAQLRCSL----SCGSRTG-HG-ARNPGS---PLMQYQCVPDLSENSQGASGPAKGKITRNSPEFEK-LEPCYNTAIIFKDEEGTGADRLMSKRCKEKLIELA  110
Sp_hh       --MVRADMVKWL------TVQITTVLCLIALTQA------CHPGRS--GKTSHRPRNRTPLQYKQRVPNISEDTFGASGPPEGRINRNDERFNT-LSPNNNDDIVFKDKEGTGADRLMTQRCKDKLNTLA  113
Lv_hh       --MVHADMVKWL------TVQITTALCLIALTQA------CHPGRS--GKTSHRPRNRTPLQYKQKVPNISEDTFGASGPPEGRIDRDDERFSK-LSPNNNDDIVFKDEEGTGADRLMTQRCKDKLNTLA  113
Ts_hh       -------MLSWC----DLLFFLSVTFSSNVHSAKVYCWRWCSSGRM-AG---MAYKKYYPLIYNERFPNEPENAPGSAGAAELRIRRSDPGFKR-LVQNNNPDIIFRDEENTGADRMMTYRCKQKLDMLA  114
XC_Hh       ------------------------------------------------------------------------------------------------------------------------RCKAKLDTLA   10
Ag_Hh       --------MVWL---------MVALLALVQLTAG------CGPGRG-IG-GPRRTRKLLPLVFKQHVPNVSENSLSASGMQEGPISRNDSKFRS-LETNYNKDIIFKDEEGTGADRVMTQRCKEKLNILA  104
Dh_Hh       MRHIAHTPRGSC---FMALLLLLLLALNFRHAHS------CGPGRG-LG--RRRERNLFPLVLKQTVPNLSEYHNSASGPLEGAIQRDSPKFKN-LVLNYNRDIEFRDEEGTGADRVMSKRCREKLNMLA  117
Dm_Hh       MRHIAHTQRCLSRLTSLVALLLIVLPMVFSPAHS------CGPGRG-LG--RHRARNLYPLVLKQTIPNLSEYTNSASGPLEGVIRRDSPKFKD-LVPNYNRDILFRDEEGTGADRLMSKRCKEKLNVLA  120
Bf_AmphiHh  ---MAGVLARWM----VTLVAISALGTHWGPSEA------CGPGGR-FG-RRRHPRKLTPFVYKQQMPAVSENTFGASGLFNGRITRDSERFHT-LKQNFNTDIIFKDEEKTGADRFMTQRCKDKLNALA  114
At_Hh       ----------MF----HTLAAVLCLAALVNSASS------CGPLRG-GG-RRRPPRKLTPLVFKQHVPNVNEFTLGASGQSEGKLTRDHPKFKSVLVPNYNSDIIFRDEEGTGADRLMTQRCKEKLNTLA  108
Cap_Hh      --------MHFH----DHLFFVFCLLALSRPAWA------CGPGRA-SG-RRRGARKMTPLVFKQHVPNISENTLGASGLNEGRITRDDPRFKD-LVENYNPDVVFKDEEGTGADRIMSQRCKDKINTLA  109
Gb_Hh       ----MPLLLRWL----------LLLLLMQGAARA------CGPGRG-AG-RRRASRRLTPLVFKQHVPNVSEHTLGAAGPAERRVARDDPRFRD-LVPNYNADIVFKDEEGTGADRLMTQRCKEKLNTLA  107
Mm_Dhh      --------MALP----ASLLPLCCLALLALSAQS------CGPGRGPVGRRRYVRKQLVPLLYKQFVPSMPERTLGASGPAEGRVTRGSERFRD-LVPNYNPDIIFKDEENSGADRLMTERCKERVNALA  111
Hs_DHH      --------MALL----TNLLPLCCLALLALPAQS------CGPGRGPVGRRRYARKQLVPLLYKQFVPGVPERTLGASGPAEGRVARGSERFRD-LVPNYNPDIIFKDEENSGADRLMTERCKERVNALA  111
Dr_dhh      ---MTLAPWLRL----ARLGLLTVCLYSWLVVDG------RGPGPG-YG-GRHRQRKLTPMSYKQYVPGVSENNLGASGRAEGRITRSSERFNE-LVCNYNTDIDFKDEERSNADRFMTKRCKDCLNKLA  114
Tr_fhh      ---MKQCWWARL----AQVSLLAAWSCVWL-VQG------CGPGPG-YG-IRTRPRKLKAMYYKQFFPNLSENNLGASGRAEGKITRNSERFNE-LVCNYNPDIVFKDEENTNADRFMTKRCKDCLNRLA  113
Dr_ihha     -------MRLPV----VFGLLVGCALIFAPVNEG------CGPGRG-HG-KRRPPKKLTPLNYKQFSPNVAEKTLGASGRIEGKITRNSERFKE-LTPNYNPDIIFKDEENTGADRLMTQRCKDKLNSLA  110
Dr_ihhb     -------MRLST----AAALLTGFILAFSPAYDG------CGPGRG-YG-KRRTPRKLTPLAYKQFSPNVAEKTLGASGRYEGKVTPSSERFKE-LTPNYNPDIIFKDEENTGADRMMTQRCKDKLNSLA  110
Mm_Ihh      ---MSPAWLRPR----LRFCLFLLLLLLVPAARG------CGPGRV-VGSRRRPPRKLVPLAYKQFSPNVPEKTLGASGRYEGKIARSSERFKE-LTPNYNPDIIFKDEENTGADRLMTQRCKDRLNSLA  115
Hs_IHH      ---MSPARLRPR----LHFCLVLLLLLVVPAAWG------CGPGRV-VGSRRRPPRKLVPLAYKQFSPNVPEKTLGASGRYEGKIARSSERFKE-LTPNYNPDIIFKDEENTGADRLMTQRCKDRLNSLA  115
Mm_Shh      ------MLLLLA----RCFLVILASSLLVCPGLA------CGPGRG-FG-KRRHPKKLTPLAYKQFIPNVAEKTLGASGRYEGKITRNSERFKE-LTPNYNPDIIFKDEENTGADRLMTQRCKDKLNALA  111
Hs_SHH      -------MLLLA----RCLLLVLVSSLLVCSGLA------CGPGRG-FG-KRRHPKKLTPLAYKQFIPNVAEKTLGASGRYEGKISRNSERFKE-LTPNYNPDIIFKDEENTGADRLMTQRCKDKLNALA  110
Dr_shhb     ----MDVRLHLK----QFALLCFISLLLTPCGLA------CGPGRG-YG-KRRHPKKLTPLAYKQFIPNVAEKTLGASGKYEGKITRNSERFKE-LIPNYNPDIIFKDEENTNADRLMTKRCKDKLNSLA  113
Dr_shha     -------MRLLT----RVLLVSLLTLSLVVSGLA------CGPGRG-YG-RRRHPKKLTPLAYKQFIPNVAEKTLGASGRYEGKITRNSERFKE-LTPNYNPDIIFKDEENTGADRLMTQRCKDKLNSLA  110
Pv_Hh       --------MKLLPPFSSICSFAFIILFITSLTHA------CGPGRG-SG-SRRKAKKHTPLVFKQNVPNVSENSLGASGMSEGRIKRDDAKFKD-LVRNHNADIVFKNEEGDGSDYHMTRRCQDKLNSLA  113
Ob_Hh       ----MPQRSLRH----QLGMILVFFLLVTSHSLA------CGPGRG-PG-KRRGPRKRTPLVFKQHIPNVSENTVGASGIHEGKITKPDPRFKE-MVTNLNPNIVFRDEEENNEDRVMSKRCKDKLNTLA  113
ruler       1.......10........20........30........40........50........60........70........80........90........100.......110.......120.......130
```

```
                         : *  .:               .** .**:            :   :          *. **  :*** * :      :  **  :
Os_hhlike    ----------GRVRVSEAWEEPTSTDPAGIHPLASLHYEGRSARLDISIDILPGISVPDRDTDVSLLTRLGGLAVCAGFDHVYHS-PDGHLTVSVRQQK-----DRHGNI--------------------   94
Nv_200640    SRVRQEWAG-RKLKVIKAWTD---QR--TAQDPASLHYEGRAL----------RLQLDN--NDRSMLSRLAGLALASGFDWVSYPLNSDYIHASVIRDV----------CQTSVDLVFILDTSG------  188
Nv_95413hh  SLVRKEWIGDVKVRVIRAYDDGTSKK--RHHGPHSLHFSGRAL----------DITTSD--EKRDKLPMLGRLAYRAGFDWV-YR-AKAYIHASVKSDDEDDY-RENTGCFPAASTARLATGEQVPMVDL  223
Nv_241466hh  TKVKQKWKG-VKLRVTEAWDE----D--GQHSLDSLHYEGRAV----------DISTSD--KDPKKLPDLGSLAVDAGFDWVYYD-RRSSIHASVRSDD-DRK-DSWGSCFHSEALVTIENGERIAIKDL  218
Acm_hh       SLVKNQWPS-LKLVVTEAWDE----Q--GQHSKNSLHYEGRAV----------DLRLSDTYKSNPKLALLGRLAVNAGFDYVLYE-SKTHIHASVREDSYVDK-TKRTGCFSSESTVRLENGAVLRVDQL  221
Sp_hh        ISVMNEWPG-IKLRVVEAWDE----D--QPNV-EPLHAEGRAV----------DITTSD--RDKNKYGALARLAVEAGFDWVNYE-SKAWVHCSVKSES-AAA-KNSGGCFPGFSQASLENGRTISMLDI  220
Lv_hh        ISVMNEWPG-IKLRVVEAWDE----D--QPNV-EPLHAEGRAV----------DITTSD--RDKNKYGALARLAVEAGFDWVSYE-SKAWVHCSVKSES-AAA-KNSGGCFPGFSQAYLKNGRMISMLDI  220
Ts_hh        ILTMNYWPN-VKLRVIDAWYE----Q--NRYSRSALHYEGRAV----------DITTSD--RDRNKLGMLARLAIQAGFDWVYYE-SHLHVHASVQPDSVFRT-KRMLNCFAGSSTVLTKENGRKRMDEL  223
XC_Hh        MLVTNFWPG-VKLRVTEAWDQ----D--GFHSADSLHYEGRAV----------DITTSD--RDRNKYGLLARLAHQAGFDWVHYE-NRMHVHASVKSDSVTRH-KRVLKCFPGHATVTTANGIRKFMSDL  119
Ag_Hh        VSVMNQWPG-LRLMVTEGWDE----D--HMHAPESLHYEGRAV----------DIMTSD--KDRSKIGMLARLAVEAGFDWVFYE-SRSHIHCSVKSDS-SQS-SHTSGCFTGDSTVLTEAGVHRKLSEL  212
Dh_Hh        YSVMNEWPG-VRLRVTESWDE----D--RQHGQESLHYEGRAV----------TIATSD--HDQSKYGMLARLAVEAGFDWVSYV-SRRHIYCSVKSDS-SPSISHMHGCFTPESTALLESGAEKALGEL  226
Dm_Hh        YSVMNEWPG-IRLLVTESWDE----D--YHHGQESLHYEGRAV----------TIATSD--RDQSKYGMLARLAVEAGFDWVSYV-SRRHIYCSVKSDS-SIS-SHVHGCFTPESTALLESGVRKPLGEL  228
Bf_AmphiHh   ISVMNQWEG-VKLRVTEGWDE----D--GFHTEESLHYEGRAV----------DITTSD--RDRTKYGMLARLAVEAGFDWVYYE-SKAHIHCSVKAES-DTT-ATQGGCFSAESWVTRDDGNRIRMRDV  222
At_Hh        ILVMNQYPG-VKLRVTEGFDE----E--SYHSTQSLHYEGRAV----------DVTTSD--RDRSKYGMLARLAVEAGFDFVYYE-SRSHIHCSVKSES-ADA-GRSGGCFDGDSTVRTEAGPKK-MSDL  215
Cap_Hh       ISVMNQWPG-VKLKVTEAWDE----D--GFHAKDSLHYEGRAV----------DITTDD--RDRSKYGMLARLAVEAGFDWVYYE-NRGHIHCSVKSDS-SIT-AKTGGCFSADDTVKRVDGSSLPIQHL  217
Gb_Hh        ISVMNQWPG-VRLRVIEGWDE----E--GGHAADSLHYEGRAV----------DVTTSD--RDRSKYGMLARLAVEAGFDWVYYE-TRGHIHCSVKSES-SQA-AKSGGCFAAESTVQTP-GGLCALAEL  214
Mm_Dhh       IAVMNMWPG-VRLRVTEGWDE----D--GHHAQDSLHYEGRAL----------DITTSD--RDRNKYGLLARLAVEAGFDWVYYE-SRNHIHVSVKADN-SLA-VRAGGCFPGNATVRLRSGERKGLREL  219
Hs_DHH       IAVMNMWPG-VRLRVTEGWDE----D--GHHAQDSLHYEGRAL----------DITTSD--RDRNKYGLLARLAVEAGFDWVYYE-SRNHVHVSVKADN-SLA-VRAGGCFPGNATVRLWSGERKGLREL  219
Dr_dhh       IAVMNQWPG-VRLRVTEAWDE----D--GHHPPGSLHYEGRAV----------DITTSD--RDTKKYGLLAQLAVEAGFDWVHYE-SKYHVHCSVKADH-SVA-VEKGGCFSASGLVTMADGVQKPMSCL  222
Tr_fhh       LAVMNQWPG-VHLRVTEAWDE----D--GHHPPGSLHYEGRAV----------DITTDD--RETEKYGLLAQLAVEAGFDWVHYE-SKYHIHCSVKADH-SVA-VEKGGCFPGWSRVTVAGGFQKSLSSL  221
Dr_ihha      ISVMNMWPG-VKLRVTEGWDE----D--GNHFEDSLHYEGRAV----------DITTSD--RDRNKYGMLARLAVEAGFDWVYYE-SKAHIHCSVKSEH-SVA-AKTGGCFPASALVTVEDGSLKTLDSL  218
Dr_ihhb      ISVMNLWPG-VRLRVTEGWDE----D--GLHSEESLHYEGRAV----------DITTSD--RDRNKYRMLARLAVEAGFDWVYYE-SKGHVHCSVKSEH-SVA-AKTGGCFPGRALVTMKDGSHRQIRDL  218
Mm_Ihh       ISVMNQWPG-VKLRVTEGWDE----D--GHHSEESLHYEGRAV----------DITTSD--RDRNKYGLLARLAVEAGFDWVYYE-SKAHVHCSVKSEH-SAA-AKTGGCFPAGAQVRLENGERVALSAV  223
Hs_IHH       ISVMNQWPG-VKLRVTEGWDE----D--GHHSEESLHYEGRAV----------DITTSD--RDRNKYGLLARLAVEAGFDWVYYE-SKAHVHCSVKSEH-SAA-AKTGGCFPAGAQVRLESGARVALSAV  223
Mm_Shh       ISVMNQWPG-VRLRVTEGWDE----D--GHHSEESLHYEGRAV----------DITTSD--RDRSKYGMLARLAVEAGFDWVYYE-SKAHIHCSVKAEN-SVA-AKSGGCFPGSATVHLEQGGTKLVKDL  219
Hs_SHH       ISVMNQWPG-VKLRVTEGWDE----D--GHHSEESLHYEGRAV----------DITTSD--RDRSKYGMLARLAVEAGFDWVYYE-SKAHIHCSVKAEN-SVA-AKSGGCFPGSATVHLEQGGTKLVKDL  218
Dr_shhb      ISVMNHWPG-VKLRVTEGWDE----D--GRHLEESLHYEGRAV----------DITTSD--RDKSKYGMLSRLAVEAGFDWVYYE-SKAHIHCSVKAEN-SVA-AKSGGCFPGSGTVTLGDGTRKPIKDL  221
Dr_shha      ISVMNHWPG-VKLRVTEGWDE----D--GHHFEESLHYEGRAV----------DITTSD--RDKSKYGTLSRLAVEAGFDWVYYE-SKAHIHCSVKAEN-SVA-AKSGGCFPGSALVSLQDGGQKAVKDL  218
Pv_Hh        VSVMNNWKG-VMLRVTEAWND----N--NSHAKDSLHYEGRAV----------DITTSD--KDRAKYGMLARLAVEAGFDWVYYE-SRGHIHCSVKSDS-SVA-IKIGGCFPGTGVLQTETGW-KTMSQV  220
Ob_Hh        IAVMNEWPG-VKLRVTEAWDT----Q--GHHAPTSLHYEGRAV----------DITTSD--RVRSRYGMLARLAVEAGFDWVYYE-SRSHIHCSVRSDS-LDT-THYGGCFPRTGKVVVRNKGTITLDQL  221
ruler        .......140.......150.......160.......170.......180.......190.......200.......210.......220.......230.......240.......250.......260
```

H e d g e   d o m a i n

```
                                     : .
  Os_hhlike -----------------IVYPSGATFLQVEIPTGY--------ENDYTISGKEASLLVDSDG----------------------RLEDELSGHFKVKDVLSLHSDTG-----------------------  154
  Nv_200640 SVGSY------NFEKMKTFVKNVVDFFNIGPKGTHVAVITYSTWAQVEFNLKAHH--------------SSKAA----------LKNAVNAIYYRSG-WTYTADALD----LAGRNIFQVANGMRP----  279
 Nv_95413hh RLGDKVAS---VDDRGGIIYSPVIMFLHRSPELIMDFLKIRT-ENQTELVITASHLIYKI---------NRQSK----------RKEACFAKDVTVGDLVFVGGRSS-TKSLSPGRVTSVERTRSR-GVF  328
Nv_241466hh KTGQRVQS---MDETGRLLYSEVLIFLDYKPWLSKVPFTIIE-TDVANLALTRNHLIFVMKRN------SSTIY----------DASAKLAQFVTPGDYVLVNSKGK----LHPSRVMSVRIEHKL-GAV  323
     Acm_hh KISDRVQV---MMQDGTIGYSEVIMFADYLPNI-------------------------------------------------------------------------------------------------  251
      Sp_hh RVGDEVAV---VNDDGALDYSDVIMIVHRKLNDSTLFYVIET-EDKSVVQLTPQHLIYVSET-------ESSFS----------QSKAMFASEVRTNQFVYTTGQNH-DRGVRPKRVVSVTTRLGR-TAV  327
      Lv_hh RVGDEVAV---VNNNGELDYSDVIMIVHRKLNDSTLFYVIET-EDKTIVQLTPQHLIYVSER-------ESNFD----------QSRAVFASEVRTNQFVYTTAQNH-DRGVRPMKVVSVTTRLGH-TAV  327
      Ts_hh EIGDRVLA---RKADGKLTFSPVILFLHRDQQTKAKFKAVHI-SNDTLLTISPDHLIYKVDPMTN----DNSGG----------SMETVFAADLLAGDQVFVRNGIF---DISRATVLGIGEIERQ-GLY  331
      XC_Hh SVGDMVAVST-GHVNSDQYYQPIIAFLHRNTSMPAKFVQIRTSPSGKVFTASSEHLIYVW---------NGQNG----------SRS-------------------------------------------  186
      Ag_Hh RIGERVQA---VDAAGRTVFSEVLMFMDRDTHQRREFVTIEA-EGGALLKVTPAHLVMVW---------RRERS----------ETRFVFADRVREGDHILVHVAGS----LEPRAVHRISATLAE-GVY  314
      Dh_Hh AIGDRVLS---MDVKGQPVYSEVILFMDRNLEQVENFVQLHT-DGGAVLTVTPAHLISVW---------QPERQ----------TLNFIFADRVEELDYVLVRDATG---ELQPQRVLRLGSVQSR-GVV  329
      Dm_Hh SIGDRVLS---MTANGQAVYSEVILFMDRNLEQMQNFVQLHT-DGGAVLTVTPAHLVSVW---------QPESQ----------KLTFVFADRIEEKNQVLVRDVET--GELRPQRVVKVGSVRSK-GVV  332
 Bf_AmphiHh RPGDKVLS---MDSGGHPVFSEVLTFMDRESRGPWVYYTIHTDDRNITVTATPSHLVFVTES-------RDLSS----------PRIAKFMSDARPGEFLLTPDSDG--GGFRKVKIVSVTMREEK-GAY  329
      At_Hh QVGERVQV---ARTDGQTDYSEVILFLDRNETQQRLYNTLET-ENGRSITLTPTHLIFTA---------SPHQT----------TPQATFAKHVEIGDYIYVASDRK----VTLEKVISVTSSAKK-GVF  317
     Cap_Hh RIGDAIQA---STDNGDVVYSPVILFLHREENAVASFVTLKT-EGGRSLTLSPSHLIHTA------------------------EHGEIYASDVKIGQHLLALNNNR---SLDKDPVVAMTTQYRR-GVF  315
      Gb_Hh RVGRRGAGA--GPGHGRLAFSPVLLFLDRDP-APRTLLRVRT-ASGRTLALTPSHLLPVA---------RAGGG----------EPEARFADAVRPGDALLVAADAG--GAVRPDRVLHVDAEATRGGVV  319
     Mm_Dhh HRGDWVLA---ADAAGRVVPTPVLLFLDRDLQRRASFVAVETERPPRKLLLTPWHLVFAARGP------APAPG----------DFAPVFARRLRAGDSVLAPGGDA----LQPARVARVAREEAV-GVF  325
     Hs_DHH HRGDWVLA---ADASGRVVPTPVLLFLDRDLQRRASFVAVETEWPPRKLLLTPWHLVFAARGP------APAPG----------DFAPVFARRLRAGDSVLAPGGDA----LRPARVARVAREEAV-GVF  325
     Dr_dhh WPGEKVLS---VSGSGEVVFSRVLLFLHLDRESRTSFFIITT-ENEKRIALTPNHLIFAAHNL------KLHHH----------DYETVFARNVRIGDYILTTGGDR---GIQPSKVVSVSLEERM-GVY  328
     Tr_fhh TPGDRVMA---LSETGQVVFSPVLLFLHRDPESRWRFLSLQT-EDGRRLAVTPHHLVFS-DAHC-----GPDSS----------QYQAQFASRAQTGTCVLVHTAGG---EVHPSRIVSITEEESV-GAY  327
    Dr_ihha QPGEKVLASSESDGSGTLVYSEVIAFLDRDPSARKQFFTIET-DSGAKLSLTAAHLLFVSEGNCS--G-SAANA----------ELRSVFASDVLPGQCVVSTQAAG--QHGRLSRVSRIQMQEDR-GVF  331
    Dr_ihhb QAGDLVLASEGSDGTGDLIYSEVLTFLDRRPITQKHFYVIRT-EDGASVSLTAAHLLFMRVGNCSNRG-EPKPG----------AVRTIFASDAQVGQCLLLGKLRK-----RFSQITHVGVREDQ-GLY  330
     Mm_Ihh KPGDRVLA---MGEDGTPTFSDVLIFLDREPNRLRAFQVIETQDPPRRLALTPAHLLFIADNH------TEPAA----------HFRATFASHVQPGQYVLVSGVPG----LQPARVAAVSTHVAL-GSY  329
     Hs_IHH RPGDRVLA---MGEDGSPTFSDVLIFLDREPHRLRAFQVIETQDPPRRLALTPAHLLFTADNH------TEPAA----------RFRATFASHVQPGQYVLVAGVPG----LQPARVAAVSTHVAL-GAY  329
     Mm_Shh RPGDRVLA---ADDQGRLLYSDFLTFLDRDEGAKKVFYVIETLEPRERLLLTAAHLLFVAPHNDS----GPTPG-----------PSALFASRVRPGQRVYVVAERGGDRRLLPAAVHSVTLREEEAGAY  331
     Hs_SHH SPGDRVLA---ADDQGRLLYSDFLTFLDRDDGAKKVFYVIETREPRERLLLTAAHLLFVAPHNDSATGEPEASSGSGPPSGGALGPRALFASRVRPGQRVYVVAERDGDRRLLPAAVHSVTLSEEAAGAY  345
    Dr_shhb KVGDRVLA---ADEKGNVLISDFIMFIDHDPTTRRQFIVIETSEPFTKLTLTAAHLVFVGN--------SSAAS----------GITATFASNVKPGDTVLVWEDTC--ESLKSVTVKRIYTEEHE-GSF  327
    Dr_shha NPGDKVLA---ADSAGNLVFSDFIMFTDRDSTTRRVFYVIETQEPVEKITLTAAHLLFVLDNS------TEDLH----------TMTAAYASSVRAGQKVMVVDDSG---QLKSVIVQRIYTEEQR-GSF  325
      Pv_Hh VAGDSVLS---MNSNGKLEYSPVIAFIDRNERELERYITLHT-EDKKDITLTSKHLIYMSTSNVTT---DDVTD----------SFNVVYADDVIEGDYVLVTSDPV-GEVIKPTRVLTISEHTIQ-GVY  331
      Ob_Hh KVGDSVLS---VDLQGELTYSEVIAFLDTNKDSSGYFHRIET-ENGHTIRLTGKHLIYSSYTNRTRFDLNDNDS----------EFEATYADQVQIGDYVMTTDRTA---GLFASRVKKIAAVSEK-GVV  333
      ruler .......270.......280.......290.......300.......310.......320.......330.......340.......350.......360.......370.......380.......390
```

Hog domain

```
    Os_hhlike ------------------------KAFRYFRLSPGVLYC-----LESVYNEV--------------------------------------EEGRLEIVSAYRTKTDNDGRRL----------------  199
    Nv_200640 ----DKGIPKIAVLLTDGYSNGNN------PLGPANDLR-----AAGVNVFC-----------------------------------------------------VGIGN------------------  321
   Nv_95413hh APLTQAGNLFVDDILVSCYAITSSDSIAHWSLAPVRLVG-----AICPRCFD-----------------------------------------IEYSGIHWYPRILLTIFGKIVELCGGFL--------  403
  Nv_241466hh APLTAQGTIIVDGVVASCYSEVTSHTISHLAFSPLRGLR-----YWLPSVFSWLHE-----------------------------------GITPAGVHWFPRFLISLNQIVRIAEFA----------  401
       Acm_hh --------------------------------------------------------------------------------------------------------------------------------  251
        Sp_hh APVTRQGSLVIDDVAISSYAVMRDEWIAHASFAPVRWYS-----YIRHNMLG----------------------------------IVDTNTGQEQRVHWYTQRLYKLGKYVMSDRLFLGFDV-----  411
        Lv_hh APVTRQGSLVIDGVAVSSYAVMRDEWVAHASFAPIRWYT-----YISHMLGI-----------------------------------TDTDTGQEQRVHWYTQGLYKLGKYVMSDRLFPGFDV-----  410
        Ts_hh APMTLEGNIFVDDVLASNYAGTSYETLAHVSMAPARLYW-----NVASTIFE---------------------------------QLGPTTAPTHYHIHWYARWLWTLADNVSTFVGIPSPLDYFPRP  421
        XC_Hh --------------------------------------------------------------------------------------------------------------------------------  186
        Ag_Hh APLTGEGTIVVDSIAASCYALIDSQTVAHWSFLPYRLAE-----KVSALFDR----------------------------------TDSLSLPRHEGIHWYAKSLYTIKDYLIPSNWLYH--------  395
        Dh_Hh APLTREGTIVVNSVAASCYAVISSQSLAHWGLAPMRLLS-----TLQSWMPA----------------------KGQLRTAQDKSTPKDATAQQQNGLHWYANALYKVKDYVLPKSWRHD--------  422
        Dm_Hh APLTREGTIVVNSVAASCYAVINSQSLAHWGLAPMRLLS-----TLEAWLPA--------------------------KEQLHSSPKVVSSAQQQNGIHWYANALYKVKDYVLPQSWRHD--------  421
  Bf_AmphiHh APLTVHGTVVVDNVAMSCYALIESQALAHWVFAPFRLYY-----QLTSSLWD----------------------------------GPSHDQTLQEGVHWYPSFFYRYGISLVEPTLLHPTATDS---  415
        At_Hh APLTREGNLVVDGVVASCYAIIEDQALAHFAFAPVRLID-----NV--WEAT----------------------LHLLRTMHILRYRESRTIPPHNGIHWYANFLYSIAHKLIPED------------  404
       Cap_Hh APLTAIGTIVVNDISSSCYAHVQSHAFAHAFLAPVRWHY-----QVLPVSDS-----------------------------------------PQEGVHWYVQLLYDISTYVLPSKMVFSPS------  391
        Gb_Hh APLTAAGTVVVDGVLASCYAVVGSHSLAHWSFAPVRAWH-----WLTAWGHA-----------------------------APDYAHPPPPARAAPGVHWYAKALYSLGQVLLPGTMLYK--------  405
       Mm_Dhh APLTAHGTLLVNDVLASCYAVLESHQWAHRAFAPLRLLH-----ALGALLPG--------------------------------------GAVQPTGMHWYSRLLYRLAEELMG--------------  396
       Hs_DHH APLTAHGTLLVNDVLASCYAVLESHQWAHRAFAPLRLLH-----ALGALLPG--------------------------------------GAVQPTGMHWYSRLLYRLAEELLG--------------  396
       Dr_dhh APLTEHGNLFVDGVLASNYATFQDHGLAHTVFWPFRVLFVFFNKEMEEDLQRVAVPYICSTNQTILTSVMHSRLSSVFKWQDATRAEMENAFLQQKEVYWYARLLHTLGRIFLDPQRFY---------  447
       Tr_fhh APLTEAGSVFVDGVLASSYALVEDHQLAHWAFGPVRLLS-----SVSQLLWAEPEERSDGSKTPLQPHALVRGDRKVCARNSTSVRSEAGPRGRTSEVHWYAQLLHRLGWIVLNPDLFHP--------  442
      Dr_ihha APLTSHGTVVVNGIVSSCYAAVDQHWLAHWAFGPLRVLY-----N---WGGP--------------------------------------VGHQVTGIHWYSSLLHWIGTQVLDPAHFHPWSMMDNDR  413
      Dr_ihhb PPLTAHGTVVVNDVLTSCYAAVNRQRLAHWAFAPLRLLY-----S---WTGP--------------------------------------DQVLKNGLHWYSQVLIGLGKLLLDSELFHPLALEATER  412
       Mm_Ihh APLTRHGTLVVEDVVASCFAAVADHHLAQLAFWPLRLFPSL---AWGSWTPS-------------------------------------------EGVHWYPQMLYRLGRLLLEESTFHPLGMSGAGS  411
       Hs_IHH APLTKHGTLVVEDVVASCFAAVADHHLAQLAFWPLRLFHSL---AWGSWTPG-------------------------------------------EGVHWYPQLLYRLGRLLLEEGSFHPLGMSGAGS  411
       Mm_Shh APLTAHGTILINRVLASCYAVIEEHSWAHRAFAPFRLAH-----ALLAALAP-----------------ARTDGGGGGSIPAAQSATEARGAEPTAGIHWYSQLLYHIGTWLLDSETMHPLGMAVKAS  437
       Hs_SHH APLTAQGTILINRVLASCYAVIEEHSWAHRAFAPFRLAH-----ALLAALAP------ARTDRGGDSGGGDRGGGGGRVALTAPGAADAPGAGATAGIHWYSQLLYQIGTWLLDSEALHPLGMAVKSS  462
      Dr_shhb APVTAHGTIIVDQVLASCYAVIENHKWAHWAFAPVRLCH-----KLMTWLFP---------------------------------ARESNVNFQEDGIHWYSNMLFHIGSWLLDRDSFHPLGILHLS-  416
      Dr_shha APVTAHGTIVVDRILASCYAVIEDQGLAHLAFAPARLYY-----YVSSFLFP------------------------------QNSSSRSNATLQQEGVHWYSRLLYQMGTWLLDSNMLHPLGMSVNSS  418
        Pv_Hh APLTLNGNIVVDGVVVSCYAVVSNANLAHVVFAPVRGLHVLS--QYVPWLAP----------------------------------STHHQNFTQNGVHWYAKLLYNIGSTFLSAETLHVP-------  416
        Ob_Hh APLTKSGNIIVDGVVVSCYALINSDYIAHASFFFLRGLH-----QVTSHIPF------------------------------VSWAESPLASYAIDGIHWYAKLLYKIAPLFLDRTLLYMND------  420
        ruler .......400.......410.......420.......430.......440.......450.......460.......470.......480.......490.......500.......510........
```
