# Supplementary material for: Evolution of hedgehog and hedgehog-related genes, their origin from Hog proteins in ancestral eukaryotes and discovery of a novel Hint motif
Source: BMC Genomics. 2008 Mar 11;9:127. doi: 10.1186/1471-2164-9-127 (PMC2362128; doi:10.1186/1471-2164-9-127)
Supplement: Additional file 11 — Multiple sequence alignment of Quahog proteins. Alignment of nematode Quahog proteins. [file 1471-2164-9-127-S11.pdf]

```
                                                                                 *.*  ::*  : :  .   : : **:                 .: **  .:.: .
Ts_QUA-1 MMFIVVCVCFLINQALASTFDPAEFSI-FQCQSHEALSIQGRGTGFVKLSCAKPVACS-KTQNCKMTTFNATCSGPNEYVHGIIKL-PEHKINQICCQL-------ADVPSSLTINERCFTDSITNQTLTEVQSALNEKE   130
 BmQUA-1 --MRSVMLFVFLSL-----LIKHSAAITYRCDDDQIVVVQSFGNDTIRMHCQKPILCGLQFLKCHYNHLQTYCGGKTNFVAHIQQLMPIAPVMHTCCNLTI----NEDVQIRAHMGNDCFLYDLPDGSNGTTAEELEKKD   129
 CeQUA-1 MRRLSAILPILLLS----NFWPTVESLNYKCHNDQILVVQSFGNDTIRMHCQRLDLCGYQKLKCDYDELQPQCGGKLNFVSHVNQKGSTAPVEHTCCNL-FNP--RSHHSIPTHIGNDCFIYELPDGSSNGKKVDPAPAD   133
 CbQUA-1 MRRLCAILPILLLS----NFWPTVDGLNYKCHNDQVLVVQSFGNDTIRMHCQRLDLCGYQKLKCDYDELQPQCGGKLNFVAHVNQKGSTAPVEHTCCNL-FNP--RSHGSIPTHIGNDCFIYELPDGSSNGKKVDPAPAD   133
 CrQUA-1 MRRLCAILPILLLS----SFWHTVESLNYKCHNDQVLVVQSFGNDTIRMHCQRLDLCGYQKLKCDYDELQPQCGGKLNFVAHVNQKGSTAPVEHTCCNL-FNP--RSHHSIPTHIGNDCFIYELPDGSSNGKKVDPAPAE   133
 OvQUA-1 MFPRSMIFFLLLSL-----GMKHSATITYRCDDDQIIVVQNFGNDTIRMHCLKPTLCGFQFLKCHYNHLQSYCGGKTNFVAHLQQSTPISPVIHTCCNLTI----NEDVQIQAHIGNDCFLYDLPDGSDGTTGEDLEKED   131
 MiQUA-1 FRLITSLFIQLFNILAILNSIPQTIAISYRCEGDQVLVVQSFGNDTIRMHCQRLNVCGDVDVHCHYEKNQPACGGKANFVAHVDQPTPLAPVSHTCCEA-IPPFEEKMNEIPSHEGNDCFIYELPDPNAPPPEEGEQNNK   139
 McQUA-1 ------------------------------------------------------------------------CGGKANFVSQVDQPSPLAPITHTCCEA-MPSLEEKITEISSHEGNDCFIYELPDPNAPPPDSTEKENN    67
 SsQUA-1 MRWRSMLFNILIITFLTIIFTTQIKGLSYRCENDQILIVQSFGNDTIRMHCQKLQLCGYSNLKCTYDREQPACGGKTNFVSHVNQLTPTGKVLHTCCDMTFKN--NKSKHIIEHDGNDCFVYELPDGTTDITPGSEADII   138
   ruler 1.......10........20........30........40........50........60........70........80........90.......100.......110.......120.......130.......140

                                                                                                            QUA domain
                   :  *             .     :::: *  :     ::* :.    ** :* *   *
Ts_QUA-1 R-------LPYSYLAR----------DDYAPAFVKLFRLAHQSDGVRIVKKIKAVEHGYLITSCQLDCI---------------------------------------------------DG------------------   184
 BmQUA-1 K-------EGYALLKDINKIPEQF--TDFSGYRLRLYLLRKKEPSQFVIKGVERNEVGYRVTICTIQCQ-------------NDNHKVKAVTSDQKHADLNKQEPIINDNNLNQINFALRNLTDDGQWIIATWAEWSYKK   247
 CeQUA-1 D-------APYAVLKNPAEIPEQF--DGVTGYRLRLFLLKNKSPPTLLVKGIERRLDGYRVTICRPRCT--------------------------------SYDKVVNDN----------EGAEDGEWKAISWSSWSSSS   222
 CbQUA-1 D-------APYAVLKNPAEIPEQF--DGVTGYRLRLFLLKNKSPPTLLVKGIERRLEGYRVTICRPRCT--------------------------------SYDKVVNDN----------EGTPESEWKAISWSSWSSSS   222
 CrQUA-1 D-------TPYAVLKNPAEIPEQF--DGVTGYRLRLFLLKNKSPPTLLVKGIERRLEGYRVTICRPRCT--------------------------------SYDKVANDN----------EGAEESEWKAISWSSWSSSS   222
 OvQUA-1 K-------EGYMLLRNINKLPGQF--ANFSGYHLRFYLLRNKGMSQNVVKGVERNELGYRITICSIQCRDSVGRRKGMIYENDEKNEEKQVIRDDKHIDLNKKEQIFNDNNLNQVKLTIRNLTDDGQWLFVTWAEWSYKQ   262
 MiQUA-1 KEEEEEENEHFTLLNSIDQLPGHINPEGYH-YRMRLFLLRYKSPPALLVKGIRRLREGYRVTICRPRCR-----------------------------------------------------------------------   207
 McQUA-1 NKNDDDENEHFTLLNSIDQLPKHLNPEGYH-YRMRLFLLRYKSPPALLVKGIKRLREGYRVTICRPRCR-----------------------------------------------------------------------   135
 SsQUA-1 KKNIDVVTNGFTVLKDASQIPEDF--GGYTGYRLRLFMLRNKSPPLLIVKAIERTSGGYRVTICRPRCG--------------------------------KFNR---------------EG------------------   211
   ruler .......150.......160.......170.......180.......190.......200.......210.......220.......230.......240.......250.......260.......270.......280


Ts_QUA-1 -----TLPEGSVPPEEGILCDEACQRLLEKNRENSAVNSVEVSPTSLPEKQLTGEPVIADNALV----------------------------------------------------------------------------   243
 BmQUA-1 WSEWSTERRIEFNDLDRTKGRGHRYRIHHSNSE-SGTATGKGQQKSEGIKTSIHESSKGHRRIH----------------------------------------------------------------------------   310
 CeQUA-1 WSTWARHAFNKAAAEGGEAAERIRTRMPIGEKTVAGAAGATGAAGSDKSNINIHVESNGNNNNSFEGGRSSSEKS---DGQLNREISGSSE------AGAG-GKGGAGADGAAGS-GAGAGAGAGTNGNINITVHTD--G   349
 CbQUA-1 WSTWARHAFNKAAAESGGSADRIRTRMPIGEKA-TAAGVPTGAAGSDKNNINIHVESNGNNNNSF-GGNGSGEKNGSGENGLNREGSGANGGAGADGAGAGRSGAGAGADGA-------AGAGAGTHGNINITVHTD--G   351
 CrQUA-1 WSTWARHAFNKAAAES-GSADRIRTRMPIGEKAGTGAGGATGAAGSDKNNINIHVESNGNNNNSF-GG-GSSEKT---DSQLNREVSGSSEASNGDGAGSG-GGAGAGKAGADGEVGSGAGAGAGTHGNINITVHTDGAG   355
 OvQUA-1 WSEWSTIHKIELNELDGTK-RRNRCGIHETDTE-IKTNTEKGQEKSERIKMFMQKLNRVREGFR----------------------------------------------------------------------------   324
 MiQUA-1 --------------------------------------------------------------------------------------------------------------------------------------------   207
 McQUA-1 --------------------------------------------------------------------------------------------------------------------------------------------   135
 SsQUA-1 ------------------------------------------------------IISQGESISTF---------------------------------------------------------------------------   222
   ruler .......290.......300.......310.......320.......330.......340.......350.......360.......370.......380.......390.......400.......410.......420


Ts_QUA-1 --------------------------------------------------------------------------------------------------------------------------------------------   243
 BmQUA-1 --------------------------------------------------------------------------------------------------------------------------------------------   310
 CeQUA-1 KSGGNAVAVANANVTVNGA-GGVSTTGTGAQTGNESGLG----GSAGTDKAGGKKGGHGDSGDSGN-NKNKDNGKGKGKGKN-----DEEDEEDNGDEDGNGKG------------------------GNGG---NPKGE   451
 CbQUA-1 KSGGNAVAVANANVTVNGANGKVDTTGTGAS------------GNGAAGGNGGN-GGHGDSGDSRDAKKDKDHGKGKGKGDG-----DGDSDGGAGNGSGNGSGKDKGDGNGD---SDGNGNGSGGSGGSGDKKPKPAGE   470
 CrQUA-1 KAGGNAVAVANANVTVNGVNGGVSTAGTGASTNGNGGAGGADGGNGGAGGKGGNGGGHGDSGDDGD-KDGKDHGKGKGKGKGKNGGDDGDDDNGDGNNGGDGDGDDNGKGSGKGSSGKGSGKESGKDKGSGGNK-KPAGE   493
 OvQUA-1 --------------------------------------------------------------------------------------------------------------------------------------------   324
 MiQUA-1 --------------------------------------------------------------------------------------------------------------------------------------------   207
 McQUA-1 --------------------------------------------------------------------------------------------------------------------------------------------   135
 SsQUA-1 --------------------------------------------------------------------------------------------------------------------------------------------   222
   ruler .......430.......440.......450.......460.......470.......480.......490.......500.......510.......520.......530.......540.......550.......560

                                                                                           .
Ts_QUA-1 ----------------------------------------------------------------PRISSAGAGGGGAVQADGN---------------------------------------------------------   262
 BmQUA-1 --------------------------------------------------KKKHSSDCDDDDDDESYGSKEVGKIDVTKEGGK---------------------------------------------------------   343
 CeQUA-1 WDDGDGDEDDDGTDGGSKESGNNGKGKGKGSGDGDGNRNGNGDGNGRPKGDGNIKINIHSPDDNDLLEKDENGPNGKG-GAGN-----------------GNGDGDKDNNGKGNGTGDGDGDGNGNGNGLTGDG------   567
 CbQUA-1 WDDGDGDDDESGKGTGSNEAGNGKGGD----GDGDGSKGAGGSGG---KPDGNIKINIHSPDDNDLLEKDENGPGGNGKGDGDKDGGAGGAGGSDKDGGDGNGGSDKDGGAGGDGNGDKDGNGNGNGNGLTDGGDGNGGS   603
 CrQUA-1 WDDGDGDDEDGGAGNGGSNE-----SD----GDGKGGKGGDGDG------KGNIKINIHSPDDNDLLEKDENGPGRKGKGDGD--------------------------GAGAGGAGKDNGNGNGNGNGKDNG-------   585
 OvQUA-1 --------------------------------------------------D----------CDDDEDYTDELKKNNVEQEEQK---------------------------------------------------------   347
 MiQUA-1 ---------------------------------------------------------------------------RVIIEEDE---------------------------------------------------------   215
 McQUA-1 ---------------------------------------------------------------------------RVVIEEDE---------------------------------------------------------   143
 SsQUA-1 --------------------------------------------------DKDISI-----PKNKIIHSNENHCEKHTTEEGH---------------------------------------------------------   250
   ruler .......570.......580.......590.......600.......610.......620.......630.......640.......650.......660.......670.......680.......690.......700
```

```
Ts_QUA-1 --------------------------------------------------------------------------------------------------------------------------------------------  262
 BmQUA-1 --------------------------------------------------------------------------------------------------------------------------------------------  343
 CeQUA-1 -------NGTGDGDNNESGNGNGDGSDKNSGAGAGTKPENREGGDGNGNGTGDGNGDGNDNGNGSKGLGTGSGDGKGEGNKSGTPGKSDGKEDGAGSNGSGNGKEGDGNKSGGSGKGGAGNGKSGDGSGDGKNNGNG-GT  699
 CbQUA-1 KNGPRDNDGNGDGNKETGAGGNGNGDENGNGAGA--------GGNGNGDGNGGSNRP-NGNGAGGDGNGTEAREG-GDGNGGSNGGSNGSGDKGAGAGGKGKGDGSVGDK-YDKGSGGAGGDKDGKGAGGSGADKDGNG-  731
 CrQUA-1 -------DGNGNGT------GNGDNDGNGNGNGV--------TGDGDGNGNGGSKGP-N----GGDGNGTGARET-GDGDG-----------DGKGPNGSGTGDGGAGGA-GGKGKGGGKGDKDGKGKGSGSGDGNGDGK  686
 OvQUA-1 --------------------------------------------------------------------------------------------------------------------------------------------  347
 MiQUA-1 --------------------------------------------------------------------------------------------------------------------------------------------  215
 McQUA-1 --------------------------------------------------------------------------------------------------------------------------------------------  143
 SsQUA-1 --------------------------------------------------------------------------------------------------------------------------------------------  250
   ruler ......710.......720.......730.......740.......750.......760.......770.......780.......790.......800.......810.......820.......830.......840

                      .
Ts_QUA-1 ---------LQDRHATN-------------------------------------------------------------------------------------------------------------------LASSAS  276
 BmQUA-1 ---------IKERFGNI-----------------------------------------------------------------------------------------------------------------------PDND  355
 CeQUA-1 GDGKDK---NGKGSGSG---DNDKSGTRAAGK-GNAEGNGKG-------------NGNDGKGSGSGDGSGAG-GKGDK---------SDSESGNEADGKDGKKNEGAGGEAAAGSGGANKG---------GSDGDDDDVDV  801
 CbQUA-1 GNGKSN---KGDKDGNGKGSDKDKNGKDGAGKAGNGDKDGKGGKKDD---------SGKDGAGSGKDKDAGKGKGAGDKDKK------GGAGAGGKGAGGAGA--GGKGAGAGAAAAGKGTG---------GGD-DDDDVDV  843
 CrQUA-1 GDGKGKGKGKGDKDGNG----KDKNG--GAGGAGAGKDNGKGAGKGDGKDKAGAGAGKDGAGAGNGKDAGKGKGAGDKDKNAGGAGGAGAGAGGKKDGGAGGAKAGTGAGVGAGAGGNGANGGKNGKGKGGD-DDDDVDV  819
 OvQUA-1 ---------LGEDSKEL-----------------------------------------------------------------------------------------------------------------------GYDY  359
 MiQUA-1 ---------RQEK-----------------------------------------------------------------------------------------------------------------------------------  219
 McQUA-1 ---------PQEKEEDV-------------------------------------------------------------------------------------------------------------------------------  151
 SsQUA-1 ----SK---KNENNSNA-------------------------------------------------------------------------------------------------------------------------------  260
   ruler ......850.......860.......870.......880.......890.......900.......910.......920.......930.......940.......950.......960.......970.......980

Ts_QUA-1 SSEHSAPGQWQPCSYCGPFIP----------------------------------------RAAVNSAINPIPSATNPIG-------------------------------------------------------------  316
 BmQUA-1 SKEKL---NQEKRKFPALDIQENMKSELDPHKTNGTGKKEELF---TVRFGEIGNQTKDGTKKIEEGTEKNQGDSSGNNANATKATESERKDHSNANKEGGGDDLTGKKITLTQKTDKSAGNQQKEKKHGELKDQLKSGS  489
 CeQUA-1 TDVEVGTKPLTGTKLEELLAKLPNETADGNATGDGNE--FGTVQTGAKHNAESS---ASGIPLVQARSNTVNGG------APVPPAPG-SGATGSGTS-GSGTSESVTNGSGATESGSTGSGTTGTGTSGTGSSGTGASA  928
 CbQUA-1 TDVEVGTKPLTGNTLKELLAKLPNETAD-KDLGNGDE--LTNASLNRNQHAEGSTGTAPGTSAAAASAPDTTGGTGGTGTTANAANTG-GGTTGSGAT-SSGTTESGSTGS---TGGSGSTG--GSGSTGSGSTGTGAST  973
 CrQUA-1 TDVEVGTKPLTGSKLQELLAKLPNETAD-KAAAKSDEDALTPKTLKRKQNAEAA---APGTTSVSSGTSGTSGTSGASSNAAAADTSGTSGTAGSGTTGTSGTTATGTSGTGTASTGTASTG-TGTAATGTGTTGTGSTG  954
 OvQUA-1 SKKELAIKSAEKQNFSELDVEENLKSELNSHKINETEEEMDLFVNPVTRREEIENQTKDHFEKMKENTLSDDGNNLKTP--TIRATIHCNISITKEVRSGHDDISSIKKTSESEEKDISTIKHRDDVDL-MNT---SQSY  493
 MiQUA-1 --------------------------------------------------------------------------------------------------------------------------------------------  219
 McQUA-1 -----GLKEWITQKLTGIESQ-------------------------------------------------------------------------------------------------------------------------  167
 SsQUA-1 --------------------------------------------------------------------------------------------------------------------------------------------  260
   ruler ......990.....1000.....1010.....1020.....1030.....1040.....1050.....1060.....1070.....1080.....1090.....1100.....1110.....1120

Ts_QUA-1 -------------------------------------GGCFSADMHVRTTHSQIRMDQLQLDDIVFVDPVEQQPIFS----MLHHDPAAEVDFIIIKTETNRSLSLTPNHLIPIVPCRRGI---LPAEKLEATVNRYSKF  412
 BmQUA-1 TQSSKPVNQTRKQELGHDPPAIPVAAGHHVGANPMPAMNCFSADTKVYTQNGEKTMKDVVVGDFVLVPVSKSQMRYERVEMFYHREPETRAKFVVLETESGRKLSLTELHLLPLGDCKEMHESMTDTTDIVDQWLRKSKF  629
 CeQUA-1 ARTSSIAGDAPQA--------AVLADTPGAAGAAGGGRSNCFSADSLVTTVTGQKRMDELQIGDYVLVPSSGNVLKYEKVEMFYHREPKTRTNFVVLYTKSGRKLSLTGRHLLPVAECSQVEQY-TMNPDGIDVAMRESKY 1060
 CbQUA-1 AQRSAVAADTPAAA---AADAVAADAAGGGGAGGGGRSNCFSADSLVTTVTGQKRMDELQIGDYVLVPSAGNVLKYERVEMFYHREPKTRTNFVVIFTKSGKKLSLTGRHLLPVAECSQVEKY-TMNPDGIDAAMRESKY 1109
 CrQUA-1 T--SAVAADTAAAD---PAAAVQADAAGATGGGGGGRSNCFSADSLVTTVTGQKRMDELQIGDYVLVPSAGNVLKYERVEMFYHREPKTRTNFVMIFTKSGKKLSLTGRHLLPVAECSQVEEY-TSTPDGIDAAMRESKY 1088
 OvQUA-1 LNEKQLLAE-----------------------------------------------------------------------------------------------------------------------------------  502
 MiQUA-1 --------------------------------------------------------------------------------------------------------------------------------------------  219
 McQUA-1 --------------------------------------------------------------------------------------------------------------------------------------------  167
 SsQUA-1 --------------------------------------------------------------------------------------------------------------------------------------------  260
   ruler .....1130.....1140.....1150.....1160.....1170.....1180.....1190.....1200.....1210.....1220.....1230.....1240.....1250.....1260
                                                                                     Hog domain

Ts_QUA-1 AHKAEQDECVL-MAYGGLVKTEKIVAISQRRLRGIFSPLTEKGTIVVNDFVVSCYSTCESHALQKLFHNSIRHISRMLRNAL------FIQLPIYLKSLYKLMHWTVSMTVA  517
 BmQUA-1 AHRARIGDCVFTMTSNHELQVDRIVKVGRQYLKGIYSPMTVEGSIVADGILASCFSQVESHFSQKLVYDFLIFLYRIFGPLMQSLDEPIQHLPTFIDSIHHLGRFAVPFVKY  741
 CeQUA-1 AEKARKGECVLSIDESGEVIADEIVRVGRMTNVGIYSPMTVEGSLIVDGVLSSCFSHLESHSAHKLIFDFIYYVYNAFGLLNTNHVD-LQPIPTFVSFAQYLSKTVLPFS-- 1169
 CbQUA-1 AEKAKKGECVLSIDASGDVIADEIVRIGRMTSTGIYSPMTVEGSLIVDGVLSSCFSHLESHSAHKLIFDFLYYVYHAFGLLNTNHVE-LQPIPTFVSFAQYLSKTVLPF--- 1217
 CrQUA-1 AEKAKKGECVLSIDEFGNVMADEIVRIGRMTNVGIYSPMTVEGSLIVDGVLSSCFSHLESHSAHKLIFDFLYYVYHAFGLLNTNHVE-LQPIPTFVSFAQYLSKTVLPFS-- 1197
 OvQUA-1 ----------------------------------------------------------------------------------------------------------------  502
 MiQUA-1 ----------------------------------------------------------------------------------------------------------------  219
 McQUA-1 ----------------------------------------------------------------------------------------------------------------  167
 SsQUA-1 ----------------------------------------------------------------------------------------------------------------  260
   ruler .....1270.....1280.....1290.....1300.....1310.....1320.....1330.....1340.....1350.....1360.....1370..
```
