# Supplementary material for: Evolution of hedgehog and hedgehog-related genes, their origin from Hog proteins in ancestral eukaryotes and discovery of a novel Hint motif
Source: BMC Genomics. 2008 Mar 11;9:127. doi: 10.1186/1471-2164-9-127 (PMC2362128; doi:10.1186/1471-2164-9-127)
Supplement: Additional file 14 — Multiple sequence alignment of non-metazoan Hog proteins. Alignment of non-metazoan Hog proteins including also the ones which are only based on EST fragments. [file 1471-2164-9-127-S14.pdf]

```
    jJl_Hog1 ----------------------------------------------------------------------------------MGKSIILACAVVACLTAAVLSQSNNHIGCNINYSGSMDNGREFNCGDVVLSANDQIQGQGFADSIDGT    68
    jJl_Hog2 ------------------------------------------------------LLVTINSSVRIPGIEIRRETKTVWQFVLQEVAGWALCVWLQPLLWWLFCWPFATLALPIRWCAHGHSKATISAGT---KAAAALPPSSLDPRIEFA    93
    jJl_Hog3 -------------------------------------------------------------SVRCENSRRR-----------------AVCVWLQALVWGVLWWACATVAVPIRGCARGHSKATISAGT---KAAGAVPPSSLDPHIEFA    69
     cBn_Hog ------------------------------------------------------------------------------------------------------------------------------------------------------
  Xx_104K18 ------------------------------------------------------------------------------------------------------------------------------------------------------
     pSm_Hog ------------------------------------------------------------------------------------------------------------------------------------------------------
     pPp_Hog MIRMLLPLAFLLVLTHQAGVLVGAQLAISVTTFGPPHADGTPNTFIG-TGSQIASSLLGSTTKALMWGVVSSSDVNAVCDQIVSTGALTFSATLTATTGETETLQQTITHVGGCRAAADGSGTQAVMVDNNGRQSAVVCPNNAGSTGCGI   149
     pSl_Hog --------------------VPGIPRDLQATTPGLAQ-KGKPNTFIGTTGDQIASSLLASKTPAMLWGSVPASAITNICQKIVKTGGLQYTAKISGDWGGSESLTLKVTATGGCGAAPDSSGVQFVFTDNYGRKSAIVCPRDAGKSGCGV   129
    rGc_Hog2 ------------------------------------------------------------------------------------------------------------------------------------------------------
     rPh_Hog ------------------------------------------------------------------------------------------------------------------------------------------------------
    rGc_Hog3 ------------------------------------------------------------------------------------------------------------------------------------------------EPLAPA     6
    rGc_Hog4 ------------------------------------------------------------------------------------------------------------------------------------------------------
    rGc_Hog6 ------------------------------------------------------------------------------------------------------------------------------------------------------
    rCc_Hog2 ------------------------------------------------------------------------------------------------------------------------------------------------------
     rGj_Hog ------------------------------------------------------------------------------------------------------------------------------------------------------
    rGc_Hog1 ------------------------------------------------------------------------------------------------------------------------------------------------DEGTDG     6
     rCc_Hog ------------------------------------------------------------------------------------------------------------------------------------------------------
     rPy_Hog ------------------------------------------------------------------------------------------------------------------------------------------------------
    rPy_Hog2 ------------------------------------------------------------------------------TAIVQDGARCAGPGQFALVSPAVLENLDDLSGDFVDPLKEYL-ASEGLGSAFTGFVRTIFNKIVGAPVYMGV    71
    rPh_Hog2 ------------------------------------------------------------------------------------------------------ENLDGLTGPFEEIVRETLGAVDGAASTFSRFVRTVFSAITGAPVYLGV    48
    rGc_Hog5 ------------------------------------------------------------------------------------------------------------------------------------------------------
     aAc_Hog ------------------------------------------------------------------------------------------------------------------------------------------------------
     aKm_Hog ------------------------------------------------------------------------------------------------------TIGTLLQGLHQNHHRDVVSFSEFLTPRIQTGFAKTAAQAGQDNFKGAN    48
     aAt_Hog ------------------------------------------------------------------------------------------------------------------------------------------------------
 aAt_Hogfrag ------------------------------------------------------------------------------------------------------------------------------------------------------
    aAt_Hog2 ------------------------------------------------------------------------------------------------------------------------------------------------------
     aCp_Hog ---------------------------------------------------------------------------------------MKVNNIKIICFLWLTILVELFWFNKESRKNVDLFHSNLSFIRVRTKSIFDTFSGMSAGGTGFR    63
     aCm_Hog ------------------------------------------------------------------------------------------MEFQKFLLIYYIYLWEIIYRIFPKLIIDQQIIINYSFVRLK--VKGILSSLQDFDSQSFR    58
   crGt_Hog1 -------------------------------------------GLSMLNGSAAGGNSSAGGQNSSSASGGSNDFVCSVANIKALHAAVQPKWDACKSNPNYEEPNKGAKRRLLEWVWEPKAGERMTAKPRYSPDHYRLAIKADRSRRHAA   107
   crGt_Hog2 ------------------------------------------------------------------------------------------------------------------------------------------------------
    hPh_Hog3 --------------------------------------------------------------------------------------------------------------------RSDSDGDSSGEDSSGEETSGEKTSGEGGSGSSLT    34
    hPh_Hog2 --------------------------------------------------DDDDSPVYDDDGTLIWQCENVTKFVCKPFEEDDNNDDDDDDDRRRRLGKDDDDDKKKDDDDDKKKKNDDDDKKKKDDDDDDE---VEVDGDFGSGEKNLT    97
    hPh_Hog1 -----------------------------------------------------------DDGTLIWQCENVTKFVCKPFEVDDDDDDDDDDDRRRRLGKDDDDDDK--------KKKDDDDDKKKDDDDDDDANNVEVDGDFGSGAKNLT    83
  fGm_GmGIN1 ------------------------------------------------------------------------------------------------------------------------------------------------------
  Mo_hoglet ------------------------------------------------------------------------------------------------------------------------------------------------------
       ruler 1.......10........20........30........40........50........60........70........80........90.......100.......110.......120.......130.......140.......150
```

```
jJl_Hog1   FSVTL--LINGTSVGTPRQIVLDFKPCSGD--GCQTPNTGCQRIGIDISGNA----------ILKVTSGSGSVSNVWMNVHPCGRSQGGGPGCFADGQTVQLSTGQS---VLVETGKIGDRILTASASG------------AGAGYSPVT   189
jJl_Hog2   CSPTRSCWVGGSASGT-FAVLVGSGSSSGQVVGSETVNTNCNRCNLQ-SGCVQPPVDGNYDLVMSITGVVGSLSAVSVTATL-EKCESGGVGCFADGQSVVLSTGES---KSIELLQVGDRVLSSLPSS-----------SCQLAFSPVT   226
jJl_Hog3   CSQTRSCWGGGSASGT-FGVRVGSRSSSGQVGGSESVNTKCNRCYLQ-SGCVQP------------------------------------------------------------------------------------------------   121
cBn_Hog    ----GDFDIGIQGPQTMIWATSGSDAVAQ---------------------------------HAANDRSAGMVDLSADGMVEDDGDDDGDDVCFSGSSMLTLKSGEK---KAIKDITIGESVVVTAED--------------GSLTHGKV    96
Xx_104K18  -------------------------------------------------------------MCCCCAAWCAAKCACCACDCL------KKLLCVDKEALAIMSDGKL---KPLAELQIGDKIKTLDSA--------------GNMVDTEV    66
pSm_Hog    -----------------------------------------------GIFLG-FHSVEFSM-----AHTFFATSLFAVLASVVLATN---TSCFPGDATVQMYNGDL---KLMRDLEVGDKVAVSKN------------------VFSDI    74
pPp_Hog    YLTGPDVTALYNQGARRRLLAR-----NLLARKLLQGDNCQTGAAKGALTGAITSMEACIPFLGPLYPFCMAGVIGVATAGGAAVGCAEANGCFPGDATVLLAGGEV---KPMTSLALGDKVAVRRHDG--------------GLDYEDI   277
pSl_Hog    YMTGSDVDSIYNKKLELEQQTLLNSSDHEAARRILLGD-CREEAGKGAIGGAITGVLGTGGCLGL-------------------------------------------------------------------------------------   193
rGc_Hog2   ----------------------------------------------------------------------------------------------------------------MADLQPGQHVHHDEH-----------------GASSPV    21
rPh_Hog    ---------PTPVPKATGGDGGE---------------------------------------------------------------------CFPAATTVRRRYGGVVSTVRMDDLRLGDEVLAAGG------------------VYTRV    54
rGc_Hog3   ETSSPTTQPSSAAETAVSIDDENET---------------------------------VEESPVGEGNGQSEDAAETGD--DEDVDDDDDDVCFSASALVELFDGSR---KRMDEVEVGDRILVGHE------------------EYSDV   100
rGc_Hog4   ----------------------------------------------------------------------------------------------------------------------------------------------TKDEFSDV     8
rGc_Hog6   ------------------------------------------------------------------------------------------------------------------------------------------------------
rCc_Hog2   ------TSGGVPGESEPAASDDLSD-----------------------------------------------LSPSPSP--DDEDEDDDHSVCFPANALVELENGAT---KMMSQVQLGDRVRVGPN------------------DFSDV    74
rGj_Hog    ---------------------------------------------------------------------------------------------------------------------LGDRVHVGGG------------------EFSPR    15
rGc_Hog1   GTDEGTTTGGLPISGEQGGPEGDIT--------------------------------------------NIEPPETPEE--PTETPEDDDPVCFPADGTVLLEDGSV---KRMDEVEIGDSVMVGEK------------------QFSEV    89
rCc_Hog    --------PPLPSAAASLLFQQEAT---------------------------------------------------------------SPAVCFPASARVQTADGRA---PRLDALAVGDRVLVGVRR--------------GRRVYSEV    62
rPy_Hog    ---------SSPGESPSDGSGDGSG-------------------------------------------------DGDGDADESPESTDDDSECFPATASVEVEGGAT---KAMADLAIGDRVRAADG------------------SFSDV    71
rPy_Hog2   ATEEDRTCGDREVEGRTFVAAMEVNESVTFAGETVLPEGRHLITYTIE----SNPLSQADNPLCLYSGSEVTGGAGGEG--GGNGTIVDEPACFPATATVELSTGAT---VAMADLAVGDRVRVAAGA--------------GAAAFSPV   198
rPh_Hog2   ATQVDRTCGDREVEGRTLVAAMSVNQTVQFVGSLSLPAGRHLLTYTIE----SNPISQVDNPLCVYTGAAIKSGDVAEG--ANGTAADNNPACFPAAATVELSTGAV---VAMADVAVGNPVPVAAGA--------------GPAAFPPV   175
rGc_Hog5   ------------------------------------------------------------------------------------------------------------------------------------------------------
aAc_Hog    ----------------------------------------------------------------------------------------------------------------LQDLRVGDRILGLDHSTG-------------RQSFSEV    25
aKm_Hog    DNLEEKIGIKEAGKLKYSLRTGRTVYLCGDNADCCKESCKKLAHPNPR----RDESRASQGSAELASLGATDNNEQADC--DKFCTAEFSLLCFPGDSTVVVRDRGR---VPLAELKVGDAVLSVRRRHAPTKEVDEISCDGWELYFDEV   189
aAt_Hog    ------------------------------------------------------------------------------------------------------------------SDAVGQ----------------------WELLXDTV    14
aAt_Hogfrag ------------------------------------------------------------------------------------------------------------------------------------------------------
aAt_Hog2   GTRNYELEITYSCSAYDATDKVCKG-------------------------------------------------------WTQRGTMSSAPACFPANATVITRGGPK----PMSKVALGEELMGLDHRTG-------------LVTFSPV    78
aCp_Hog    SGWGNVISNAISLAFPSSCVGQLIN-------------------------------------IGYLSNELDEDDYPYNP--NCGAQSQSNNLCFPGNSLVITRERGE---IKLEDLRIGEYVLIRDLNT-------------MKFKYSKV   158
aCm_Hog    SDWGNVISASLQVSFPTSCIGDIIN-------------------------------------MGYVSTELDEDDYGFNT--NCYANSETNNLCFPGENIVLSKTRGY---IPIKELKIGEHILTFDYKS-------------LKTKYSEV   153
crGt_Hog1  KQSDTSSELGSYISLFSSLAGNSSNSSASQGSANAGSASMSSCEKNAL----SGACEKLAACKDPVCFSYYNEPEIEQLCGMCSMGSQAWFGCFAEDSKVSVEGKGS---VAINEIQIGDMVLSADASG---------------APQHSR   235
crGt_Hog2  ---------------------------------------------------------------------------------------------------------------------------------------------------YRR     3
hPh_Hog3   PYDKCRRAVSSVCGRKFQLDEARDKLREALIDELGLAAIDLDSLPSEL-PYAIKEVIETERFVERCTEECTRQCRRIVVTILQWV---NRRSCFPAHAQVSVLRDGAQQNVPISQVGVGDLVMARNG-------------------FEPI   161
hPh_Hog2   DYEKCELAVADVCGRDLDANEALSILEQSLLDNLGVAIDNVAYLPYDENLSKKKNKKLQKKAEKTCRNVCKDECRSAEVLLNEWVADTGLSSCFPAHAQVTVMRDGAQVASPISQVGVGDLVKTTDG-------------------FETI   228
hPh_Hog1   DYEKCELAVADVCDRELNASAALSILEQSLLDNLGVAIDNVEYLPYDTSLSKKKNKKLREKAEKSCKRTCKNECRSAEVLLNEWVADTGLSSCFPAHAQVTVMRDGAQVASPISQVGVGDLVKTTDG-------------------FETI   214
fGm_GmGIN1 ------------------------------------------------------------------------------------------EGCFAADSKVILKNGKV---TKISELVIGDYVCCGFEDG--------------KQVYSEV    43
Mo_hoglet  ------------------------------------------------------------------------------------------FGCFSQSSTVVVEGRGR---ISITDVQPGDMLVDGSAAG-----------------TSQF    40
ruler      ......160.......170.......180.......190.......200.......210.......220.......230.......240.......250.......260.......270.......280.......290.......300
```

Hog domain

```
    jJl_Hog1 WIMNHLTTKEVLEFSLQARNGTT----------NASLQVTHFHNML----------------------------------VEGKGEVQAQDVHQGDTMR-------VMVENCFVEALVTKIE--K-LSSQKYVRLVVT--ESGTIVVNGV  283
    jJl_Hog2 WIMKHSSPKDVLAFTLESDRGVT-----------AT------------------------------------------------------------------------------------------------------------------  251
    jJl_Hog3 ------------------------------------------------------------------------------------------------------------------------------------------------------  121
     cBn_Hog VFLPHTKGNNHKIRFLELTTNKA-----------SRIQLSRKHLIL--ANDCGK--------------------------DSKFELYHASDVTLAMCLS---------GVDGPEKVIQ--IG--R-FRGEGIYSV-ITDHSHGLIVVDGL  192
  Xx_104K18 IMFAH-VGNDQKI-MYNIITWSG-----------KSIRASPNHLIPVASS-NEY--------------------------KYAKN------VTEQDMIN----TLDLDTKRMCAEQVRA-IA--L-EECTGYVAP-IT--MSGDFLANGV  159
     pSm_Hog YAFGH-KDADVISEFIQVHTA------------SDMIELSEGHFIPVDAK-------------------------------GKLVYKRAKDLQVGETLWG---------SSQITELSK--------VEKLGLYNPFTL---SGNIMVNNV  160
     pPp_Hog YAFGH-KDALAAANYVQLSLKPVGANMSDPALESTKLELTPLHFTVILS-------------------------------GSEITYKRAQDVRVGDMMWAQ-------ASTHAAELSPYLVTDISTVEKQGLYNPFTL---GGTIIVNGV  385
     pSl_Hog ------------------------------------------------------------------------------------------------------------------------------------------------------  193
    rGc_Hog2 FFFTH-RTAKPELWFYSISTASG-----------HAVSMTAKHYLY--AD-GRL--------------------------TAAHA------VQVGQMLR---------TKAGESAVTS--VK--R-VRDTGLFAP-HS--MHGDLLVDGI  107
     rPh_Hog YLWSH-ADAAAVTTFVRLVATRADGG-------LHTLLISAGHLLPTVVRGGSG--------------------------DRGPTLTAAVSLAVGDTLF---------AADGSPLVLTAVVP--G-VAAAGLYHP-HT--TAGNLVVDGV  155
    rGc_Hog3 FMFTH-RSAHVPYQFVQLTTSSG-----------KSMKVTRGHYVY--LN-GML--------------------------KMAST------AKPGDTVA---------VGSGGHEEVVD-VR--R-VWGKGLYNP-QT--IHGNIVVDDF  187
    rGc_Hog4 YMFSH-RDVTAADPSYVRIVTAE-----------TSVTMTTGHYVP--VMKNVT--------------------------GRASTFVQAEWVQVGDLVQ---------LASGQWAVVSD-VF--A-DEASGLFNP-HT--LAGTICVDGV  102
    rGc_Hog6 --------------------ANG-----------SELLASRSHYVF--AG-GAL---------------------KTAARVQLRERLRVFDAR-GERAET---------------VVS---K--RLLLRRGLYNP-QT--ASGALLLFAP   71
    rCc_Hog2 FMFTH-KTAAIKYSFVTLATQSG-----------HTLSLTKGHYLY--VN-GVV--------------------------AAAKT------VRSGDFIT---------LADGTTSSVTQ-VG--T-EIA---------------------  143
     rGj_Hog VLFSH-RDASTRHAFVQVSTACG-----------RSVAATHGHYLY--VN-GRL--------------------------AIAGS------VKVGDRLQ---------DAHGAPLDVTK-VE--V-VTKTGLYNP-QT--LHGDIVVDSV  102
    rGc_Hog1 FMFSH-RLASVKHRFVRMELANG-----------LSIEATTGHYVY--VN-GRL--------------------------LAASS------VKVGDRME---------LVSGDRVAVSR-VS--V-VKKSGLFNP-QT--LHGDIIVNGV  176
     rCc_Hog FAFTH-ADPHATHAFVELATRPRN----------HTLRLTEGHWLL--AD-GAW--------------------------MPARA------VRVGMALQ--------DGWTGEARRVSG-VR--V-VGDSGLYSP-QT--YHGAVVVDGV  151
     rPy_Hog FLFTH-ADPTAKSTFVTLTTAAG------------VLTVTPGHYVL--VNEGVR--------------------------TAASA------VVVGDVLSYMPPAGCAAAAAATPAVVTA-TG--R-VVAKGLYNP-QT--LVGSIVVDGF  167
    rPy_Hog2 YTFTH-RSSGGAHPVVTATTRSG-----------HALTVTPGHLVY--IN-GRA--------------------------APLRS------VRVGDALD--------VAADAASSVVTA-VS--T-GTSAGLYNP-QT--LQGDIV----  282
    rPh_Hog2 YTFTH-RTAGGAHPVVTATTRRG-----------HSLTATPGHLFY--FK-GHA--------------------------TPMRS------VRVGNPLN--------VAADPTSSVVTA-VS--V-GTAAGLYNP-QT--LQ--------  255
    rGc_Hog5 --------------------------------------------------------------------------------------------------------------------------------------P-QT--VQGDIIVNGV   13
     aAc_Hog RGWLH-RNPMQTMSMMELHTEDG-----------LAVVTSPGHMLALSNDSSAIDG------------------------REALSYRRASDIRPGDTLVS---------ANGSSIAVEETMS----TKMEGLFAPLTMH--SNYFVGSGE  124
     aKm_Hog LAFLH-SDASMEAEFLQVRHEAG------------QLHLTPNHLLFARSRTASA--------------------------SSAPAPVFARDVCAGDHLL----APWIDGSFSEPEVLE--VT--K-VRRRGVYAP-LL--ESGAFIVDGT  288
     aAt_Hog INWLH-HSPDREGEVLQIRHEAG------------QVQLTASHLLXVRKP-GRA--------------------------GAVPC--RADEVRLGDRLL----VPWIDGSIAEPAVLR--ID--H-TCKRGLYAP-LV--SCGTVFVDGT  110
 aAt_Hogfrag --------------------------------------------------------------------------------------------------------------------------HEGRAGLRGAHAPLTA---TGTLLVDGV   25
    aAt_Hog2 RAWLH-RDTASEVAMVRIRTD------------AGDFIASPFHSIAS---------------------------------GEDGAYTFATGLKPGDAVVT---------AKGPATVQSISAG--CRARM-PHSYPSGC---LGCLYLCDV  167
     aCp_Hog EIMLH-KDKNIYLDDEWIQVEYLGM--------EKPLVLSPNHLIFIQYL-GEKPHEGNCYYPSQIEKAIGIEVTPQLEKKKKLTSIQAKDIRVGDSVI--IYSKERVGWVTQVSIISNVNSNQKNYEYVGRYAP-LT--TDGYLIVNGV  293
     aCm_Hog IMMLH-NDPNFYPDDNWIIIKYLNV--------DIPLILSPNHLIF-KLDLGDFPQKGDCEINTTIFSHFHNIYINNKIDHFKVVSVLAKDIRIGDALIINSINGIIVSWITDINIYNVESQ--KYLQLKGRYSP-LT--KDGHLIVNNV  288
   crGt_Hog1 VIFKHDHKDVSSVISISYEDR--------------TLRLTPTHLIPKYSDKCGDS------------------------FCHLAKNVPASSIQAGDRIY----------VHSGEGFQAKIVS----AVSKSAAKVRYLLTENDRIVVDGI  333
   crGt_Hog2 TYFIHDHEKPSETLKISVGQE--------------EIEMTPSHFLPIYSAECGEK------------------------FCQFAKLVPAASVKPGDFLY----------TQCGFEMVKG--------VSSSRSFVRYLLVEGGNLMVNGV   97
    hPh_Hog3 FFQGHASKDASPYVRLSLESK-------------LTLELSPDHYLRLVNP-----------------------------HGELETHVLAKDAAVGMRLAVS--------AEAEAEVKTATVLQVERTVLAGAYNPYTT---SGTIIVNGV  258
    hPh_Hog2 YFQGHASKDASPYVRLSLDSK-------------HTLELSPDHYLRLVNL-----------------------------QCELETHVLAKDAAVGMRLAVS--------AEAEEACQTATVKQVERTVLAGAYNPYTT---SGTIIVNGI  325
    hPh_Hog1 YFQGHASKDASPYVRLSLDST-------------HTLELSPDHYLRLVNL-----------------------------QCELETHVLAKDAAVGMRLAVS--------AEAEEACQTATVKQVERTVLAGAYNPYTT---SGTIIVNGI  311
  fGm_GmGIN1 FLMIH-ADPNAVTKFQSIDFVKQDGS-------QGNLHITPKHHIF-VN-------------------------------NGETD--FANNVTTNTKLF----------VSDGEKFVTVLPIRVTKERRKGYYSPLTR---SGTILVDEV  138
   Mo_hoglet VSFMHVGEQLAPFVAIQAGNN--------------TIKLTALHLLYVVDA------------------------------KQGAELVKASEVRVGNTVI----------LADGEHAIVDAVS---IVEEEGFISV-LT--ASGQLAVDGI  130
       ruler .......310.......320.......330.......340.......350.......360.......370.......380.......390.......400.......410.......420.......430.......440.......450
```

```
jJl_Hog1       LGSSLESSG------------------------------------------------------------------------------------------------------------------------------ 292
jJl_Hog2       --------------------------------------------------------------------------------------------------------------------------------------- 251
jJl_Hog3       --------------------------------------------------------------------------------------------------------------------------------------- 121
cBn_Hog        RASSFAVNHGI----------------------------VNAFYH--I--------------------------------------------------------------------------------------- 210
Xx_104K18      LVSCYAEIESQ-------------------SLAHAAFEPIRWWHS--MMNSM--APEFLAVKLKSEKQSEGIHWYPASLFVMAGSLIDILTAKKKETVETEETTGLVAAESEDHLLFLMA--------------- 256
pSm_Hog        EASSHSEWFLDS------LFDAIGATEYLPHAYQAVLAPLRAIYN--IVGKEIYAEGYAAMDSCVNFAEFGAKYGGSIALTVGIAGVATAMLATKA--------------------------------------- 248
pPp_Hog        AASAHSEWFLDG------AFDALGLTHWIPSAYQMVLAPMRLAYYGLGKQAYLDLYLRLDALVDVAQFGTKFGGPVVTAAAGLGSSIIAALLIFKSSRPKTA--------------------------------- 481
pSl_Hog        --------------------------------------------------------------------------------------------------------------------------------------- 193
rGc_Hog2       VVSSYSRTIEP-------------------RLAHALLMPLRWLAR--ATGSK---EVLAGVFYEGGRGLERFLPKGQAQY------------------------------------------------------- 163
rPh_Hog        VASDLTTALPA-------------------ALASAALAPLRAAGV--AGWMA---PARVASRLLRRGCAPVVRAVAAARWSVGT--------------------------------------------------- 215
rGc_Hog3       VCSTYTHTIVP-------------------RVAHSLLAPLRLLHK--LVHML---TATSQKGFFVDSRAI----------------------------------------------------------------- 233
rGc_Hog4       VVSTFTRSVSA-------------------DAAHALLLPIRALYW--AGVVR---EKLAGKWFXAGG-------------------------------------------------------------------- 145
rGc_Hog6       RGRAAGVLCSAYT------------ARVPPRAAHALLAPLRWLHA--VRAAL----RHK---------------------------------------------------------------------------- 112
rCc_Hog2       --------------------------------------------------------------------------------------------------------------------------------------- 143
rGj_Hog        RASTFTKAVEI-------------------KMAHALMAPLRMMYN--AVGLS---ATFLDKGADTIMSMLPKGRTEL---------------------------------------------------------- 155
rGc_Hog1       RASTFTQAVQV-------------------CTASALMAPLRFAYA--LFGWS---ASFLNGGMESVANMLPDGAAAL---------------------------------------------------------- 229
rCc_Hog        VA------------------------------------------------------------------------------------------------------------------------------------- 153
rPy_Hog        AAFTFTTXVE----------------------------------------------------------------------------------------------------------------------------- 177
rPy_Hog2       --------------------------------------------------------------------------------------------------------------------------------------- 282
rPh_Hog2       --------------------------------------------------------------------------------------------------------------------------------------- 255
rGc_Hog5       LASTYTTAVE-------------------PTMAHALLAPLRAAYS--RLGFCMTAFDAGADMLASYMPSGSVVN------------------------------------------------------------- 66
aAc_Hog        VDASKLVLAHS---------------------FANIADPQRYERFWHGLLSVIQTIAPXFNXTRWKLTATTCIP------------------------------------------------------------- 177
aKm_Hog        VVSCYAIPDNITESLVFRKLAQITSARSMQAAYHALFLPLRVACR--LRCTLPISD------------------------------------------------------------------------------- 342
aAt_Hog        AASCYGLPDRLHASPLVQRVARLADGKGFHAAAHAVFLPLRLLHA--ACGERGGGPRHAKAAALPGAGEERKQLEPSSRKEAGAYPPLCLGSVHPGILLPDLSAGFRVVAVA----------------------- 220
aAt_Hogfrag    LCSCYAPPAAWAVPHE---------------ACHAALLPLRLLDSLRAAAERWSRPAGAKEPLLTVEAVWLLPRSADETVHPYASGLLRTAQAAKAAAACCRALLLSCRLAAVAEPGPAASAARPPAAAAAAAVR 145
aAt_Hog2       GCQ-------------------------------------------------------------------------------------------------------------------------------- 170
aCp_Hog        LVSSYSKPFPWPMELLNPSHNLIDLLARPIVNIEIHFKQIFDYTRNGVVK-ILRYISNISSKYTFDQSIALSLEIIRAISLFVK--------------------------------------------------- 376
aCm_Hog        FVSSFSKPYQWPVNIIQPNHLFLFKILKPFIFLQIYIYKISYIRT--LIQYIIKFVSYFSN-YKYDNSIPEILQLLRIFLAPISKKKYYLTIAYIIKLYLVIFLISNIFTMIFINKIYYNKYSLNYHNC------ 414
crGt_Hog1      LASVFSTAAEKFETLPFHLLDRFLPGVLQSKAVAATLETILESPILQGFETIVNSLSTLAVPRNANSNALLAATPFKSL-------------------------------------------------------- 412
crGt_Hog2      LASVQSTAAGALETLPFRFLDTIFPGSLEAPAIKQALRTVLESPMLRSFETLIARTHALPITIAPLLVSSSSSS------------------------------------------------------------- 171
hPh_Hog3       EVSCHSSWFLEG----------VTSAAATPLLYQQLLAPLRALYSVAPGLVKSFCAKFDGDSRPMSELGLRQIVGSLANIASA---------------------------------------------------- 331
hPh_Hog2       AASCHSSWFLEG----------VTSATATPLLYQQLLAPLRALYAVAPRLVKSFCANFDGDNRPMSELGLRQIVGSIVTSISA---------------------------------------------------- 398
hPh_Hog1       AASCHSSWFLEG----------VTSAAATPLLYQQLLAPLRALYAVAPRLVKSFCAKFDGDNRPMSELGLRQIVGSIFTSVSA---------------------------------------------------- 384
fGm_GmGIN1     LCSCYASAPPY------------------QALLNFVLVPLRMYTKIFPSNYLDKEIHPYVKFLYKGRWIMGCL-------------------------------------------------------------- 193
Mo_hoglet      VASSYTASKVY-------------PGEP-ISLRHALNQPLIWLAQAAPSLAAPMLSSEYHWYQQVTRAPRAMAHAFRSIAKAVEARMPALATVSCTGSL------------------------------------ 215
ruler          .......460.......470.......480.......490.......500.......510.......520.......530.......540.......550.......560.......570.......580.....
```

Motif J

Motif K
