# Supplementary material for: Evolution of hedgehog and hedgehog-related genes, their origin from Hog proteins in ancestral eukaryotes and discovery of a novel Hint motif
Source: BMC Genomics. 2008 Mar 11;9:127. doi: 10.1186/1471-2164-9-127 (PMC2362128; doi:10.1186/1471-2164-9-127)
Supplement: Additional file 15 — Multiple sequence alignment of the Hint region of VWA-Vint proteins with Hog domains of Hh proteins. [file 1471-2164-9-127-S15.pdf]

```
fGz_FG08272.1 GVCFAASTEVTL--------ASG-RI---VQMRKL-RR-GMK-VRT----------PR----------GSRRVALVLKTPVE-QEVLCRV-------------GDVLVTPWHPI---SSDS---------  69
fNc_NCU05555.1 NPCFAGETNVRV------------GV---AVAEDV-AADSDQ-LVT---KEIEISKLRRGMMVQTPKGFRKVRAVLKTPVCE-ERMCLVM-----ARDDEA--RQLLVTPWHPV----------------  86
fCg_CHGG_08787 NGCFAGCSPVLL-------AG-GKGL---VRINRL-RA-GME-VVT----------PC----------GPRKVAAVLRMPVR-RVEMCLVAAAAGPSGKGK--SRLLVTPWHPV-ELHQGG---------  83
fMg_MGG_09762 NPCFAGTTMVRL-------AGDG-GV---VPISWL-RA-GIS-VAT----------PA-----GPRAVRTVLRTRVR------SQPMVRL----------P--GGVVVTPWHPV---KMGGDGA------  74
aTt_00471620 GGCMHGDSLVQL--------SDS-SF---KMVKEV-KK-GDK-VIC----------PL-----LENQCVEVECVVLSKCEDG-TKEFVQL----------G--TDLWITPKHPI---RVNG---------  75
aTt_00696950 GGCIHGDSLVYL--------QNG-KT---KKVSEI-RK-GDV-VQC----------PE-----LGQLQSTEVVCIVVSKCENNSHSFVQI----------G--ENLWITPKHPI---RING---------  76
aTt_00214760 GGCIHGKCKVLL--------ENN-QY---KNVEEI-RK-GDI-VIC----------PR-----IGNKAVKVVCVVKTLCTGS-NHSFVKL-------------GNLLITPWHPV---RLMHEQQ------  77
aTt_0320310 NPCFEGNSEVKM--------ANG-TI---KKVKEI-KK-GDE-VFC----------PN-----TGKAEKVKCVIETEVKEN--LTQLVRL----------G--KGLLITPYHPV---RIQG---------  74
aTt_00070820 APCFDGNCLVKM--------ANG-DI---KKVMDI-RK-GDL-IAS----------PA-----INGVEAKVSCVVKTPCLNN-QAYFVEF----------E--DGLIITPYHPI---RVNG---------  75
fCt_PI-CtrI VGCFTKGTQVMM--------ADG-AD---KSIESI-EV-GDK-V-------------------MGKDGMPREVVGLPRG----YDDMYKV-------------RQLSSTRRN------------------  61
aCp_Hog NLCFPGNSLVIT--------RER-GE---IKLEDL-RI-GEY-VLI----------RD---LNTMKFKYSKVEIMLHKDKNI-YLDDEWI-----QVEYLGMEKPLVLSPNHLIFIQYLGEKPHEGNCYY  96
Mo_hoglet-1 FGCFSQSSTVVV--------EGR-GR---ISITDV-QP-GDM-LVD-----------------GSAAGTSQFVSFMHVGEQ--LAPFVAI-----QAGN----NTIKLTALHLL---YVVD---------  75
Xx_104K18 LLCVDKEALAIM--------SDG-KL---KPLAEL-QI-GDK-IKT----------LD---S-AGNMVDTEVIMFAHVGNDQ-KIMYNII-------TWSG--KSIRASPNHLI---PVAS---------  79
aKm_Hog LLCFPGDSTVVV--------RDR-GR---VPLAEL-KV-GDA-VLSVRRRHAPTKEVDEISCDGWELYFDEVLAFLHSDASM-EAEFLQV-----RHEA----GQLHLTPNHLL---FARSRTA------  96
Ts_Xhog3 PGCFPSDALVKT---------RS-RW---KTIQEL-HI-GDE-VLD----------LD---E-RGRPVYTEIFAWLRKDMLG--HSFVKI-----TTAD-G--ISLTLTPNHLI---FKLIPDCT-----  82
Sp_hh GGCFPGFSQASL--------ENG-RT---ISMLDI-RV-GDE-VAV----------VN---D-DGALDYSDVIMIVHRKLND-STLFYVI-----ETED-K--SVVQLTPQHLI---YVSETES------  83
Lv_hh GGCFPGFSQAYL--------KNG-RM---ISMLDI-RV-GDE-VAV----------VN---N-NGELDYSDVIMIVHRKLND-STLFYVI-----ETED-K--TIVQLTPQHLI---YVSERES------  83
Nv_140260 VYCFPESSTVQL--------SDN-HR---IPMKEL-KI-GDR-VKT----------LD---A-RGYHGYSDVIGFLHRVDGH-VIDYLSI-----KLAN-G--KHLRVSDKHLV---FKGK---------  80
Nv_87496 KRCFPGNGKVEL--------ESG-VQ---MYIRDL-RI-GDR-IKT----------VG---S-SGDVIYSDVIAFLHKNTTI-VVEFVAL-----HLTG-E--HRIIISAKHLI---FVSK---------  80
Nv_239508 KRCFPGNGKVEL--------ESG-VQ---MYIRDL-RI-GDR-IKT----------VG---S-SGDVIYSDVIAFLHKNTTI-VVEFVAL-----HLTG-E--HRIIISAKHLI---FVSK---------  80
XC_Hh LKCFPGHATVTT--------ANG-IR---KFMSDL-SV-GDMVAVS----------TG---HVNSDQYYQPIIAFLHRNTSM-PAKFVQI-----RTSPSG--KVFTASSEHLI---YVWN---------  83
pSm_Hog TSCFPGDATVQM--------YNG-DL---KLMRDL-EV-GDK-VAV------------------SKNVFSDIYAFGHKDADV-ISEFIQV-----HTAS----DMIELSEGHFI---PVDA---------  75
rPh_Hog GECFPAATTVRR--------RYG-GVVSTVRMDDL-RL-GDE-VLA-----------------AGGV-YTRVYLWSHADAAA-VTTFVRL----VATRADGGLHTLLISAGHLL---PTVVRGG------  86
Nv_95413hh TGCFPAASTARL--------ATG-EQ---VPMVDL-RL-GDK-VAS----------VD---D-RGGIIYSPVIMFLHRSPEL-IMDFLKI-----RTEN-Q--TELVITASHLI---YKIN---------  80
rCc_Hog AVCFPASARVQT--------ADG-RA---PRLDAL-AV-GDRVLVG----------VR-----RGRRVYSEVFAFTHADPHA-THAFVEL-----ATRPRN--HTLRLTEGHWL----------------  77
rGj_Hog -------------------------------------L-GDR--------------VH-----VGGGEFSPRVLFSHRDAST-RHAFVQV-----STAC-G--RSVAATHGHYL---YVNG---------  52
rCc_Hog2 SVCFPANALVEL--------ENG-AT---KMMSQV-QL-GDR-VRV------------------GPNDFSDVFMFTHKTAAI-KYSFVTL-----ATQS-G--HTLSLTKGHYL---YVNG---------  76
rPy_Hog SECFPATASVEV--------EGG-AT---KAMADL-AI-GDR-VRA----------AD--------GSFSDVFLFTHADPTA-KSTFVTL-----TTAA----GVLTVTPGHYV---LVNEGVR------  78
rPy_Hog2 PACFPATATVEL--------STG-AT---VAMADL-AV-GDR-VRV----------AA---G-AGAAAFSPVYTFTHRSSGG-AHPVVTA-----TTRS-G--HALTVTPGHLV---YING---------  80
rPh_Hog2 PACFPAAATVEL--------STG-AV---VAMADV-AV-GDA-VRV----------AA---G-AGAAAFSPVYTFTHRTAGG-AH---------------------------------------------  55
Nv_120428 LLCFPSAAQVEL--------DSG-ER---VAMSEL-RI-GDA-VKT----------TD---K-QGEAFYTKVVTFLQREPSH-LAKYYTL-----MTES-G--AQVTMTESHLI---FTKNLQV------  83
Acm_DY579185 QSCFPSTSKVEL--------KSG-KK---IAMNKL-EI-GDL-VKT----------YS---N-DGEIVFSPVITFLDQDIDY-KGHYYTI-----STAC-G--FKITLSEAHLI---FKITQTP------  83
Gb_Hh GGCFAAESTVQT---------PG-GL---CALAEL-RV-GRR-GAG----------AG---PGHGRLAFSPVLLFLDRDPA--PRTLLRV-----RTAS-G--RTLALTPSHLL---PVAR---------  79
fGm_GmGIN1 EGCFAADSKVIL--------KNG-KV---TKISEL-VI-GDYVCCG----------FE-----DGKQVYSEVFLMIHADPNA-VTKFQSI----DFVKQDGSQGNLHITPKHHI---FVNN---------  84
Ts_hh LNCFAGSSTVLT--------KEN-GR---KRMDEL-EI-GDR-VLA----------RK---A-DGKLTFSPVILFLHRDQQT-KAKFKAV-----HISN-D--TLLTISPDHLI---YKVDPMT------  83
Bf_AmphiHh GGCFSAESWVTR--------DDG-NR---IRMRDV-RP-GDK-VLS----------MD---S-GGHPVFSEVLTFMDRESRG-PWVYYTI-----HTDDRN--ITVTATPSHLV---FVTESRD------  84
Cap_Hh GGCFSADDTVKR--------VDG-SS---LPIQHL-RI-GDA-IQA----------ST---D-NGDVVYSPVILFLHREENA-VASFVTL-----KTEG-G--RSLTLSPSHLI---HTAEHGE------  83
Mm_Ihh GGCFPAGAQVRL--------ENG-ER---VALSAV-KP-GDR-VLA----------MG---E-DGTPTFSDVLIFLDREPNR-LRAFQVI-----ETQDPP--RRLALTPAHLL---FIADNHT------  84
Hs_IHH GGCFPAGAQVRL--------ESG-AR---VALSAV-RP-GDR-VLA----------MG---E-DGSPTFSDVLIFLDREPHR-LRAFQVI-----ETQDPP--RRLALTPAHLL---FTADNHT------  84
Mm_Dhh GGCFPGNATVRL--------RSG-ER---KGLREL-HR-GDW-VLA----------AD---A-AGRVVPTPVLLFLDRDLQR-RASFVAV-----ETERPP--RKLLLTPWHLV---FAARGPA------  84
Hs_DHH GGCFPGNATVRL--------WSG-ER---KGLREL-HR-GDW-VLA----------AD---A-SGRVVPTPVLLFLDRDLQR-RASFVAV-----ETEWPP--RKLLLTPWHLV---FAARGPA------  84
Mm_Shh GGCFPGSATVHL--------EQG-GT---KLVKDL-RP-GDR-VLA----------AD---D-QGRLLYSDFLTFLDRDEGA-KKVFYVI-----ETLEPR--ERLLLTAAHLL---FVAPHND------  84
Hs_SHH GGCFPGSATVHL--------EQG-GT---KLVKDL-SP-GDR-VLA----------AD---D-QGRLLYSDFLTFLDRDDGA-KKVFYVI-----ETREPR--ERLLLTAAHLL---FVAPHND------  84
Nv_241466hh GSCFHSEALVTI--------ENG-ER---IAIKDL-KT-GQR-VQS----------MD---E-TGRLLYSEVLIFLDYKPWLSKVPFTII-----ETDV----ANLALTRNHLI---FVMKRNS------  83
Pv_Hh GGCFPGTGVLQT---------ET-GW---KTMSQV-VA-GDS-VLS----------MN---S-NGKLEYSPVIAFIDRNERE-LERYITL-----HTED-K--KDITLTSKHLI---YMSTSNV------  82
Ob_Hh GGCFPRTGKVVV--------RNK-GT---ITLDQL-KV-GDS-VLS----------VDL----QGELTYSEVIAFLDTNKDS-SGYFHRI-----ETEN-G--HTIRLTGKHLIYSSYTNRTRF------  86
Tn_Hh GGCFDGDSTVRT---------EA-GP---KKMSDL-QV-GER-VQV----------AR---T-DGQTDYSEVILFLDRNETQ-QRLYNTL-----ETEN-G--RSITLTPTHLI---FTASPHQ------  82
Acm_hh TGCFSSESTVRL--------ENG-AV---LRVDQL-KI-SDR-VQV----------MM---Q-DGTIGYSEVIMFADYLPNI------------------------------------------------  53
Dh_Hh HGCFTPESTALL--------ESG-AE---KALGEL-AI-GDR-VLS----------MD---V-KGQPVYSEVILFMDRNLEQ-VENFVQL-----HTDG-G--AVLTVTPAHLI---SVWQPE-------  82
Dm_Hh HGCFTPESTALL--------ESG-VR---KPLGEL-SI-GDR-VLS----------MT---A-NGQAVYSEVILFMDRNLEQ-MQNFVQL-----HTDG-G--AVLTVTPAHLV---SVWQPE-------  82
Ag_Hh SGCFTGDSTVLT--------EAG-VH---RKLSEL-RI-GER-VQA----------VD---A-AGRTVFSEVLMFMDRDTHQ-RREFVTI-----EAEG-G--ALLKVTPAHLV---MVWRRE-------  82
Hm_CO905822 -------------------------------------------------------------K-TKKFLYSPVYMLAHKNDTK-KTIFLKI-----SCKN-G--SFVTLSSKHLV---Y-TDCY-------  48
Ts_Xhog2 ARCFHGDDWVLT---------TN-GR---MQMKHLLQK-KDAQVLT----------RS---E-NGHLEYSPVMTWIHAQKET-KAQFINL-----ETES-G--HRLSLTPLHMI---YQTNCD-------  83
XC_Shog2 GKCLYIDANVRM--------SDG-SM---KRVKDL-QI-GDE-VLA----------YN---E-AQGIHPSRIFGELHYDNET-MMKLVEI-----ETSS-G--RRIPVTPAHTV---FVRQCL-------  82
XC_Xhog5 RGCFHGADIVQT--------KEY-GN---MTIHELSKH-RDGRVLT----------RN---D-DGQLDYTPVRYWLHAQPTT-SMKFLIL-----HTES-G--HRLSITADHLI---YQTECR-------  84
Ts_Xhog1 ARCFHGDSIVQT---------EQ-GP---MQMKEALGK-SNLRVLA----------RD---A-DQNLVYSPITSWIHANKDR-STEFVQI-----VTDN-S--KKLLLTDLHLI---YESDCQ-------  83
XC_Thog GSCFHSTGTVQT--------AQY-GV---MSIGQLFHTYKDAHVLS----------RN---A-LGQLEYAPVEYWLHAEPTT-SEEFIEL-----GTEQ-G--HRLLLTPDHLI---YKTDCE-------  85
XC_XHog4 PRCFSGESTVFT--------EAY-GR---LTMGMLFSVYQDARPLA----------RN---D-QGQLEYTDVAYWLHAEPNV-RSPFLEI-----WTNS-S--HRLQLTPEHLI---YQTDCQ-------  85
XC_Shog1 ASCFHGSGSVQT--------EQY-GR---ITMSQVFHSHQDARVLT----------RN---D-KGQLEYTSIVYWLHAEPDR-NQSFLEI-----TTDT-S--HRLLLTAGHLI---YRTDCT-------  85
XC_Xhog2 ------------------------SI-------------------------------------DSKPIYTAVEWLYHKDPEM-QADFISI-----STKS-Q--KTIRMSPNHLI---PVVSCN-------  50
XC_Xhog3 PRCFSGNMMVST---------PN-GQ---KRMDRI-RA-GEE-VLV----------TL---T-DSKPIYEPIEWLYHMNPDI-EADLITL-----RTRS-G--RVLQLSHNHLI---PVVPCS-------  81
Ts_qua-1 GGCFSADMHVRT---------TH-SQ---IRMDQL-QL-DDI-VF------------------VDPVEQQPIFSMLHHDPAA-EVDFIII-----KTET-N--RSLSLTPNHLI---PIVPCR-------  77
XC_Xhog1 AGCFSADTIVHT---------PH-GE---KRMDQL-RV-GDH-VLS----------TASWDG-NSAISYQPITQFIHNDPLV-VAGYTVI-----STVS-G--RSLSLTPYHLL---PQFECH-------  84
Bm_qua-1 MNCFSADTKVYT---------QN-GE---KTMKDV-VV-GDF-VLV----------PV---S-KSQMRYERVEMFYHREPET-RAKFVVL-----ETES-G--RKLSLTELHLL---PLGDCK-------  81
Ce_qua-1 SNCFSADSLVTT---------VT-GQ---KRMDEL-QI-GDY-VLV----------PS---S-GNVLKYEKVEMFYHREPKT-RTNFVVL-----YTKS-G--RKLSLTGRHLL---PVAECS-------  81
Cb_qua-1 SNCFSADSLVTT---------VT-GQ---KRMDEL-QI-GDY-VLV----------PS---A-GNVLKYERVEMFYHREPKT-RTNFVVI-----FTKS-G--KKLSLTGRHLL---PVAECS-------  81
Cr_qua-1 SNCFSADSLVTT--------VT--GQ---KRMDEL-QI-GDY-VLV----------PS----AGNVLKYERVEMFYHREPKT-RTNFVMI-----FTKS-G--KKLSLTGRHLL---PVAECS-------  81
Ce_wrt-1 PACFTGNSKVMT---------PA-GE---KSMADL-SV-GDM-VMT----------YE-----YGKMTYTRVASWLHRLPDT-KAAFIKL-----TTEQ-G--AIIDMTPQHFI---YKANCV-------  80
Cb_wrt-1 PACFTGDAKVMT---------PS-GE---KTMSEL-RV-GDI-VQT----------Y----E-HGKMAYTRVASWLHRLPET-KAAFIKL-----TTDN-G--PEVSMTPQHFI---YKADCV-------  80
Pt_wrt ----------------------------------------------------------------------------------------------------------------------------------
Ce_wrt-7 YYCFPNDAVVNV--------YEK-AV---KRMDEL-EI-GDW-VEA----------LD---ENGEDITFLPVKYWLHRDPEQ-EAEFLEF-----SLDN-G--ETFTLTEKHLV---YTTECR-------  83
Ce_wrt-8 HYCFPADAEVNV--------YEK-GV---KRMDEL-EV-GDW-VQA----------LH-----GKETTYSPVKYWLHRDPEQ-EAEFVEF-----LLEN-G--ESFTLTEKHLV---FATDCQ-------  81
Ce_wrt-4 YYCFPGDAMVNV--------YNG-GF---KRMDEL-AV-GDW-VQA----------LD---KNGSQVTFIPVQYWLHRDPKQ-VADFVEF-----TLDN-G--ETFSLTEKHLV---FVTQCS-------  83
Cb_wrt-4 YYCFPADATVHV--------YDQ-GT---KRMDEL-EV-GDW-VQA----------FE---KNGETVTHVPVQYWIHRDQTQ-KATFIEF-----SLDN-G--EKFSLTEKHMV---YVSQCE-------  83
Ce_hog-1 HACFSTDSWMTTPSGKKRMTTPS-GK---KRMDQV-GI-GDL-VLT----------GN-----LTATYYTPIITWMHREPEN-RYNFYTI-----MTEY-G--KMLAVSAKHLI---YRNLCD-------  89
Cb_hog-1 HACFSTDSWMTT---------PT-GK---KRMDQV-AI-GDL-VLT----------GN-----LTATYYTPIISWMHREPDN-RYNFHTI-----MTEY-G--KMLAVSAKHLI---YRNLCD-------  80
Ce_grd-2 GGCFSSDTLVTT---------PS-GK---KRMDEI-DV-GDY-VLT----------AN-----RVKTHFTPVTLWIHRESEK-LEEFLTI-----TTER-G--STLQLTPLHFM---YRTKCN-------  80
Ce_grd-11 LTCFSRDTWVTT---------PS-GK---KRMDEI-EI-GDY-VLT----------AD-----LKTALFSAITLWIHREPET-VQEFLEI-----KTDN-G--KTLQLTAGHFI---YATECRYLP-SKN  86
Ce_grd-1 GACFSLDTWVTT---------PT-GK---KRMDQI-DI-GDY-VLT----------AD-----LEKTYFTPITLWIHREPEK-VQEFLTI-----MTEY-G--KTLRITSRHFM---YRNKCG-------  80
Cb_grd-1 GACFSLDTWVTT---------PS-GK---KRMDQI-DI-GDY-VLT----------AG-----LDETYFTPITLWIHREPER-VQEFLTI-----MTEY-G--KTLRMTARHFM---YRNKCG-------  80
Ce_wrt-6 MQCFSGDMEVET---------ED-GI---KMIKDL-KI-GDK-VLS----------MD-----EAFVTYSPVIMFLHKRDEE-IAEFNLI-----ETAN-G--HSIKLTDNHLI---YVSDCR-------  80
Cb_wrt-6 MQCFSGDMEVET---------ED-GV---KLIKDL-KI-GDK-VLS----------MD-----EAFVTYSPIIMFLHKRDDE-KAEFNLI-----ETSN-G--HSIKLTDNHLI---YVSDCN-------  80
Bm_wrt-6 LQCFSGDTTVQT---------PD-QI---KRIDEL-QV-GDQ-VLS----------IE-----ESLISYSPVVMFLHRSDNE-SAIFIKI-----TLEN-G--EIIKLTDYHLL---YVTSCA-------  80
Mi_wrt-6 LQCFSGDQLVNT---------PK-GE---KRMDEL-KI-GDL-VLS----------VD-----ESLVAYSPILMFLHRLPNE-KAIFKII-----TTLE-G--QQLKLTEFHLV---W-AGCS-------  79
ruler 1.......10........20........30........40........50........60........70........80........90.......100.......110.......120.......130
```

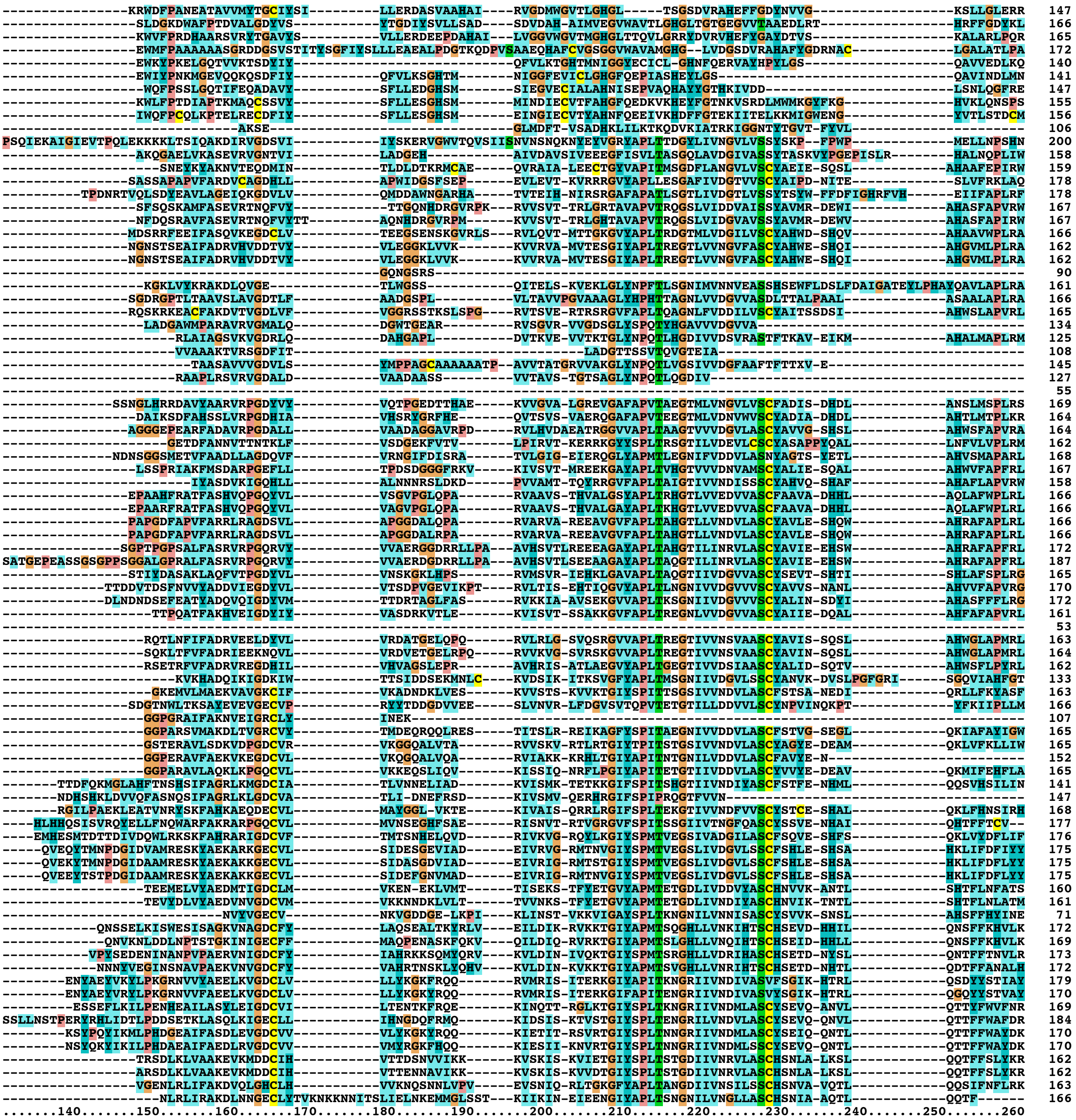
fGz_FG08272.1 147
fNc_NCU05555.1 166
fCg_CHGG_08787 165
fMg_MGG_09762 172
aTt_00471620 140
aTt_00696950 141
aTt_00214760 147
aTt_0320310 155
aTt_00070820 156
fCt_PI-CtrI 106
aCp_Hog 200
Mo_hoglet-1 158
Xx_104K18 159
aKm_Hog 178
Ts_Xhog3 178
Sp_hh 167
Lv_hh 167
Nv_140260 166
Nv_87496 162
Nv_239508 162
XC_Hh 90
pSm_Hog 161
rPh_Hog 166
Nv_95413hh 165
rCc_Hog 134
rGj_Hog 125
rCc_Hog2 108
rPy_Hog 145
rPy_Hog2 127
rPh_Hog2 55
Nv_120428 169
Acm_DY579185 164
Gb_Hh 164
fGm_GmGIN1 162
Ts_hh 168
Bf_AmphiHh 167
Cap_Hh 158
Mm_Ihh 166
Hs_IHH 166
Mm_Dhh 166
Hs_DHH 166
Mm_Shh 172
Hs_SHH 187
Nv_241466hh 165
Pv_Hh 170
Ob_Hh 172
Tn_Hh 161
Acm_hh 53
Dh_Hh 163
Dm_Hh 164
Ag_Hh 162
Hm_CO905822 133
Ts_Xhog2 163
XC_Shog2 166
XC_Xhog5 107
Ts_Xhog1 165
XC_Thog 165
XC_XHog4 152
XC_Shog1 165
XC_Xhog2 141
XC_Xhog3 147
Ts_qua-1 168
XC_Xhog1 177
Bm_qua-1 176
Ce_qua-1 175
Cb_qua-1 175
Cr_qua-1 175
Ce_wrt-1 160
Cb_wrt-1 161
Pt_wrt 71
Ce_wrt-7 172
Ce_wrt-8 169
Ce_wrt-4 173
Cb_wrt-4 172
Ce_hog-1 179
Cb_hog-1 170
Ce_grd-2 169
Ce_grd-11 184
Ce_grd-1 170
Cb_grd-1 170
Ce_wrt-6 162
Cb_wrt-6 162
Bm_wrt-6 163
Mi_wrt-6 166
ruler ....140.......150.......160.......170.......180.......190.......200.......210.......220.......230.......240.......250.......260

```
fGz_FG08272.1  SNGV---------------------------------VVGNGVERDESGLVMGFRQIHLLFVPCKSPIPNLPRTSLGQLFPKLL-------------------  198
fNc_NCU05555.1 VLRS-----------------------------------LERLQSSPDGLI---LGEGVDRDLQTGLVKGFRGKTSVHVDHHDD-------------------  212
fCg_CHGG_08787 AGGV-----------------AM---GAGLTRDPVTGKVIGFSAVKPEEVK-----ALGMRQQGRGVFA----------------------------------  209
fMg_MGG_09762  PKNG-----------------VATSAGVLRDATGRVIGFRVPDGGGEVRVG---TAAQGFDVVVRRSTV----------------------------------  221
aTt_00471620   MKGW-----------------------------------KEGKVIIRSRVR------DQITNQVKAFIQ----------------------------------  168
aTt_00696950   MKGW-----------------------------------KEGKVLITARIK-----SEITGKVTAFVQQ----------------------------------  170
aTt_00214760   GYVI-----------------------------------VNQQQTKRDKLT---HEVIGIQQNLEIKL-----------------------------------  177
aTt_0320310    IRDQ-----------------ATGLVVGLRSNRQWISKIQDLKYSSLSNIKQ--MSLNKLLQISKKLIKI---------------------------------  206
aTt_00070820   IRDP----------------------------------TTTLVIGLKQNIK---NQLSIENIQNYQIIV----------------------------------  188
fCt_PI-CtrI    -------------------------------------------EKTKTGIE--------LVKAKTKVFG----------------------------------  124
aCp_Hog        LIDL-----------------------LARPIVNIEIHFKQIFDYTRNGVV---KILRYISNISSKYTFDQSIALSLEIIRAISLFVK---------------  262
Mo_hoglet-1    LAQA-----------------AP---SLAAPMLSSEYHWYQQVTRAPRAMA---HAFRSIAKAVEARMPALATVSCTGSL-----------------------  215
Xx_104K18      WHSM------------------------MNSMAPEFLAVKLKSEKQSEGIH---WYPASLFVMAGSLIDILTAKKKETVETEETTGLVAAESEDHLLFLMA--  233
aKm_Hog        ITSA------------------------------------RSMQAAYHALF---LPLRVACRLRCTLPISDD-------------------------------  211
Ts_Xhog3       FYAF------------------------FKLVVKHQPTVDALFSPTYSGTH---WYVEIWKPFRSAIS-----------------------------------  219
Sp_hh          YSYI-----------------------RHNML-----GIVDTNTGQEQRVH---WYTQRLYKLGKYVMS-----------DRLFLGFDV--------------  214
Lv_hh          YTYI------------------S---HMLGIT--------DTDTGQEQRVH---WYTQGLYKLGKYVMS-----------DRLFPGFDV--------------  213
Nv_140260      WANV-----------------KA---AFGSFI------GWFPVSQPVSGIH---WYAESLISMVQMFSQLK--------------------------------  208
Nv_87496       WYDL-----------------WN---FFGHSF----SVFDMQNSVTGNEIH---WYAYALMK-ARFALP-----------NNLASFLGA--------------  212
Nv_239508      WYDL-----------------WN---FFGHSF----SVFDMQNSVTGNEIH---WYAYALMK-ARFALP-----------NNLASFLGA--------------  212
XC_Hh          -------------------------------------------------------------------------------------------------------   90
pSm_Hog        IYNI----------------------VGKEIYAEGYAAMDSCVNFAEFGAK---YGGSIALTVGIAGVA-----------TAMLATKA---------------  213
rPh_Hog        AGVA------------------------------------GWMAPARVASR---LLRRGCAPVVRAVAA-----------ARWSVGT----------------  203
Nv_95413hh     VGAI----------------------------------CPRCFDIEYSGIH---WYPRILLTIFGKIVELCGGFL----------------------------  203
rCc_Hog        -------------------------------------------------------------------------------------------------------  134
rGj_Hog        MYNA----------------------------------VGLSATFLDKGAD---TIMSMLPKGRTEL------------------------------------  155
rCc_Hog2       -------------------------------------------------------------------------------------------------------  108
rPy_Hog        -------------------------------------------------------------------------------------------------------  145
rPy_Hog2       -------------------------------------------------------------------------------------------------------  127
rPh_Hog2       -------------------------------------------------------------------------------------------------------   55
Nv_120428      FYSM----------------------------------APHYLGSGGTYLH---KYLKVVLRPVGIRVFGEEKFYKGPFEEYAKKN-----------------  218
Acm_DY579185   LY-----------------------------------------------------------------------------------------------------  166
Gb_Hh          WHWL-----------------------TAWGHAAPDYAHPPPPARAAPGVH---WYAKALYSLGQVPAPGHHALQVIRFHRLWCWRPSTCRPGEPRFALKAQL  241
fGm_GmGIN1     YTKI------------------------------------FPSNYLDKEIH---PYVKFLYK-GRWIMGCL--------------------------------  193
Ts_hh          YWNV-----------------------ASTIF----EQLGPTTAPTHYHIH---WYARWLWTLADNVSTFVGIPSPLDYFPRP--------------------  221
Bf_AmphiHh     YYQL-----------------------TSSLW-----DGPSHDQTLQEGVH---WYPSFFYRYGISLVE-----------PTLLHPTATDS------------  216
Cap_Hh         HYQV-----------------------------------LPVSDSPQEGVH---WYVQLLYDISTYVLP-----------SKMVFSPS---------------  197
Mm_Ihh         FPSL-----------------------------------AWGSWTPSEGVH---WYPQMLYRLGRLLLE-----------ESTFHPLGMSGAGS---------  211
Hs_IHH         FHSL-----------------------------------AWGSWTPGEGVH---WYPQLLYRLGRLLLE-----------EGSFHPLGMSGAGS---------  211
Mm_Dhh         LHAL--------------------------------GALLPGGAVQPTGMH---WYSRLLYRLAEELMG----------------------------------  200
Hs_DHH         LHAL--------------------------------GALLPGGAVQPTGMH---WYSRLLYRLAEELLG----------------------------------  200
Mm_Shh         AHAL-----------LAALAPARTDGGGGGSIPAAQSATEARGAEPTAGIH---WYSQLLYHIGTWLLD-----------SETMHPLGMAVKAS---------  241
Hs_SHH         AHALLAALAPARTDRGGDSGGGDRGGGGGRVALTAPGAADAPGAGATAGIH---WYSQLLYQIGTWLLD-----------SEALHPLGMAVKSS---------  267
Nv_241466hh    LRYW-----------------------LPSVF------SWLHEGITPAGVH---WFPRFLISLNQIVRI-----------AEFA-------------------  206
Pv_Hh          LHVL-------------------------SQYVPWLAPSTHHQNFTQNGVH---WYAKLLYNIGSTFLS-----------AETLHVP----------------  218
Ob_Hh          LHQV------------------------TSHIPFVSWAESPLASYAIDGIH---WYAKLLYKIAPLFLD-----------RTLLYMND---------------  222
Tn_Hh          IDNV---------------WEAT---LHLLRTMHILRYRESRTIPPHNGIH---WYANFLYSIAHKLIPED--------------------------------  211
Acm_hh         -------------------------------------------------------------------------------------------------------   53
Dh_Hh          LSTL-------------QSWMPA---KGQLRTAQDKSTPKDATAQQQNGLH---WYANALYKVKDYVLP-----------KSWRHD-----------------  219
Dm_Hh          LSTL-----------EAWLPAKE---QLHSSP------KVVSSAQQQNGIH---WYANALYKVKDYVLP-----------QSWRHD-----------------  216
Ag_Hh          AEKV-----------------SA---LFDRTD--------SLSLPRHEGIH---WYAKSLYTIKDYLIP-----------SNWLYH-----------------  206
Hm_CO905822    APLR-----------------------VACLV------FRK-KFQTNEEMP---KYIIALNQLGKRANL-----------VSKP-------------------  173
Ts_Xhog2       VYSL------------------------FTCPASLISDSFSHQQQDYVEIP---KLLLGALNLQKYLIQ----------------------------------  205
XC_Shog2       NRMY----------------IATTNIPHIQSIIQPIHAPTRIVTPTQHSPS---IYDSYILRICLLQPPI---------------------------------  217
XC_Xhog5       ------------------------------------------------------WGFEGNSSISEGS------------------------------------  120
Ts_Xhog1       LRRM----------------------LASILPEQLYEVMMFSTAVGDIKLP---SLLVGLIDISKHVIH----------------------------------  209
XC_Thog        VDWA----------------------GRNILPSTVYQALFRSDPINTAHVP---QILRSMWEISD--------------------------------------  205
XC_XHog4       -------------------------------------------------------------------------------------------------------  152
XC_Shog1       VEKT-------------------------------------LRGILPSSLY---KIIFRNQDLNRANVPR---------------------------------  195
XC_Xhog2       IRNR-----------------------MAKMW--SGVFGENVLAPEDKGIP---LPLQMLLKMAQFMLP-----------ANVFTV-----------------  188
XC_Xhog3       -------------------------------------------------------------------------------------------------------  147
Ts_qua-1       ISRM-----------------------LRNAL--------------FIQLP---IYLKSLYKLMHWTVS-----------MTVA-------------------  201
XC_Xhog1       -------------------------------------------------------------------------------------------------------  177
Bm_qua-1       LYRI-----------------------FGPLM--------QSLDEPIQHLP---TFIDSIHHLGRFAVP-----------FVKY-------------------  215
Ce_qua-1       VYNA-----------------------FGLLN---------TNHVDLQPIP---TFVSFAQYLSKTVLP-----------FS---------------------  211
Cb_qua-1       VYHA-----------------------FGLLN---------TNHVELQPIP---TFVSFAQYLSKTVLP-----------FS---------------------  211
Cr_qua-1       VYHA-----------------------FGLLN---------TNHVELQPIP---TFVSFAQYLSKTVLP-----------FS---------------------  211
Ce_wrt-1       VQQK-----------------------MRSVL---------GSLEETGHLP---ATSEFFLNIIDVLLP-----------HKY--------------------  197
Cb_wrt-1       MQQK-----------------------IRSLM---------GLFEETGHLP---VTSEFFLSIIDVLLP-----------HKY--------------------  198
Pt_wrt         INKI-----------------------ISSIATSFATKTGVVSEEETVELP---AVAKYLYDTLNLIIP-----------ETIFDGETRNMFMKH--------  129
Ce_wrt-7       WKNK-----------------------ITKYF---------WSYETERNIG---QSLNSLIAIFNLVVP-----------SNMY-------------------  210
Ce_wrt-8       LKNR-----------------------ISKYF-------WNEESNTEGNIG---TSLNFLIEIFELIVP-----------SKMISY-----------------  211
Ce_wrt-4       WKSQ-----------------------IRNYF------WTVEDSTNEDNIG---YGLNGVMAVLDIVIP-----------SKLM-------------------  214
Cb_wrt-4       FKNL-----------------------LMKFF-------GTADSTKEENLG---YGIHSLLDVVDLVLP-----------AKFV-------------------  212
Ce_hog-1       AQSW-----------------------LWIF---------GETVFHKATIP---IGSALASDVLRLVIP----------------------------------  213
Cb_hog-1       IQSW-----------------------LRLF---------GDSVFHTTAIP---VGSSLASDLLRLVVP----------------------------------  204
Ce_grd-2       LRQK-----------------------VLNLF--------GILHMNEIELP---TGTAVYKELLSLVIP-----------MGK--------------------  207
Ce_grd-11      LRNL-----------------------IVQYF--------GDLYLDEIELP---TGTSLYKEVLTLVIP-----------IRK--------------------  222
Ce_grd-1       LRSV-----------------------LVEFF--------GDLYNNKIELP---TGTTLSRDIISLIVP-----------IQK--------------------  208
Cb_grd-1       LRNK-----------------------LTEFF--------GDLYNKKIELP---TGTTLSKEIMSLVLP-----------IRK--------------------  208
Ce_wrt-6       TSSV-----------------------FHNLM-------FFKSSTEEGDLP---VGVETLTSVMDLFIP-----------QSFV-------------------  202
Cb_wrt-6       TSGV-----------------------FNSFA-------LFKTSQDDGSLP---VGVETLTSVMDLFIP-----------QSFV-------------------  202
Bm_wrt-6       FRYL------------------------------------ISTDQNTDGLL---PGIQFLTQISDLFLP-----------YSIV-------------------  197
Mi_wrt-6       -------------------------------------------------------------------------------------------------------  166
ruler          .......270.......280.......290.......300.......310.......320.......330.......340.......350.......360...
```
