# Supplementary material for: Evolution of hedgehog and hedgehog-related genes, their origin from Hog proteins in ancestral eukaryotes and discovery of a novel Hint motif
Source: BMC Genomics. 2008 Mar 11;9:127. doi: 10.1186/1471-2164-9-127 (PMC2362128; doi:10.1186/1471-2164-9-127)
Supplement: Additional file 17 — Multiple sequence alignment of the Hint region of the bacterial proteins with a novel Hint-like domain with Hog domains. Multiple sequence alignment of the bacterial Hint domains from Additional file 16 with Hog domains from animals. Note the roughly similar length. [file 1471-2164-9-127-S17.pdf]

```
                                                                                      .      .  :  *  .
Mo_hoglet-1        FGCFSQSSTVVVEGRGRISITDV---QPGDML-VDGS-AAGT-----SQFVSFMHVG--EQLAPFVAI---QAGN--NTIKLTALHLLYV---------------------------VDA   76
fGm_GmGIN1         EGCFAADSKVILKNGKVTKISEL---VIGDYV--CCGFEDGK--QVYSEVFLMIHADP-NAVTKFQSIDFVKQDGSQGNLHITPKHHIFVN-----------------------------   83
XC_Shog2           GKCLYIDANVRMSDGSMKRVKDL---QIGDEV-LAYN-EAQG--IHPSRIFGELHYDN-ETMMKLVEI---ETSS-GRRIPVTPAHTVFVR--QCL--------------------SD   84
Hm_CO905822        ----------------------------------------KTKK--FLYSPVYMLAHKND-TKKTIFLKI---SCKN-GSFVTLSSKHLVYT---DCY----------------------   48
Ts_Xhog3           PGCFPSDALVKT-RSRWKTIQEL---HIGDEV-LDLD-ERGR--PVYTEIFAWLRKDM--LGHSFVKI---TTAD-GISLTLTPNHLIFKLIPDCT---------------TPDNRTVQ   90
XC_Xhog2           -------------------------------------S-IDSK--PIYTAVEWLYHKDP-EMQADFISI---STKS-QKTIRMSPNHLIPVV--SCN------T-------TDFQKMGLAH   61
XC_Xhog3           PRCFSGNMMVST-PNGQKRMDRI---RAGEEV-LVTL-TDSK--PIYEPIEWLYHMNP-DIEADLITL---RTRS-GRVLQLSHNHLIPVV--PCS------N-------DHSHKLDVVQ   92
Ts_qua-1           GGCFSADMHVRT-THSQIRMDQL---QLDDIV-FV-----DP--VEQQPIFSMLHHDP-AAEVDFIII---KTET-NRSLSLTPNHLIPIV--PCR------R-------GILPAEKLEA   88
XC_Xhog1           AGCFSADTIVHT-PHGEKRMDQL---RVGDHV-LSTASWDGNSAISYQPITQFIHNDP-LVVAGYTVI---STVS-GRSLSLTPYHLLPQF--ECH---HLHH-------QSISVRQYEL   98
Bm_qua-1           MNCFSADTKVYT-QNGEKTMKDV---VVGDFV-LVPV-SKSQ--MRYERVEMFYHREP-ETRAKFVVL---ETES-GRKLSLTELHLLPLG--DCK---EMHE-------SMTDTTDIVD   95
Ce_qua-1           SNCFSADSLVTT-VTGQKRMDEL---QIGDYV-LVPS-SGNV--LKYEKVEMFYHREP-KTRTNFVVL---YTKS-GRKLSLTGRHLLPVA--ECS----QVE-------QYTMNPDGID   94
Cb_qua-1           SNCFSADSLVTT-VTGQKRMDEL---QIGDYV-LVPS-AGNV--LKYERVEMFYHREP-KTRTNFVVI---FTKS-GKKLSLTGRHLLPVA--ECS----QVE-------KYTMNPDGID   94
Cr_qua-1           SNCFSADSLVTT-VTGQKRMDEL---QIGDYV-LVPS-AGNV--LKYERVEMFYHREP-KTRTNFVMI---FTKS-GKKLSLTGRHLLPVA--ECS----QVE-------EYTSTPDGID   94
Ce_wrt-7           YYCFPNDAVVNVYEKAVKRMDEL---EIGDWV-EALDENGED--ITFLPVKYWLHRDP-EQEAEFLEF---SLDN-GETFTLTEKHLVYTT--ECR-----------------QNSSEL   89
Ce_wrt-8           HYCFPADAEVNVYEKGVKRMDEL---EVGDWV-QALH--GKE--TTYSPVKYWLHRDP-EQEAEFVEF---LLEN-GESFTLTEKHLVFAT--DCQ------------------QNVKN   86
Ce_wrt-4           YYCFPGDAMVNVYNGGFKRMDEL---AVGDWV-QALDKNGSQ--VTFIPVQYWLHRDP-KQVADFVEF---TLDN-GETFSLTEKHLVFVT--QCS---------------VPYSEDE   90
Cb_wrt-4           YYCFPADATVHVYDQGTKRMDEL---EVGDWV-QAFEKNGET--VTHVPVQYWIHRDQ-TQKATFIEF---SLDN-GEKFSLTEKHMVYVS--QCE----------------NNNYVE   89
Cb_hog-1           HACFSTDSWMTT-PTGKKRMDQV---AIGDLV-LTGN--LTA--TYYTPIISWMHREP-DNRYNFHTI---MTEY-GKMLAVSAKHLIYRN--LCD------------ENYAEYVRYL   90
Ce_grd-2           GGCFSSDTLVTT-PSGKKRMDEI---DVGDYV-LTAN--RVK--THFTPVTLWIHRES-EKLEEFLTI---TTER-GSTLQLTPLHFMYRT--KCN--------------ESSEFLKIL   89
Ce_grd-11          LTCFSRDTWVTT-PSGKKRMDEI---EIGDYV-LTAD--LKT--ALFSAITLWIHREP-ETVQEFLEI---KTDN-GKTLQLTAGHFIYAT--ECRYLPSKNSSLLNSTPERYRHLIDTL  104
Ce_grd-1           GACFSLDTWVTT-PTGKKRMDQI---DIGDYV-LTAD--LEK--TYFTPITLWIHREP-EKVQEFLTI---MTEY-GKTLRITSRHFMYRN--KCG------------KSYPQYIKML   90
Cb_grd-1           GACFSLDTWVTT-PSGKKRMDQI---DIGDYV-LTAG--LDE--TYFTPITLWIHREP-ERVQEFLTI---MTEY-GKTLRMTARHFMYRN--KCG------------NSYQKYIKIL   90
Ce_wrt-6           MQCFSGDMEVET-EDGIKMIKDL---KIGDKV-LSMD--EAF--VTYSPVIMFLHKRD-EEIAEFNLI---ETAN-GHSIKLTDNHLIYVS--DCR-----------------------T   81
Cb_wrt-6           MQCFSGDMEVET-EDGVKLIKDL---KIGDKV-LSMD--EAF--VTYSPIIMFLHKRD-DEKAEFNLI---ETSN-GHSIKLTDNHLIYVS--DCN-----------------------A   81
Bm_wrt-6           LQCFSGDTTVQT-PDQIKRIDEL---QVGDQV-LSIE--ESL--ISYSPVVMFLHRSD-NESAIFIKI---TLEN-GEIIKLTDYHLLYVT--SCA-----------------------V   81
Mi_wrt-6           LQCFSGDQLVNT-PKGEKRMDEL---KIGDLV-LSVD--ESL--VAYSPILMFLHRLP-NEKAIFKII---TTLE-GQQLKLTEFHLVWA---GCS------------------------   79
Ce_wrt-1           PACFTGNSKVMT-PAGEKSMADL---SVGDMV-MTYE--YGK--MTYTRVASWLHRLP-DTKAAFIKL---TTEQ-GAIIDMTPQHFIYKA--NCV------------------------   80
Cb_wrt-1           PACFTGDAKVMT-PSGEKTMSEL---RVGDIV-QTYE--HGK--MAYTRVASWLHRLP-ETKAAFIKL---TTDN-GPEVSMTPQHFIYKA--DCV------------------------   80
Ts_Xhog1           ARCFHGDSIVQTEQGPMQMKEAL---GKSNLRVLARD-ADQN--LVYSPITSWIHANK-DRSTEFVQI---VTDN-SKKLLLTDLHLIYES--DCQ------------------------   83
XC_Xhog5           RGCFHGADIVQTKEYGNMTIHELSK-HRDGRV-LTRN-DDGQ--LDYTPVRYWLHAQP-TTSMKFLIL---HTES-GHRLSITADHLIYQT--ECR------------------------   84
Ts_Xhog2           ARCFHGDDWVLT-TNGRMQMKHLLQ-KKDAQV-LTRS-ENGH--LEYSPVMTWIHAQK-ETKAQFINL---ETES-GHRLSLTPLHMIYQT--NCD------------------------   83
XC_Thog            GSCFHSTGTVQTAQYGVMSIGQLFHTYKDAHV-LSRN-ALGQ--LEYAPVEYWLHAEP-TTSEEFIEL---GTEQ-GHRLLLTPDHLIYKT--DCE------------------------   85
XC_Shog1           ASCFHGSGSVQTEQYGRITMSQVFHSHQDARV-LTRN-DKGQ--LEYTSIVYWLHAEP-DRNQSFLEI---TTDT-SHRLLLTAGHLIYRT--DCT------------------------   85
XC_XHog4           PRCFSGESTVFTEAYGRLTMGMLFSVYQDARP-LARN-DQGQ--LEYTDVAYWLHAEP-NVRSPFLEI---WTNS-SHRLQLTPEHLIYQT--DCQ------------------------   85
Nv_140260          VYCFPESSTVQLSDNHRIPMKEL---KIGDRV-KTLD-ARGY--HGYSDVIGFLHRVD-GHVIDYLSI---KLAN-GKHLRVSDKHLVFKG-----------------------------KMD   82
Nv_239508          KRCFPGNGKVELESGVQMYIRDL---RIGDRI-KTVG-SSGD--VIYSDVIAFLHKNT-TIVVEFVAL---HLTG-EHRIIISAKHLIFVS----------------------------KNG   82
Nv_120428          LLCFPSAAQVELDSGERVAMSEL---RIGDAV-KTTD-KQGE--AFYTKVVTFLQREP-SHLAKYYTL---MTES-GAQVTMTESHLIFT---KNL----------------QVSSNG   87
Acm_DY579185       QSCFPSTSKVELKSGKKIAMNKL---EIGDLV-KTYS-NDGE--IVFSPVITFLDQDI-DYKGHYYTI---STAC-GFKITLSEAHLIFKI-----------------------TQTPD   84
Ts_hh              LNCFAGSSTVLTKENGRKRMDEL---EIGDRV-LARK-ADGK--LTFSPVILFLHRDQ-QTKAKFKAV---HISN-DTLLTISPDHLIYKV--DPM---------------TNDNS   87
Nv_95413hh         TGCFPAASTARLATGEQVPMVDL---RLGDKV-ASVD-DRGG--IIYSPVIMFLHRSP-ELIMDFLKI---RTEN-QTELVITASHLIYK-----------------------INRQ   82
Pv_Hh              GGCFPGTGVLQT-ETGWKTMSQV---VAGDSV-LSMN-SNGK--LEYSPVIAFIDRNE-RELERYITL---HTED-KKDITLTSKHLIYMSTSNVT---------------TDDV   87
Ob_Hh              GGCFPRTGKVVVRNKGTITLDQL---KVGDSV-LSVD-LQGE--LTYSEVIAFLDTNK-DSSGYFHRI---ETEN-GHTIRLTGKHLIYS---SYT------------NRTRFDLNDN   91
Cap_Hh             GGCFSADDTVKRVDGSSLPIQHL---RIGDAI-QAST-DNGD--VVYSPVILFLHREE-NAVASFVTL---KTEG-GRSLTLSPSHLIHT------------------------------   78
Bf_AmphiHh         GGCFSAESWVTRDDGNRIRMRDV---RPGDKV-LSMD-SGGH--PVFSEVLTFMDRES-RGPWVYYTI---HTDDRNITVTATPSHLVFVT-------------------------ESRDL   85
Nv_241466hh        GSCFHSEALVTIENGERIAIKDL---TKGQRV-QSMD-ETGR--LLYSEVLIFLDYKPWLSKVPFTII---ETDV--ANLALTRNHLIFVM--KRN--------------------SST   85
Gb_Hh              GGCFAAESTVQT-PGGLCALAEL---RVGRRG-AGAGPGHGR--LAFSPVLLFLDRDP--APRTLLRV---RTAS-GRTLALTPSHLLPVA-------------------------RAG   81
Mm_Ihh             GGCFPAGAQVRLENGERVALSAV---KPGDRV-LAMG-EDGT--PTFSDVLIFLDREP-NRLRAFQVI---ETQDPPRRLALTPAHLLFIA------------------DNHTEP   86
Hs_IHH             GGCFPAGAQVRLESGARVALSAV---RPGDRV-LAMG-EDGS--PTFSDVLIFLDREP-HRLRAFQVI---ETQDPPRRLALTPAHLLFTA--DNH------------------TEP   86
Mm_Dhh             GGCFPGNATVRLRSGERKGLREL---HRGDWV-LAAD-AAGR--VVPTPVLLFLDRDL-QRRASFVAV---ETERPPRKLLLTPWHLVFAA--RGP--------------------APA   86
Hs_DHH             GGCFPGNATVRLWSGERKGLREL---HRGDWV-LAAD-ASGR--VVPTPVLLFLDRDL-QRRASFVAV---ETEWPPRKLLLTPWHLVFAA-----------------RGPAPA   86
Mm_Shh             GGCFPGSATVHLEQGGTKLVKDL---RPGDRV-LAAD-DQGR--LLYSDFLTFLDRDE-GAKKVFYVI---ETLEPRERLLLTAAHLLFVA----------------PHNDSGP   87
Hs_SHH             GGCFPGSATVHLEQGGTKLVKDL---SPGDRV-LAAD-DQGR--LLYSDFLTFLDRDD-GAKKVFYVI---ETREPRERLLLTAAHLLFVAPHNDS-------ATGEPEASSGSGPPSGG  102
Tn_Hh              GGCFDGDSTVRT-EAGPKKMSDL---QVGERV-QVAR-TDGQ--TDYSEVILFLDRNE-TQQRLYNTL---ETEN-GRSITLTPTHLIFTA-----------------------SPH   81
Dh_Hh              HGCFTPESTALLESGAEKALGEL---AIGDRV-LSMD-VKGQ--PVYSEVILFMDRNL-EQVENFVQL---HTDG-GAVLTVTPAHLISVW-----------------------QPE   82
Dm_Hh              HGCFTPESTALLESGVRKPLGEL---SIGDRV-LSMT-ANGQ--AVYSEVILFMDRNL-EQMQNFVQL---HTDG-GAVLTVTPAHLVSVW-----------------------QPE   82
Ag_Hh              SGCFTGDSTVLTEAGVHRKLSEL---RIGERV-QAVD-AAGR--TVFSEVLMFMDRDT-HQRREFVTI---EAEG-GALLKVTPAHLVMVW-----------------------RRE   82
Sp_hh              GGCFPGFSQASLENGRTISMLDI---RVGDEV-AVVN-DDGA--LDYSDVIMIVHRKL-NDSTLFYVI---ETED-KSVVQLTPQHLIYVS--ETE-----------------SS   84
Lv_hh              GGCFPGFSQAYLKNGRMISMLDI---RVGDEV-AVVN-NNGE--LDYSDVIMIVHRKL-NDSTLFYVI---ETED-KTIVQLTPQHLIYVS--ERE-----------------SN   84
bPl_plu1731        YGCIAEGTLIDMADGSKKKVEDI---RSGDKV-----------LTKQGGVLQVKSRIVGHDTEFVDL----IYNNDEKVSLTPTHPVATLR----------------------------   73
bSa_STIAU_1829     NSCMAAGTRIQMADGRILPVEQV---EIGDKVLANKE---------GLILTITDVARGHEIDDFVQL----HDNAGHRVTLTQMHPVIKAD---------------------GKV   78
bMx_MXAN_6253      NSCMAEGTQVKLADGTLAPVESV---KLGDKVIADAK---------GTVLTVTDVARGNEAKPLFRL----RDSVGHDVTLTEMHPVIMS---------------------TGKV   78
bRM_MED297_11140   YSCVAKGSLIAMADGSVKAIEDV---MIGDVV-----------LSDGKAMQVTDTSIGYEAEPMIAI----TDDLGHNVLLTRTHPVQTS----------------------------   72
bSp_Draf4685       YGCLAENTRILMQDGGEKLISDL---TIGEYV-----------LSRNNIPLRIEAITNGQERNFIEIETTQNSKGKIRTITSSLGHPFITS-----------------------------   76
bYi_YintA_01002283 YGCLGKGTPILMQDGKEKAIDDI---LIGEWV-----------QSQDGRALRVEDVITGAEQNYLEIEVRNSCGQLKTLITSLGHPFCTT-----------------------------   76
ruler              1.......10........20........30........40........50........60........70........80........90........100.......110.......120
```

```
     Mo_hoglet-1 KQGAELVKASEVRVGNTVILADGEHA------------------IVDAVS-IVEEEGFISVLTASGQLAVDGIVASSYTASKVYPGEP-----------ISLRH-ALNQPLIWLAQAA--   163
      fGm_GmGIN1 --NGETDFANNVTTNTKLFVSDGEK---------------FVTVLPIRVT-KERRKGYYSPLTRSGTILVDEVLCSCYAS-----APP----------YQALLN-FVLVPLRMYTKIF--   167
        XC_Shog2 GTNWLTKSAYEVEVGECVPRYYTDDGD-------------VVEESLVNVR-LFDGVSVTQPVTETGTILLDDVVLSCYNP-----VIN-----------QKPTY-FKIIPLLMNRMYI--   171
      Hm_CO905822 ----KVKHADQIKIGDKIWTTSIDDSEK------------MNLCKVDSIK-ITKSVGFYAPLTMSGNIIVDGVLSSCYAN-----VKDVSLPGFGRISGQVIAH-FGTAPLR--------   137
        Ts_Xhog3 LSDYEAVLAGEIQKGDVLVQMDDAWNG-------------ARHATVTEIH-NIRSRGAFAPATLSGTLIVDGTLVSSYTSYWFFDFIG-----------HRFVHEIIFAPLRFFYAFFKL   185
        XC_Xhog2 FTNSHSIFAGRLKMGDCIATLVNNE---------------LIADKVISMK-TETKKGIFSPITSHGTIIVNDIYASCFST-----FEN-----------HMLQQ-SVHSILINIRNRM--   146
        XC_Xhog3 FASNQSIFAGRLKLGDCVATLIDNE---------------FRSDKIVSMV-QERHRGIFSPIPRQGTFVVN-------------------------------------------------   147
        Ts_qua-1 TVNRYSKFAHKAEQDECVLMAYGGL---------------VKTEKIVAIS-QRRLRGIFSPLTEKGTIVVNDFVVSCYST-----CES-----------HALQK-LFHNSIRHISRML--   173
        XC_Xhog1 LFNQWARFAKRARPGQCVLMVNSEGH--------------FSAERISNVT-RTVGRGVFSPITSSGIIVTNGFQASCYSS-----VEN-----------HAIQH-TFFTCV---------   177
        Bm_qua-1 QWLRKSKFAHRARIGDCVFTMTSNHE--------------LQVDRIVKVG-RQYLKGIYSPMTVEGSIVADGILASCFSQ-----VES-----------HFSQK-LVYDFLIFLYRIF--   181
        Ce_qua-1 VAMRESKYAEKARKGECVLSIDESGE--------------VIADEIVRVG-RMTNVGIYSPMTVEGSLIVDGVLSSCFSH-----LES-----------HSAHK-LIFDFIYYVYNA---   179
        Cb_qua-1 AAMRESKYAEKAKKGECVLSIDASGD--------------VIADEIVRIG-RMTSTGIYSPMTVEGSLIVDGVLSSCFSH-----LES-----------HSAHK-LIFDFLYYVYHA---   179
        Cr_qua-1 AAMRESKYAEKAKKGECVLSIDEFGN--------------VMADEIVRIG-RMTNVGIYSPMTVEGSLIVDGVLSSCFSH-----LES-----------HSAHK-LIFDFLYYVYHA---   179
        Ce_wrt-7 KISWESISAGKVNAGDCFYLAQSEALTK------------YRLVEILDIK-RVKKTGIYAPMTSQGHLLVNKIHTSCHSE-----VDH-----------HILQN-SFFKHVLKWKNKI--   177
        Ce_wrt-8 LDDLNPTSTGKINIGECFFMAQPENASK------------FQKVQILDIQ-RVRKTGIYAPMTSLGHLLVNQIHTSCHSE-----IDH-----------HLLQN-SFFKHVLKLKNRISK   176
        Ce_wrt-4 NINANPVPAERVNIGDCFYIAHRKKSQM------------YQRVKVLDIN-IVQKTGIYSPMTSRGHLLVDRIHASCHSE-----TDN-----------YSLQN-TFFTNVLRWKSQ---   177
        Cb_wrt-4 GINSNAVPAEKVNVGDCFYVAHRTNSKL------------YQHVKVLDIN-KVKKTGIYAPMTSVGHLLVNRIHTSCHSE-----TDN-----------HTLQD-TFFANALHFKNLL--   177
        Cb_hog-1 PKGRNVVFAEELKVGDCLVLLYKGK---------------YRQQRVMRIS-ITERKGIFAPITENGRIIVNDIVASVYSG-----IKH-----------TRLQG-QYYSTVAYIQS----   173
        Ce_grd-2 PENHEAILASYLEIGDCVILTENTK---------------FRQEKINQTT-RGLKTGIYSPLTKNGRIIVNDMLASCYSE-----VQA-----------NVLQT-TYFWVFNRLRQKV--   174
       Ce_grd-11 PDDSETKLASQLKIGECLLIHNGDQ---------------FRMQKIDSIS-KTVSTGIYSPLTENGRILVNDVLASCYSE-----VQQ-----------NVLQT-TFFWAFDRLRNL---   188
        Ce_grd-1 PHDGEAIFASDLEVGDCVVVLYKGK---------------YRQQKIETIT-RSVRTGIYSPLTNNGRIIVNDMLASCYSE-----IQQ-----------NTLQT-TFFWAYDKLRSVL--   175
        Cb_grd-1 PHDAEAIFAEDLRVGDCVVVMYRGK---------------FHQQKIESII-KNVRTGIYSPLTNNGRIIVNDMLSSCYSE-----VQQ-----------NTLQT-TFFWAYDKLRNK---   174
        Ce_wrt-6 RSDLKLVAAKEVKMDDCIHVTTDSNV--------------VIKKKVSKIS-KVIETGIYSPLTSTGDIIVNRVLASCHSN-----LAL-----------KSLQQ-TFFSLYKRTSSVF--   167
        Cb_wrt-6 RSDLKLVAAKEVKMDDCIHVTTENNA--------------VIKKKVSKIS-KVVDTGIYSPLTSTGDIIVNRVLASCHSN-----LAL-----------KSLQQ-TFFSLYKRTSGVFNS   169
        Bm_wrt-6 GENLRLIFAKDVQLGHCLHVVKNQSNN-------------LVPVEVSNIQ-RLTGKGFYAPLTANGDIIVNSILSSCHSN-----VAV-----------QTLQQ-SIFNFLRKFRY----   166
        Mi_wrt-6 --NLRLIRAKDLNNGECLYTVKNKKNNITSLIELNKEMMGLSSTKIIKIN-EIEENGIYAPLTSNGNILVNGLLASCHSN-----IAA-----------QTLQQ-TF-------------   166
        Ce_wrt-1 TEEMELVYAEDMTIGDCLMVKENEK---------------LVMTTISEKS-TFYETGVYAPMTETGDLIVDDVYASCHNV-----VKA-----------NTLSH-TFLNFATSVQQKM--   165
        Cb_wrt-1 TEVYDLVYAEDVNVGDCVMVKKNNDK--------------LVLTTVVNKS-TFYETGVYAPMTETGDLIVNDIYASCHNV-----IKT-----------NTLSH-TFLNLATMMQQKI--   166
        Ts_Xhog1 GGPARSVMAKDLTVGRCVYTMDEQRQQ-------------LRESTITSLR-REIKAGFYSPITAEGNIVVDDVLASCFST-----VGS-----------EGLQK-IAFAYIGWLRRML--   170
        XC_Xhog5 GGPGRAIFAKNVEIGRCLYINEKWG---------------FEGN-------SSISEGS--------------------------------------------------------------   120
        Ts_Xhog2 -GKEMVLMAEKVAVGKCIFVKADNDK--------------LVESKVVSTS-KVVKTGIYSPITTSGSIVVNDVLASCFST-----SAN-----------EDIQR-LLFKYASFVYSLFTC   170
         XC_Thog GSTERAVLSDKVDPGDCVRVKGGQA---------------LVTARVVSKV-RTLRTGIYTPITSTGSIVVNDVLASCYAG-----YED-----------EAMQK-LVFKLLIWVDWA---   169
        XC_Shog1 GGPARAVLAQKLKPGQCVLVKKEQS---------------LIQVKISSIQ-NRFLPGIYAPITETGTIIVDDVLASCYVV-----YED-----------EAVQK-MIFEHFLAVEKTL--   170
        XC_XHog4 GGPERAVFAEKVKEGDCVLVKQGQA---------------LVQARVIAKK-KRHLTGIYAPITNTGNILVDDVLASCFAV-----YEN--------------------------------   152
       Nv_140260 SRRFEEIFASQVKEGDCLVTEEGSENSKG-----------VRLSRVLQVT-MTTGKGVYAPLTRDGTMLVDGILVSCYAH-----WDS-----------HQVAH-AAVWPLRAWANVKAA   173
       Nv_239508 NSTSEAIFADRVHVDDTVYVLEGGK---------------LVVKKVVRVA-MVTESGIYAPLTREGTLVVNGVFASCYAH-----WES-----------HQIAH-GVMLPLRAWYDLWNF   169
       Nv_120428 LHRRDAVYAARVRPGDYVYVQTPGEDT-------------THAEKVVGVA-LGREVGAFAPVTAEGTMLVNGVLVSCFAD-----ISD-----------HDLAN-SLMSPLRSFYSMA--   174
    Acm_DY579185 AIKSDFAHSSLVRPGDHIAVHSRYG---------------RFHEQVTSVS-VAERQGAFAPVTEEGTMLVDNVWVSCYAD-----IAD-----------HDLAH-TLMTPLKRLY-----   166
           Ts_hh GGSMETVFAADLLAGDQVFVRNGIFD--------------ISRATVLGIG-EIERQGLYAPMTLEGNIFVDDVLASNYAG-----TSY-----------ETLAH-VSMAPARLYWNV---   172
      Nv_95413hh SKRKEACFAKDVTVGDLVFVGGRSSTKS------------LSPGRVTSVE-RTRSRGVFAPLTQAGNLFVDDILVSCYAI-----TSS-----------DSIAH-WSLAPVRLVGAIC--   170
           Pv_Hh TDSFNVVYADDVIEGDYVLVTSDPVGEV------------IKPTRVLTIS-EHTIQGVYAPLTLNGNIVVDGVVVSCYAV-----VSN-----------ANLAH-VVFAPVRGLHVLSQ-   176
           Ob_Hh DSEFEATYADQVQIGDYVMTTDRTAG--------------LFASRVKKIA-AVSEKGVVAPLTKSGNIIVDGVVVSCYAL-----INS-----------DYIAH-ASFFFLRGLHQVTSH   179
          Cap_Hh -AEHGEIYASDVKIGQHLLALNNNRS--------------LDKDPVVAMT-TQYRRGVFAPLTAIGTIVVNDISSSCYAH-----VQS-----------HAFAH-AFLAPVRWHYQ----   161
      Bf_AmphiHh SSPRIAKFMSDARPGEFLLTPDSDGGG-------------FRKVKIVSVT-MREEKGAYAPLTVHGTVVVDNVAMSCYAL-----IES-----------QALAH-WVFAPFRLYYQLT--   172
     Nv_241466hh IYDASAKLAQFVTPGDYVLVNSKGK---------------LHPSRVMSVR-IEHKLGAVAPLTAQGTIIVDGVVASCYSE-----VTS-----------HTISH-LAFSPLRGLRYWLPS   172
           Gb_Hh GGEPEARFADAVRPGDALLVAADAGGA-------------VRPDRVLHVDAEATRGGVVAPLTAAGTVVVDGVLASCYAV-----VGS-----------HSLAH-WSFAPVRAWHWLTAW   171
          Mm_Ihh AAHFRATFASHVQPGQYVLVSGVPG---------------LQPARVAAVS-THVALGSYAPLTRHGTLVVEDVVASCFAA-----VAD-----------HHLAQ-LAFWPLRLFPSLA--   171
          Hs_IHH AARFRATFASHVQPGQYVLVAGVPG---------------LQPARVAAVS-THVALGAYAPLTKHGTLVVEDVVASCFAA-----VAD-----------HHLAQ-LAFWPLRLFHSLA--   171
          Mm_Dhh PGDFAPVFARRLRAGDSVLAPGGDA---------------LQPARVARVA-REEAVGVFAPLTAHGTLLVNDVLASCYAV-----LES-----------HQWAH-RAFAPLRLLHAL---   170
          Hs_DHH PGDFAPVFARRLRAGDSVLAPGGDA---------------LRPARVARVA-REEAVGVFAPLTAHGTLLVNDVLASCYAV-----LES-----------HQWAH-RAFAPLRLLHA----   169
          Mm_Shh TPGPSALFASRVRPGQRVYVVAERGGDRR-----------LLPAAVHSVTLREEEAGAYAPLTAHGTILINRVLASCYAV-----IEE-----------HSWAH-RAFAPFRLAHALLAA   179
          Hs_SHH ALGPRALFASRVRPGQRVYVVAERDGDRR-----------LLPAAVHSVTLSEEAAGAYAPLTAQGTILINRVLASCYAV-----IEE-----------HSWAH-RAFAPFRLAHALLAA   194
           Tn_Hh QTTPQATFAKHVEIGDYIYVASDRK---------------VTLEKVISVT-SSAKKGVFAPLTREGNLVVDGVVASCYAI-----IED-----------QALAH-FAFAPVRLIDNVWEA   168
           Dh_Hh RQTLNFIFADRVEELDYVLVRDATGE--------------LQPQRVLRLG-SVQSRGVVAPLTREGTIVVNSVAASCYAV-----ISS-----------QSLAH-WGLAPMRLLSTL---   167
           Dm_Hh SQKLTFVFADRIEEKNQVLVRDVETGE-------------LRPQRVVKVG-SVRSKGVVAPLTREGTIVVNSVAASCYAV-----INS-----------QSLAH-WGLAPMRLLST----   167
           Ag_Hh RSETRFVFADRVREGDHILVHVAGS---------------LEPRAVHRIS-ATLAEGVYAPLTGEGTIVVDSIAASCYAL-----IDS-----------QTVAH-WSFLPYRLAEKV---   166
           Sp_hh FSQSKAMFASEVRTNQFVYTTGQNHDRG------------VRPKRVVSVT-TRLGRTAVAPVTRQGSLVIDDVAISSYAV-----MRD-----------EWIAH-ASFAPVRWYSYI---   171
           Lv_hh FDQSRAVFASEVRTNQFVYTTAQNHDRG------------VRPMKVVSVT-TRLGHTAVAPVTRQGSLVIDGVAVSSYAV-----MRD-----------EWVAH-ASFAPIRWYTYI---   171
     bPl_plu1731 ----GIVKADELKIGDTIYTRDGQTTLSSVKL------RNTDPLNVYNFV-LEKTDDKSELLLEDALLFAGGILVGDNNL-----------------------------QRKVSIAN---   150
  bSa_STIAU_1829 VAARSLKIRDQVRTNRGIATIKSAKRVP------------VNGKQVFNLA-LGTPYELRTVGPQERTLFAGGFLVGDKSMQ-------------------DLLQNPQPPPMDVASSLP--   164
   bMx_MXAN_6253 VAAKNLKVKDQVRTVKGVSTLTSITPVS------------PDKMRVYNLR-LGTDPELLAVGKNGRTLYAGGFLVGDMTMQ------------------DELQAPKREPQVSLLNRLP--   165
bRM_MED297_11140 --NRGVIWAEEVQAGDQLMTQQGSSTVT-----------AVEEQLFNDTVHNLELEPASGTNETDELMFANGIAIGDLGYQSS----------------LTFKDKAPSTVEATLEQLP--   161
    bSp_Draf4685 ---TGVVIARELNHTSKLITLEGECEIISIEQ-------QQGELKVYNLQ-LTAEAPVDKGLYDDNTLYANGILVGDLQMQ------------------RIYEDEYYQRPVNILSKLP--   165
bYi_YintA_01002283 ---TGVELASELTLTSRLLTPESDCGINRIN-------KQHGEIEVYNLH-LSTDDPIKDLTESNSTMYAAGVLVGDSTMQ------------------YHYEEAYHHRSVNILNQLP--   165
           ruler .......130.......140.......150.......160.......170.......180.......190.......200.......210.......220.......230.......240
```

```
Mo_hoglet-1     ------------PS-LAAPMLS------------------SEYHWYQQVTRAPRAMAHAFRSIAKAVEARMPALATVSCTGSL----------  215
fGm_GmGIN1      ------------PSNYLD----------------------KEIHPYVKFLYK-GRW------IMGCL--------------------------  193
XC_Shog2        ------------ATTNIPHIQSII----------------QPIHAPTRIVTPTQHSPSIYDSYILRICLLQPPI-------------------  217
Hm_CO905822     ------------VACLVFRKKFQTN---------------EEMPKYIIALNQLGKRAN----LVSKP--------------------------  173
Ts_Xhog3        VVK---------HQPTVDALFSPTY---------------SGTHWYVEIWKPFRS-------AIS----------------------------  219
XC_Xhog2        ------------AKMWSGVFGENVLAPED-----------KGIPLPLQMLLKMAQF------MLPANVFTV----------------------  188
XC_Xhog3        ---------------------------------------------------------------------------------------------  147
Ts_qua-1        ------------RNALF-----------------------IQLPIYLKSLYKLMHW------TVSMTVA------------------------  201
XC_Xhog1        ---------------------------------------------------------------------------------------------  177
Bm_qua-1        ------------GP-LMQSLDEPI----------------QHLPTFIDSIHHLGRF------AVPFVKY------------------------  215
Ce_qua-1        ------------FG-LLNTNHVDL----------------QPIPTFVSFAQYLSKT------VLPFS--------------------------  211
Cb_qua-1        ------------FG-LLNTNHVEL----------------QPIPTFVSFAQYLSKT------VLPFS--------------------------  211
Cr_qua-1        ------------FG-LLNTNHVEL----------------QPIPTFVSFAQYLSKT------VLPFS--------------------------  211
Ce_wrt-7        ------------TK-YFWSYETE-----------------RNIGQSLNSLIAIFNL------VVPSNMY------------------------  210
Ce_wrt-8        ------------YF-WNEESNTE-----------------GNIGTSLNFLIEIFEL------IVPSKMISY----------------------  211
Ce_wrt-4        ------------IRNYFWTVEDSTNE--------------DNIGYGLNGVMAVLDI------VIPSKLM------------------------  214
Cb_wrt-4        ------------MK-FFGTADSTKE---------------ENLGYGIHSLLDVVDL------VLPAKFV------------------------  212
Cb_hog-1        ------------WLRLFGDSVFHT----------------TAIPVGSSLASDLLRL------VVP----------------------------  204
Ce_grd-2        ------------LN-LFGILHMNE----------------IELPTGTAVYKELLSL------VIPMGK-------------------------  207
Ce_grd-11       ------------IVQYFGDLYLDE----------------IELPTGTSLYKEVLTL------VIPIRK-------------------------  222
Ce_grd-1        ------------VE-FFGDLYNNK----------------IELPTGTTLSRDIISL------IVPIQK-------------------------  208
Cb_grd-1        ------------LTEFFGDLYNKK----------------IELPTGTTLSKEIMSL------VLPIRK-------------------------  208
Ce_wrt-6        ------------HNLMFFKSSTEE----------------GDLPVGVETLTSVMDL------FIPQSFV------------------------  202
Cb_wrt-6        ------------FA-LFKTSQDD-----------------GSLPVGVETLTSVMDL------FIPQSFV------------------------  202
Bm_wrt-6        ---------------LISTDQNT-----------------DGLLPGIQFLTQISDL------FLPYSIV------------------------  197
Mi_wrt-6        ---------------------------------------------------------------------------------------------  166
Ce_wrt-1        ------------RS-VLGSLEET-----------------GHLPATSEFFLNIIDV------LLPHKY-------------------------  197
Cb_wrt-1        ------------RS-LMGLFEET-----------------GHLPVTSEFFLSIIDV------LLPHKY-------------------------  198
Ts_Xhog1        ------------AS-ILPEQLYEVMMFSTAVGD-------IKLPSLLVGLIDISKH------VIH----------------------------  209
XC_Xhog5        ---------------------------------------------------------------------------------------------  120
Ts_Xhog2        ------------PASLISDSFSHQQQDY------------VEIPKLLLGALNLQKY------LIQ----------------------------  205
XC_Thog         ------------GRNILPSTVYQALFRSDPINT-------AHVPQILRSMWEISD--------------------------------------  205
XC_Shog1        ------------RG-ILP----------------------SSLYKIIFRNQDLNRA------NVPR---------------------------  195
XC_XHog4        ---------------------------------------------------------------------------------------------  152
Nv_140260       FGS---------FIGWFPVSQPV-----------------SGIHWYAESLISMVQM------FSQLK--------------------------  208
Nv_239508       ------------FGHSFSVFDMQNSVTG------------NEIHWYAYALMKARF-------ALPNNLASFLGA-------------------  212
Nv_120428       ------------PH-YLGSGG-------------------TYLHKYLKVVLRPVGIR-----VFGEEKFYKGPFEEYAKKN------------  218
Acm_DY579185    ---------------------------------------------------------------------------------------------  166
Ts_hh           ------------ASTIFEQLGPTTAPTH------------YHIHWYARWLWTLADNVSTFVGIPSPLDYFPRP--------------------  221
Nv_95413hh      ------------PR-CFDIEY-------------------SGIHWYPRILLTIFGK------IVELCGGFL----------------------  203
Pv_Hh           ------------YVPWLAPSTHHQNFTQ------------NGVHWYAKLLYNIGST------FLSAETLHVP---------------------  218
Ob_Hh           IP----------FVSWAESPLASYAI--------------DGIHWYAKLLYKIAPL------FLDRTLLYMND--------------------  222
Cap_Hh          ---------------VLPVSDSPQ----------------EGVHWYVQLLYDISTY------VLPSKMVFSPS--------------------  197
Bf_AmphiHh      ------------SSLWDGPSHDQTLQ--------------EGVHWYPSFFYRYGIS------LVEPTLLHPTATDS-----------------  216
Nv_241466hh     V-----------FS-WLHEGITP-----------------AGVHWFPRFLISLNQI------VRIAEFA------------------------  206
Gb_Hh           ------------GH-AAPDYAHPPPPARAA----------PGVHWYAKALYSLGQVPAPGHHALQVIRFHRLWCWRPSTCRPGEPRFALKAQL  241
Mm_Ihh          ------------WGSWTPS---------------------EGVHWYPQMLYRLGRL------LLEESTFHPLGMSGAGS--------------  211
Hs_IHH          ------------WGSWTPG---------------------EGVHWYPQLLYRLGRL------LLEEGSFHPLGMSGAGS--------------  211
Mm_Dhh          ------------GA-LLPGGAVQP----------------TGMHWYSRLLYRLAEE------LMG----------------------------  200
Hs_DHH          ------------LGALLPGGAVQP----------------TGMHWYSRLLYRLAEE------LLG----------------------------  200
Mm_Shh          LAP---------ARTDGGGGGSIPAAQSATEARGAEPT--AGIHWYSQLLYHIGTW------LLDSETMHPLGMAVKAS--------------  241
Hs_SHH          LAPARTDRGGDSGGGDRGGGGGRVALTAPGAADAPGAGATAGIHWYSQLLYQIGTW------LLDSEALHPLGMAVKSS--------------  267
Tn_Hh           ------------TLHLLRTMHILRYRESRTIPPH------NGIHWYANFLYSIAHK------LIPED--------------------------  211
Dh_Hh           ------------QS-WMPAKGQLRTAQDKSTPKDATAQQQNGLHWYANALYKVKDY------VLPKSWRHD----------------------  219
Dm_Hh           ------------LEAWLPAKEQLHSSPKVVSSAQQQ----NGIHWYANALYKVKDY------VLPQSWRHD----------------------  216
Ag_Hh           ------------SA-LFDRTDSLSLPRH------------EGIHWYAKSLYTIKDY------LIPSNWLYH----------------------  206
Sp_hh           ------------RHNMLGIVDTNTGQE-------------QRVHWYTQRLYKLGKY------VMSDRLFLGFDV-------------------  214
Lv_hh           -------------SHMLGITDTDTGQE-------------QRVHWYTQGLYKLGKY------VMSDRLFPGFDV-------------------  213
bPl_plu1731     ---------------------------------------------------------------------------------------------  150
bSa_STIAU_1829  ------------------------------------------ETWRPDFANAQAE--------------------------------------  177
bMx_MXAN_6253   ------------------------------------------KAWHTDFRNGVRSTQSR----------------------------------  182
bRM_MED297_11140 -----------------------------------------EAWHQDYLNSLK---------------------------------------  173
bSp_Draf4685    ------------------------------------------KEWHQDYQNHLNAAGK-----------------------------------  181
bYi_YintA_01002283 ---------------------------------------EEWRQDYQNHIKTIR------------------------------------  180
ruler           .......250.......260.......270.......280.......290.......300.......310.......320.......330...
```
