# Supplementary material for: Evolution of hedgehog and hedgehog-related genes, their origin from Hog proteins in ancestral eukaryotes and discovery of a novel Hint motif
Source: BMC Genomics. 2008 Mar 11;9:127. doi: 10.1186/1471-2164-9-127 (PMC2362128; doi:10.1186/1471-2164-9-127)
Supplement: Additional file 2 — Full image of the protein sequence logo of aligned Hog domains shown in Figure 1. The color scheme is similar to the one used in the multiple sequence alignments (N,Q,S,T: green; C: yellow; P: pink; G: orange; K,R: red; A,I,L,M,V: blue; F,W,Y: cyan blue; H, purple, D,E: magenta; gaps: white). The extend of the Hint domain and the SRR region are indicated above the logo with a red line. Red boxes underneath the logo indicate the different motifs A, B, F, J, K, L. [file 1471-2164-9-127-S2.pdf]

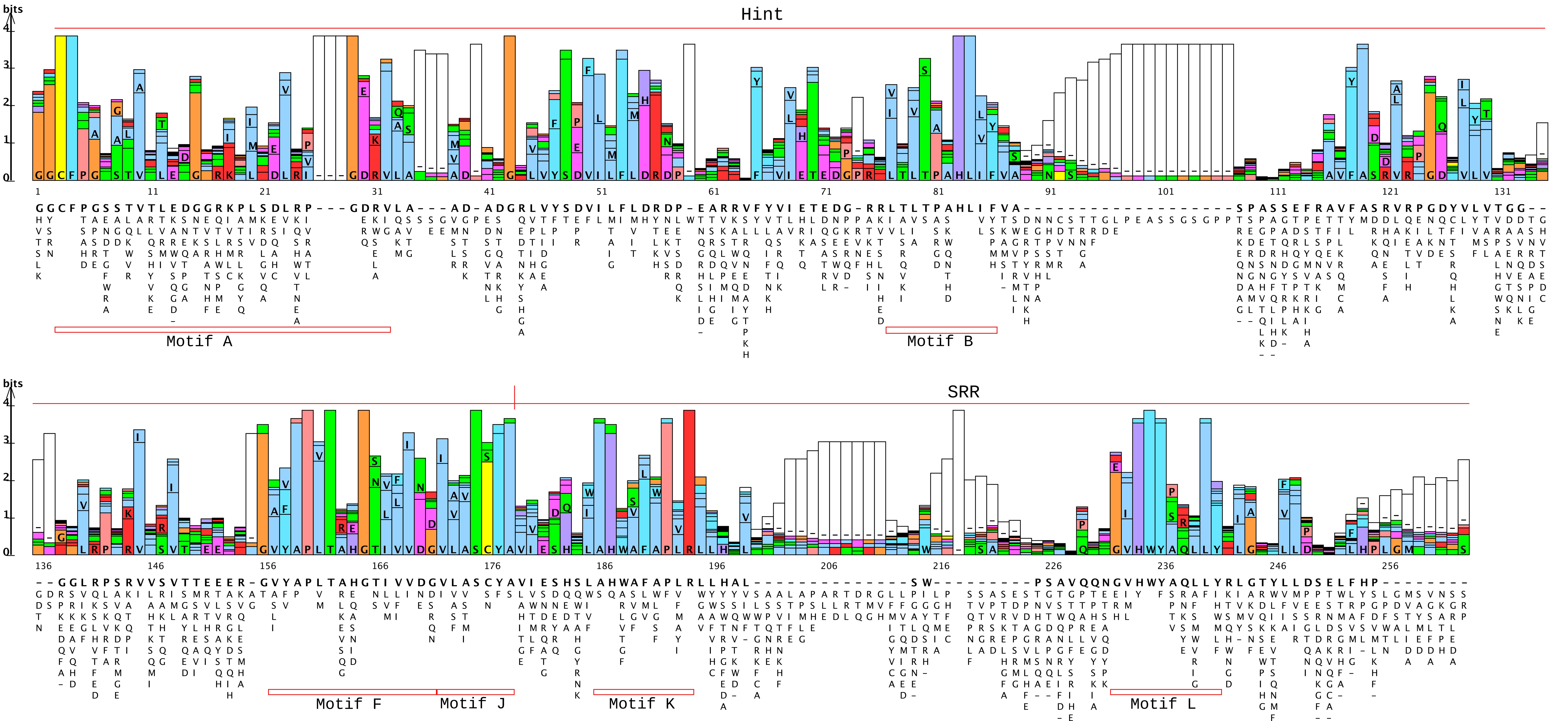
bits
Hint
Motif A
Motif B
bits
SRR
Motif F
Motif J
Motif K
Motif L
