# Supplementary material for: Evolution of hedgehog and hedgehog-related genes, their origin from Hog proteins in ancestral eukaryotes and discovery of a novel Hint motif
Source: BMC Genomics. 2008 Mar 11;9:127. doi: 10.1186/1471-2164-9-127 (PMC2362128; doi:10.1186/1471-2164-9-127)
Supplement: Additional file 3 — Protein sequence logo of nematode Hog domains. Protein sequence logo generated from nematode Hog domains shown in the multiple sequence aligment of Additional file 1. This logo is in register with the Hh Hog domain logo of Additional file 2. The two conserved cysteine residues specific to nematode Hh-related proteins are indicated with red arrows. [file 1471-2164-9-127-S3.pdf]

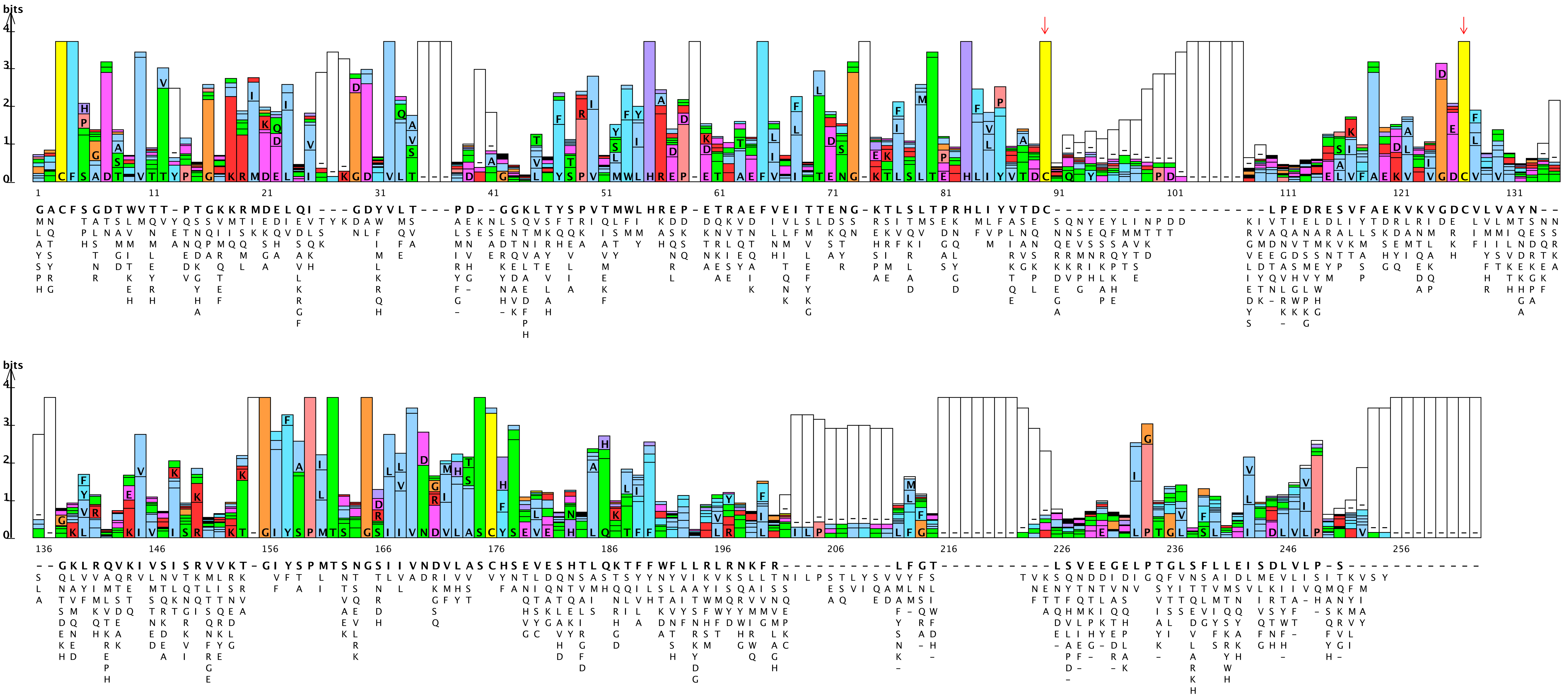
bits
4
3
2
1
0
1
11
21
31
41
51
61
71
81
91
101
111
121
131
bits
136
146
156
166
176
186
196
206
216
226
236
246
256
