# Supplementary material for: Evolution of hedgehog and hedgehog-related genes, their origin from Hog proteins in ancestral eukaryotes and discovery of a novel Hint motif
Source: BMC Genomics. 2008 Mar 11;9:127. doi: 10.1186/1471-2164-9-127 (PMC2362128; doi:10.1186/1471-2164-9-127)
Supplement: Additional file 16 — Multiple sequence alignment of bacterial proteins with a novel type of Hint-like region. Full length multiple sequence alignment of bacterial proteins. Motifs A and B are marked, as well as a small region with some similarity to the beginning of motif F in BIL-As. Note: the conserved upstream regions may be secreted, since some of the sequences have bacterial signal peptides for secretion. Species and the accession number for the sequences are: bSa_STIAU_1829: Stigmatella aurantiaca DW4/3-1 (ZP_01466308); bMx_MXAN_6253: Myxococcus xanthus DK 1622 (YP_634382); bRM_MED297_11140: Reinekea sp. MED297 (ZP_01113290); bPl_plu1731: Photorhabdus luminescens subsp. laumondii TTO1 (NP_929012); bSp_Draf4685: Serratia proteamaculans 568 (ZP_01534811); bYi_YintA_01002283: Yersinia intermedia ATCC 29909 (ZP_00833384). [file 1471-2164-9-127-S16.pdf]

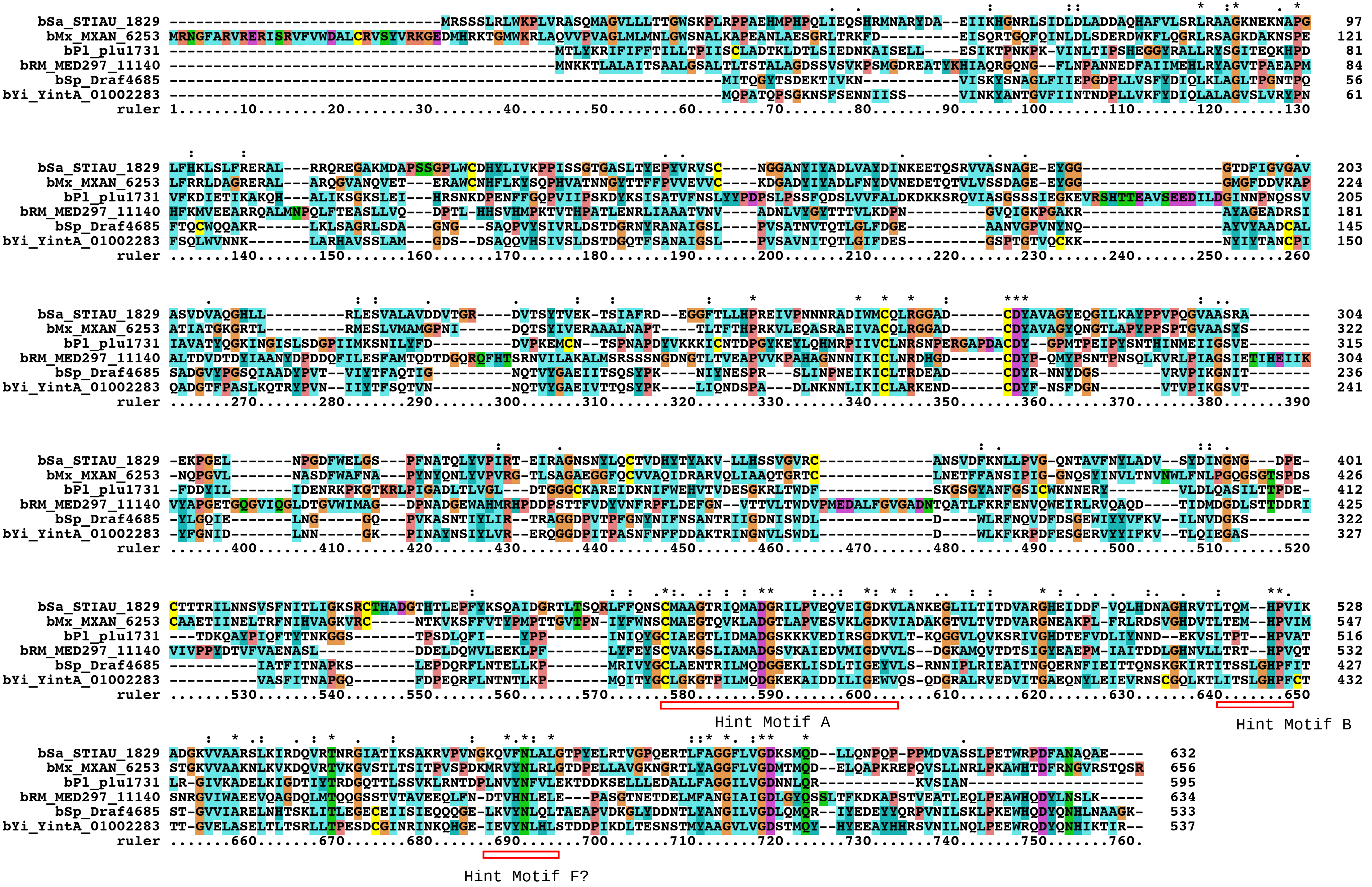

bSa_STIAU_1829 -------------------------------MRSSSLRLWKPLVRASQMAGVLLLTTGWSKPLRPPAEHMPHPQLIEQSHRMNARYDA--EIIKHGNRLSIDLDLADDAQHAFVLSRLRAAGKNEKNAPG 97
bMx_MXAN_6253 MRNGFARVRERISRVFVWDALCRVSYVRKGEDMHRKTGMWKRLAQVVPVAGLMLMNLGWSNALKAPEANLAESGRLTRKFD---------EISQRTGQFQINLDLSDERDWKFLQGRLRSAGKDAKNSPE 121
bPl_plu1731 --MTLYKRIFIFFTILLTPIISCLADTKLDTLSIEDNKAISELL----ESIKTPNKPK-VINLTIPSHEGGYRALLRYSGITEQKHPD 81
bRM_MED297_11140 --MNKKTLALAITSAALGSALTLTSTALAGDSSVSVKPSMGDREATYKHIAQRGQNG--FLNPANNEDFAIIMEHLRYAGVTPAEAPM 84
bSp_Draf4685 ---MITQGYTSDEKTIVKN-----------VISKYSNAGLFIIEPGDPLLVSFYDIQLKLAGLTPGNTPQ 56
bYi_YintA_01002283 ---MQPATQPSGKNSFSENNIISS------VINKYANTGVFIINTNDPLLVKFYDIQLALAGVSLVRYPN 61
ruler 1.......10........20........30........40........50........60........70........80........90.......100.......110.......120.......130

bSa_STIAU_1829 LFHKLSLFRERAL---RRQREGAKMDAPSSGPLWCDHYLIVKPPISSGTGASLTYEPYVRVSC----NGGANYIYADLVAYDINKEETQSRVVASNAGE-EYGG----------------GTDFIGVGAV 203
bMx_MXAN_6253 LFRRLDAGRERAL---ARQGVANQVET---ERAWCNHFLKYSQPHVATNNGYTTFFPVVEVVC----KDGADYIYADLFNYDVNEDETQTVLVSSDAGE-EYGG----------------GMGFDDVKAP 224
bPl_plu1731 VFKDIETIKAKQH---ALIKSGKSLEI---HRSNKDPENFFGQPVIIPSKDYKSISATVFNSLYYPDPSLPSSFQDSLVVFALDKDKKSRQVIASGSSSIEGKEVRSHTTEAVSEEDILDGINNPNQSSV 205
bRM_MED297_11140 HFKMVEEARRQALMNPQLFTEASLLVQ---DPTL-HHSVHMPKTVTHPATLENRLIAAATVNV----ADNLVYGYTTTVLKDPN---------GVQIGKPGAKR----------------AYAGEADRSI 181
bSp_Draf4685 FTQCWQQAKR------LKLSAGRLSDA---GNG---SAQPVYSIVRLDSTDGRNYRANAIGSL----PVSATNVTQTLGLFDGE---------AANVGPVNYNQ----------------AYVYAADCAL 145
bYi_YintA_01002283 FSQLWVNNK-------LARHAVSSLAM---GDS--DSAQQVHSIVSLDSTDGQTFSANAIGSL----PVSAVNITQTLGIFDES---------GSPTGTVQCKK----------------NYIYTANCPI 150
ruler ....140.......150.......160.......170.......180.......190.......200.......210.......220.......230.......240.......250.......260

bSa_STIAU_1829 ASVDVAQGHLL---------RLESVALAVDDVTGR----DVTSYTVEK-TSIAFRD--EGGFTLLHPREIVPNNNRADIWMCQLRGGAD------CDYAVAGYEQGILKAYPPVPQGVAASRA------- 304
bMx_MXAN_6253 ATIATGKGRTL---------RMESLVMAMGPNI------DQTSYIVERAAALNAPT----TLTFTHPRKVLEQASRAEIVACQLRGGAD------CDYAVAGYQNGTLAPYPPSPTGVAASYS------- 322
bPl_plu1731 IAVATYQGKINGISLSDGPIIMKSNILYFD---------DVPKEMCN--TSPNAPDYVKKKICNTDPGYKEYLQHMRPIIVCLNRSNPERGAPDACDY--GPMTPEIPYSNTHINMEIIGSVE------- 315
bRM_MED297_11140 ALTDVDTDYIAANYDPDDQFILESFAMTQDTDGQRQFHTSRNVILAKALMSRSSSNGDNGTLTVEAPVVKPAHAGNNNIKICLNRDHGD------CDYP-QMYPSNTPNSQLKVRLPIAGSIETIHEIIK 304
bSp_Draf4685 SADGVYPGSQIAADYPVT--VIYTFAQTIG---------NQTVYGAEIITSQSYPK----NIYNESPR---SLINPNEIKICLTRDEAD------CDYR-NNYDGS-------VRVPIKGNIT------- 236
bYi_YintA_01002283 QADGTFPASLKQTRYPVN--IIYTFSQTVN---------NQTVYGAEIVTTQSYPK----LIQNDSPA---DLNKNNLIKICLARKEND------CDYF-NSFDGN-------VTVPIKGSVT------- 241
ruler ....270.......280.......290.......300.......310.......320.......330.......340.......350.......360.......370.......380.......390

bSa_STIAU_1829 -EKPGEL-------NPGDFWELGS---PFNATQLYVPIRT-EIRAGNSNYLQCTVDHYTYAKV-LLHSSVGVRC-------------ANSVDFKNLLPVG-QNTAVFNYLADV--SYDINGNG---DPE- 401
bMx_MXAN_6253 -NQPGVL-------NASDFWAFNA---PYNYQNLYVPVRG-TLSAGAEGGFQCVVAQIDRARVQLIAAQTGRTC-------------LNETFFANSIPIG-GNQSYINVLTNVNWLFNLPGQGSGTSPDS 426
bPl_plu1731 -FDDYIL-------IDENRKPKGTKRLPIGADLTLVGL---DTGGGCKAREIDKNIFWEHVTVDESGKRLTWDF-------------SKGSGYANFGSICWKNNERY--------VLDLQASILTTPDE- 412
bRM_MED297_11140 VYAPGETGQGVIQGLDTGVWIMAG---DPNADGEWAHMRHPDDPSTTFVDYVNFRPFLDEFGN--VTTVLTWDVPMEDALFGVGADNTQATLFKRFENVQWEIRLRVQAQD----TIDMDGDLSTTDDRI 425
bSp_Draf4685 -YLGQIE-------LNG-----GQ---PVKASNTIYLIR--TRAGGDPVTPFGNYNIFNSANTRIIGDNISWDL-------------D----WLRFNQVDFDSGEWIYYVFKV--ILNVDGKS------- 322
bYi_YintA_01002283 -YFGNID-------LNN-----GK---PINAYNSIYLVR--ERQGGDPITPASNFNFFDDAKTRINGNVLSWDL-------------D----WLKFKRPDFESGERVYYIFKV--TLQIEGAS------- 327
ruler ....400.......410.......420.......430.......440.......450.......460.......470.......480.......490.......500.......510.......520

bSa_STIAU_1829 CTTTRILNNSVSFNITLIGKSRCTHADGTHTLEPFYKSQAIDGRTLTSQRLFFQNSCMAAGTRIQMADGRILPVEQVEIGDKVLANKEGLILTITDVARGHEIDDF-VQLHDNAGHRVTLTQM--HPVIK 528
bMx_MXAN_6253 CAAETIINELTRFNIHVAGKVRC-----NTKVKSFFVTYPMPTTGVTPN-IYFWNSCMAEGTQVKLADGTLAPVESVKLGDKVIADAKGTVLTVTDVARGNEAKPL-FRLRDSVGHDVTLTEM--HPVIM 547
bPl_plu1731 ---TDKQAYPIQFTYTNKGGS-------TPSDLQFI----YPP-------INIQYGCIAEGTLIDMADGSKKKVEDIRSGDKVLT-KQGGVLQVKSRIVGHDTEFVDLIYNND--EKVSLTPT--HPVAT 516
bRM_MED297_11140 VIVPPYDTVFVAENASL-----------DDELDQWVLEEKLPF-------LYFEYSCVAKGSLIAMADGSVKAIEDVMIGDVVLS--DGKAMQVTDTSIGYEAEPM-IAITDDLGHNVLLTRT--HPVQT 532
bSp_Draf4685 ----------IATFITNAPKS-------LEPDQRFLNTELLKP-------MRIVYGCLAENTRILMQDGGEKLISDLTIGEYVLS-RNNIPLRIEAITNGQERNFIEITTQNSKGKIRTITSSLGHPFIT 427
bYi_YintA_01002283 ----------VASFITNAPGQ-------FDPEQRFLNTNTLKP-------MQITYGCLGKGTPILMQDGKEKAIDDILIGEWVQS-QDGRALRVEDVITGAEQNYLEIEVRNSCGQLKTLITSLGHPFCT 432
ruler ....530.......540.......550.......560.......570.......580.......590.......600.......610.......620.......630.......640.......650
Hint Motif A
Hint Motif B

bSa_STIAU_1829 ADGKVVAARSLKIRDQVRTNRGIATIKSAKRVPVNGKQVFNLALGTPYELRTVGPQERTLFAGGFLVGDKSMQD--LLQNPQP-PPMDVASSLPETWRPDFANAQAE---- 632
bMx_MXAN_6253 STGKVVAAKNLKVKDQVRTVKGVSTLTSITPVSPDKMRVYNLRLGTDPELLAVGKNGRTLYAGGFLVGDMTMQD--ELQAPKREPQVSLLNRLPKAWHTDFRNGVRSTQSR 656
bPl_plu1731 LR-GIVKADELKIGDTIYTRDGQTTLSSVKLRNTDPLNVYNFVLEKTDDKSELLLEDALLFAGGILVGDNNLQR-----------KVSIAN-------------------- 595
bRM_MED297_11140 SNRGVIWAEEVQAGDQLMTQQGSSTVTAVEEQLFN-DTVHNLELE---PASGTNETDELMFANGIAIGDLGYQSSLTFKDKAPSTVEATLEQLPEAWHQDYLNSLK----- 634
bSp_Draf4685 ST-GVVIARELNHTSKLITLEGECEIISIEQQQGE-LKVYNLQLTAEAPVDKGLYDDNTLYANGILVGDLQMQR--IYEDEYYQRPVNILSKLPKEWHQDYQNHLNAAGK- 533
bYi_YintA_01002283 TT-GVELASELTLTSRLLTPESDCGINRINKQHGE-IEVYNLHLSTDDPIKDLTESNSTMYAAGVLVGDSTMQY--HYEEAYHHRSVNILNQLPEEWRQDYQNHIKTIR-- 537
ruler ....660.......670.......680.......690.......700.......710.......720.......730.......740.......750.......760.
Hint Motif F?
